# Prevalence of childhood anemia: Potential sociodemographic and dietary factors in Nigeria

**DOI:** 10.1371/journal.pone.0278952

**Published:** 2022-12-09

**Authors:** Jahid Hasan Shourove, Fariha Chowdhury Meem, Sabrina Akther Lima, G. M. Rabiul Islam

**Affiliations:** 1 Food Engineering and Tea Technology, Shahjalal University of Science and Technology, Sylhet, Bangladesh; 2 Rajshahi Medical College, Rajshahi, Bangladesh; 3 Harvard T.H. Chan School of Public Health, Harvard University, Cambridge, MA, United States of America; Università degli Studi di Milano, ITALY

## Abstract

**Background:**

Childhood anemia is a global public health issue. In this study, we assessed the potential sociodemographic and dietary factors associated with the prevalence of anemia among children aged 6–59 months in Nigeria.

**Methods:**

In this cross-sectional study, we collected dietary information and demographic data on 6,338 children with anemia from the Nigerian Demographic and Health Surveys (2018). The association between the occurrence of anemia and the demographic and dietary factors was determined by conducting Chi-squared tests. Additionally, bivariate and multivariate order logit models were constructed and reported as odds ratios.

**Results:**

The results of the multivariate analysis showed that the risk of anemia was reduced by 13% and 44% in children aged 13–36 months (OR = 0.87; 95% CI = 0.77–0.98; *p =* 0.019) and 37–59 months (OR = 0.56; 95% CI = 0.49–0.63; *p* < 0.001), respectively, compared to the risk of anemia in children aged 6–12 months. Anemia was 28% less likely in children of non-anemic mothers (OR = 0.72; 95% CI = 0.66–0.80; *p* < 0.001) than children of anemic mothers. Children fed pumpkin, carrot, squash, and sweet potato showed a lower occurrence of anemia by 17% (OR = 0.83; 95% CI = 0.70–0.99; *p* = 0.036) compared to those who were not fed these vegetables. Chances of anemia increased by 14% in children who were fed white potatoes, white yams, manioc, cassava, and other root-based foods (OR = 1.14; 95% CI = 1.01–1.29; *p =* 0.036).

**Conclusion:**

This study highlighted the impact of a plant-based diet on the high prevalence of childhood anemia in Nigeria. Therefore, reformation of dietary habits, the inclusion of nutritional supplements, and food-fortification programs with reductions in maternal anemia are recommended.

## Introduction

Anemia is a disorder characterized by a reduction in the volume of red blood cells (RBC) and is a major health issue among children, particularly in underdeveloped nations [1]. In 2019, the worldwide occurrence of anemia reached 39.8% among children aged 6–59 months [2]. Anemia negatively affects the physical and psychological development and immunity of children, as well as increases their susceptibility to infection and death [3, 4]. In developing countries, anemia has several causative factors, and it results from intricate problems that are difficult to identify [5]. Economic status, maternal education level, dietary habits, genetic disorders, digestion, complications related to absorption, and helminth infection in the intestinal tract are significant risk factors for anemia in children. Among them, micronutrient deficiency, including that of iron, folate, vitamin A, and vitamin B_12_, resulting from poor dietary habits, is a major cause of anemia [6, 7]. According to the World Health Organization (WHO), about 50% of cases of anemia occur due to iron deficiency [8]. Anemia might develop at any period of life, although it is most common in children, adolescents, and women throughout their reproductive period [9]. The intake of food during infancy and early childhood is much sensitive as it is related to the growth and development of children [10]. Complementary feeding starts in the early growth stage of children, and if the weaning meals contain insufficient bioavailable iron, iron reserves might get depleted. An imbalance between iron intake and higher iron demands due to their rapid growth rate might lead to anemia caused by iron deficiency [11]. According to the World Health Organization (WHO) and United Nations Children’s Emergency Fund (UNICEF), children should be exclusively breastfed for the first 6 months of age [12], and complementary foods (CFs) must be given to them immediately after this period with continued breastfeeding until at least 2 years of age [10, 13]. Both early and late introduction of CFs has been found to be associated with potential negative health outcomes [10]. Infants who are exclusively breastfed for more than 6 months in developing countries may be at increased risk of anemia [14]. Breast milk continued with complementary foods helps to prevent iron and vitamin A deficiency among children during their weening period [15]. Furthermore, if the mother has anemia or excessive blood loss during delivery, it may cause anemia among the mothers, followed by the children during the weaning period, as the breast milk encompasses somewhat low iron [16]. Effective breastfeeding can reduce worldwide under-5 mortality by 13%, and CF reduces by an additional 6% [17]. Besides dietary factors, parental education, family income, and the number of children in the family are also important determinants of nutritional knowledge and diet [18, 19]. Although the important risk factors for the occurrence of anemia have been identified in many countries, the associated reasons might change depending on the locality and time [5].

Nigeria is a sub-Saharan African country, where a large proportion (68.9%) of under 5 years children were affected by anemia in 2019 [2]. In this study, we identified the sociodemographic and dietary factors associated with the occurrence of anemia in Nigerian children (6–59 months) as they are more prone to anemia due to their high nutritional demand, and complementary feed is introduced after 6 months that may cause a shock of complementary food digestion and nutrient absorption. To the best of our knowledge, this is the first cross-sectional study to examine the association of demographic factors and the diets of individuals with the occurrence of anemia using representative data of a country from the Demographic Health Survey. The information obtained from the results of this study on the risk factors for anemia might help to establish integrated, timely, and appropriate disease management programs in Nigeria.

## Methods

### Data collection and sampling

**Fig 1:**
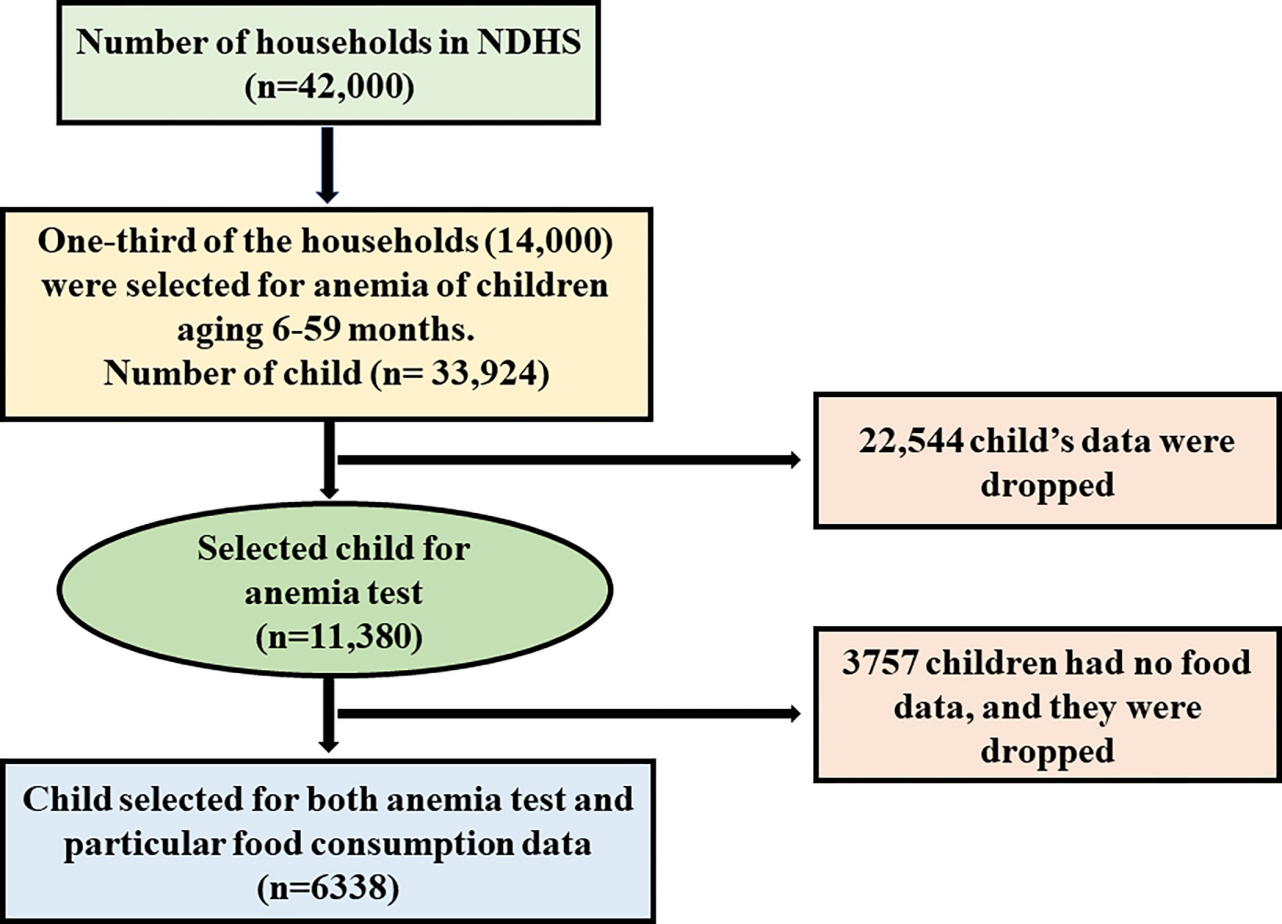
A flowchart of the data sampling process.

The data used in this study were obtained from the 2018 Nigerian Demographic and Health Surveys (NDHS, 2018). The sampling frame used for the 2018 NDHS was the Population and Housing Census of the Federal Republic of Nigeria (NPHC), which was conducted in 2006 by the National Population Commission. The NDHS is a nationally representative health survey that was conducted using a stratified two-stage sampling strategy from August 14 to December 29, 2018 [12]. It was a Nigerian cross-sectional survey that collected information on primary health aspects, such as nutrition, food, and the health of children, men, and women. The status of anemia was assessed by measuring hemoglobin levels with the HemoCue rapid test, which involved collecting a drop of capillary blood from the fingertip or heel in a microcuvette, followed by analyzing the hemoglobin content with a battery-operated portable HemoCue analyzer [12]. One-third of the households surveyed had their children tested for anemia between the ages of 6 and 59 months (n = 11,380). Among them, the demographic and dietary data of 6,338 children with anemia were analyzed for this study. The entire sampling method is presented in **Fig 1.**

Consent for obtaining and testing the blood samples of the children was given by a parent or an adult responsible for the children. The data collection procedures were approved by the National Health Research Ethics Committee of Nigeria and the ICF Institutional Review Board. Further details are provided in the final report of the DHS. Approval to use the data was obtained from the DHS program [12].

### Outcomes

In this study, the status of anemia among children aged 6–59 months was used as the outcome. For the diagnosis of anemia, the WHO guidelines were adopted by the Demographic Health Surveys, and we also followed similar guidelines in this study [2, 12]. The anemia status was grouped into four classes based on the hemoglobin level (adjusted for altitude): (I) Non-anemic (≥11.0 g/dL), (II) Mildly anemic (10.0–10.9 g/dL), (III) Moderately anemic (7.0–9.9 g/dL), and (IV) Severely anemic (<7.0 g/dL). The categorization of anemia in the severe, moderate, and mild categories was based on blood hemoglobin cutoffs (adjusted for altitude and smoking), as suggested by the Centers for Disease Control and Prevention of the United States [20]; this classification was also accepted by the WHO [21], and the Nigerian Demographic and Health Surveys [12].

### Explanatory variables

Some demographic and socioeconomic data and the food provided to the children were used as explanatory variables to determine their association with anemia in children. Maternal Education (no education, primary, secondary, or higher education), sex of the child (male or female), religion (Christianity, Islam, or others), wealth index (poor, middle, or rich), residence (urban or rural), currently breastfeeding (yes or no), child age (0–12 months, 13–36 months, or 37–59 months), maternal age (15–29 years or 30–49 years), and maternal anemia status (anemic or non-anemic) were considered potentially important factors associated with anemia in children and were included in this analysis.

The wealth index was calculated using the household score determined via principal component analysis based on the number and kinds of consumer goods, ranging from a television to a bicycle or car, and housing characteristics, such as the source of drinking water, toilet facilities, and flooring materials. The combined wealth index was measured and classified as: poorest, poorer, middle, richer, and richest [12]. These data were regrouped as poor (poorest and poorer), middle, and rich (richer and richest) in this study.

The dietary information was collected based on a 24-hour recall system. The parents were asked whether they gave their children any of the food classes on the previous day: (I) fortified baby food (Cerelac, Nutren, Frisolac H, Weatabix, etc.); (II) tinned powder or fresh milk of any animal rather than breast milk; (III) fruit juice; (IV) infant formula; (V) soup; (VI) bread, rice, noodles, porridge, or other foods made from grains; (VII) potatoes, cassava, or other tubers; (VIII) eggs; (IX) any meat (beef, pork, lamb, goat, chicken, or duck); (X) pumpkin, carrots, squash, or sweet potatoes (yellow or orange inside); (XI) any dark green leafy vegetables; (XII) ripe mangoes, papayas, other fruits containing vitamin A; (XIII) any other fruit; (XIV) liver, heart, other organ meat; (XV) fresh or dried fish or shellfish; (XVI) food made from beans, peas, lentils, or nuts; (XVII) cheese or other food made from milk; (XVIII) plane water. A score of “1” was given if yes and “0” if no.

### Ethical consideration

The NDHS data collection protocol was approved by the National Health Research Ethics Committee of Nigeria (NHREC) and the ICF Institutional Review Board [12]. All respondents were asked for consent before the interview, as per NDHS standards, following an oral explanation by the interviewers. The participants were informed that their participation was voluntary, the possible risks of participation, the aim of the survey, and the confidentiality of their information. Further ethical approval was not needed as we obtained approval for accessing and using the data from the DHS Program.

### Statistical analysis

The data were analyzed using the STATA software (version 16.0, Stata Corp LLC) [22]. The Chi-squared test was performed to determine the relationship between anemia and some of the demographic and dietary factors. The association between the outcome variable (anemia occurrence in Nigeria) and the explanatory variables (demographic factors and the food provided to the child) was determined by constructing the order logit model (fitted in the “ologit” command) using the maximum likelihood estimate.

## Results

### Descriptive analysis

In total, 6,338 Nigerian children aged 6–59 months were included in this study. The number and percentage of children belonging to each anemia class were categorized by the selected explanatory variables, and the results of the Chi-squared test are summarized in **Tables 1 and 2**.

**Table 1:** The percentage of children with anemia among Nigerian children (0–59 months) classified by socioeconomic and demographic factors *(N = 6*,*338)*.

Variables	n (%)	Anemia status (%)				χ^2^ value	*p*-value
			Anemia		Not anemia		
		Severe	Moderate	mild			
***Educational status***							
No education	2388 (37.68)	119 (4.98)	1102 (46.15)	617 (25.84)	550 (23.03)		
Primary	1057 (16.68)	31 (2.93)	457 (43.24)	302 (28.57)	267 (25.26)	202.13	*<0*.*001*
Secondary	2333 (36.81)	56 (2.40)	901 (38.62)	661 (28.53)	715 (30.65)		
Higher	560 (8.84)	1 (0.18)	132 (23.57)	171 (30.54)	256 (45.71)		
***Sex of child***							
Male	3210 (50.65)	115 (3.58)	1390 (43.30)	865 (26.95)	840 (26.17)	21.91	*<0*.*001*
Female	3128 (49.35)	92 (2.94)	1202 (38.43)	886 (28.32)	948 (30.31)		
***Religion***							
Christian	2933 (46.28)	74 (2.52)	1142 (38.94)	838 (28.57)	879 (29.97)		
Islam	3346 (52.79)	128 (3.83)	1432 (42.80)	890 (26.60)	896 (26.78)	31.76	*<0*.*001*
Others	59 (0.93)	5 (8.47)	18 (30.51)	23 (38.98)	13 (22.03)		
***Wealth index***							
Poor	2571 (40.56)	107 (4.16)	1170 (45.51)	697 (27.11)	597 (23.22)	99.26	*<0*.*001*
Middle	1220 (19.25)	42 (3.44)	521 (42.70)	324 (26.56)	333 (27.30)		
Rich	2547 (40.19)	58 (2.28)	901 (35.37)	730 (28.66)	858 (33.69)		
***Residence***							
Urban	2426 (38.28)	34 (1.40)	897 (36.97)	689 (28.40)	806 (33.22)	92.48	*<0*.*001*
Rural	3912 (61.72)	173 (4.42)	1695 (43.33)	1062 (27.15)	982 (25.10)		
***Currently breast feeding***							
No	1530 (24.14)	41 (2.68)	568 (37.12)	442 (28.89)	479 (31.31)	17.16	*0*.*001*
Yes	4808 (75.86)	166 (3.45)	2024 (42.10)	1309 (27.23)	1309 (27.23)		
***Child age***							
0–12 month	1442 (22.75)	55 (3.81)	671 (46.53)	426 (29.54)	290 (20.11)	170.29	*<0*.*001*
13–36 months	2808 (44.30)	101 (3.60)	1205 (42.91)	803 (28.60)	699 (24.89)		
37–59 months	2088 (32.94)	51 (2.44)	716 (34.29)	522 (25.00)	799 (38.27)		
***Maternal age***							
15–29 years	3496 (55.16)	119 (3.40)	1483 (42.42)	941 (26.92)	953 (27.26)	8.80	*0*.*032*
30–49 years	2842 (44.84)	88 (3.10)	1109 (39.02)	810 (28.50)	835 (29.38)		
***Maternal anemia***							
Anemic	3696 (58.31)	148 (4.00)	1667 (45.10)	1039 (28.11)	842 (22.78)	146.57	*<0*.*001*
Not anemic	2642 (41.69)	59 (2.23)	925 (35.01)	712 (26.95)	946 (35.81)		

**Source: Data extracted from NDHS 2018

**Table 2:** The percentage of children with anemia among Nigerian children (0–59 months) classified by dietary variables *(N = 6*,*338)*.

Variables	n (%)	Anemia status (%)				χ^2^ value	*p*-value
		Anemia			Not anemia		
		Severe	Moderate	Mild			
***Fortified baby food***							
No	6070 (95.77)	203 (3.34)	2491 (41.04)	1685 (27.76)	1691 (27.86)	10.63	0.014
Yes	268 (4.23)	4 (1.49)	101 (37.69)	66 (24.63)	97 (36.19)		
***Tinned*, *powdered*, *or fresh milk***							
No	5433 (85.72)	188 (3.46)	2256 (41.52)	1500 (27.61)	1489 (27.41)	16.79	0.001
Yes	905 (14.28)	19 (2.10)	336 (37.13)	251 (27.73)	299 (33.04)		
***Juice***							
No	5664 (89.37)	192 (3.39)	2383 (42.07)	1548 (27.33)	1541 (27.21)	41.25	<0.001
Yes	674 (10.63)	15 (2.23)	209 (31.01)	203 (30.12)	247 (36.65)		
***Infant formula***							
No	5880 (92.77)	201(3.42)	2441 (41.51)	1612 (27.41)	1626 (27.65)	23.66	<0.001
Yes	458 (7.23)	6 (1.31)	151 (32.97)	139 (30.35)	162 (35.37)		
***Soup***							
No	5894 (92.99)	191 (3.24)	2413 (40.93)	1613 (27.36)	1678 (28.46)	4.24	0.237
Yes	443 (7.01)	16 (3.61)	179 (40.41)	138 (31.15)	110 (24.83)		
***Bread*, *rice*, *noodles*, *porridge*, *or other foods made from grains***							
No	1897 (29.93)	47 (2.48)	751 (39.59)	543 (28.62)	556 (29.31)	8.42	0.038
Yes	4441 (70.07)	160 (3.60)	1841 (41.45)	1208 (27.20)	1232 (27.74)		
***Potatoes*, *cassava*, *or other tubers***							
No	4608 (72.70)	169 (3.67)	1871 (40.60)	1247 (27.06)	1321 (28.67)	11.89	0.008
Yes	1730 (27.30)	38 (2.20)	721 (41.68)	504 (29.13)	467 (26.99)		
***Eggs***							
No	5374 (84.79)	182 (3.39)	2257 (42.00)	1469 (27.34)	1466 (27.28)	24.02	< 0.001
Yes	964 (15.21)	25 (2.59)	335 (34.75)	282 (29.25)	322 (33.40)		
***Any meat (beef*, *pork*, *lamb*, *goat*, *chicken*, *or duck)***							
No	5347 (84.79)	180 (3.37)	2231 (41.72)	1453 (27.17)	1483 (27.74)	12.07	0.007
Yes	991 (15.21)	27 (2.72)	361 (36.43)	298 (30.07)	305 (30.78)		
***Pumpkin*, *carrots*, *squash*, *or sweet potatoes (yellow or orange inside)***							
No	5808 (91.64)	178 (3.06)	2369 (40.79)	1624 (27.96)	1637 (28.19)	11.64	0.009
Yes	530 (8.36)	29 (5.47)	223 (42.08)	127 (23.96)	151 (28.49)		
***Any dark green leafy vegetables***							
No	4166 (65.73)	137 (3.29)	1705 (40.93)	1137 (27.29)	1187 (28.49)	0.86	0.836
Yes	2172 (34.27)	70 (3.22)	887 (40.84)	614 (28.27)	601 (27.67)		
***Ripe mangoes*, *papayas*, *other vitamin A fruits***							
No	6079 (95.91)	202 (3.32)	2478 (40.91)	1670 (27.47)	1720 (28.29)	3.15	0.369
Yes	259 (4.09)	5 (1.93)	105 (40.54)	81 (31.27)	68 (26.25)		
***Any other fruits***							
No	5427 (85.63)	173 (3.19)	2262 (41.68)	1484 (27.34)	1508 (27.79)	9.88	0.020
Yes	911 (14.37)	34 (3.73)	330 (36.22)	267 (29.31)	280 (30.74)		
***Liver*, *heart*, *other organ meats***							
No	6116 (96.50)	198 (3.24)	2529 (41.35)	1674 (27.37)	1715 (28.04)	15.18	0.002
Yes	22 (3.50)	9 (4.05)	63 (28.38)	77 (34.68)	73 (32.88)		
***Fresh or dried fish or shellfish***							
No	4813 (75.94)	173 (3.59)	1975 (41.03)	1313 (27.28)	1352 (28.09)	7.67	0.053
Yes	1525 (24.06)	34 (2.23)	617 (40.46)	438 (28.72)	436 (28.59)		
***Food made from beans*, *peas*, *lentils*, *nuts***							
No	4453 (70.26)	141 (3.17)	1829 (41.07)	1209 (27.15)	1274 (28.61)	2.65	0.449
Yes	1885 (29.74)	66 (3.50)	763 (40.48)	542 (28.75)	514 (27.27)		
***Cheese or other food made with milk***							
No	5662 (89.33)	184 (3.25)	2322 (41.01)	1568 (27.69)	1588 (28.05)	0.80	0.849
Yes	676 (10.67)	23 (3.40)	270 (39.94)	183 (27.07)	200 (29.59)		
***Plane water***							
No	644 (10.16)	28 (4.35)	234 (36.34)	184 (28.57)	198 (30.75)	8.08	0.044
Yes	5694 (89.84)	179 (3.14)	2358 (41.41)	1567 (27.52)	1590 (27.92)		

**Source: Data extracted from NDHS 2018

The results of the Chi-squared test showed that the factors such as the education status of the mother, the sex of the child, religion, the wealth index, residence, breastfeeding status, the age of the child, maternal age, and maternal anemia were significantly associated with the occurrence of anemia (p < 0.05).

Regarding the association between the occurrence of anemia and the maternal education status, we found that 57% of the children whose mothers had no education were severely anemic, while only 0.48% of the children whose mothers had higher education status were severely anemic. Similarly, moderate anemia was considerably higher among the children of uneducated mothers (no education = 42.52%, primary = 17.63%, secondary = 34.76%, and higher = 5.09%). Severe anemia was considerably higher among male children (male = 55.56% and female = 44.44%).

### Sociodemographic factors associated with childhood anemia in Nigeria

The sociodemographic factors associated with anemia among Nigerian children are presented in **Table 3**. Maternal education status, wealth index, the age of the child, and maternal anemia status were significantly associated with anemia in children. The chances of anemia were 27% lower among children whose mothers received higher education than in those children whose mothers did not receive any education (OR = 0.73; 95% CI = 0.58–0.91; *p =* 0.004). The children belonging to families with a high socioeconomic status had a 16% lower chance of anemia than those belonging to families with a poor socioeconomic status (OR = 0.84; 95% CI = 0.74–0.94; *p =* 0.004). The age of the children was significantly associated with anemia in children, and the chances of anemia were 13% and 44% lower among the children aged 13–36 months (OR = 0.87; 95% CI = 0.77–0.98; *p =* 0.019) and 37–59 months (OR = 0.56; 95% CI = 0.49–0.63; *p* < 0.001), respectively. Maternal anemia status was significantly associated with anemia in children. Children of non-anemic mothers had a 28% lower chance of suffering from anemia (OR = 0.72; 95% CI = 0.66–0.80; *p* < 0.001). The residence of the children was a significant factor in the bivariate analysis, but its association with anemia was not significant in the multivariate analysis.

**Table 3:** The results of the bivariate and multivariate analyses of the effects of some socioeconomic and demographic factors on childhood anemia by ordered logistic regression reporting Odds ratio.

	*Bivariate analysis*		*Multivariate analysis*	
*Variable*	Odds ratio (OR)	P-value	Odds ratio (OR)	P-value
***Educational status of mother***				
Ref. (No education)	-	-	-	-
Primary	1.04 (0.91–1.18)	0.564	1.07 (0.92–1.24)	0.376
Secondary	0.91(0.82–1.01)	0.071	0.95 (0.82–1.10)	0.515
Higher	0.64(0.53–0.76)	< 0.001	0.73 (0.58–0.91)	0.004
***Sex of child***				
Ref. (male)	-	-	-	-
Female	0.94 (0.86–1.03)	0.164	0.95 (0.87–1.04)	0.269
***Religion***				
Ref. (Christian)	-	-	-	-
Islam	1.02 (0.93–1.11)	0.721	0.94 (0.84–1.05)	0.290
Others	1.45 (0.89–2.35)	0.135	1.33 (0.81–2.18)	0.253
**Wealth index**				
Ref. (poor)	-	-	-	-
Middle	0.90 (0.80–1.02)	0.106	0.91 (0.80–1.03)	0.132
Rich	0.81 (0.74–0.90)	< 0.001	0.84 (0.74–0.94)	0.004
***Residence***				
Ref. (urban)	-	-	-	-
Rural	1.15 (1.04–1.26)	0.005	1.11(0.99–1.24)	0.066
***Currently breast feeding***				
Ref. (no)	-	-	-	-
Yes	1.06 (0.95–1.18)	0.309	1.00 (0.89–1.13)	0.942
***Child age***				
Ref. (6–12 months)	-	-	-	-
(13–36) months	0.88(0.78–0.98)	0.023	0.87 (0.77–0.98)	0.019
(37–59) months	0.57(0.50–0.65	< 0.001	0.56 (0.49–0.63)	<0.001
***Maternal age***				
Ref. (15–29 years)	-	-	-	-
(30–49) years versus	0.99 (0.90–1.08)	0.780	1.03 (0.94–1.13)	0.519
***Maternal anemia status***				
Ref. (anemic)	-	-	-	-
Non anemic	0.71 (0.65–0.78)	< 0.001	0.72 (0.66–0.80)	<0.001

*The probability of being severely, moderately, and mildly anemic vs. non-anemic in the relevant category of the socioeconomic and demographic status from the reference category of the independent variables.

### Dietary factors associated with childhood anemia in Nigeria

We found that some food groups, such as juice, white potatoes, white yams, manioc, cassava, and other foods including roots, pumpkin, carrots, squash, or sweet potatoes (yellow or orange inside), are associated with childhood anemia in Nigeria **(Table 4).** Children who consumed juice had 15% lower chances of anemia (OR = 0.85; 95% CI = 0.72–1.00; *p =* 0.046). The chance of anemia was also 14% higher among the children who consumed white potatoes, white yams, manioc, cassava, or other food items made from roots (OR = 1.14; 95% CI = 1.01–1.29; *p =* 0.036). Children who consumed pumpkin, carrots, squash, or sweet potatoes (yellow or orange inside) were also 17% less likely to have anemia (OR = 0.83; 95% CI = 0.70–0.99; *p =* 0.036).

**Table 4:** The results of the bivariate and multivariate analyses of the effects of diet on childhood anemia by ordered logistic regression reporting Odds ratio.

*Variable*	*Bivariate analysis*		*Multivariate analysis*	
	Odd ratio (OR)	P value	Odds ratio (OR)	P value
***Fortified baby food***				
*Yes (ref*. *no)*	0.77 (0.61–0.96)	0.022	0.82 (0.64–1.04)	0.102
***Tinned*, *powdered or fresh milk***				
*Yes (ref*. *no)*	0.88 (0.77–1.01)	0.063	0.92 (0.80–1.05)	0.246
***Gave child juice***				
*Yes (ref*. *no)*	0.84 (0.72–0.98)	0.027	0.85(0.72–1.00)	0.046
***Infant formula***				
*Yes (ref*. *no)*	0.90 (0.75–1.08)	0.256	1.01(0.83–1.22)	0.954
***Soup***				
*Yes (ref*. *no)*	1.19(0.99–1.42)	0.053	1.2 (0.99–1.44)	0.059
***Bread*, *rice*, *noodles*, *porridge*, *or other foods made from grains***				
*Yes (ref*. *no)*	0.99 (0.89–1.09)	0.847	0.95 (0.85–1.06)	0.393
***White potatoes*, *white yams*, *manioc*, *cassava or other food made from roots***				
*Yes (ref*. *no)*	1.12 (1.01–1.24)	0.030	1.14(1.01–1.29)	0.036
***Eggs***				
*Yes (ref*. *no)*	0.90 (0.79–1.02)	0.111	0.90 (0.78–1.04)	0.154
***Any meat (beef*, *pork*, *lamb*, *goat*, *chicken or duck)***				
*Yes (ref*. *no)*	0.99 (0.88–1.13)	0.986	1.01 (0.87–1.16)	0.926
***Pumpkin*, *carrots*, *squash or sweet potatoes (yellow or orange inside)***				
*Yes (ref*. *no)*	0.87 (0.74–1.03)	0.106	0.83 (0.70–0.99)	0.036
***Any dark green leafy vegetables***				
*Yes (ref*. *no)*	1.05 (0.95–1.15)	0.359	1.01(0.90–1.13)	0.832
***Ripe mangoes*, *papayas*, *other vitamin A fruits***				
*Yes (ref*. *no)*	1.17 (0.94–1.48)	0.165	1.21 (0.94–1.55)	0.138
***Any other fruits or vegetables***				
*Yes (ref*. *no)*	0.96 (0.84–1.09)	0.559	0.93 (0.81–1.08)	0.351
***Liver*, *heart*, *other organ meats***				
*Yes (ref*. *no)*	1.04 (0.80–1.34)	0.781	1.11 (0.84–1.47)	0.453
***Fresh or dried fish or shellfish***				
*Yes (ref*. *no)*	1.04 (0.94–1.16)	0.447	1.00 (0.89–1.13)	0.983
***Food made from beans*, *peas*, *lentils*, *nuts***				
*Yes (ref*. *no)*	1.07 (0.97–1.18)	0.188	1.09 (0.98–1.22)	0.097
***Cheese or other food made with milk***				
*Yes (ref*. *no)*	0.95 (0.82–1.09)	0.096	0.98 (0.84–1.15)	0.817
***Plane water***				
*Yes (ref*. *no)*	1.07 (0.92–1.24)	0.384	1.07 (0.91–1.25)	0.419

*The probability of being severely, moderately, and mildly anemic vs. non-anemic in the relevant category of the dietary status from the reference category of the independent variables.

## Discussion

The results of this study showed that among 6,338 children, 71% of the children (n = 4,550) were anemic in Nigeria. Maternal education was significantly associated with the occurrence of anemia among children. Children whose mothers received higher education had lower chances of anemia. Studies in India and Korea reported similar results that maternal education level played a key role in childhood anemia [23, 24]. This might be because educated mothers are more concerned about their children’s health, nutrition, and diets [18, 19, 25]. Furthermore, educated mothers can make better decisions, which increases their ability to purchase different types of foods for their children [26, 27].

The age of the child is another factor associated with the occurrence of anemia. In this study, anemia was more prevalent among children aged 6–12 months compared to those aged 37–59 months. These results were similar to those of studies that reported a higher occurrence of anemia among children in their early stages of life (6–23 months) [17, 28–30]. A study found that anemia was likely to occur in breastfed babies older than six months who were either not getting iron supplementation or were provided non-iron-fortified formula before the age of 12 months, and they were categorized into the high-risk group [31]. Iron supplementation is not required in the first four to six months of life since children can use iron stored in the liver or obtained from dietary formula or breast milk [31]. They need an additional source of iron after six months of age [31]. Iron reserves might get depleted if weaning meals have insufficient bioavailable iron [7]. Their iron demand is higher than the amount provided by breast milk exclusively at that stage, resulting in iron deficiency anemia [25].

In this study, the wealth index was also found to be a significant predictor of anemia in children. Children from wealthy families had a lower risk of anemia than those from poor families due to the better nutritional status of wealthy families [3, 17]. Similar findings were observed in different countries, including Ethiopia, Bangladesh, and Ghana [17, 28, 32]. This is because poor families might not be able to afford a sufficient and diverse nutrient-rich diet (such as iron, vitamins, and other nutrients), as well as health care, resulting in poor child health [28, 33].

In this study, we found that children with anemic mothers had a substantially higher risk of anemia than children with non-anemic mothers. Children and their mothers had similar socioeconomic conditions, food patterns, nutritional status, genetic features, and health facilities, which might be the underlying cause of the link between maternal and childhood anemia [25, 29, 30]. Our results were consistent with those of other studies conducted in other countries [3, 25, 29, 32].

Rural children are 15% more anemic than urban children in Nigeria. Anemia in children is also 1.21 times more common in rural children than in urban children in Bangladesh [8], which is consistent with our findings. Childhood anemia is more common among rural children due to malnutrition induced by the lack of a balanced diet [8], poor hygiene and sanitation, lower maternal education level, and a lack of dietary knowledge [34].

Besides socioeconomic and demographic variables, poor dietary habits are another major cause of childhood anemia. Lower consumption of foods (such as meat) containing iron and vegetables containing vitamins A and C that help in iron metabolism might lead to iron deficiency anemia [24]. The presence of iron-absorption inhibitors, including phytates in bran, calcium in dairy products, polyphenols in certain vegetables, tannins in tea, and oxalate, phosphate, and fiber in a cereal-based diet might also lead to anemia [35]. In this study, the occurrence of anemia was significantly lower in the children who consumed juice **(Table 4)**. Citrus fruits and juices containing vitamins A and C promote higher absorption of dietary iron in children [36]. Ascorbic acid enhances iron absorption by facilitating effective transport via the microvilli of the duodenum through reduction processes involving the conversion of ferric ion to ferrous ion [37]. Consuming iron-fortified cereals and citrus fruits containing vitamin C can boost iron absorption in infants [36].

To prevent iron insufficiency in toddlers, an iron-rich diet consisting of heme iron foods, such as red meat, poultry, and fish, is ideal. Meat consumption might account for about 40% of the total absorbed iron due to its high absorbability [38].

Although breastfeeding was unrelated to the occurrence of anemia in children in our study **(Table 3)**, breast milk is considered to be the best food for infants since it includes 50% bioavailable iron [39]. Providing cow milk instead of breast milk to infants under the age of one year is thought to be the most important dietary risk factor for iron deficiency and anemia. Cow milk has low bioavailable iron content and high calcium content, which reduces iron absorption from other foods [25, 39].

Anemia is common in children from Togo, although the children are breastfed. This is because their staple meals are low in diversity, consisting primarily of starchy foods such as corn, millet, cassava, and rice, all of which are high in carbohydrates [40]. We found that children who were fed white potatoes, white yams, manioc, cassava, or other root-based foods had a 14% increased risk of anemia **(Table 4)**. This might be because tubers and roots contain a significant amount of phytates [41]. Antinutritional components discovered in cassava include hydrogen cyanide (20.13 mg/100 g), oxalate (3.27 mg/100 g), and phytate (62.4 mg/100 g) [42]. Garri, a cassava-based food, is a staple food of Nigeria [42]. However, this monotonous plant-based diet provides insufficient quantities of bioavailable iron. Phytates decrease iron absorption in a dose-dependent manner, and even small amounts of phytates can have a noticeable effect. This is more common in newborns and children whose physiological iron needs are the highest [43]. Gegios et al. found that healthy children aged 2–5 years in Nigeria, who primarily eat cassava, are at a high risk of vitamin A, zinc, and iron deficiency [44]. Anemia was greatly reduced in children who ate yellow or orange vegetables such as pumpkin, carrots, squash, or sweet potatoes. Several studies found improvements in the level of iron in the body after the consumption of vitamin A-fortified foods and β-carotene-rich foods such as amaranth, gac (*Momordica cochinchinensis*) fruit, spinach, cabbage, radish leaves, papaya, and carrot, similar to our findings [45–49]. The consumption of carotene-rich yellow and green leafy vegetables can increase the total size of the pool of vitamin A and the Hb concentration and lower anemia rates [50]. Pro-vitamin A carotenoids found in yellow and orange fruits and vegetables are the main dietary source of vitamin A for young children in developing countries [51]. Several studies have found that vitamin A deficiency is an important cause of nutritional anemia, and the daily administration of vitamin A supplements significantly increased the hemoglobin content in anemic children [52–54].

### Strength and limitations of the study

The data quality in this research is quite good since it is based on a nationally representative survey that is monitored and managed by an international expert group. The outcomes from nationally representative data are more effective for policymakers in creating suitable solutions. Furthermore, in this study, we try to find out the association between anemia and most of the potential variables available in the data set.

However, this study has some limitations. We used secondary data of NDHS, and the dataset encompasses 24 hours recall dietary data. However, in the case of dietary data collection based on one-time 24 hours recall system may contain somewhat less reliable information [55]. Nevertheless, DHS, through their expert opinion, recognized that 24-hour recall systems could provide valuable dietary information [12]. The amount and frequency of food intake were not collected during this survey. Helminth infection, one of the major causes of anemia, was not examined in this study.

## Conclusion

We found a high rate of childhood anemia in Nigeria. The staple food of Nigeria is Cassava-based, and our results showed that the consumption of white potatoes, white yams, manioc, cassava, and other root-based foods increases the risk of childhood anemia. Incorporating a diverse iron-rich diet and fortified foods with vitamins, reducing maternal anemia, and improving socioeconomic conditions might considerably decrease the occurrence of childhood anemia in Nigeria. Furthermore, the iron supplementation intervention program already adopted by the Nigerian government should be continued until the prevalence of anemia comes below the 40% level. The findings of this study might help to improve the programs that incorporate and consider recognized risk factors, especially for children who are 6–12 months old.

## Supporting information

S1 Questionnaire This is the English version of the questionnaire. (PDF)
