# Supplementary material for: Prevalence of childhood anemia: Potential sociodemographic and dietary factors in Nigeria
Source: PLoS One. 2022 Dec 9;17(12):e0278952. doi: 10.1371/journal.pone.0278952 (PMC9733855; doi:10.1371/journal.pone.0278952)
Supplement: S1 Questionnaire — (PDF) [file pone.0278952.s001.pdf]

QUESTIONNAIRES

Appendix E

FORMATTING DATE: 16 Mar 2017
ENGLISH LANGUAGE: 26 May 2018

NIGERIA DEMOGRAPHIC AND HEALTH SURVEYS 2018
HOUSEHOLD QUESTIONNAIRE

NIGERIA
NATIONAL POPULATION COMMISSION

IDENTIFICATION

STATE ______________________ [ ][ ][ ]

LOCAL GOVT. AREA ______________________ [ ][ ][ ]

LOCALITY ______________________ [ ][ ][ ][ ][ ]

ENUMERATION AREA ______________________ [ ][ ][ ][ ]

NAME OF HOUSEHOLD HEAD ______________________

CLUSTER NUMBER . . . . [ ][ ][ ][ ]

HOUSEHOLD NUMBER . . . . [ ][ ][ ][ ]

HOUSEHOLD SELECTED FOR MAN'S SURVEY? (1=YES, 2=NO) . . . . [ ]

INTERVIEWER VISITS

	1	2	3	FINAL VISIT
DATE	________	________	________	DAY [ ][ ]<br>MONTH [ ][ ]<br>YEAR [ ][ ][ ][ ]
INTERVIEWER'S NAME	________	________	________	INT. NO. [ ][ ][ ][ ]
RESULT*	________	________	________	RESULT* [ ]
NEXT VISIT: DATE	________	________		TOTAL NUMBER OF VISITS [ ]
TIME	________	________		

*RESULT CODES:

1 COMPLETED
2 NO HOUSEHOLD MEMBER AT HOME OR NO COMPETENT RESPONDENT AT HOME AT TIME OF VISIT
3 ENTIRE HOUSEHOLD ABSENT FOR EXTENDED PERIOD OF TIME
4 POSTPONED
5 REFUSED
6 DWELLING VACANT OR ADDRESS NOT A DWELLING
7 DWELLING DESTROYED
8 DWELLING NOT FOUND
9 OTHER ______________________ (SPECIFY)

TOTAL PERSONS IN HOUSEHOLD [ ][ ]

TOTAL ELIGIBLE WOMEN [ ][ ]

TOTAL ELIGIBLE MEN [ ][ ]

LINE NO. OF RESPONDENT TO HOUSEHOLD QUESTIONNAIRE [ ][ ]

LANGUAGE OF QUESTIONNAIRE** [0][1] LANGUAGE OF INTERVIEW** [ ][ ] NATIVE LANGUAGE OF RESPONDENT** [ ][ ] TRANSLATOR USED (YES = 1, NO = 2) [ ]

LANGUAGE OF QUESTIONNAIRE** **ENGLISH**

**LANGUAGE CODES:
01 ENGLISH
02 HAUSA
03 YORUBA
04 IGBO

SUPERVISOR	FIELD EDITOR
NAME ________ NUMBER [ ][ ][ ][ ]	NAME ________ NUMBER [ ][ ][ ][ ]

THIS PAGE IS INTENTIONALLY BLANK

INTRODUCTION AND CONSENT

Hello. My name is ____. I am working with National Population Commission. We are conducting a survey about health and other topics all over Nigeria. The information we collect will help the government to plan health services. Your household was selected for the survey. I would like to ask you some questions about your household. The questions usually take about 20 to 30 minutes. All of the answers you give will be confidential and will not be shared with anyone other than members of our survey team. You don't have to be in the survey, but we hope you will agree to answer the questions since your views are important. If I ask you any question you don't want to answer, just let me know and I will go on to the next question or you can stop the interview at any time. In case you need more information about the survey, you may contact the person listed on this card.

GIVE CARD WITH CONTACT INFORMATION

Do you have any questions?
May I begin the interview now?

SIGNATURE OF INTERVIEWER ____ DATE ______________________

RESPONDENT AGREES TO BE INTERVIEWED . . 1 ↓

RESPONDENT DOES NOT AGREE TO BE INTERVIEWED . . 2 → END

100	RECORD THE TIME.	HOURS . . . . [ ][ ]<br>MINUTES . . . . [ ][ ]

HOUSEHOLD SCHEDULE

							IF AGE 15 OR OLDER			
LINE NO.	USUAL RESIDENTS AND VISITORS	RELATIONSHIP TO HEAD OF HOUSEHOLD	SEX	RESIDENCE		AGE	MARITAL STATUS	ELIGIBILITY		
1	2	3	4	5	6	7	8	9	10	11
	Please give me the names of the persons who usually live in your household and guests of the household who stayed here last night, starting with the head of the household.<br><br>AFTER LISTING THE NAMES AND RECORDING THE RELATIONSHIP AND SEX FOR EACH PERSON, ASK QUESTIONS 2A-2C TO BE SURE THAT THE LISTING IS COMPLETE.<br><br>THEN ASK APPROPRIATE QUESTIONS IN COLUMNS 5-20 FOR EACH PERSON.	What is the relationship of (NAME) to the head of the household?<br><br>SEE CODES BELOW.	Is (NAME) male or female?	Does (NAME) usually live here?	Did (NAME) stay here last night?	How old is (NAME)?<br><br>IF 95 OR MORE, RECORD '95'.	What is (NAME)'s current marital status?<br><br>1 = MARRIED OR LIVING TOGETHER<br>2 = DIVORCED/ SEPARATED<br>3 = WIDOWED<br>4 = NEVER-MARRIED AND NEVER LIVED TOGETHER	CIRCLE LINE NUMBER OF ALL WOMEN AGE 15-49	**IF HOUSE-HOLD SELEC-TED FOR MAN'S SURVEY**<br><br>CIRCLE LINE NUMBER OF ALL MEN AGE 15-59	CIRCLE LINE NUMBER OF ALL CHILDREN AGE 0-5
			M F	Y N	Y N	IN YEARS				
01			1 2	1 2	1 2			01	01	01
02			1 2	1 2	1 2			02	02	02
03			1 2	1 2	1 2			03	03	03
04			1 2	1 2	1 2			04	04	04
05			1 2	1 2	1 2			05	05	05
06			1 2	1 2	1 2			06	06	06
07			1 2	1 2	1 2			07	07	07
08			1 2	1 2	1 2			08	08	08
09			1 2	1 2	1 2			09	09	09
10			1 2	1 2	1 2			10	10	10

2A) Just to make sure that I have a complete listing: are there any other people such as small children or infants that we have not listed?	YES → ADD TO TABLE	NO
2B) Are there any other people who may not be members of your family, such as domestic servants, lodgers, or friends who usually live here?	YES → ADD TO TABLE	NO
2C) Are there any guests or temporary visitors staying here, or anyone else who stayed here last night, who have not been listed?	YES → ADD TO TABLE	NO

CODES FOR Q. 3: RELATIONSHIP TO HEAD OF HOUSEHOLD

01 = HEAD
02 = WIFE OR HUSBAND
03 = SON OR DAUGHTER
04 = SON-IN-LAW OR DAUGHTER-IN-LAW
05 = GRANDCHILD
06 = PARENT
07 = PARENT-IN-LAW
08 = BROTHER OR SISTER
09 = BROTHER-IN-LAW/SISTER IN-LAW
10 = NIECE/NEPHEW BY BLOOD
11 = NIECE/NEPHEW BY MARRIAGE
12 = OTHER RELATIVE
13 = ADOPTED/FOSTER/ STEPCHILD
14 = NOT RELATED
15 = CO-WIFE
98 = DON'T KNOW

HOUSEHOLD SCHEDULE

							IF AGE 15 OR OLDER			
LINE NO.	USUAL RESIDENTS AND VISITORS	RELATIONSHIP TO HEAD OF HOUSEHOLD	SEX	RESIDENCE		AGE	MARITAL STATUS	ELIGIBILITY		
1	2	3	4	5	6	7	8	9	10	11
	Please give me the names of the persons who usually live in your household and guests of the household who stayed here last night, starting with the head of the household.<br><br>AFTER LISTING THE NAMES AND RECORDING THE RELATIONSHIP AND SEX FOR EACH PERSON, ASK QUESTIONS 2A-2C TO BE SURE THAT THE LISTING IS COMPLETE.<br><br>THEN ASK APPROPRIATE QUESTIONS IN COLUMNS 5-20 FOR EACH PERSON.	What is the relationship of (NAME) to the head of the household?<br><br>SEE CODES BELOW.	Is (NAME) male or female?	Does (NAME) usually live here?	Did (NAME) stay here last night?	How old is (NAME)?<br><br>IF 95 OR MORE, RECORD '95'.	What is (NAME)'s current marital status?<br><br>1 = MARRIED OR LIVING TOGETHER<br>2 = DIVORCED/ SEPARATED<br>3 = WIDOWED<br>4 = NEVER-MARRIED AND NEVER LIVED TOGETHER	CIRCLE LINE NUMBER OF ALL WOMEN AGE 15-49	IF HOUSE-HOLD SELEC-TED FOR MAN'S SURVEY<br><br>CIRCLE LINE NUMBER OF ALL MEN AGE 15-59	CIRCLE LINE NUMBER OF ALL CHILDREN AGE 0-5
			M F	Y N	Y N	IN YEARS				
11		☐☐	1 2	1 2	1 2	☐☐	☐	11	11	11
12		☐☐	1 2	1 2	1 2	☐☐	☐	12	12	12
13		☐☐	1 2	1 2	1 2	☐☐	☐	13	13	13
14		☐☐	1 2	1 2	1 2	☐☐	☐	14	14	14
15		☐☐	1 2	1 2	1 2	☐☐	☐	15	15	15
16		☐☐	1 2	1 2	1 2	☐☐	☐	16	16	16
17		☐☐	1 2	1 2	1 2	☐☐	☐	17	17	17
18		☐☐	1 2	1 2	1 2	☐☐	☐	18	18	18
19		☐☐	1 2	1 2	1 2	☐☐	☐	19	19	19
20		☐☐	1 2	1 2	1 2	☐☐	☐	20	20	20

TICK HERE IF CONTINUATION SHEET USED ☐

CODES FOR Q. 3: RELATIONSHIP TO HEAD OF HOUSEHOLD

01 = HEAD
02 = WIFE OR HUSBAND
03 = SON OR DAUGHTER
04 = SON-IN-LAW OR DAUGHTER-IN-LAW
05 = GRANDCHILD
06 = PARENT
07 = PARENT-IN-LAW
08 = BROTHER OR SISTER
09 = BROTHER-IN-LAW/SISTER IN-LAW
10 = NIECE/NEPHEW BY BLOOD
11 = NIECE/NEPHEW BY MARRIAGE
12 = OTHER RELATIVE
13 = ADOPTED/FOSTER/ STEPCHILD
14 = NOT RELATED
15 = CO-WIFE
98 = DON'T KNOW

LINE NO.	IF AGE 0-17 YEARS				IF AGE 5 YEARS OR OLDER		IF AGE 5-24 YEARS		IF AGE 0-4 YEARS		
	SURVIVORSHIP AND RESIDENCE OF BIOLOGICAL PARENTS				EVER ATTENDED SCHOOL		CURRENT/RECENT SCHOOL ATTENDANCE		BIRTH REGISTRATION		
	12	13	14	15	16	17	18	19	20A	20B	20C
	Is (NAME)'s natural mother alive?	Does (NAME)'s natural mother usually live in this household or was she a guest last night?<br><br>IF YES: What is her name?<br><br>RECORD MOTHER'S LINE NUMBER.<br><br>IF NO, RECORD '00'.	Is (NAME)'s natural father alive?	Does (NAME)'s natural father usually live in this household or was he a guest last night?<br><br>IF YES: What is his name?<br><br>RECORD FATHER'S LINE NUMBER.<br><br>IF NO, RECORD '00'.	Has (NAME) ever attended school?	What is the highest level of school (NAME) has attended?<br><br>What is the highest class/year (NAME) completed at that level?<br><br>SEE CODES BELOW.	Did (NAME) attend school at any time during the 2017-2018 (2018-2019) school year?	During [this/that] school year, what level and class/year [is/was] (NAME) attending?<br><br>SEE CODES BELOW.	Was (NAME'S) birth registered?	With which authority was (NAME'S) birth registered?<br><br>1 = NPOPC<br>2 = LGA<br>3 = PRIVATE CLINIC/ HOSPITAL<br>4 = OTHER	May I see (NAME'S) birth certificate?<br><br>1 = SEEN<br>2 = NOT SEEN
01	Y N DK<br>1 2 8<br>GO TO 14		Y N DK<br>1 2 8<br>GO TO 16		Y N<br>1 2<br>GO TO 21	LEVEL CLASS/YEAR	Y N<br>1 2<br>GO TO 21	LEVEL CLASS/YEAR	Y N DK<br>1 2 8<br>NEXT LINE		
02	1 2 8<br>GO TO 14		1 2 8<br>GO TO 16		1 2<br>GO TO 21		1 2<br>GO TO 21		1 2 8<br>NEXT LINE		
03	1 2 8<br>GO TO 14		1 2 8<br>GO TO 16		1 2<br>GO TO 21		1 2<br>GO TO 21		1 2 8<br>NEXT LINE		
04	1 2 8<br>GO TO 14		1 2 8<br>GO TO 16		1 2<br>GO TO 21		1 2<br>GO TO 21		1 2 8<br>NEXT LINE		
05	1 2 8<br>GO TO 14		1 2 8<br>GO TO 16		1 2<br>GO TO 21		1 2<br>GO TO 21		1 2 8<br>NEXT LINE		
06	1 2 8<br>GO TO 14		1 2 8<br>GO TO 16		1 2<br>GO TO 21		1 2<br>GO TO 21		1 2 8<br>NEXT LINE		
07	1 2 8<br>GO TO 14		1 2 8<br>GO TO 16		1 2<br>GO TO 21		1 2<br>GO TO 21		1 2 8<br>NEXT LINE		
08	1 2 8<br>GO TO 14		1 2 8<br>GO TO 16		1 2<br>GO TO 21		1 2<br>GO TO 21		1 2 8<br>NEXT LINE		
09	1 2 8<br>GO TO 14		1 2 8<br>GO TO 16		1 2<br>GO TO 21		1 2<br>GO TO 21		1 2 8<br>NEXT LINE		
10	1 2 8<br>GO TO 14		1 2 8<br>GO TO 16		1 2<br>GO TO 21		1 2<br>GO TO 21		1 2 8<br>NEXT LINE		

CODES FOR Qs. 17 AND 19: EDUCATION

LEVEL
0 = PRESCHOOL
1 = PRIMARY
2 = SECONDARY
3 = HIGHER
8 = DON'T KNOW

EDUCATION YEAR
01-03 = YEARS AT PRE-PRIMARY/KINDERGARTEN
01-06 = YEARS 1-6 AT PRIMARY LEVEL
01-06 = YEARS 1-6 AT SECONDARY LEVEL
01 - TOTAL NUMBER OF YEARS AT HIGHER LEVEL*
00 = LESS THAN 1 YEAR COMPLETED
(USE '00' FOR Q. 17 ONLY. THIS CODE IS NOT ALLOWED FOR Q. 19.)
98 = DON'T KNOW

***FOR `HIGHER' TOTAL THE NUMBER OF YEARS AT THE POST-SECONDARY LEVEL**

HOUSEHOLD SCHEDULE

	IF AGE 0-17 YEARS				IF AGE 5 YEARS OR OLDER		IF AGE 5-24 YEARS		IF AGE 0-4 YEARS		
LINE NO.	SURVIVORSHIP AND RESIDENCE OF BIOLOGICAL PARENTS				EVER ATTENDED SCHOOL		CURRENT/RECENT SCHOOL ATTENDANCE		BIRTH REGISTRATION		
	12	13	14	15	16	17	18	19	20A	20B	20C
	Is (NAME)'s natural mother alive?	Does (NAME)'s natural mother usually live in this household or was she a guest last night?<br><br>IF YES: What is her name?<br><br>RECORD MOTHER'S LINE NUMBER.<br><br>IF NO, RECORD '00'.	Is (NAME)'s natural father alive?	Does (NAME)'s natural father usually live in this household or was he a guest last night?<br><br>IF YES: What is his name?<br><br>RECORD FATHER'S LINE NUMBER.<br><br>IF NO, RECORD '00'.	Has (NAME) ever attended school?	What is the highest level of school (NAME) has attended?<br><br>What is the highest class/year (NAME) completed at that level?<br><br>SEE CODES BELOW.	Did (NAME) attend school at any time during the 2017-2018 (2018-2019) school year?	During [this/that] school year, what level and class/year [is/was] (NAME) attending?<br><br>SEE CODES BELOW.	Was (NAME'S) birth registered?	With which authority was (NAME'S) birth registered?<br><br>1 = NPOPC<br>2 = LGA<br>3 = PRIVATE CLINIC/ HOSPITAL<br>4 = OTHER	May I see (NAME'S) birth certificate?<br><br>1 = SEEN<br>2 = NOT SEEN
	Y N DK		Y N DK		Y N	LEVEL CLASS/YEAR	Y N	LEVEL CLASS/YEAR	Y N DK		
11	1 2 8<br>GO TO 14		1 2 8<br>GO TO 16		1 2<br>GO TO 21		1 2<br>GO TO 21		1 2 8<br>NEXT LINE		
12	1 2 8<br>GO TO 14		1 2 8<br>GO TO 16		1 2<br>GO TO 21		1 2<br>GO TO 21		1 2 8<br>NEXT LINE		
13	1 2 8<br>GO TO 14		1 2 8<br>GO TO 16		1 2<br>GO TO 21		1 2<br>GO TO 21		1 2 8<br>NEXT LINE		
14	1 2 8<br>GO TO 14		1 2 8<br>GO TO 16		1 2<br>GO TO 21		1 2<br>GO TO 21		1 2 8<br>NEXT LINE		
15	1 2 8<br>GO TO 14		1 2 8<br>GO TO 16		1 2<br>GO TO 21		1 2<br>GO TO 21		1 2 8<br>NEXT LINE		
16	1 2 8<br>GO TO 14		1 2 8<br>GO TO 16		1 2<br>GO TO 21		1 2<br>GO TO 21		1 2 8<br>NEXT LINE		
17	1 2 8<br>GO TO 14		1 2 8<br>GO TO 16		1 2<br>GO TO 21		1 2<br>GO TO 21		1 2 8<br>NEXT LINE		
18	1 2 8<br>GO TO 14		1 2 8<br>GO TO 16		1 2<br>GO TO 21		1 2<br>GO TO 21		1 2 8<br>NEXT LINE		
19	1 2 8<br>GO TO 14		1 2 8<br>GO TO 16		1 2<br>GO TO 21		1 2<br>GO TO 21		1 2 8<br>NEXT LINE		
20	1 2 8<br>GO TO 14		1 2 8<br>GO TO 16		1 2<br>GO TO 21		1 2<br>GO TO 21		1 2 8<br>NEXT LINE		

CODES FOR Qs. 17 AND 19: EDUCATION

LEVEL	EDUCATION YEAR
0 = PRESCHOOL	01-03 = YEARS AT PRE-PRIMARY/KINDERGARTEN
1 = PRIMARY	01-06 = YEARS 1-6 AT PRIMARY LEVEL
2 = SECONDARY	01-06 = YEARS 1-6 AT SECONDARY LEVEL
3 = HIGHER	01 - TOTAL NUMBER OF YEARS AT HIGHER LEVEL*
8 = DON'T KNOW	00 = LESS THAN 1 YEAR COMPLETED (USE '00' FOR Q. 17 ONLY. THIS CODE IS NOT ALLOWED FOR Q. 19.)
	98 = DON'T KNOW

***FOR `HIGHER' TOTAL THE NUMBER OF YEARS AT THE POST-SECONDARY LEVEL**

HOUSEHOLD NOT SELECTED FOR MAN'S SURVEY ☐ ↓					HOUSEHOLD SELECTED FOR MAN'S SURVEY ☐ → 101				
IF AGE 5 YEARS OR OLDER									
SEEING DIFFICULTY			HEARING DIFFICULTY			OTHER FUNCTIONAL DIFFICULTIES			
**21**	**22**	**23**	**24**	**25**	**26**	**27**	**28**	**29**	**30**
Does (NAME) wear glasses or contact lenses to help them see?	I would like to know if (NAME) has difficulty seeing even when wearing glasses or contact lenses. Would you say that (NAME) has no difficulty seeing, some difficulty, a lot of difficulty, or cannot see at all?<br><br>1 = NO DIFFICULTY SEEING<br>2 = SOME DIFFICULTY<br>3 = A LOT OF DIFFICULTY<br>4 = CANNOT SEE AT ALL<br>8 = DON'T KNOW	I would like to know if (NAME) has difficulty seeing. Would you say that (NAME) has no difficulty seeing, some difficulty, a lot of difficulty, or cannot see at all?<br><br>1 = NO DIFFICULTY SEEING<br>2 = SOME DIFFICULTY<br>3 = A LOT OF DIFFICULTY<br>4 = CANNOT SEE AT ALL<br>8 = DON'T KNOW	Does (NAME) wear a hearing aid?	I would like to know if (NAME) has difficulty hearing even when using a hearing aid. Would you say that (NAME) has no difficulty hearing, some difficulty, a lot of difficulty, or cannot hear at all?<br><br>1 = NO DIFFICULTY HEARING<br>2 = SOME DIFFICULTY<br>3 = A LOT OF DIFFICULTY<br>4 = CANNOT HEAR AT ALL<br>8 = DON'T KNOW	I would like to know if (NAME) has difficulty hearing. Would you say that (NAME) has no difficulty hearing, some difficulty, a lot of difficulty, or cannot hear at all?<br><br>1 = NO DIFFICULTY HEARING<br>2 = SOME DIFFICULTY<br>3 = A LOT OF DIFFICULTY<br>4 = CANNOT HEAR AT ALL<br>8 = DON'T KNOW	I would like to know if (NAME) has difficulty communicating when using his/her usual language. Would you say that (NAME) has no difficulty understanding or being understood, some difficulty, a lot<br><br>1 = NO DIFFICULTY COMMUNICATING<br>2 = SOME DIFFICULTY<br>3 = A LOT OF DIFFICULTY<br>4 = CANNOT COMMUNI CATE AT ALL<br>8 = DON'T KNOW	I would like to know if (NAME) has difficulty remembering or concentrating. Would you say that (NAME) has no difficulty remembering or concentrating, some difficulty, a lot of difficulty, or cannot remember or concentrate at all?<br><br>1 = NO DIFFICULTY REMEMBERING/ CONCENTRATING<br>2 = SOME DIFFICULTY<br>3 = A LOT OF DIFFICULTY<br>4 = CANNOT REMEMBER/ CONCENTRATE AT ALL<br>8 = DON'T KNOW	I would like to know if (NAME) has difficulty walking or climbing steps. Would you say that (NAME) has no difficulty walking or climbing steps, some difficulty, a lot of difficulty, or cannot walk or climb steps at all?<br><br>1 = NO DIFFICULTY WALKING OR CLIMBING<br>2 = SOME DIFFICULTY<br>3 = A LOT OF DIFFICULTY<br>4 = CANNOT WALK OR CLIMB AT ALL<br>8 = DON'T KNOW	I would like to know if (NAME) has difficulty washing all over or dressing. Would you say that (NAME) has no difficulty washing all over or dressing, some difficulty, a lot of difficulty, or cannot wash all over or dress at all?<br><br>1 = NO DIFFICULTY WASHING OR DRESSING<br>2 = SOME DIFFICULTY<br>3 = A LOT OF DIFFICULTY<br>4 = CANNOT WASH OR DRESS AT ALL<br>8 = DON'T KNOW
Y N DK<br>1 2 8<br>GO TO 23	1 2 3 4 8<br>(GO TO 24)	1 2 3 4 8	Y N DK<br>1 2 8<br>GO TO 26	1 2 3 4 8<br>(GO TO 27)	1 2 3 4 8	1 2 3 4 8	1 2 3 4 8	1 2 3 4 8	1 2 3 4 8
1 2 8<br>GO TO 23	1 2 3 4 8<br>(GO TO 24)	1 2 3 4 8	1 2 8<br>GO TO 26	1 2 3 4 8<br>(GO TO 27)	1 2 3 4 8	1 2 3 4 8	1 2 3 4 8	1 2 3 4 8	1 2 3 4 8
1 2 8<br>GO TO 23	1 2 3 4 8<br>(GO TO 24)	1 2 3 4 8	1 2 8<br>GO TO 26	1 2 3 4 8<br>(GO TO 27)	1 2 3 4 8	1 2 3 4 8	1 2 3 4 8	1 2 3 4 8	1 2 3 4 8
1 2 8<br>GO TO 23	1 2 3 4 8<br>(GO TO 24)	1 2 3 4 8	1 2 8<br>GO TO 26	1 2 3 4 8<br>(GO TO 27)	1 2 3 4 8	1 2 3 4 8	1 2 3 4 8	1 2 3 4 8	1 2 3 4 8
1 2 8<br>GO TO 23	1 2 3 4 8<br>(GO TO 24)	1 2 3 4 8	1 2 8<br>GO TO 26	1 2 3 4 8<br>(GO TO 27)	1 2 3 4 8	1 2 3 4 8	1 2 3 4 8	1 2 3 4 8	1 2 3 4 8
1 2 8<br>GO TO 23	1 2 3 4 8<br>(GO TO 24)	1 2 3 4 8	1 2 8<br>GO TO 26	1 2 3 4 8<br>(GO TO 27)	1 2 3 4 8	1 2 3 4 8	1 2 3 4 8	1 2 3 4 8	1 2 3 4 8
1 2 8<br>GO TO 23	1 2 3 4 8<br>(GO TO 24)	1 2 3 4 8	1 2 8<br>GO TO 26	1 2 3 4 8<br>(GO TO 27)	1 2 3 4 8	1 2 3 4 8	1 2 3 4 8	1 2 3 4 8	1 2 3 4 8
1 2 8<br>GO TO 23	1 2 3 4 8<br>(GO TO 24)	1 2 3 4 8	1 2 8<br>GO TO 26	1 2 3 4 8<br>(GO TO 27)	1 2 3 4 8	1 2 3 4 8	1 2 3 4 8	1 2 3 4 8	1 2 3 4 8
1 2 8<br>GO TO 23	1 2 3 4 8<br>(GO TO 24)	1 2 3 4 8	1 2 8<br>GO TO 26	1 2 3 4 8<br>(GO TO 27)	1 2 3 4 8	1 2 3 4 8	1 2 3 4 8	1 2 3 4 8	1 2 3 4 8
1 2 8<br>GO TO 23	1 2 3 4 8<br>(GO TO 24)	1 2 3 4 8	1 2 8<br>GO TO 26	1 2 3 4 8<br>(GO TO 27)	1 2 3 4 8	1 2 3 4 8	1 2 3 4 8	1 2 3 4 8	1 2 3 4 8

HOUSEHOLD NOT SELECTED FOR MAN'S SURVEY ☐ HOUSEHOLD SELECTED FOR MAN'S SURVEY ☐ → 101

IF AGE 5 YEARS OR OLDER

SEEING DIFFICULTY			HEARING DIFFICULTY			OTHER FUNCTIONAL DIFFICULTIES			
**21**	**22**	**23**	**24**	**25**	**26**	**27**	**28**	**29**	**30**
Does (NAME) wear glasses or contact lenses to help them see?	I would like to know if (NAME) has difficulty seeing even when wearing glasses or contact lenses. Would you say that (NAME) has no difficulty seeing, some difficulty, a lot of difficulty, or cannot see at all?<br><br>1 = NO DIFFICULTY SEEING<br>2 = SOME DIFFICULTY<br>3 = A LOT OF DIFFICULTY<br>4 = CANNOT SEE AT ALL<br>8 = DON'T KNOW	I would like to know if (NAME) has difficulty seeing. Would you say that (NAME) has no difficulty seeing, some difficulty, a lot of difficulty, or cannot see at all?<br><br>1 = NO DIFFICULTY SEEING<br>2 = SOME DIFFICULTY<br>3 = A LOT OF DIFFICULTY<br>4 = CANNOT SEE AT ALL<br>8 = DON'T KNOW	Does (NAME) wear a hearing aid?	I would like to know if (NAME) has difficulty hearing even when using a hearing aid. Would you say that (NAME) has no difficulty hearing, some difficulty, a lot of difficulty, or cannot hear at all?<br><br>1 = NO DIFFICULTY HEARING<br>2 = SOME DIFFICULTY<br>3 = A LOT OF DIFFICULTY<br>4 = CANNOT HEAR AT ALL<br>8 = DON'T KNOW	I would like to know if (NAME) has difficulty hearing. Would you say that (NAME) has no difficulty hearing, some difficulty, a lot of difficulty, or cannot hear at all?<br><br>1 = NO DIFFICULTY HEARING<br>2 = SOME DIFFICULTY<br>3 = A LOT OF DIFFICULTY<br>4 = CANNOT HEAR AT ALL<br>8 = DON'T KNOW	I would like to know if (NAME) has difficulty communicating when using his/her usual language. Would you say that (NAME) has no difficulty understanding or being understood, some difficulty, a lot<br><br>1 = NO DIFFICULTY COMMUNICATING<br>2 = SOME DIFFICULTY<br>3 = A LOT OF DIFFICULTY<br>4 = CANNOT COMMUNI CATE AT ALL<br>8 = DON'T KNOW	I would like to know if (NAME) has difficulty remembering or concentrating. Would you say that (NAME) has no difficulty remembering or concentrating, some difficulty, a lot of difficulty, or cannot remember or concentrate at all?<br><br>1 = NO DIFFICULTY REMEMBERING/ CONCENTRATING<br>2 = SOME DIFFICULTY<br>3 = A LOT OF DIFFICULTY<br>4 = CANNOT REMEMBER/ CONCENTRATE AT ALL<br>8 = DON'T KNOW	I would like to know if (NAME) has difficulty walking or climbing steps. Would you say that (NAME) has no difficulty walking or climbing steps, some difficulty, a lot of difficulty, or cannot walk or climb steps at all?<br><br>1 = NO DIFFICULTY WALKING OR CLIMBING<br>2 = SOME DIFFICULTY<br>3 = A LOT OF DIFFICULTY<br>4 = CANNOT WALK OR CLIMB AT ALL<br>8 = DON'T KNOW	I would like to know if (NAME) has difficulty washing all over or dressing. Would you say that (NAME) has no difficulty washing all over or dressing, some difficulty, a lot of difficulty, or cannot wash all over or dress at all?<br><br>1 = NO DIFFICULTY WASHING OR DRESSING<br>2 = SOME DIFFICULTY<br>3 = A LOT OF DIFFICULTY<br>4 = CANNOT WASH OR DRESS AT ALL<br>8 = DON'T KNOW
Y N DK<br>1 2 8<br>GO TO 23	1 2 3 4 8<br>(GO TO 24)	1 2 3 4 8	Y N DK<br>1 2 8<br>GO TO 26	1 2 3 4 8<br>(GO TO 27)	1 2 3 4 8	1 2 3 4 8	1 2 3 4 8	1 2 3 4 8	1 2 3 4 8
1 2 8<br>GO TO 23	1 2 3 4 8<br>(GO TO 24)	1 2 3 4 8	1 2 8<br>GO TO 26	1 2 3 4 8<br>(GO TO 27)	1 2 3 4 8	1 2 3 4 8	1 2 3 4 8	1 2 3 4 8	1 2 3 4 8
1 2 8<br>GO TO 23	1 2 3 4 8<br>(GO TO 24)	1 2 3 4 8	1 2 8<br>GO TO 26	1 2 3 4 8<br>(GO TO 27)	1 2 3 4 8	1 2 3 4 8	1 2 3 4 8	1 2 3 4 8	1 2 3 4 8
1 2 8<br>GO TO 23	1 2 3 4 8<br>(GO TO 24)	1 2 3 4 8	1 2 8<br>GO TO 26	1 2 3 4 8<br>(GO TO 27)	1 2 3 4 8	1 2 3 4 8	1 2 3 4 8	1 2 3 4 8	1 2 3 4 8
1 2 8<br>GO TO 23	1 2 3 4 8<br>(GO TO 24)	1 2 3 4 8	1 2 8<br>GO TO 26	1 2 3 4 8<br>(GO TO 27)	1 2 3 4 8	1 2 3 4 8	1 2 3 4 8	1 2 3 4 8	1 2 3 4 8
1 2 8<br>GO TO 23	1 2 3 4 8<br>(GO TO 24)	1 2 3 4 8	1 2 8<br>GO TO 26	1 2 3 4 8<br>(GO TO 27)	1 2 3 4 8	1 2 3 4 8	1 2 3 4 8	1 2 3 4 8	1 2 3 4 8
1 2 8<br>GO TO 23	1 2 3 4 8<br>(GO TO 24)	1 2 3 4 8	1 2 8<br>GO TO 26	1 2 3 4 8<br>(GO TO 27)	1 2 3 4 8	1 2 3 4 8	1 2 3 4 8	1 2 3 4 8	1 2 3 4 8
1 2 8<br>GO TO 23	1 2 3 4 8<br>(GO TO 24)	1 2 3 4 8	1 2 8<br>GO TO 26	1 2 3 4 8<br>(GO TO 27)	1 2 3 4 8	1 2 3 4 8	1 2 3 4 8	1 2 3 4 8	1 2 3 4 8
1 2 8<br>GO TO 23	1 2 3 4 8<br>(GO TO 24)	1 2 3 4 8	1 2 8<br>GO TO 26	1 2 3 4 8<br>(GO TO 27)	1 2 3 4 8	1 2 3 4 8	1 2 3 4 8	1 2 3 4 8	1 2 3 4 8
1 2 8<br>GO TO 23	1 2 3 4 8<br>(GO TO 24)	1 2 3 4 8	1 2 8<br>GO TO 26	1 2 3 4 8<br>(GO TO 27)	1 2 3 4 8	1 2 3 4 8	1 2 3 4 8	1 2 3 4 8	1 2 3 4 8

HOUSEHOLD CHARACTERISTICS

NO.	QUESTIONS AND FILTERS	CODING CATEGORIES	SKIP
101	What is the main source of drinking water for members of your household?	**PIPED WATER**<br>PIPED INTO DWELLING .... 11<br>PIPED TO YARD/PLOT .... 12<br>PIPED TO NEIGHBOR .... 13<br>PUBLIC TAP/STANDPIPE .... 14<br><br>TUBE WELL OR BOREHOLE .... 21<br>**DUG WELL**<br>PROTECTED WELL .... 31<br>UNPROTECTED WELL .... 32<br>**WATER FROM SPRING**<br>PROTECTED SPRING .... 41<br>UNPROTECTED SPRING .... 42<br><br>RAINWATER .... 51<br>TANKER TRUCK .... 61<br>CART WITH SMALL TANK .... 71<br>SURFACE WATER (RIVER/DAM/ LAKE/POND/STREAM/CANAL/ IRRIGATION CHANNEL) .... 81<br><br>BOTTLED WATER .... 91<br>SACHET WATER .... 92<br><br>OTHER ______________ 96<br>(SPECIFY)	11, 12, 13 → 106<br>14–81 → 103<br>96 → 103
102	What is the main source of water used by your household for other purposes such as cooking and handwashing?	**PIPED WATER**<br>PIPED INTO DWELLING .... 11<br>PIPED TO YARD/PLOT .... 12<br>PIPED TO NEIGHBOR .... 13<br>PUBLIC TAP/STANDPIPE .... 14<br><br>TUBE WELL OR BOREHOLE .... 21<br>**DUG WELL**<br>PROTECTED WELL .... 31<br>UNPROTECTED WELL .... 32<br>**WATER FROM SPRING**<br>PROTECTED SPRING .... 41<br>UNPROTECTED SPRING .... 42<br><br>RAINWATER .... 51<br>TANKER TRUCK .... 61<br>CART WITH SMALL TANK .... 71<br>SURFACE WATER (RIVER/DAM/ LAKE/POND/STREAM/CANAL/ IRRIGATION CHANNEL) .... 81<br><br>OTHER ______________ 96<br>(SPECIFY)	11, 12, 13 → 106
103	Where is that water source located?	IN OWN DWELLING .... 1<br>IN OWN YARD/PLOT .... 2<br>ELSEWHERE 3	1, 2 → 105
104	How long does it take to go there, get water, and come back?	MINUTES .... [ ][ ][ ]<br><br>DON'T KNOW 998	
105	CHECK 101 AND 102: CODE '14' OR '21' CIRCLED?<br>YES [ ] ↓	NO [ ]	→ 107

HOUSEHOLD CHARACTERISTICS

NO.	QUESTIONS AND FILTERS	CODING CATEGORIES	SKIP
106	In the past two weeks, was the water from this source not available for at least one full day?	YES . . . . 1<br>NO . . . . 2<br>DON'T KNOW 8	
107	Do you do anything to the water to make it safer to drink?	YES . . . . 1<br>NO . . . . 2<br>DON'T KNOW 8	 NO, DON'T KNOW → 109
108	What do you usually do to make the water safer to drink?<br><br>Anything else?<br><br>RECORD ALL MENTIONED.	BOIL . . . . A<br>ADD BLEACH/CHLORINE . . . . B<br>STRAIN THROUGH A CLOTH . . . . C<br>USE WATER FILTER (CERAMIC/<br>SAND/COMPOSITE/ETC) . . . . D<br>SOLAR DISINFECTION . . . . E<br>LET IT STAND AND SETTLE . . . . F<br>ALUM . . . . G<br>OTHER ______ X<br>(SPECIFY)<br>DON'T KNOW . . . . Z	
109	What kind of toilet facility do members of your household usually use?<br><br>IF NOT POSSIBLE TO DETERMINE, ASK PERMISSION TO OBSERVE THE FACILITY.	**FLUSH OR POUR FLUSH TOILET**<br>FLUSH TO PIPED SEWER SYSTEM . . . . 11<br>FLUSH TO SEPTIC TANK . . . . 12<br>FLUSH TO PIT LATRINE . . . . 13<br>FLUSH TO SOMEWHERE ELSE . . . . 14<br>FLUSH, DON'T KNOW WHERE . . . . 15<br>**PIT LATRINE**<br>VENTILATED IMPROVED PIT LATRINE . . . . 21<br>PIT LATRINE WITH SLAB . . . . 22<br>PIT LATRINE WITHOUT SLAB/OPEN PIT . . . . 23<br><br>COMPOSTING TOILET . . . . 31<br>BUCKET TOILET . . . . 41<br>HANGING TOILET/HANGING LATRINE . . . . 51<br>NO FACILITY/BUSH/FIELD . . . . 61<br>OTHER ______ 96<br>(SPECIFY)	 <br><br><br><br><br><br><br><br><br><br><br><br>NO FACILITY/BUSH/FIELD → 113
110	Do you share this toilet facility with other households?	YES . . . . 1<br>NO 2	 NO → 112
111	Including your own household, how many households use this toilet facility?	NO. OF HOUSEHOLDS<br>IF LESS THAN 10 . . . . [0][ ]<br><br>10 OR MORE HOUSEHOLDS . . . . 95<br>DON'T KNOW 98	
112	Where is this toilet facility located?	IN OWN DWELLING . . . . 1<br>IN OWN YARD/PLOT . . . . 2<br>ELSEWHERE 3	

NO.	QUESTIONS AND FILTERS	CODING CATEGORIES	SKIP
113	What type of fuel does your household mainly use for cooking?	ELECTRICITY . . . 01<br>LPG . . . 02<br>NATURAL GAS . . . 03<br>BIOGAS . . . 04<br>KEROSENE . . . 05<br>COAL, LIGNITE . . . 06<br>CHARCOAL . . . 07<br>WOOD . . . 08<br>STRAW/SHRUBS/GRASS . . . 09<br>AGRICULTURAL CROP . . . 10<br>ANIMAL DUNG . . . 11<br><br>NO FOOD COOKED IN HOUSEHOLD . . . 95<br><br>OTHER ______ (SPECIFY) 96	95 → 116
114	Is the cooking usually done in the house, in a separate building, or outdoors?	IN THE HOUSE . . . 1<br>IN A SEPARATE BUILDING . . . 2<br>OUTDOORS . . . 3<br><br>OTHER ______ (SPECIFY) 6	2, 3, 6 → 116
115	Do you have a separate room which is used as a kitchen?	YES . . . 1<br>NO . . . 2	
116	How many rooms in this household are used for sleeping?	ROOMS . . . [ ][ ]	
117	Does this household own any livestock, herds, other farm animals, or poultry?	YES . . . 1<br>NO . . . 2	2 → 119
118	How many of the following animals does this household own?<br>IF NONE, RECORD '00'.<br>IF 95 OR MORE, RECORD '95'.<br>IF UNKNOWN, RECORD '98'.<br><br>a) Milk cows or bulls?<br>b) Other cattle?<br>c) Horses, donkeys, or mules?<br>d) Goats?<br>e) Sheep?<br>f) Chickens or other poultry?<br>g) Pigs?<br>h) Camels?	a) COWS/BULLS . . . [ ][ ]<br>b) OTHER CATTLE . . . [ ][ ]<br>c) HORSES/DONKEYS/MULES . . . [ ][ ]<br>d) GOATS . . . [ ][ ]<br>e) SHEEP . . . [ ][ ]<br>f) CHICKENS/POULTRY . . . [ ][ ]<br>f) PIGS . . . [ ][ ]<br>f) CAMEL . . . [ ][ ]	
119	Does any member of this household own any agricultural land?	YES . . . 1<br>NO . . . 2	2 → 121
120	How many plot/acres/hectares of agricultural land do members of this household own?<br><br>IF 95 OR MORE, CIRCLE '9950'.	PLOT . . . 1 [ ][ ].[ ]<br>ACRES . . . 2 [ ][ ].[ ]<br>HECTARES . . . 3 [ ][ ].[ ]<br><br>95 OR MORE PLOT/ACRES/HECTARES . . 9950<br>DON'T KNOW . . . 9998	

HOUSEHOLD CHARACTERISTICS

NO.	QUESTIONS AND FILTERS	CODING CATEGORIES	SKIP
121	Does your household have:<br>a) Electricity?<br>b) A radio?<br>c) A television?<br>d) A non-mobile telephone?<br>e) A computer?<br>f) A refrigerator?<br>g) A table?<br>h) A chair?<br>i) A bed?<br>j) A sofa?<br>k) A cupboard?<br>l) An air conditioner?<br>m) An electric iron?<br>n) A generator?<br>o) A fan?	YES NO<br>a) ELECTRICITY . . . . 1 2<br>b) RADIO . . . . 1 2<br>c) TELEVISION . . . . 1 2<br>d) NON-MOBILE TELEPHONE . . 1 2<br>e) COMPUTER . . . . 1 2<br>f) REFRIGERATOR . . . . 1 2<br>g) TABLE . . . . 1 2<br>h) CHAIR . . . . 1 2<br>i) BED . . . . 1 2<br>j) SOFA . . . . 1 2<br>k) CUPBOARD . . . . 1 2<br>l) AIR CONDITIONER . . . . 1 2<br>m) ELECTRIC IRON . . . . 1 2<br>n) GENERATOR . . . . 1 2<br>o) FAN 1 2	
122	Does any member of this household own:<br>a) A watch?<br>b) A mobile phone?<br>c) A bicycle?<br>d) A motorcycle or motor scooter?<br>e) An animal-drawn cart?<br>f) A car or truck?<br>g) A boat with a motor?<br>h) A canoe?<br>i) A Keke Napep?	YES NO<br>a) WATCH . . . . 1 2<br>b) MOBILE PHONE . . . . 1 2<br>c) BICYCLE . . . . 1 2<br>d) MOTORCYCLE/SCOOTER . . . . . 1 2<br>e) ANIMAL-DRAWN CART . . . . . 1 2<br>f) CAR/TRUCK . . . . 1 2<br>g) BOAT WITH MOTOR . . . . 1 2<br>h) CANOE . . . . 1 2<br>i) KEKE - NAPEP 1 2	
123	Does any member of this household have a bank account?	YES . . . . 1<br>NO 2	
124	How often does anyone smoke inside your house? Would you say daily, weekly, monthly, less often than once a month, or never?	DAILY . . . . 1<br>WEEKLY . . . . 2<br>MONTHLY . . . . 3<br>LESS OFTEN THAN ONCE A MONTH . . . . 4<br>NEVER 5	
127	Does your household have any mosquito nets?	YES . . . . 1<br>NO 2	 → 139
128	How many mosquito nets does your household have?<br>IF 7 OR MORE NETS, RECORD '7'.	NUMBER OF NETS . . . . ☐	

MOSQUITO NETS

		NET #1	NET #2	NET #3
129	ASK THE RESPONDENT TO SHOW YOU ALL THE NETS IN THE HOUSEHOLD.<br><br>IF MORE THAN 3 NETS, USE ADDITIONAL QUESTIONNAIRE(S).	OBSERVED, HANGING . . 1<br>OBSERVED, NOT HANGING . . . . 2<br>NOT OBSERVED 3	OBSERVED, HANGING . . 1<br>OBSERVED, NOT HANGING . . . . 2<br>NOT OBSERVED 3	OBSERVED, HANGING . . 1<br>OBSERVED, NOT HANGING . . . . 2<br>NOT OBSERVED 3
130	How many months ago did your household get the mosquito net?<br><br>IF LESS THAN ONE MONTH AGO, RECORD '00'.	MONTHS AGO . . . . . [ ][ ]<br><br>MORE THAN 36 MONTHS AGO . . . . . 95<br><br>NOT SURE 98	MONTHS AGO . . . . . [ ][ ]<br><br>MORE THAN 36 MONTHS AGO . . . . . 95<br><br>NOT SURE 98	MONTHS AGO . . . . . [ ][ ]<br><br>MORE THAN 36 MONTHS AGO . . . . . 95<br><br>NOT SURE 98
131	OBSERVE OR ASK BRAND/TYPE OF MOSQUITO NET.<br><br>IF BRAND IS UNKNOWN AND YOU CANNOT OBSERVE THE NET, SHOW PICTURES OF TYPICAL NET TYPES/BRANDS TO RESPONDENT.	**LONG-LASTING INSECTICIDE-TREATED NET (LLIN)**<br>PERMANET . . . . 11<br>OLYSET . . . . 12<br>ICONLIFE . . . . 13<br>DURANET . . . . 14<br>NETPROTEC . . . . 15<br>BASF INTERCEPTC . . 17<br>YORKOOL . . . . 18<br><br>OTHER/DON'T KNOW BRAND . . . . 16<br><br>OTHER TYPE . . . . 96<br>DON'T KNOW TYPE . . 98	**LONG-LASTING INSECTICIDE-TREATED NET (LLIN)**<br>PERMANET . . . . 11<br>OLYSET . . . . 12<br>ICONLIFE . . . . 13<br>DURANET . . . . 14<br>NETPROTEC . . . . 15<br>BASF INTERCEPTC . . 17<br>YORKOOL . . . . 18<br><br>OTHER/DON'T KNOW BRAND . . . . 16<br><br>OTHER TYPE . . . . 96<br>DON'T KNOW TYPE . . 98	**LONG-LASTING INSECTICIDE-TREATED NET (LLIN)**<br>PERMANET . . . . 11<br>OLYSET . . . . 12<br>ICONLIFE . . . . 13<br>DURANET . . . . 14<br>NETPROTEC . . . . 15<br>BASF INTERCEPTC . . 17<br>YORKOOL . . . . 18<br><br>OTHER/DON'T KNOW BRAND . . . . 16<br><br>OTHER TYPE . . . . 96<br>DON'T KNOW TYPE . . 98
134	Did you get the net through a net mass distribution campaign, during an antenatal care visit, or during an immunization visit?	YES, DISTRIBUTION CAMPAIGN . . . . 1<br>YES, ANC . . . . 2<br>YES, IMMUNIZATION VISIT . . . . 3<br>(SKIP TO 136)<br>NO 4	YES, DISTRIBUTION CAMPAIGN . . . . 1<br>YES, ANC . . . . 2<br>YES, IMMUNIZATION VISIT . . . . 3<br>(SKIP TO 136)<br>NO 4	YES, DISTRIBUTION CAMPAIGN . . . . 1<br>YES, ANC . . . . 2<br>YES, IMMUNIZATION VISIT . . . . 3<br>(SKIP TO 136)<br>NO 4
135	Where did you get the net?	GOVT. HEALTH FACILITY . . . . 01<br>PRIVATE HEALTH FACILITY . . . . 02<br>PHARMACY . . . . 03<br>SHOP/MARKET . . . . . 04<br>COMMUNITY HEALTH WORKER . . . . 05<br>RELIGIOUS INSTITUTION . . . . . 06<br>SCHOOL . . . . 07<br>OTHER . . . . 96<br>DON'T KNOW 98	GOVT. HEALTH FACILITY . . . . 01<br>PRIVATE HEALTH FACILITY . . . . 02<br>PHARMACY . . . . 03<br>SHOP/MARKET . . . . . 04<br>COMMUNITY HEALTH WORKER . . . . 05<br>RELIGIOUS INSTITUTION . . . . . 06<br>SCHOOL . . . . 07<br>OTHER . . . . 96<br>DON'T KNOW 98	GOVT. HEALTH FACILITY . . . . 01<br>PRIVATE HEALTH FACILITY . . . . 02<br>PHARMACY . . . . 03<br>SHOP/MARKET . . . . . 04<br>COMMUNITY HEALTH WORKER . . . . 05<br>RELIGIOUS INSTITUTION . . . . . 06<br>SCHOOL . . . . 07<br>OTHER . . . . 96<br>DON'T KNOW 98

MOSQUITO NETS

		NET #1	NET #2	NET #3
136	Did anyone sleep inside this mosquito net last night?	YES . . . . 1 (SKIP TO 137)<br>NO . . . . 2<br>NOT SURE . . . . 8 (SKIP TO 138)	YES . . . . 1 (SKIP TO 137)<br>NO . . . . 2<br>NOT SURE . . . . 8 (SKIP TO 138)	YES . . . . 1 (SKIP TO 137)<br>NO . . . . 2<br>NOT SURE . . . . 8 (SKIP TO 138)
136A	Why did not anyone sleep inside this net?	NO MOSQUITOES . . . . . 01<br>NO MALARIA . . . . 02<br>TOO HOT . . . . 03<br>DIFFICULT TO HANG . . 04<br>DON'T LIKE SMELL . . . . . 05<br>FEEL `CLOSED IN' OR CONSTRAINED . . 06<br>NET TOO OLD/TORN . . 07<br>NET TOO DIRTY . . . . 08<br>NET NOT AVAILABLE LAST NIGHT (WASHING) . . 09<br>FEEL ITN CHEMICALS ARE UNSAFE . . . . 10<br>ITN PROVOKES COUGH 11<br>USERS DID NOT SLEEP HERE LAST NIGHT . . 12<br>NET NOT NEEDED LAST NIGHT . . . . 13<br>NO SPACE TO HANG . . 14<br>OTHER ________ 96 (SPECIFY)<br>DON'T KNOW . . . . 98<br>(SKIP TO 138)	NO MOSQUITOES . . . . . 01<br>NO MALARIA . . . . 02<br>TOO HOT . . . . 03<br>DIFFICULT TO HANG . . 04<br>DON'T LIKE SMELL . . . . . 05<br>FEEL `CLOSED IN' OR CONSTRAINED . . 06<br>NET TOO OLD/TORN . . 07<br>NET TOO DIRTY . . . . 08<br>NET NOT AVAILABLE LAST NIGHT (WASHING) . . 09<br>FEEL ITN CHEMICALS ARE UNSAFE . . . . 10<br>ITN PROVOKES COUGH 11<br>USERS DID NOT SLEEP HERE LAST NIGHT . . 12<br>NET NOT NEEDED LAST NIGHT . . . . 13<br>NO SPACE TO HANG . . 14<br>OTHER ________ 96 (SPECIFY)<br>DON'T KNOW . . . . 98<br>(SKIP TO 138)	NO MOSQUITOES . . . . . 01<br>NO MALARIA . . . . 02<br>TOO HOT . . . . 03<br>DIFFICULT TO HANG . . 04<br>DON'T LIKE SMELL . . . . . 05<br>FEEL `CLOSED IN' OR CONSTRAINED . . 06<br>NET TOO OLD/TORN . . 07<br>NET TOO DIRTY . . . . 08<br>NET NOT AVAILABLE LAST NIGHT (WASHING) . . 09<br>FEEL ITN CHEMICALS ARE UNSAFE . . . . 10<br>ITN PROVOKES COUGH 11<br>USERS DID NOT SLEEP HERE LAST NIGHT . . 12<br>NET NOT NEEDED LAST NIGHT . . . . 13<br>NO SPACE TO HANG . . 14<br>OTHER ________ 96 (SPECIFY)<br>DON'T KNOW . . . . 98<br>(SKIP TO 138)
137	Who slept inside this mosquito net last night?<br><br>RECORD THE PERSON'S NAME AND LINE NUMBER FROM HOUSEHOLD SCHEDULE.	NAME ________<br>LINE NO. . . . . . [ ][ ]<br>NAME ________<br>LINE NO. . . . . . [ ][ ]<br>NAME ________<br>LINE NO. . . . . . [ ][ ]<br>NAME ________<br>LINE NO. . . . . . [ ][ ]	NAME ________<br>LINE NO. . . . . . [ ][ ]<br>NAME ________<br>LINE NO. . . . . . [ ][ ]<br>NAME ________<br>LINE NO. . . . . . [ ][ ]<br>NAME ________<br>LINE NO. . . . . . [ ][ ]	NAME ________<br>LINE NO. . . . . . [ ][ ]<br>NAME ________<br>LINE NO. . . . . . [ ][ ]<br>NAME ________<br>LINE NO. . . . . . [ ][ ]<br>NAME ________<br>LINE NO. . . . . . [ ][ ]
138		GO BACK TO 129 FOR NEXT NET; OR, IF NO MORE NETS, GO TO 139.	GO BACK TO 129 FOR NEXT NET; OR, IF NO MORE NETS, GO TO 139.	GO TO 129 IN FIRST COLUMN OF A NEW QUESTIONNAIRE; OR, IF NO MORE NETS, GO TO 139.

ADDITIONAL HOUSEHOLD CHARACTERISTICS

NO.	QUESTIONS AND FILTERS	CODING CATEGORIES	SKIP
139	We would like to learn about the places that households use to wash their hands. Can you please show me where members of your household most often wash their hands?	OBSERVED, FIXED PLACE . . . . 1<br>OBSERVED, MOBILE . . . . 2<br>NOT OBSERVED,<br>NOT IN DWELLING/YARD/PLOT . . . . 3<br>NOT OBSERVED, NO PERMISSION TO SEE . . . . . 4<br>NOT OBSERVED, OTHER REASON 5	3, 4, 5 → 142
140	OBSERVE PRESENCE OF WATER AT THE PLACE FOR HANDWASHING.<br><br>RECORD OBSERVATION.	WATER IS AVAILABLE . . . . 1<br>WATER IS NOT AVAILABLE 2	
141	OBSERVE PRESENCE OF SOAP, DETERGENT, OR OTHER CLEANSING AGENT AT THE PLACE FOR HANDWASHING.<br><br>RECORD OBSERVATION.	SOAP OR DETERGENT<br>(BAR, LIQUID, POWDER, PASTE) . . . . A<br>ASH, MUD, SAND . . . . B<br><br>NONE . . . . Y	
142	OBSERVE MAIN MATERIAL OF THE FLOOR OF THE DWELLING.<br><br>RECORD OBSERVATION.	**NATURAL FLOOR**<br>EARTH/SAND . . . . 11<br>DUNG . . . . 12<br>**RUDIMENTARY FLOOR**<br>WOOD PLANKS . . . . 21<br>PALM/BAMBOO . . . . 22<br>**FINISHED FLOOR**<br>PARQUET OR POLISHED WOOD . . . . 31<br>VINYL OR ASPHALT STRIPS . . . . 32<br>CERAMIC TILES . . . . 33<br>CEMENT . . . . 34<br>CARPET/RU(. . . . 35<br><br>OTHER ________________________ 96<br>(SPECIFY)	
143	OBSERVE MAIN MATERIAL OF THE ROOF OF THE DWELLING.<br><br>RECORD OBSERVATION.	**NATURAL ROOFING**<br>NO ROOF . . . . 11<br>THATCH/PALM LEAF . . . . 12<br><br>**RUDIMENTARY ROOFING**<br>RUSTIC MAT . . . . 21<br>PALM/BAMBOO . . . . 22<br>WOOD PLANKS . . . . 23<br>CARDBOARD . . . . 24<br>**FINISHED ROOFING**<br>METAL/ZINC . . . . 31<br>WOOD . . . . 32<br>CALAMINE/CEMENT FIBER . . . . 33<br>CERAMIC TILES . . . . 34<br>CEMENT . . . . 35<br>ROOFING SHINGLES . . . . 36<br><br>OTHER ________________________ 96<br>(SPECIFY)	

ADDITIONAL HOUSEHOLD CHARACTERISTICS

NO.	QUESTIONS AND FILTERS	CODING CATEGORIES	SKIP
144	OBSERVE MAIN MATERIAL OF THE EXTERIOR WALLS OF THE DWELLING.<br><br>RECORD OBSERVATION.	**NATURAL WALLS**<br>NO WALLS . . . . . 11<br>CANE/PALM/TRUNKS . . . . . 12<br>DIRT . . . . . 13<br>**RUDIMENTARY WALLS**<br>BAMBOO WITH MUD . . . . . 21<br>STONE WITH MUD . . . . . 22<br>UNCOVERED ADOBE . . . . . 23<br>PLYWOOD . . . . . 24<br>CARDBOARD . . . . . 25<br>REUSED WOOD . . . . . 26<br>**FINISHED WALLS**<br>CEMENT . . . . . 31<br>STONE WITH LIME/CEMENT . . . . . 32<br>BRICKS . . . . . 33<br>CEMENT BLOCKS . . . . . 34<br>COVERED ADOBE . . . . . 35<br>WOOD PLANKS/SHINGLES . . . . . 36<br><br>OTHER ______ 96<br>(SPECIFY)	
145	I would like to check whether the salt used in your household is iodized. May I have a sample of the salt used to cook meals in your household?<br><br>TEST SALT FOR IODINE.	IODINE PRESENT . . . . . 1<br>NO IODINE . . . . . 2<br><br>NO SALT IN HOUSEHOLD . . . . . 3<br><br>SALT NOT TESTED ______ 6<br>(SPECIFY REASON)	
146	RECORD THE TIME.	HOURS . . . . . [ ][ ]<br><br>MINUTES . . . . . [ ][ ]	

INTERVIEWER'S OBSERVATIONS

TO BE FILLED IN AFTER COMPLETING INTERVIEW

COMMENTS ABOUT INTERVIEW:

COMMENTS ON SPECIFIC QUESTIONS:

ANY OTHER COMMENTS:

SUPERVISOR'S OBSERVATIONS

EDITOR'S OBSERVATIONS

FORMATTING DATE: 07 Jun 2017
ENGLISH LANGUAGE: 26 May 2018

NIGERIA DEMOGRAPHIC AND HEALTH SURVEY - 2018
WOMAN'S QUESTIONNAIRE

NIGERIA
NATIONAL POPULATION COMMISSION

IDENTIFICATION

STATE ______

LOCAL GOVT. AREA ______

LOCALITY ______

ENUMERATION AREA ______

NAME OF HOUSEHOLD HEAD ______

CLUSTER NUMBER . . . .

HOUSEHOLD NUMBER . . . .

NAME AND LINE NUMBER OF WOMAN ______

CHECK COVER PAGE OF HOUSEHOLD QUESTIONNAIRE: HOUSEHOLD SELECTED FOR MAN'S SURVEY? (1=YES, 2=NO)

CHECK HOUSEHOLD QUESTIONNAIRE DVH01: WOMAN SELECTED FOR DV MODULE? (1=YES, 2=NO)

INTERVIEWER VISITS

	1	2	3	FINAL VISIT
DATE	______	______	______	DAY<br>MONTH<br>YEAR
INTERVIEWER'S NAME	______	______	______	INT. NO.
RESULT*	______	______	______	RESULT*
NEXT VISIT: DATE	______	______		TOTAL NUMBER OF VISITS
TIME	______	______		

*RESULT CODES: 1 COMPLETED, 2 NOT AT HOME, 3 POSTPONED, 4 REFUSED, 5 PARTLY COMPLETED, 6 INCAPACITATED, 7 OTHER ______ (SPECIFY)

LANGUAGE OF QUESTIONNAIRE** 0 1 | LANGUAGE OF INTERVIEW** | NATIVE LANGUAGE OF RESPONDENT** | TRANSLATOR USED (YES = 1, NO = 2)

LANGUAGE OF QUESTIONNAIRE** **ENGLISH**

**LANGUAGE CODES:
01 ENGLISH
02 HAUSA
03 YORUBA
04 IGBO

SUPERVISOR		FIELD EDITOR	
NAME	NUMBER	NAME	NUMBER

INTRODUCTION AND CONSENT

Hello. My name is ______________________________. I am working with National Population Commission. We are conducting a survey about health and other topics all over Nigeria. The information we collect will help the government to plan health services. Your household was selected for the survey. The questions usually take about 30 to 60 minutes. All of the answers you give will be confidential and will not be shared with anyone other than members of our survey team. You don't have to be in the survey, but we hope you will agree to answer the questions since your views are important. If I ask you any question you don't want to answer, just let me know and I will go on to the next question or you can stop the interview at any time.

In case you need more information about the survey, you may contact the person listed on the card that has already been given to your household.

Do you have any questions?
May I begin the interview now?

SIGNATURE OF INTERVIEWER ______________________________ DATE ____________________

RESPONDENT AGREES TO BE INTERVIEWED . . 1 ↓ RESPONDENT DOES NOT AGREE TO BE INTERVIEWED . . 2 → END

SECTION 1. RESPONDENT'S BACKGROUND

NO.	QUESTIONS AND FILTERS	CODING CATEGORIES	SKIP
101	RECORD THE TIME.	HOURS . . . . [ ][ ]<br>MINUTES . . . . [ ][ ]	
102	How long have you been living continuously in (NAME OF CURRENT CITY, TOWN OR VILLAGE OF RESIDENCE)?<br><br>IF LESS THAN ONE YEAR, RECORD '00' YEARS.	YEARS . . . . [ ][ ]<br>ALWAYS . . . . 95<br>VISITOR 96	95, 96 → 105
103	Just before you moved here, did you live in a city, in a town, or in a rural area?	CITY . . . . 1<br>TOWN . . . . 2<br>RURAL AREA 3	
104	Before you moved here, which state did you live in?	ABIA . . . . 01<br>ADAMAWA . . . . 02<br>AKWA IBOM . . . . 03<br>ANAMBRA . . . . 04<br>BAUCHI . . . . 05<br>BAYELSA . . . . 06<br>BENUE . . . . 07<br>BORNO . . . . 08<br>CROSS RIVER . . . . 09<br>DELTA . . . . 10<br>EBONYI . . . . 11<br>EDO . . . . 12<br>EKITI . . . . 13<br>ENUGU . . . . 14<br>FCT-ABUJA . . . . 15<br>GOMBE . . . . 16<br>IMO . . . . 17<br>JIGAWA . . . . 18<br>KADUNA . . . . 19<br>KANO . . . . 20<br>KATSINA . . . . 21<br>KEBBI . . . . 22<br>KOGI . . . . 23<br>KWARA . . . . 24<br>LAGOS . . . . 25<br>NASARAWA . . . . 26<br>NIGER . . . . 27<br>OGUN . . . . 28<br>ONDO . . . . 29<br>OSUN . . . . 30<br>OYO . . . . 31<br>PLATEAU . . . . 32<br>RIVERS 33	

NO.	QUESTIONS AND FILTERS	CODING CATEGORIES	SKIP
		SOKOTO . . . 34<br>TARABA . . . 35<br>YOBE . . . 36<br>ZAMFARA . . . 37<br><br>OUTSIDE OF NIGERIA . . . 96	
105	In what month and year were you born?	MONTH . . . [ ][ ]<br><br>DON'T KNOW MONTH . . . 98<br><br>YEAR . . . [ ][ ][ ][ ]<br><br>DON'T KNOW YEAR . . . 9998	
106	How old were you at your last birthday?<br><br>COMPARE AND CORRECT 105 AND/OR 106 IF INCONSISTENT.	AGE IN COMPLETED YEARS . . . [ ][ ]	
107	Have you ever attended school?	YES . . . 1<br>NO . . . 2	 → 111
108	What is the highest level of school you attended: primary, secondary, or higher?	PRIMARY . . . 1<br>SECONDARY . . . 2<br>HIGHER . . . 3	
109	What is the highest (class/year) you completed at that level?<br><br>IF COMPLETED LESS THAN ONE YEAR AT THAT LEVEL, RECORD '00'.	CLASS/YEAR . . . [ ][ ]	
110	CHECK 108:<br>PRIMARY OR SECONDARY [ ] ↓	HIGHER [ ]	→ 113
111	Now I would like you to read this sentence to me.<br><br>SHOW CARD TO RESPONDENT.<br><br>IF RESPONDENT CANNOT READ WHOLE SENTENCE,<br>PROBE: Can you read any part of the sentence to me?	CANNOT READ AT ALL . . . 1<br>ABLE TO READ ONLY PART OF THE SENTENCE . . . 2<br>ABLE TO READ WHOLE SENTENCE . . . 3<br>NO CARD WITH REQUIRED LANGUAGE ______ 4<br>(SPECIFY LANGUAGE)<br>BLIND/VISUALLY IMPAIRED . . . 5	

SECTION 1. RESPONDENT'S BACKGROUND

NO.	QUESTIONS AND FILTERS	CODING CATEGORIES	SKIP
112	CHECK 111:<br>CODE '2', '3' OR '4' CIRCLED ☐ ↓ CODE '1' OR '5' CIRCLED ☐ →		→ 114
113	Do you read a newspaper or magazine at least once a week, less than once a week or not at all?	AT LEAST ONCE A WEEK . . . . 1<br>LESS THAN ONCE A WEEK . . . . 2<br>NOT AT ALL 3	
114	Do you listen to the radio at least once a week, less than once a week or not at all?	AT LEAST ONCE A WEEK . . . . 1<br>LESS THAN ONCE A WEEK . . . . 2<br>NOT AT ALL 3	
115	Do you watch television at least once a week, less than once a week or not at all?	AT LEAST ONCE A WEEK . . . . 1<br>LESS THAN ONCE A WEEK . . . . 2<br>NOT AT ALL 3	
116	Do you own a mobile telephone?	YES . . . . 1<br>NO 2	 → 118
117	Do you use your mobile phone for any financial transactions?	YES . . . . 1<br>NO 2	
118	Do you have an account in a bank or other financial institution that you yourself use?	YES . . . . 1<br>NO 2	
119	Have you ever used the internet?	YES . . . . 1<br>NO 2	 → 122
120	In the last 12 months, have you used the internet?<br><br>IF NECESSARY, PROBE FOR USE FROM ANY LOCATION, WITH ANY DEVICE.	YES . . . . 1<br>NO 2	 → 122
121	During the last one month, how often did you use the internet: almost every day, at least once a week, less than once a week, or not at all?	ALMOST EVERY DAY . . . . 1<br>AT LEAST ONCE A WEEK . . . . 2<br>LESS THAN ONCE A WEEK . . . . 3<br>NOT AT ALL 4	
122	What is your religion?	CATHOLIC . . . . 1<br>OTHER CHRISTIAN . . . . 2<br>ISLAM . . . . 3<br>TRADITIONALIST . . . . 4<br><br>OTHER ______ 6<br>(SPECIFY)	
123	What is your ethnic group?	______ ☐☐☐<br>(ETHNIC GROUP)	
124	In the last 12 months, how many times have you been away from home for one or more nights?	NUMBER OF TIMES . . . . ☐☐<br><br>NONE 00	 <br>→ 201
125	In the last 12 months, have you been away from home for more than one month at a time?	YES . . . . 1<br>NO 2	

SECTION 2. REPRODUCTION

NO.	QUESTIONS AND FILTERS	CODING CATEGORIES	SKIP
201	Now I would like to ask about all the births you have had during your life. Have you ever given birth?	YES . . . . . 1<br>NO 2	 →206
202	Do you have any sons or daughters to whom you have given birth who are now living with you?	YES . . . . . 1<br>NO 2	 →204
203	a) How many sons live with you?<br>b) And how many daughters live with you?<br>IF NONE, RECORD '00'.	a) SONS AT HOME . . . . . ☐☐<br>b) DAUGHTERS AT HOME . . . . . ☐☐	
204	Do you have any sons or daughters to whom you have given birth who are alive but do not live with you?	YES . . . . . 1<br>NO 2	 →206
205	a) How many sons are alive but do not live with you?<br>b) And how many daughters are alive but do not live with you?<br>IF NONE, RECORD '00'.	a) SONS ELSEWHERE . . . . . ☐☐<br>b) DAUGHTERS ELSEWHERE . . . . . ☐☐	
206	Have you ever given birth to a boy or girl who was born alive but later died?<br>IF NO, PROBE: Any baby who cried, who made any movement, sound, or effort to breathe, or who showed any other signs of life even if for a very short time?	YES . . . . . 1<br>NO 2	 →208
207	a) How many boys have died?<br>b) And how many girls have died?<br>IF NONE, RECORD '00'.	a) BOYS DEAD . . . . . ☐☐<br>b) GIRLS DEAD . . . . . ☐☐	
208	SUM ANSWERS TO 203, 205, AND 207, AND ENTER TOTAL. IF NONE, RECORD '00'.	TOTAL BIRTHS . . . . . ☐☐	
209	CHECK 208:<br>Just to make sure that I have this right: you have had in TOTAL _____ births during your life. Is that correct?<br>YES ☐ ↓ NO ☐ → PROBE AND CORRECT 201-208 AS NECESSARY.		
210	CHECK 208:<br>ONE OR MORE BIRTHS ☐ ↓ NO BIRTHS ☐		→226

211 Now I would like to record the names of all your births, whether still alive or not, starting with the first one you had.
RECORD NAMES OF ALL THE BIRTHS IN 212. RECORD TWINS AND TRIPLETS ON SEPARATE ROWS. IF THERE ARE MORE THAN 10 BIRTHS, USE AN ADDITIONAL QUESTIONNAIRE, STARTING WITH THE SECOND ROW.

212<br><br>What name was given to your (first/ next) baby?<br><br>RECORD NAME.<br><br>BIRTH HISTORY NUMBER.	213<br><br>Is (NAME) a boy or a girl?	214<br><br>Were any of these births twins?	215<br><br>On what day, month, and year was (NAME) born?	216<br><br>Is (NAME) still alive?	217<br>IF ALIVE:<br><br>How old was (NAME) at (NAME)'s last birthday?<br><br>RECORD AGE IN COMP-LETED YEARS.	218<br>IF ALIVE:<br><br>Is (NAME) living with you?	219<br>IF ALIVE:<br><br>RECORD HOUSEHOLD LINE NUMBER OF CHILD. RECORD '00' IF CHILD NOT LISTED IN HOUSEHOLD.	220<br>IF DEAD:<br><br>How old was (NAME) when (he/she) died?<br><br>IF '12 MONTHS' OR '1 YR', ASK: Did (NAME) have (his/her) first birthday?<br><br>THEN ASK: Exactly how many months old was (NAME) when (he/she) died?<br><br>RECORD DAYS IF LESS THAN 1 MONTH; MONTHS IF LESS THAN TWO YEARS; OR YEARS.	220B<br>**IF DEATH AT AGE 0-5**<br><br>On what day, month and year did (NAME) die?	221<br><br>Were there any other live births between (NAME OF PREVIOUS BIRTH) and (NAME), including any children who died after birth?
01	BOY 1<br>GIRL 2	SING 1<br>MULT 2	DAY<br>MONTH<br>YEAR	YES 1<br>NO 2<br>(SKIP TO 220)	AGE IN YEARS	YES 1<br>NO 2	HOUSEHOLD LINE NUMBER<br>(NEXT BIRTH)	DAYS 1<br>MONTHS 2<br>YEARS 3	DAY<br>MONTH<br>YEAR	
02	BOY 1<br>GIRL 2	SING 1<br>MULT 2	DAY<br>MONTH<br>YEAR	YES 1<br>NO 2<br>(SKIP TO 220)	AGE IN YEARS	YES 1<br>NO 2	HOUSEHOLD LINE NUMBER<br>(SKIP TO 221)	DAYS 1<br>MONTHS 2<br>YEARS 3	DAY<br>MONTH<br>YEAR	YES 1<br>(ADD BIRTH)<br>NO 2<br>(NEXT BIRTH)
03	BOY 1<br>GIRL 2	SING 1<br>MULT 2	DAY<br>MONTH<br>YEAR	YES 1<br>NO 2<br>(SKIP TO 220)	AGE IN YEARS	YES 1<br>NO 2	HOUSEHOLD LINE NUMBER<br>(SKIP TO 221)	DAYS 1<br>MONTHS 2<br>YEARS 3	DAY<br>MONTH<br>YEAR	YES 1<br>(ADD BIRTH)<br>NO 2<br>(NEXT BIRTH)
04	BOY 1<br>GIRL 2	SING 1<br>MULT 2	DAY<br>MONTH<br>YEAR	YES 1<br>NO 2<br>(SKIP TO 220)	AGE IN YEARS	YES 1<br>NO 2	HOUSEHOLD LINE NUMBER<br>(SKIP TO 221)	DAYS 1<br>MONTHS 2<br>YEARS 3	DAY<br>MONTH<br>YEAR	YES 1<br>(ADD BIRTH)<br>NO 2<br>(NEXT BIRTH)
05	BOY 1<br>GIRL 2	SING 1<br>MULT 2	DAY<br>MONTH<br>YEAR	YES 1<br>NO 2<br>(SKIP TO 220)	AGE IN YEARS	YES 1<br>NO 2	HOUSEHOLD LINE NUMBER<br>(SKIP TO 221)	DAYS 1<br>MONTHS 2<br>YEARS 3	DAY<br>MONTH<br>YEAR	YES 1<br>(ADD BIRTH)<br>NO 2<br>(NEXT BIRTH)

212	213	214	215	216	217 IF ALIVE:	218 IF ALIVE:	219 IF ALIVE:	220 IF DEAD:	220B **IF DEATH AT AGE 0-5**	221
What name was given to your (first/ next) baby?<br><br>RECORD NAME.<br><br>BIRTH HISTORY NUMBER.	Is (NAME) a boy or a girl?	Were any of these births twins?	On what day, month, and year was (NAME) born?	Is (NAME) still alive?	How old was (NAME) at (NAME)'s last birthday?<br><br>RECORD AGE IN COMP-LETED YEARS.	Is (NAME) living with you?	RECORD HOUSEHOLD LINE NUMBER OF CHILD. RECORD '00' IF CHILD NOT LISTED IN HOUSEHOLD.	How old was (NAME) when (he/she) died?<br><br>IF '12 MONTHS' OR '1 YR', ASK: Did (NAME) have (his/her) first birthday?<br><br>THEN ASK: Exactly how many months old was (NAME) when (he/she) died?<br><br>RECORD DAYS IF LESS THAN 1 MONTH; MONTHS IF LESS THAN TWO YEARS; OR YEARS.	On what day, month and year did (NAME) die?	Were there any other live births between (NAME OF PREVIOUS BIRTH) and (NAME), including any children who died after birth?
06	BOY 1<br>GIRL 2	SING 1<br>MULT 2	DAY<br>MONTH<br>YEAR	YES 1<br>NO 2<br>(SKIP TO 220)	AGE IN YEARS	YES 1<br>NO 2	HOUSEHOLD LINE NUMBER<br>(SKIP TO 221)	DAYS 1<br>MONTHS 2<br>YEARS 3	DAY<br>MONTH<br>YEAR	YES 1 (ADD BIRTH)<br>NO 2 (NEXT BIRTH)
07	BOY 1<br>GIRL 2	SING 1<br>MULT 2	DAY<br>MONTH<br>YEAR	YES 1<br>NO 2<br>(SKIP TO 220)	AGE IN YEARS	YES 1<br>NO 2	HOUSEHOLD LINE NUMBER<br>(SKIP TO 221)	DAYS 1<br>MONTHS 2<br>YEARS 3	DAY<br>MONTH<br>YEAR	YES 1 (ADD BIRTH)<br>NO 2 (NEXT BIRTH)
08	BOY 1<br>GIRL 2	SING 1<br>MULT 2	DAY<br>MONTH<br>YEAR	YES 1<br>NO 2<br>(SKIP TO 220)	AGE IN YEARS	YES 1<br>NO 2	HOUSEHOLD LINE NUMBER<br>(SKIP TO 221)	DAYS 1<br>MONTHS 2<br>YEARS 3	DAY<br>MONTH<br>YEAR	YES 1 (ADD BIRTH)<br>NO 2 (NEXT BIRTH)
09	BOY 1<br>GIRL 2	SING 1<br>MULT 2	DAY<br>MONTH<br>YEAR	YES 1<br>NO 2<br>(SKIP TO 220)	AGE IN YEARS	YES 1<br>NO 2	HOUSEHOLD LINE NUMBER<br>(SKIP TO 221)	DAYS 1<br>MONTHS 2<br>YEARS 3	DAY<br>MONTH<br>YEAR	YES 1 (ADD BIRTH)<br>NO 2 (NEXT BIRTH)
10	BOY 1<br>GIRL 2	SING 1<br>MULT 2	DAY<br>MONTH<br>YEAR	YES 1<br>NO 2<br>(SKIP TO 220)	AGE IN YEARS	YES 1<br>NO 2	HOUSEHOLD LINE NUMBER<br>(SKIP TO 221)	DAYS 1<br>MONTHS 2<br>YEARS 3	DAY<br>MONTH<br>YEAR	YES 1 (ADD BIRTH)<br>NO 2 (NEXT BIRTH)

SECTION 2. REPRODUCTION

NO.	QUESTIONS AND FILTERS	CODING CATEGORIES	SKIP
222	Have you had any live births since the birth of (NAME OF LAST BIRTH)?	YES . . . . 1 (RECORD BIRTH(S) IN TABLE)<br>NO 2	
223	COMPARE 208 WITH NUMBER OF BIRTHS IN BIRTH HISTORY<br>NUMBERS ARE SAME ☐<br>NUMBERS ARE DIFFERENT ☐ → (PROBE AND RECONCILE)		
223A	CHECK 220B: ENTER THE NUMBER OF DEATHS IN JANUARY 2014 OR LATER<br>IF NONE, RECORD `0'.	NUMBER OF DEATHS . . . . ☐	
224	CHECK 215: ENTER THE NUMBER OF BIRTHS IN 2013-2018	NUMBER OF BIRTHS . . . . ☐<br>NONE 0	→ 226
225	**C** FOR EACH BIRTH IN 2013-2018, ENTER 'B' IN THE MONTH OF BIRTH IN THE CALENDAR. WRITE THE NAME OF THE CHILD TO THE LEFT OF THE 'B' CODE. FOR EACH BIRTH, ASK THE NUMBER OF COMPLETED MONTHS THE PREGNANCY LASTED AND RECORD 'P' IN EACH OF THE PRECEDING MONTHS ACCORDING TO THE DURATION OF PREGNANCY. (NOTE: THE NUMBER OF 'P's MUST BE ONE LESS THAN THE NUMBER OF MONTHS THAT THE PREGNANCY LASTED.)		
226	Are you pregnant now?	YES . . . . 1<br>NO . . . . 2<br>UNSURE 8	 NO, UNSURE → 230
227	How many months pregnant are you?<br>RECORD NUMBER OF COMPLETED MONTHS.<br>**C** ENTER 'P's IN THE CALENDAR, BEGINNING WITH THE MONTH OF INTERVIEW AND FOR THE TOTAL NUMBER OF COMPLETED MONTHS.	MONTHS . . . . ☐☐	
228	When you got pregnant, did you want to get pregnant at that time?	YES . . . . 1<br>NO 2	YES → 230
229	CHECK 208: TOTAL NUMBER OF BIRTHS<br>ONE OR MORE ☐ → a) Did you want to have a baby later on or did you not want any more children?<br>NONE ☐ → b) Did you want to have a baby later on or did you not want any children?	LATER . . . . 1<br>NO MORE/NONE 2	
230	Have you ever had a pregnancy that miscarried, was aborted, or ended in a stillbirth?	YES . . . . 1<br>NO 2	 NO → 239
231	When did the last such pregnancy end?	MONTH . . . . ☐☐<br>YEAR . . . . ☐☐☐☐	

NO.	QUESTIONS AND FILTERS	CODING CATEGORIES	SKIP
232	CHECK 231: LAST PREGNANCY ENDED IN 2013-2018 ☐		→ 234
	LAST PREGNANCY ENDED IN 2012 OR EARLIER ☐		→ 239

LINE NO.	233 In what month and year did the preceding such pregnancy end?	234 How many months pregnant were you when that pregnancy ended?	235 Since January 2013, have you had any other pregnancies that did not result in a live birth?	
01		NUMBER OF MONTHS	YES . . . . 1<br>NO 2	→ NEXT LINE<br>→ 236
02	MONTH YEAR	NUMBER OF MONTHS	YES . . . . 1<br>NO 2	→ NEXT LINE<br>→ 236
03	MONTH YEAR	NUMBER OF MONTHS	YES . . . . 1<br>NO 2	→ NEXT LINE<br>→ 236
04	MONTH YEAR	NUMBER OF MONTHS	YES . . . . 1<br>NO 2	→ 236

NO.	QUESTIONS AND FILTERS	CODING CATEGORIES	SKIP
236	**C** FOR EACH PREGNANCY THAT DID NOT END IN A LIVE BIRTH IN 2013-2018 OR LATER, ENTER 'T' IN THE CALENDAR IN THE MONTH THAT THE PREGNANCY TERMINATED AND 'P' FOR THE REMAINING NUMBER OF COMPLETED MONTHS OF PREGNANCY.<br><br>IF THERE ARE MORE THAN FOUR PREGNANCIES THAT DID NOT END IN A LIVE BIRTH, USE AN ADDITIONAL QUESTIONNAIRE STARTING ON THE SECOND LINE.		
237	Did you have any miscarriages, abortions or stillbirths that ended before 2013?	YES . . . . 1<br>NO 2	 → 239
238	When did the last such pregnancy that terminated before 2013 end?	MONTH . . . .<br>YEAR	

NO.	QUESTIONS AND FILTERS	CODING CATEGORIES	SKIP
239	When did your last menstrual period start?<br><br>______________________<br>(DATE, IF GIVEN)	DAYS AGO . . . . 1 [ ][ ]<br>WEEKS AGO . . . . 2 [ ][ ]<br>MONTHS AGO . . . . 3 [ ][ ]<br>YEARS AGO . . . . 4 [ ][ ]<br><br>IN MENOPAUSE/<br>HAS HAD HYSTERECTOMY . . . . 994<br><br>BEFORE LAST BIRTH . . . . 995<br><br>NEVER MENSTRUATED 996	
240	From one menstrual period to the next, are there certain days when a woman is more likely to become pregnant?	YES . . . . 1<br>NO . . . . 2<br>DON'T KNOW 8	NO, DON'T KNOW → 242
241	Is this time just before her period begins, during her period, right after her period has ended, or halfway between two periods?	JUST BEFORE HER PERIOD BEGINS . . . . 1<br>DURING HER PERIOD . . . . 2<br>RIGHT AFTER HER PERIOD HAS ENDED . . . . 3<br>HALFWAY BETWEEN TWO PERIODS . . . . 4<br><br>OTHER ______________________ 6<br>(SPECIFY)<br>DON'T KNOW 8	
242	After the birth of a child, can a woman become pregnant before her menstrual period has returned?	YES . . . . 1<br>NO . . . . 2<br>DON'T KNOW 8	

SECTION 3. CONTRACEPTION

301	Now I would like to talk about family planning - the various ways or methods that a couple can use to delay or avoid a pregnancy. Have you ever heard of (METHOD)?	
01	Female Sterilization.<br>PROBE: Women can have an operation to avoid having any more children.	YES . . . . . 1<br>NO 2
02	Male Sterilization.<br>PROBE: Men can have an operation to avoid having any more children.	YES . . . . . 1<br>NO 2
03	IUD.<br>PROBE: Women can have a loop or coil placed inside them by a doctor or a nurse which can prevent pregnancy for one or more years.	YES . . . . . 1<br>NO 2
04	Injectables.<br>PROBE: Women can have an injection by a health provider that stops them from becoming pregnant for one or more months.	YES . . . . . 1<br>NO 2
05	Implants.<br>PROBE: Women can have one or more small rods placed in their upper arm by a doctor or nurse which can prevent pregnancy for one or more years.	YES . . . . . 1<br>NO 2
06	Pill.<br>PROBE: Women can take a pill every day to avoid becoming pregnant.	YES . . . . . 1<br>NO 2
07	Condom.<br>PROBE: Men can put a rubber sheath on their penis before sexual intercourse.	YES . . . . . 1<br>NO 2
08	Female Condom.<br>PROBE: Women can place a sheath in their vagina before sexual intercourse.	YES . . . . . 1<br>NO 2
09	Emergency Contraception.<br>PROBE: As an emergency measure, within three days after they have unprotected sexual intercourse, women can take special pills to prevent pregnancy.	YES . . . . . 1<br>NO 2
10	Standard Days Method.<br>PROBE: A woman uses a string of colored beads to know the days she can get pregnant. On the days she can get pregnant, she uses a condom or does not have sexual intercourse.	YES . . . . . 1<br>NO 2
11	Lactational Amenorrhea Method (LAM).<br>PROBE: Up to six months after childbirth, before the menstrual period has returned, women use a method requiring frequent breastfeeding day and night.	YES . . . . . 1<br>NO 2
12	Rhythm Method.<br>PROBE: To avoid pregnancy, women do not have sexual intercourse on the days of the month they think they can get pregnant.	YES . . . . . 1<br>NO 2
13	Withdrawal.<br>PROBE: Men can be careful and pull out before climax.	YES . . . . . 1<br>NO 2
14	Have you heard of any other ways or methods that women or men can use to avoid pregnancy?	YES, MODERN METHOD<br>________________ A<br>(SPECIFY)<br>YES, TRADITIONAL METHOD<br>________________ B<br>(SPECIFY)<br>NO . . . . . Y

SECTION 3. CONTRACEPTION

NO.	QUESTIONS AND FILTERS	CODING CATEGORIES	SKIP
302	CHECK 226:<br>NOT PREGNANT OR UNSURE ☐ ↓	PREGNANT ☐	→ 312
303	Are you or your partner currently doing something or using any method to delay or avoid getting pregnant?	YES . . . . 1<br>NO 2	 → 312
304	Which method are you using?<br><br>RECORD ALL MENTIONED.<br><br>IF MORE THAN ONE METHOD MENTIONED, FOLLOW SKIP INSTRUCTION FOR HIGHEST METHOD IN LIST.	FEMALE STERILIZATION . . . . A<br>MALE STERILIZATION . . . . B<br>IUD . . . . C<br>INJECTABLES . . . . D<br>IMPLANTS . . . . E<br>PILL . . . . F<br>CONDOM . . . . G<br>FEMALE CONDOM . . . . H<br>EMERGENCY CONTRACEPTION . . . . I<br>STANDARD DAYS METHOD . . . . J<br>LACTATIONAL AMENORRHEA METHOD . . . . K<br>RHYTHM METHOD . . . . L<br>WITHDRAWAL . . . . M<br>OTHER MODERN METHOD . . . . X<br>OTHER TRADITIONAL METHOD . . . . Y	A–B → 307<br>C–E → 309<br>G → 306<br>H–Y → 309
305	What is the brand name of the pills you are using?<br><br>IF DON'T KNOW THE BRAND, ASK TO SEE THE PACKAGE.	DUOFEMCONFIDENCE . . . . 01<br>MICROGYNON . . . . 02<br>LOFEMENAL . . . . 03<br>NEOGYNON . . . . 04<br>OTHER ______ 96<br>(SPECIFY)<br>DON'T KNOW 98	→ 309
306	What is the brand name of the condoms you are using?<br><br>IF DON'T KNOW THE BRAND, ASK TO SEE THE PACKAGE.	MALE CONDOMS<br>GOLD CIRCLE . . . . 01<br>DUREX . . . . 02<br>ROUGH RIDER . . . . 03<br>TWIN LOTUS . . . . 04<br>PLAIN CONDOMS . . . . 05<br>GO FLEX . . . . 06<br>OTHER ______ 96<br>(SPECIFY)<br>DON'T KNOW 98	→ 309

NO.	QUESTIONS AND FILTERS	CODING CATEGORIES	SKIP
307	In what facility did the sterilization take place?<br><br>PROBE TO IDENTIFY THE TYPE OF SOURCE.<br><br>IF UNABLE TO DETERMINE IF PUBLIC OR PRIVATE SECTOR, WRITE THE NAME OF THE PLACE.<br><br>_______________<br>(NAME OF PLACE)	**PUBLIC SECTOR**<br>GOVERNMENT HOSPITAL . . . . 11<br>GOVERNMENT HEALTH CENTER . . . . 12<br>FAMILY PLANNING CLINIC . . . . 13<br>MOBILE CLINIC . . . . 14<br>OTHER PUBLIC SECTOR<br>_______________ 16<br>(SPECIFY)<br><br>**PRIVATE MEDICAL SECTOR**<br>PRIVATE HOSPITAL/CLINIC . . . . 21<br>PRIVATE DOCTOR'S OFFICE . . . . 22<br>MOBILE CLINIC . . . . 23<br>NON-GOVERNMENT ORGANIZATION . . . . 24<br><br>OTHER PRIVATE MEDICAL SECTOR<br>_______________ 26<br>(SPECIFY)<br><br>OTHER _______________ 96<br>(SPECIFY)<br>DON'T KNOW 98	
308	In what month and year was the sterilization performed?	MONTH . . . . [ ][ ]<br>YEAR . . . . [ ][ ][ ][ ]	→ 310
309	Since what month and year have you been using (CURRENT METHOD) without stopping?<br><br>PROBE: For how long have you been using (CURRENT METHOD) now without stopping?	MONTH . . . . [ ][ ]<br>YEAR . . . . [ ][ ][ ][ ]	
310	CHECK 308 AND 309, 215 AND 231: ANY BIRTH OR PREGNANCY TERMINATION AFTER MONTH AND YEAR OF START OF USE OF CONTRACEPTION IN 308 OR 309<br><br>NO [ ] ↓ YES [ ] → GO BACK TO 308 OR 309, PROBE AND RECORD MONTH AND YEAR AT START OF CONTINUOUS USE OF CURRENT METHOD (MUST BE AFTER LAST BIRTH OR PREGNANCY TERMINATION).		

311	CHECK 308 AND 309:<br>YEAR IS 2013-2018: C ENTER CODE FOR METHOD USED IN MONTH OF INTERVIEW IN THE CALENDAR AND IN EACH MONTH BACK TO THE DATE STARTED USING. THEN CONTINUE<br>YEAR IS 2012 OR EARLIER: C ENTER CODE FOR METHOD USED IN MONTH OF INTERVIEW IN THE CALENDAR AND EACH MONTH BACK TO JANUARY 2013 . THEN (SKIP TO 324)
312	I would like to ask you some questions about the times you or your partner may have used a method to avoid getting pregnant during the last few years.<br>C USE CALENDAR TO PROBE FOR EARLIER PERIODS OF USE AND NONUSE, STARTING WITH MOST RECENT USE, BACK TO JANUARY 2013. USE NAMES OF CHILDREN, DATES OF BIRTH, AND PERIODS OF PREGNANCY AS REFERENCE POINTS.

		COLUMN 1	COLUMN 2	COLUMN 3
312A	MONTH AND YEAR OF START OF INTERVAL OF USE OR NON-USE.	MONTH [ ][ ]<br>[ ][ ][ ][ ] YEAR	MONTH [ ][ ]<br>[ ][ ][ ][ ] YEAR	MONTH [ ][ ]<br>[ ][ ][ ][ ] YEAR
312B	Between (EVENT) in (MONTH/YEAR) and (EVENT) in (MONTH/YEAR), did you or your partner use any method of contraception?	YES . . . . 1<br>NO . . . . 2 (SKIP TO 312I)	YES . . . . 1<br>NO . . . . 2 (SKIP TO 312I)	YES . . . . 1<br>NO . . . . 2 (SKIP TO 312I)
312C	Which method was that?	METHOD CODE . . [ ]	METHOD CODE . . [ ]	METHOD CODE . . [ ]
312D	How many months after (EVENT) in (MONTH/YEAR) did you start to use (METHOD)?<br>CIRCLE '95' IF RESPONDENT GIVES THE DATE OF STARTING TO USE THE METHOD.	IMMEDIATELY . . . . . 00<br>MONTHS . . [ ][ ]<br>(SKIP TO 312F)<br>DATE GIVEN 95	IMMEDIATELY . . . . . 00<br>MONTHS . . [ ][ ]<br>(SKIP TO 312F)<br>DATE GIVEN 95	IMMEDIATELY . . . . . 00<br>MONTHS . . [ ][ ]<br>(SKIP TO 312F)<br>DATE GIVEN 95
312E	RECORD MONTH AND YEAR RESPONDENT STARTED USING METHOD.	MONTH [ ][ ]<br>[ ][ ][ ][ ] YEAR	MONTH [ ][ ]<br>[ ][ ][ ][ ] YEAR	MONTH [ ][ ]<br>[ ][ ][ ][ ] YEAR
312F	For how many months did you use (METHOD)?<br>CIRCLE '95' IF RESPONDENT GIVES THE DATE OF TERMINATION OF USE.	MONTHS . . [ ][ ]<br>(SKIP TO 312H)<br>DATE GIVEN 95	MONTHS . . [ ][ ]<br>(SKIP TO 312H)<br>DATE GIVEN 95	MONTHS . . [ ][ ]<br>(SKIP TO 312H)<br>DATE GIVEN 95
312G	RECORD MONTH AND YEAR RESPONDENT STOPPED USING METHOD.	MONTH [ ][ ]<br>[ ][ ][ ][ ] YEAR	MONTH [ ][ ]<br>[ ][ ][ ][ ] YEAR	MONTH [ ][ ]<br>[ ][ ][ ][ ] YEAR
312H	Why did you stop using (METHOD)?	REASON STOPPED . . . . . [ ]	REASON STOPPED . . . . . [ ]	REASON STOPPED . . . . . [ ]
312I		GO BACK TO 312A IN NEXT COLUMN; OR, IF NO MORE GAPS, GO TO 313.	GO BACK TO 312A IN NEXT COLUMN; OR, IF NO MORE GAPS, GO TO 313.	GO BACK TO 312A IN NEW QUESTIONNAIRE; OR, IF NO MORE GAPS, GO TO 313.

SECTION 3. CONTRACEPTION

NO.	QUESTIONS AND FILTERS	CODING CATEGORIES	SKIP
313	CHECK THE CALENDAR FOR USE OF ANY CONTRACEPTIVE METHOD IN ANY MONTH<br>NO METHOD USED ☐ (continue) / ANY METHOD USED ☐ → 315		ANY METHOD USED → 315
314	Have you ever used anything or tried in any way to delay or avoid getting pregnant?	YES . . . 1<br>NO . . . 2	1, 2 → 326
315	CHECK 304:<br><br>CIRCLE METHOD CODE:<br><br>IF MORE THAN ONE METHOD CODE CIRCLED IN 304, CIRCLE CODE FOR HIGHEST METHOD IN LIST.	NO CODE CIRCLED . . . 00<br>FEMALE STERILIZATION . . . 01<br>MALE STERILIZATION . . . 02<br>IUD . . . 03<br>INJECTABLES . . . 04<br>IMPLANTS . . . 05<br>PILL . . . 06<br>CONDOM . . . 07<br>FEMALE CONDOM . . . 08<br>EMERGENCY CONTRACEPTION . . . 09<br>STANDARD DAYS METHOD . . . 10<br>LACTATIONAL AMENORRHEA METHOD . . . 11<br>RHYTHM METHOD . . . 12<br>WITHDRAWAL . . . 13<br>OTHER MODERN METHOD . . . 95<br>OTHER TRADITIONAL METHOD . . . 96	00 → 326<br>01 → 319<br>02 → 327<br>11, 12, 13 → 323
316	You first started using (CURRENT METHOD) in (DATE FROM 309). Where did you get it at that time?<br><br>PROBE TO IDENTIFY THE TYPE OF SOURCE.<br><br>IF UNABLE TO DETERMINE IF PUBLIC OR PRIVATE SECTOR, WRITE THE NAME OF THE PLACE.<br><br>__________<br>(NAME OF PLACE)	**PUBLIC SECTOR**<br>GOVERNMENT HOSPITAL . . . 11<br>GOVERNMENT HEALTH CENTER . . . 12<br>FAMILY PLANNING CLINIC . . . 13<br>MOBILE CLINIC . . . 14<br>FIELDWORKER . . . 15<br>OTHER PUBLIC SECTOR<br>__________ 16<br>(SPECIFY)<br><br>**PRIVATE MEDICAL SECTOR**<br>PRIVATE HOSPITAL/CLINIC . . . 21<br>PHARMACY . . . 22<br>CHEMIST/PMS STORE . . . 23<br>PRIVATE DOCTOR . . . 24<br>MOBILE CLINIC . . . 25<br>FIELDWORKER . . . 26<br>OTHER PRIVATE MEDICAL SECTOR<br>__________ 27<br>(SPECIFY)<br><br>**OTHER SOURCE**<br>SHOP . . . 31<br>CHURCH . . . 32<br>FRIEND/RELATIVE . . . 33<br>NGO . . . 34<br><br>OTHER __________ 96<br>(SPECIFY)	
317	CHECK 304:<br><br>CIRCLE METHOD CODE:<br><br>IF MORE THAN ONE METHOD CODE CIRCLED IN 304, CIRCLE CODE FOR HIGHEST METHOD IN LIST.	IUD . . . 03<br>INJECTABLES . . . 04<br>IMPLANTS . . . 05<br>PILL . . . 06<br>CONDOM . . . 07<br>FEMALE CONDOM . . . 08<br>EMERGENCY CONTRACEPTION . . . 09<br>STANDARD DAYS METHOD . . . 10<br>OTHER MODERN METHOD . . . 95<br>OTHER TRADITIONAL METHOD . . . 96	07 → 323<br>08, 09, 10, 95 → 322<br>96 → 323

NO.	QUESTIONS AND FILTERS	CODING CATEGORIES	SKIP
318	At that time, were you told about side effects or problems you might have with the method?	YES . . . . 1<br>NO 2	→ 321<br>→ 320
319	When you got sterilized, were you told about side effects or problems you might have with the method?	YES . . . . 1<br>NO 2	→ 321
320	Were you ever told by a health or family planning worker about side effects or problems you might have with the method?	YES . . . . 1<br>NO 2	 → 322
321	Were you told what to do if you experienced side effects or problems?	YES . . . . 1<br>NO 2	
322	CHECK 318 AND 319:<br>ANY 'YES' ☐ ↓ | OTHER ☐ ↓<br>a) At that time, were you told about other methods of family planning that you could use?<br>b) When you obtained (CURRENT METHOD FROM 315) from (SOURCE OF METHOD FROM 307 OR 316), were you told about other methods of family planning that you could use?	YES . . . . 1<br>NO 2	→ 324
323	Were you ever told by a health or family planning worker about other methods of family planning that you could use?	YES . . . . 1<br>NO 2	
324	CHECK 304:<br><br>CIRCLE METHOD CODE:<br><br>IF MORE THAN ONE METHOD CODE CIRCLED IN 304, CIRCLE CODE FOR HIGHEST METHOD IN LIST.	FEMALE STERILIZATION . . . . 01<br>MALE STERILIZATION . . . . 02<br>IUD . . . . 03<br>INJECTABLES . . . . 04<br>IMPLANTS . . . . 05<br>PILL . . . . 06<br>CONDOM . . . . 07<br>FEMALE CONDOM . . . . 08<br>EMERGENCY CONTRACEPTION . . . . 09<br>STANDARD DAYS METHOD . . . . 10<br>LACTATIONAL AMENORRHEA METHOD . . . . 11<br>RHYTHM METHOD . . . . 12<br>WITHDRAWAL . . . . 13<br>OTHER MODERN METHOD . . . . 95<br>OTHER TRADITIONAL METHOD 96	01–02 → 327<br><br><br><br><br><br><br><br><br><br>11–13 → 327<br><br><br><br>96 → 327

NO.	QUESTIONS AND FILTERS	CODING CATEGORIES	SKIP
325	Where did you obtain (CURRENT METHOD) the last time?<br><br>PROBE TO IDENTIFY THE TYPE OF SOURCE.<br><br>IF UNABLE TO DETERMINE IF PUBLIC OR PRIVATE SECTOR, WRITE THE NAME OF THE PLACE.<br><br>__________<br>(NAME OF PLACE)	**PUBLIC SECTOR**<br>GOVERNMENT HOSPITAL . . . . . 11<br>GOVERNMENT HEALTH CENTER . . . . . 12<br>FAMILY PLANNING CLINIC . . . . . 13<br>MOBILE CLINIC . . . . . 14<br>FIELDWORKER . . . . . 15<br>OTHER PUBLIC SECTOR<br>__________ 16<br>(SPECIFY)<br><br>**PRIVATE MEDICAL SECTOR**<br>PRIVATE HOSPITAL/CLINIC . . . . . 21<br>PHARMACY . . . . . 22<br>CHEMIST/PMS STORE . . . . . 23<br>PRIVATE DOCTOR . . . . . 24<br>MOBILE CLINIC . . . . . 25<br>FIELDWORKER . . . . . 26<br>OTHER PRIVATE MEDICAL SECTOR<br>__________ 27<br>(SPECIFY)<br><br>**OTHER SOURCE**<br>SHOP . . . . . 31<br>CHURCH . . . . . 32<br>FRIEND/RELATIVE . . . . . 33<br>NGO . . . . . 34<br><br>OTHER __________ 96<br>(SPECIFY)	→ 327
326	Do you know of a place where you can obtain a method of family planning?	YES . . . . . 1<br>NO 2	
327	In the last 12 months, were you visited by a fieldworker?	YES . . . . . 1<br>NO 2	 NO → 329
328	Did the fieldworker talk to you about family planning?	YES . . . . . 1<br>NO 2	
329	CHECK 202: CHILDREN LIVING WITH RESPONDENT<br>YES ☐ ↓ / NO ☐ ↓<br>a) In the last 12 months, have you visited a health facility for care for yourself or your children?<br>b) In the last 12 months, have you visited a health facility for care for yourself?	YES . . . . . 1<br>NO 2	 NO → 401
330	Did any staff member at the health facility speak to you about family planning methods?	YES . . . . . 1<br>NO 2	

No.	Question	Last birth	Next-to-last birth
401	CHECK 224:<br>ONE OR MORE BIRTHS IN 2013-2018 [ ] ↓<br>NO BIRTHS IN 2013-2018 [ ] → 648		
402	CHECK 215. RECORD THE BIRTH HISTORY NUMBER IN 403 AND THE NAME AND SURVIVAL STATUS IN 404 FOR EACH BIRTH IN 2013-2018. ASK THE QUESTIONS ABOUT ALL OF THESE BIRTHS. BEGIN WITH THE LAST BIRTH. IF THERE ARE MORE THAN 2 BIRTHS, USE LAST COLUMN OF ADDITIONAL QUESTIONNAIRE(S).<br>Now I would like to ask some questions about your children born in the last five years. (We will talk about each separately.)		
403	BIRTH HISTORY NUMBER FROM 212 IN BIRTH HISTORY.	LAST BIRTH<br>BIRTH HISTORY NUMBER . . . . [ ][ ]	NEXT-TO-LAST BIRTH<br>BIRTH HISTORY NUMBER . . . . [ ][ ]
404	FROM 212 AND 216:	NAME ______<br>LIVING [ ] ↓ DEAD [ ] ↓	NAME ______<br>LIVING [ ] ↓ DEAD [ ] ↓
405	When you got pregnant with (NAME), did you want to get pregnant at that time?	YES . . . . 1 (SKIP TO 408)<br>NO 2	YES . . . . 1 (SKIP TO 426)<br>NO 2
406	CHECK 208:<br>ONLY ONE BIRTH [ ] ↓<br>a) Did you want to have a baby later on, or did you not want any children?<br>MORE THAN ONE BIRTH [ ] ↓<br>b) Did you want to have a baby later on, or did you not want any more children?	LATER . . . . 1<br>NO MORE/NONE . . . . 2 (SKIP TO 408)	LATER . . . . 1<br>NO MORE/NONE . . . . 2 (SKIP TO 426)
407	How much longer did you want to wait?	MONTHS . . . . 1 [ ][ ]<br>YEARS . . . . 2 [ ][ ]<br>DON'T KNOW 998	MONTHS . . . . 1 [ ][ ]<br>YEARS . . . . 2 [ ][ ]<br>DON'T KNOW 998
408	Did you see anyone for antenatal care for this pregnancy?	YES . . . . 1<br>NO . . . . 2 (SKIP TO 414)	
409	Whom did you see?<br>Anyone else?<br>PROBE TO IDENTIFY EACH TYPE OF PERSON AND RECORD ALL MENTIONED.	**HEALTH PERSONNEL**<br>DOCTOR . . . . A<br>NURSE/MIDWIFE . . . . B<br>AUXILIARY MIDWIFE . . . . . C<br>COMMUNITY EXTENSION HEALTH WORKER . . . . . D<br>**OTHER PERSON**<br>TRADITIONAL BIRTH ATTENDANT . . . . E<br>COMMUNITY/ VILLAGE HEALTH WORKER . . . . F<br>OTHER ______ (SPECIFY) X	

NO.	QUESTIONS AND FILTERS	LAST BIRTH<br>NAME ______	NEXT-TO-LAST BIRTH<br>NAME ______
410	Where did you receive antenatal care for this pregnancy?<br><br>Anywhere else?<br><br>PROBE TO IDENTIFY THE TYPE OF SOURCE.<br><br>IF UNABLE TO DETERMINE IF PUBLIC OR PRIVATE SECTOR, WRITE THE NAME OF THE PLACE.<br><br>______<br>(NAME OF PLACE)	**HOME**<br>HER HOME . . . . A<br>OTHER HOME . . . . B<br><br>**PUBLIC SECTOR**<br>GOVERNMENT HOSPITAL . . C<br>GOVERNMENT HEALTH CENTER . . . . D<br>GOVERNMENT HEALTH POST . . . . E<br>OTHER PUBLIC SECTOR<br>______ F<br>(SPECIFY)<br><br>**PRIVATE MEDICAL SECTOR**<br>PRIVATE HOSPITAL/ CLINIC . . . . G<br>OTHER PRIVATE MEDICAL SECTOR<br>______ H<br>(SPECIFY)<br><br>OTHER ______ X<br>(SPECIFY)	
411	How many months pregnant were you when you first received antenatal care for this pregnancy?	MONTHS . . . . [ ][ ]<br><br>DON'T KNOW 98	
412	How many times did you receive antenatal care during this pregnancy?	NUMBER OF TIMES . . . . . [ ][ ]<br><br>DON'T KNOW 98	
413	As part of your antenatal care during this pregnancy, were any of the following done at least once:<br><br>a) Was your blood pressure measured?<br>b) Did you give a urine sample?<br>c) Did you give a blood sample?	YES NO<br><br>a) BP . . . . 1 2<br>b) URINE . . . . 1 2<br>c) BLOOD 1 2	
414	During this pregnancy, were you given an injection in the arm to prevent the baby from getting tetanus, that is, convulsions after birth?	YES . . . . 1<br>NO . . . . 2<br>(SKIP TO 417)<br>DON'T KNOW 8	
415	During this pregnancy, how many times did you get a tetanus injection?	TIMES . . . . [ ]<br><br>DON'T KNOW 8	
416	CHECK 415:	2 OR MORE TIMES [ ] (SKIP TO 420)<br>OTHER [ ]	
417	At any time before this pregnancy, did you receive any tetanus injections?	YES . . . . 1<br>NO . . . . 2<br>(SKIP TO 420)<br>DON'T KNOW 8	

SECTION 4. PREGNANCY AND POSTNATAL CARE

NO.	QUESTIONS AND FILTERS	LAST BIRTH<br>NAME ______	NEXT-TO-LAST BIRTH<br>NAME ______
418	Before this pregnancy, how many times did you receive a tetanus injection?<br><br>IF 7 OR MORE TIMES, RECORD '7'.	TIMES . . . . [ ]<br>DON'T KNOW 8	
419	CHECK 418:<br>ONLY ONE [ ] / MORE THAN ONE TIME [ ]<br>a) How many years ago did you receive that tetanus injection?<br>b) How many years ago did you receive the last tetanus injection prior to this pregnancy?	YEARS AGO . . . . . [ ][ ]	
420	During this pregnancy, were you given or did you buy any iron tablets or iron syrup?<br><br>SHOW TABLETS/SYRUP.	YES . . . . 1<br>NO . . . . 2<br>(SKIP TO 422)<br>DON'T KNOW 8	
421	During the whole pregnancy, for how many days did you take the tablets or syrup?<br><br>IF ANSWER IS NOT NUMERIC, PROBE FOR APPROXIMATE NUMBER OF DAYS.	DAYS . . . . . [ ][ ][ ]<br>DON'T KNOW 998	
422	During this pregnancy, did you take any drug for intestinal worms?	YES . . . . 1<br>NO . . . . 2<br>DON'T KNOW 8	
423	During this pregnancy, did you take SP/Fansidar to keep you from getting malaria?	YES . . . . 1<br>NO . . . . 2<br>(SKIP TO 426)<br>DON'T KNOW 8	
424	How many times did you take SP/Fansidar during this pregnancy?	TIMES . . . . [ ][ ]	
425	Did you get the SP/Fansidar during any antenatal care visit, during another visit to a health facility or from another source?<br><br>IF MORE THAN ONE SOURCE, RECORD THE HIGHEST SOURCE ON THE LIST.	ANTENATAL VISIT . . . . 1<br>ANOTHER FACILITY VISIT . . . . . 2<br>COMMUNITY HEALTH EXTENSION WORKER . . . . . 3<br>OTHER SOURCE 6	
426	When (NAME) was born, was (NAME) very large, larger than average, average, smaller than average, or very small?	VERY LARGE . . . . 1<br>LARGER THAN AVERAGE . . . . 2<br>AVERAGE . . . . 3<br>SMALLER THAN AVERAGE . . . . 4<br>VERY SMALL . . . . 5<br>DON'T KNOW 8	VERY LARGE . . . . 1<br>LARGER THAN AVERAGE . . . . 2<br>AVERAGE . . . . 3<br>SMALLER THAN AVERAGE . . . . 4<br>VERY SMALL . . . . 5<br>DON'T KNOW 8
427	Was (NAME) weighed at birth?	YES . . . . 1<br>NO . . . . 2<br>(SKIP TO 429)<br>DON'T KNOW 8	YES . . . . 1<br>NO . . . . 2<br>(SKIP TO 429)<br>DON'T KNOW 8

NO.	QUESTIONS AND FILTERS	LAST BIRTH<br>NAME ______	NEXT-TO-LAST BIRTH<br>NAME ______
428	How much did (NAME) weigh?<br><br>RECORD WEIGHT IN KILOGRAMS FROM HEALTH CARD, IF AVAILABLE.	KG FROM CARD<br>1 [ ] . [ ][ ][ ]<br>KG FROM RECALL<br>2 [ ] . [ ][ ][ ]<br>DON'T KNOW 99998	KG FROM CARD<br>1 [ ] . [ ][ ][ ]<br>KG FROM RECALL<br>2 [ ] . [ ][ ][ ]<br>DON'T KNOW 99998
429	Who assisted with the delivery of (NAME)?<br><br>Anyone else?<br><br>PROBE FOR THE TYPE(S) OF PERSON(S) AND RECORD ALL MENTIONED.<br><br>IF RESPONDENT SAYS NO ONE ASSISTED, PROBE TO DETERMINE WHETHER ANY ADULTS WERE PRESENT AT THE DELIVERY.	**HEALTH PERSONNEL**<br>DOCTOR .... A<br>NURSE/MIDWIFE .... B<br>COMMUNITY HEALTH EXTENSION WORKER .. C<br>AUXILIARY MIDWIFE .... D<br>**OTHER PERSON**<br>TRADITIONAL BIRTH ATTENDANT .... E<br>RELATIVE/FRIEND .... F<br>OTHER ______ X<br>(SPECIFY)<br>NO ONE ASSISTED .... Y<br>(E, F, X, Y: SKIP TO 430)	**HEALTH PERSONNEL**<br>DOCTOR .... A<br>NURSE/MIDWIFE .... B<br>COMMUNITY HEALTH EXTENSION WORKER .. C<br>AUXILIARY MIDWIFE .... D<br>**OTHER PERSON**<br>TRADITIONAL BIRTH ATTENDANT .... E<br>RELATIVE/FRIEND .... F<br>OTHER ______ X<br>(SPECIFY)<br>NO ONE ASSISTED .... Y<br>(E, F, X, Y: SKIP TO 430)
429A	Immediately after delivery of (NAME) did you receive an injection in the thigh or buttock?	YES .... 1<br>NO .... 2<br>DON'T KNOW 8	YES .... 1<br>NO .... 2<br>DON'T KNOW 8
430	Where did you give birth to (NAME)?<br><br>PROBE TO IDENTIFY THE TYPE OF SOURCE.<br><br>IF UNABLE TO DETERMINE IF PUBLIC OR PRIVATE SECTOR, WRITE THE NAME OF THE PLACE.<br><br>______<br>(NAME OF PLACE)	**HOME**<br>HER HOME .... 11<br>OTHER HOME .... 12<br>(11, 12: SKIP TO 434)<br>**PUBLIC SECTOR**<br>GOVERNMENT HOSPITAL .. 21<br>GOVERNMENT HEALTH CENTER .... 22<br>GOVERNMENT HEALTH POST .... 23<br>OTHER PUBLIC SECTOR ______ 26<br>(SPECIFY)<br>**PRIVATE MEDICAL SECTOR**<br>PRIVATE HOSPITAL/ CLINIC .... 31<br>OTHER PRIVATE MEDICAL SECTOR ______ 36<br>(SPECIFY)<br>OTHER ______ 96<br>(SPECIFY)<br>(SKIP TO 434)	**HOME**<br>HER HOME .... 11<br>OTHER HOME .... 12<br>(11, 12: SKIP TO 459)<br>**PUBLIC SECTOR**<br>GOVERNMENT HOSPITAL .. 21<br>GOVERNMENT HEALTH CENTER .... 22<br>GOVERNMENT HEALTH POST .... 23<br>OTHER PUBLIC SECTOR ______ 26<br>(SPECIFY)<br>**PRIVATE MEDICAL SECTOR**<br>PRIVATE HOSPITAL/ CLINIC .... 31<br>OTHER PRIVATE MEDICAL SECTOR ______ 36<br>(SPECIFY)<br>OTHER ______ 96<br>(SPECIFY)<br>(SKIP TO 459)

NO.	QUESTIONS AND FILTERS	LAST BIRTH<br>NAME ______	NEXT-TO-LAST BIRTH<br>NAME ______
430A	Did you move from another health facility to come to this facility or did you go directly from home to this facility, or from somewhere else that was not a health facility?	CAME FROM ANOTHER HEALTH FACILITY . . . . 1<br>CAME FROM HOME . . . . 2<br>CAME FROM OTHER NON-FACILITY LOCATION . . 3<br>DON'T KNOW . . . . 8<br>(SKIP TO 430F)	CAME FROM ANOTHER HEALTH FACILITY . . . . 1<br>CAME FROM HOME . . . . 2<br>CAME FROM OTHER NON-FACILITY LOCATION . . 3<br>DON'T KNOW . . . . 8<br>(SKIP TO 430F)
430B	Which health facility referred or send you to this facility where you gave birth to (NAME)?<br><br>PROBE TO IDENTIFY THE TYPE OF SOURCE.<br><br>IF UNABLE TO DETERMINE IF PUBLIC OR PRIVATE SECTOR, WRITE THE NAME OF THE PLACE.<br><br>______<br>(NAME OF PLACE)	**PUBLIC SECTOR**<br>GOVERNMENT HOSPITAL . . 21<br>GOVERNMENT HEALTH CENTER . . . . 22<br>GOVERNMENT HEALTH POST . . . . 23<br>OTHER PUBLIC SECTOR<br>______ 26<br>(SPECIFY)<br><br>**PRIVATE MEDICAL SECTOR**<br>PRIVATE HOSPITAL/ CLINIC . . . . 31<br>OTHER PRIVATE MEDICAL SECTOR<br>______ 36<br>(SPECIFY)<br><br>NO FORMAL REFERRAL . . . . . 41<br><br>OTHER ______ 96<br>(SPECIFY)	**PUBLIC SECTOR**<br>GOVERNMENT HOSPITAL . . 21<br>GOVERNMENT HEALTH CENTER . . . . 22<br>GOVERNMENT HEALTH POST . . . . 23<br>OTHER PUBLIC SECTOR<br>______ 26<br>(SPECIFY)<br><br>**PRIVATE MEDICAL SECTOR**<br>PRIVATE HOSPITAL/ CLINIC . . . . 31<br>OTHER PRIVATE MEDICAL SECTOR<br>______ 36<br>(SPECIFY)<br><br>NO FORMAL REFERRAL . . . . . 41<br><br>OTHER ______ 96<br>(SPECIFY)
430C	Why did you move from this facility to the facility where you gave birth to (NAME)?	PROBLEM DURING LABOR/ EMERGENCY . . . . 1<br>HEALTH PROFESSIONAL NOT AVAILABLE . . . . 2<br>FACILITY TOO CROWDED/ NO BED AVAILABLE . . . . . 3<br>FACILITY NOT OPEN . . . . 4<br><br>OTHER ______ 6<br>(SPECIFY)	PROBLEM DURING LABOR/ EMERGENCY . . . . 1<br>HEALTH PROFESSIONAL NOT AVAILABLE . . . . 2<br>FACILITY TOO CROWDED/ NO BED AVAILABLE . . . . . 3<br>FACILITY NOT OPEN . . . . 4<br><br>OTHER ______ 6<br>(SPECIFY)
430D	Did a health worker go with you when you moved to the facility where you gave birth to (NAME)?	YES . . . . 1<br>NO . . . . 2<br>DON'T KNOW 8	YES . . . . 1<br>NO . . . . 2<br>DON'T KNOW 8

NO.	QUESTIONS AND FILTERS	LAST BIRTH<br>NAME ________	NEXT-TO-LAST BIRTH<br>NAME ________
430E<br><br>430F	What means of transportation did you use to get from the facility that referred you to the faclity where you gave birth to (NAME)?<br><br>What means of transportation did you use to get to the health facility where you gave birth to (NAME)?<br><br>PROBE FOR THE TYPE (S) OF TRANSPORT USED AND RECORD ALL MENTIONED.	**MOTORISED**<br>AMBULANCE . . . . . A<br>PRIVATE CAR/TRUCK . . . . . B<br>TAXI/PAID DRIVER . . . . . C<br>TRICYCLE . . . . . D<br>MOTORCYCLE/SCOOTER . . E<br>BOAT WITH MOTOR . . . . . F<br>PUBLIC TRANSPORT/BUS . . G<br><br>**NOT MOTORISED**<br>BICYCLE . . . . . H<br>CANOE/BOAT WITHOUT MOTOR . . . . . I<br>ANIMAL-DRAWN CART . . . . . J<br>WALKING (ON FOOT) . . . . . K<br>CARRIED . . . . . L<br><br>OTHER ________ X<br>(SPECIFY)<br>DON'T KNOW . . . . . Z	**MOTORISED**<br>AMBULANCE . . . . . A<br>PRIVATE CAR/TRUCK . . . . . B<br>TAXI/PAID DRIVER . . . . . C<br>TRICYCLE . . . . . D<br>MOTORCYCLE/SCOOTER . . E<br>BOAT WITH MOTOR . . . . . F<br>PUBLIC TRANSPORT/BUS . . G<br><br>**NOT MOTORISED**<br>BICYCLE . . . . . H<br>CANOE/BOAT WITHOUT MOTOR . . . . . I<br>ANIMAL-DRAWN CART . . . . . J<br>WALKING (ON FOOT) . . . . . K<br>CARRIED . . . . . L<br><br>OTHER ________ X<br>(SPECIFY)<br>DON'T KNOW . . . . . Z
430G	How long did it take for you to decide to go and reach the health facility?<br><br>IF LESS THAN ONE HOUR, RECORD IN MINUTES.	MINUTES . . . . . 1 [ ][ ]<br>HOURS . . . . . 2 [ ][ ]<br><br>DON'T KNOW 998	MINUTES . . . . . 1 [ ][ ]<br>HOURS . . . . . 2 [ ][ ]<br><br>DON'T KNOW 998
431	How long after (NAME) was delivered did you stay there?<br><br>IF LESS THAN ONE DAY, RECORD HOURS;<br>IF LESS THAN ONE WEEK, RECORD DAYS.	HOURS . . . . . 1 [ ][ ]<br>DAYS . . . . . 2 [ ][ ]<br>WEEKS . . . . . 3 [ ][ ]<br><br>DON'T KNOW 998	
432	Was (NAME) delivered by caesarean, that is, did they cut your belly open to take the baby out?	YES . . . . . 1<br>NO . . . . . 2<br>(SKIP TO 434)	YES . . . . . 1<br>NO . . . . . 2<br>(SKIP TO 459)
433	When was the decision made to have the caesarean section? Was it before or after your labor pains started?	BEFORE . . . . . 1<br>AFTER 2	BEFORE . . . . . 1<br>AFTER 2
433A	What was the reason for taking the decision to have the caesarean section?	EXCESS BLEEDING . . . . . 1<br>BREECH POSITON . . . . . 2<br>MEDICAL CONDITION OF MOTHER . . . . . 3<br>CORD PROBLEM . . . . . 4<br>VOLUNTARY . . . . . 5<br><br>OTHER ________ 6<br>(SPECIFY)	EXCESS BLEEDING . . . . . 1<br>BREECH POSITON . . . . . 2<br>MEDICAL CONDITION OF MOTHER . . . . . 3<br>CORD PROBLEM . . . . . 4<br>VOLUNTARY . . . . . 5<br><br>OTHER ________ 6<br>(SPECIFY)
434	Immediately after the birth, was (NAME) put on your chest?	YES . . . . . 1<br>NO . . . . . 2<br>(SKIP TO 434B)<br>DON'T KNOW 8	
434A	Was (NAME)'s bare skin touching your bare skin?	YES . . . . . 1<br>NO . . . . . 2<br>DON'T KNOW 8	

NO.	QUESTIONS AND FILTERS	LAST BIRTH<br>NAME ______	NEXT-TO-LAST BIRTH<br>NAME ______
434B	Was (NAME) wiped dry within a few minutes after birth?	YES . . . 1<br>NO . . . 2<br>DON'T KNOW . . . 8	
434C	How long after the birth was (NAME) bathed for the first time?<br><br>IF LESS THAN ONE HOUR, RECORD `00' HOURS;<br>IF LESS THAN 24 HOURS, RECORD HOURS; OTHERWISE, RECORD DAYS.	IMMEDIATELY . . . 000<br>HOURS . . . 1 [ ][ ]<br>DAYS . . . 2 [ ][ ]<br>DON'T KNOW . . . 998	
434D	CHECK 430: PLACE OF DELIVERY	CODE 11, 12, OR 96 CIRCLED [ ] ↓<br>OTHER [ ] (SKIP TO 434H)	
434E	What was used to cut the cord?	RAZOR BLADE . . . 1<br>KNIFE . . . 2<br>SCISSORS . . . 3<br>SICKLE . . . 4<br>OTHER ______ 6<br>(SPECIFY)<br>DON'T KNOW . . . 8	
434F	Was it new or had it ever been used before?	NEW . . . 1<br>USED BEFORE . . . 2<br>DON'T KNOW . . . 8	
434G	Was it boiled before it was used to cut the cord?	YES . . . 1<br>NO . . . 2<br>DON'T KNOW . . . 8	
434H	Was anything applied to the stump of the cord at any time?	YES . . . 1<br>NO . . . 2<br>DON'T KNOW . . . 8<br>(NO, DON'T KNOW: SKIP TO 434M)	
434I	What was applied?<br><br>Anything else?	CHLORHEXIDINE . . . A<br>OTHER ANTISEPTIC (ALCOHOL, SPIRIT, GENTIAN VIOLET, DETOL) . . . B<br>OLIVE OIL . . . C<br>ASH . . . D<br>ANIMAL DUNG . . . E<br>TURMERIC . . . F<br>OTHER ______ X<br>(SPECIFY)<br>DON'T KNOW . . . Z	
434J	CHECK 434I: SUBSTANCE APPLIED TO CORD	CODE `A' NOT CIRCLED [ ] ↓<br>CODE `A' CIRCLED [ ] (SKIP TO 434L)	
434K	Was chlorohexidine applied to the stump at any time?<br><br>SHOW SAMPLE OF CHLORHEXIDINE	YES . . . 1<br>NO . . . 2<br>DON'T KNOW . . . 8<br>(NO, DON'T KNOW: SKIP TO 434M)	

NO.	QUESTIONS AND FILTERS	LAST BIRTH<br>NAME ______	NEXT-TO-LAST BIRTH<br>NAME ______
434L	How long after the cord was cut was chlorhexidine first applied?<br><br>IF LESS THAN 1 HOUR, RECORD `00' HOURS; IF LESS THAN 24 HOURS, RECORD HOURS; OTHERWISE, RECORD DAYS.	HOURS . . . . 1 [ ][ ]<br>DAYS . . . . 2 [ ][ ]<br>DON'T KNOW 998	
434M	CHECK 430: PLACE OF DELIVERY	CODE 11, 12, OR 96 CIRCLED [ ] (SKIP TO 449)<br>OTHER [ ]	
435	I would like to talk to you about checks on your health after delivery, for example, someone asking you questions about your health or examining you. Did anyone check on your health while you were still in the facility?	YES . . . . 1<br>NO . . . . 2 (SKIP TO 438)	
436	How long after delivery did the first check take place?<br><br>IF LESS THAN ONE DAY, RECORD HOURS;<br>IF LESS THAN ONE WEEK, RECORD DAYS.	HOURS . . . . 1 [ ][ ]<br>DAYS . . . . 2 [ ][ ]<br>WEEKS . . . . 3 [ ][ ]<br>DON'T KNOW 998	
437	Who checked on your health at that time?<br><br>PROBE FOR MOST QUALIFIED PERSON.	**HEALTH PERSONNEL**<br>DOCTOR . . . . 11<br>NURSE/MIDWIFE . . . . 12<br>COMMUNITY HEALTH EXTENSION WORKER . . 13<br>AUXILIARY MIDWIFE . . . . 14<br>**OTHER PERSON**<br>TRADITIONAL BIRTH ATTENDANT . . . . 21<br>COMMUNITY/ VILLAGE HEALTH WORKER . . . . 22<br><br>OTHER ______ 96<br>(SPECIFY)	
438	Now I would like to talk to you about checks on (NAME)'s health after delivery – for example, someone examining (NAME), checking the cord, or seeing if (NAME) is OK. Did anyone check on (NAME)'s health while you were still in the facility?	YES . . . . 1<br>NO . . . . 2<br>(SKIP TO 441)<br>DON'T KNOW 8	
439	How long after delivery was (NAME)'s health first checked?<br><br>IF LESS THAN ONE DAY, RECORD HOURS;<br>IF LESS THAN ONE WEEK, RECORD DAYS.	HOURS . . . . 1 [ ][ ]<br>DAYS . . . . 2 [ ][ ]<br>WEEKS . . . . 3 [ ][ ]<br>DON'T KNOW 998	

NO.	QUESTIONS AND FILTERS	LAST BIRTH<br>NAME ______	NEXT-TO-LAST BIRTH<br>NAME ______
440	Who checked on (NAME)'s health at that time?<br><br>PROBE FOR MOST QUALIFIED PERSON.	**HEALTH PERSONNEL**<br>DOCTOR . . . . 11<br>NURSE/MIDWIFE . . . . 12<br>COMMUNITY HEALTH EXTENSION WORKER . . 13<br>AUXILIARY MIDWIFE . . . . 14<br>**OTHER PERSON**<br>TRADITIONAL BIRTH ATTENDANT . . . . 21<br>COMMUNITY/ VILLAGE HEALTH WORKER . . . . 22<br><br>OTHER ______ 96<br>(SPECIFY)	
441	Now I want to talk to you about what happened after you left the facility. Did anyone check on your health after you left the facility?	YES . . . . 1<br>NO . . . . 2<br>(SKIP TO 445)	
442	How long after delivery did that check take place?<br><br>IF LESS THAN ONE DAY, RECORD HOURS;<br>IF LESS THAN ONE WEEK, RECORD DAYS.	HOURS . . . . 1 [ ][ ]<br>DAYS . . . . 2 [ ][ ]<br>WEEKS . . . . 3 [ ][ ]<br><br>DON'T KNOW 998	
443	Who checked on your health at that time?<br><br>PROBE FOR MOST QUALIFIED PERSON.	**HEALTH PERSONNEL**<br>DOCTOR . . . . 11<br>NURSE/MIDWIFE . . . . 12<br>COMMUNITY HEALTH EXTENSION WORKER . . 13<br>AUXILIARY MIDWIFE . . . . 14<br>**OTHER PERSON**<br>TRADITIONAL BIRTH ATTENDANT . . . . 21<br>COMMUNITY/ VILLAGE HEALTH WORKER . . . . 22<br><br>OTHER ______ 96<br>(SPECIFY)	

NO.	QUESTIONS AND FILTERS	LAST BIRTH<br>NAME ______	NEXT-TO-LAST BIRTH<br>NAME ______
444	Where did the check take place?<br><br>PROBE TO IDENTIFY THE TYPE OF SOURCE.<br><br>IF UNABLE TO DETERMINE IF PUBLIC OR PRIVATE SECTOR, WRITE THE NAME OF THE PLACE.<br><br>______<br>(NAME OF PLACE)	**HOME**<br>HER HOME . . . . 11<br>OTHER HOME . . . . 12<br><br>**PUBLIC SECTOR**<br>GOVERNMENT HOSPITAL . . 21<br>GOVERNMENT HEALTH CENTER . . . . 22<br>GOVERNMENT HEALTH POST . . . . 23<br>OTHER PUBLIC SECTOR<br>______ 26<br>(SPECIFY)<br><br>**PRIVATE MEDICAL SECTOR**<br>PRIVATE HOSPITAL/ CLINIC . . . . 31<br>OTHER PRIVATE MEDICAL SECTOR<br>______ 36<br>(SPECIFY)<br><br>OTHER ______ 96<br>(SPECIFY)	
445	I would like to talk to you about checks on (NAME)'s health after you left (FACILITY IN 430). Did any health care provider or a traditional birth attendant check on (NAME)'s health in the two months after you left (FACILITY IN 430)?	YES . . . . 1<br>NO . . . . 2<br>(SKIP TO 457) ←<br>DON'T KNOW 8	
446	How many hours, days or weeks after the birth of (NAME) did that check take place?<br><br>IF LESS THAN ONE DAY, RECORD HOURS;<br>IF LESS THAN ONE WEEK, RECORD DAYS.	HOURS . . . . 1 [ ][ ]<br>DAYS . . . . 2 [ ][ ]<br>WEEKS . . . . 3 [ ][ ]<br>DON'T KNOW 998	
447	Who checked on (NAME)'s health at that time?<br><br>PROBE FOR MOST QUALIFIED PERSON.	**HEALTH PERSONNEL**<br>DOCTOR . . . . 11<br>NURSE/MIDWIFE . . . . 12<br>COMMUNITY HEALTH EXTENSION WORKER . . 13<br>AUXILIARY MIDWIFE . . . . 14<br>**OTHER PERSON**<br>TRADITIONAL BIRTH ATTENDANT . . . . 21<br>COMMUNITY/ VILLAGE HEALTH WORKER . . . . 22<br><br>OTHER ______ 96<br>(SPECIFY)	

NO.	QUESTIONS AND FILTERS	LAST BIRTH<br>NAME ________	NEXT-TO-LAST BIRTH<br>NAME ________
448	Where did this check of (NAME) take place?<br><br>PROBE TO IDENTIFY THE TYPE OF SOURCE.<br><br>IF UNABLE TO DETERMINE IF PUBLIC OR PRIVATE SECTOR, WRITE THE NAME OF THE PLACE.<br><br>________<br>(NAME OF PLACE)	**HOME**<br>HER HOME . . . . 11<br>OTHER HOME . . . . 12<br><br>**PUBLIC SECTOR**<br>GOVERNMENT HOSPITAL . . 21<br>GOVERNMENT HEALTH CENTER . . . . 22<br>GOVERNMENT HEALTH POST . . . . 23<br>OTHER PUBLIC SECTOR<br>________ 26<br>(SPECIFY)<br><br>**PRIVATE MEDICAL SECTOR**<br>PRIVATE HOSPITAL/ CLINIC . . . . 31<br>OTHER PRIVATE MEDICAL SECTOR<br>________ 36<br>(SPECIFY)<br><br>OTHER ________ 96<br>(SPECIFY)<br><br>(SKIP TO 457)	
449	I would like to talk to you about checks on your health after delivery, for example, someone asking you questions about your health or examining you. Did anyone check on your health after you gave birth to (NAME)?	YES . . . . 1<br>NO . . . . 2<br>(SKIP TO 453)	
450	How long after delivery did the first check take place?<br><br>IF LESS THAN ONE DAY, RECORD HOURS;<br>IF LESS THAN ONE WEEK, RECORD DAYS.	HOURS . . . . 1 [ ][ ]<br>DAYS . . . . 2 [ ][ ]<br>WEEKS . . . . 3 [ ][ ]<br><br>DON'T KNOW 998	
451	Who checked on your health at that time?<br><br>PROBE FOR MOST QUALIFIED PERSON.	**HEALTH PERSONNEL**<br>DOCTOR . . . . 11<br>NURSE/MIDWIFE . . . . 12<br>COMMUNITY HEALTH EXTENSION WORKER . . 13<br>AUXILIARY MIDWIFE . . . . 14<br>**OTHER PERSON**<br>TRADITIONAL BIRTH ATTENDANT . . . . 21<br>COMMUNITY/ VILLAGE HEALTH WORKER . . . . 22<br><br>OTHER ________ 96<br>(SPECIFY)	

NO.	QUESTIONS AND FILTERS	LAST BIRTH<br>NAME ______	NEXT-TO-LAST BIRTH<br>NAME ______
452	Where did this first check take place?<br><br>PROBE TO IDENTIFY THE TYPE OF SOURCE.<br><br>IF UNABLE TO DETERMINE IF PUBLIC OR PRIVATE SECTOR, WRITE THE NAME OF THE PLACE.<br><br>______<br>(NAME OF PLACE)	**HOME**<br>HER HOME . . . . 11<br>OTHER HOME . . . . 12<br><br>**PUBLIC SECTOR**<br>GOVERNMENT HOSPITAL . . 21<br>GOVERNMENT HEALTH CENTER . . . . 22<br>GOVERNMENT HEALTH POST . . . . 23<br>OTHER PUBLIC SECTOR<br>______ 26<br>(SPECIFY)<br><br>**PRIVATE MEDICAL SECTOR**<br>PRIVATE HOSPITAL/ CLINIC . . . . 31<br>OTHER PRIVATE MEDICAL SECTOR<br>______ 36<br>(SPECIFY)<br><br>OTHER ______ 96<br>(SPECIFY)	
453	I would like to talk to you about checks on (NAME)'s health after delivery – for example, someone examining (NAME), checking the cord, or seeing if (NAME) is OK. In the two months after (NAME) was born, did any health care provider or a traditional birth attendant check on (NAME)'s health?	YES . . . . 1<br>NO . . . . 2<br>(SKIP TO 457)<br>DON'T KNOW 8	
454	How many hours, days or weeks after the birth of (NAME) did the first check take place?<br><br>IF LESS THAN ONE DAY, RECORD HOURS;<br>IF LESS THAN ONE WEEK, RECORD DAYS.	HOURS AFTER BIRTH . . . . . 1 [ ][ ]<br>DAYS AFTER BIRTH . . . . . 2 [ ][ ]<br>WEEKS AFTER BIRTH . . . . . 3 [ ][ ]<br><br>DON'T KNOW 998	
455	Who checked on (NAME)'s health at that time?<br><br>PROBE FOR MOST QUALIFIED PERSON.	**HEALTH PERSONNEL**<br>DOCTOR . . . . 11<br>NURSE/MIDWIFE . . . . 12<br>COMMUNITY HEALTH EXTENSION WORKER . . 13<br>AUXILIARY MIDWIFE . . . . 14<br>**OTHER PERSON**<br>TRADITIONAL BIRTH ATTENDANT . . . . 21<br>COMMUNITY/ VILLAGE HEALTH WORKER . . . . 22<br><br>OTHER ______ 96<br>(SPECIFY)	

NO.	QUESTIONS AND FILTERS	LAST BIRTH<br>NAME ______	NEXT-TO-LAST BIRTH<br>NAME ______
456	Where did this first check of (NAME) take place?<br><br>PROBE TO IDENTIFY THE TYPE OF SOURCE.<br><br>IF UNABLE TO DETERMINE IF PUBLIC OR PRIVATE SECTOR, WRITE THE NAME OF THE PLACE.<br><br>______<br>(NAME OF PLACE)	**HOME**<br>HER HOME . . . . 11<br>OTHER HOME . . . . 12<br><br>**PUBLIC SECTOR**<br>GOVERNMENT HOSPITAL . . 21<br>GOVERNMENT HEALTH CENTER . . . . 22<br>GOVERNMENT HEALTH POST . . . . 23<br>OTHER PUBLIC SECTOR<br>______ 26<br>(SPECIFY)<br><br>**PRIVATE MEDICAL SECTOR**<br>PRIVATE HOSPITAL/ CLINIC . . . . 31<br>OTHER PRIVATE MEDICAL SECTOR<br>______ 36<br>(SPECIFY)<br><br>OTHER ______ 96<br>SPECIFY	
457	During the first two days after (NAME)'s birth, did any health care provider do the following:<br>a) Examine the cord?<br>b) Measure (NAME)'s temperature?<br>c) Counsel you on danger signs for newborns?<br>d) Counsel you on breastfeeding?<br>e) Observe (NAME) breastfeeding?	YES NO DK<br>a) CORD . . . . 1 2 8<br>b) TEMP. . . . . . 1 2 8<br>c) SIGNS . . . . . 1 2 8<br>d) COUNSEL BREAST-FEED 1 2 8<br>e) OBSERVE BREAST-FEED 1 2 8	
458	Has your menstrual period returned since the birth of (NAME)?	YES . . . . 1<br>(SKIP TO 460)<br>NO . . . . 2<br>(SKIP TO 461)	
459	Did your period return between the birth of (NAME) and your next pregnancy?		YES . . . . 1<br>NO . . . . 2<br>(SKIP TO 463)
460	For how many months after the birth of (NAME) did you not have a period?	MONTHS . . . . [ ][ ]<br><br>DON'T KNOW 98	MONTHS . . . . [ ][ ]<br><br>DON'T KNOW 98
461	CHECK 226: IS RESPONDENT PREGNANT?	NOT PREGNANT [ ]<br>PREGNANT OR UNSURE [ ]<br>(SKIP TO 463)	
462	Have you had sexual intercourse since the birth of (NAME)?	YES . . . . 1<br>NO . . . . 2<br>(SKIP TO 464)	

NO.	QUESTIONS AND FILTERS	LAST BIRTH<br>NAME ________	NEXT-TO-LAST BIRTH<br>NAME ________
463	For how many months after the birth of (NAME) did you not have sexual intercourse?	MONTHS . . . . [ ][ ]<br>DON'T KNOW 98	MONTHS . . . . [ ][ ]<br>DON'T KNOW 98
464	Did you ever breastfeed (NAME)?	YES . . . . 1 (SKIP TO 466)<br>NO 2	YES . . . . 1<br>NO 2
465	CHECK 404: IS CHILD LIVING?	LIVING [ ] (SKIP TO 470)<br>DEAD [ ] (SKIP TO 471)	
466	How long after birth did you first put (NAME) to the breast?<br><br>IF LESS THAN 1 HOUR, RECORD ‘00' HOURS;<br>IF LESS THAN 24 HOURS, RECORD HOURS;<br>OTHERWISE, RECORD DAYS.	IMMEDIATELY . . . . 000<br>HOURS . . . . 1 [ ][ ]<br>DAYS . . . . 2 [ ][ ]	
467	In the first three days after delivery, was (NAME) given anything to drink other than breast milk?	YES . . . . 1<br>NO 2	
468	CHECK 404: IS CHILD LIVING?	LIVING [ ]<br>DEAD [ ] (SKIP TO 471)	LIVING [ ]<br>DEAD [ ] (SKIP TO 471)
469	Are you still breastfeeding (NAME)?	YES . . . . 1<br>NO 2	
470	Did (NAME) drink anything from a bottle with a nipple yesterday or last night?	YES . . . . 1<br>NO . . . . 2<br>DON'T KNOW 8	YES . . . . 1<br>NO . . . . 2<br>DON'T KNOW 8
471		GO BACK TO 405 IN NEXT COLUMN; OR, IF NO MORE BIRTHS, GO TO 501A.	GO BACK TO 405 IN NEXT-TO-LAST COLUMN OF NEW QUESTIONNAIRE; OR, IF NO MORE BIRTHS, GO TO 501A.

SECTION 5A. CHILD IMMUNIZATION (LAST BIRTH)

NO.	QUESTIONS AND FILTERS	CODING CATEGORIES	SKIP
501A	CHECK 215 IN THE BIRTH HISTORY: ANY BIRTHS IN 2015-2018?<br>ONE OR MORE BIRTHS IN 2015-2018 ☐	NO BIRTHS IN 2015-2018 ☐	→ 601
502A	RECORD THE NAME AND BIRTH HISTORY NUMBER FROM 212 OF THE LAST CHILD BORN IN 2015-2018.<br>NAME OF LAST BIRTH ____________	BIRTH HISTORY NUMBER . . . . ☐☐	
503A	CHECK 216 FOR CHILD:<br>LIVING ☐	DEAD ☐	→ 501B
504A	Do you have a card or other document where (NAME)'s vaccinations are written down?	YES, HAS ONLY A CARD . . . . 1<br>YES, HAS ONLY AN OTHER DOCUMENT . . . . . 2<br>YES, HAS CARD AND OTHER DOCUMENT . . . . . 3<br>NO, NO CARD AND NO OTHER DOCUMENT . . 4	1 → 507A<br><br>3 → 507A
505A	Did you ever have a vaccination card for (NAME)?	YES . . . . 1<br>NO 2	
506A	CHECK 504A:<br>CODE '2' CIRCLED ☐	CODE '4' CIRCLED ☐	→ 511A
507A	May I see the card or other document where (NAME)'s vaccinations are written down?	YES, ONLY CARD SEEN . . . . 1<br>YES, ONLY OTHER DOCUMENT SEEN . . . . 2<br>YES, CARD AND OTHER DOCUMENT SEEN . . 3<br>NO CARD AND NO OTHER DOCUMENT SEEN . . 4	 <br><br>4 → 511A

SECTION 5A. CHILD IMMUNIZATION (LAST BIRTH)

NO.	QUESTIONS AND FILTERS	CODING CATEGORIES	SKIP
	NAME OF LAST BIRTH ______________________	BIRTH HISTORY NUMBER . . . . [ ][ ]	
508A	COPY DATES FROM THE CARD.<br>WRITE '44' IN 'DAY' COLUMN IF CARD SHOWS THAT A DOSE WAS GIVEN, BUT NO DATE IS RECORDED.		

	DAY	MONTH	YEAR
BCG			
HEPATITIS B AT BIRTH			
ORAL POLIO VACCINE (OPV) 0 (BIRTH DOSE)			
ORAL POLIO VACCINE (OPV) 1			
ORAL POLIO VACCINE (OPV) 2			
ORAL POLIO VACCINE (OPV) 3			
DPT-HEP.B-HIB (PENTAVALENT) 1			
DPT-HEP.B-HIB (PENTAVALENT) 2			
DPT-HEP.B-HIB (PENTAVALENT) 3			
PNEUMOCOCCAL 1			
PNEUMOCOCCAL 2			
PNEUMOCOCCAL 3			
INACTIVATED POLIO VIRUS (IPV)			
MEASLES 1			
MEASLES/MMR 2			
VITAMIN A (MOST RECENT)			

NO.	QUESTIONS AND FILTERS	CODING CATEGORIES	SKIP
509A	CHECK 508A: 'BCG' TO 'MEASLES/MMR 2' ALL RECORDED?<br>NO [ ] ↓ YES [ ]		526A
510A	In addition to what is recorded on (this document/these documents), did (NAME) receive any other vaccinations, including vaccinations received in campaigns or immunization days or child health days?<br><br>RECORD 'YES' ONLY IF THE RESPONDENT MENTIONS AT LEAST ONE OF THE VACCINATIONS IN 508A THAT ARE NOT RECORDED AS HAVING BEEN GIVEN.	YES . . . . 1<br>(PROBE FOR VACCINATIONS AND WRITE '66' IN THE CORRESPONDING DAY COLUMN IN 508A THEN WRITE '00' IN THE CORRESPONDING DAY COLUMN FOR ALL VACCINATIONS NOT GIVEN)<br>(THEN SKIP TO 526A)<br><br>NO . . . . 2<br>DON'T KNOW . . . . 8<br>(WRITE '00' IN THE CORRESPONDING DAY COLUMN FOR ALL VACCINATIONS NOT GIVEN)<br>(THEN SKIP TO 526A)	

SECTION 5A. CHILD IMMUNIZATION (LAST BIRTH)

NO.	QUESTIONS AND FILTERS	CODING CATEGORIES	SKIP
	NAME OF LAST BIRTH ________________	BIRTH HISTORY NUMBER . . . . [ ][ ]	
511A	Did (NAME) ever receive any vaccinations to prevent (NAME) from getting diseases, including vaccinations received in campaigns or immunization days or child health days?	YES . . . . . 1<br>NO . . . . . 2<br>DON'T KNOW 8	 2, 8 → 526A
512A	Has (NAME) ever received a BCG vaccination against tuberculosis, that is, an injection in the arm or shoulder that usually causes a scar?	YES . . . . . 1<br>NO . . . . . 2<br>DON'T KNOW 8	
513A	Within 24 hours after birth, did (NAME) receive a Hepatitis B vaccination, that is, an injection in the thigh to prevent Hepatitis B?	YES . . . . . 1<br>NO . . . . . 2<br>DON'T KNOW 8	
514A	Has (NAME) ever received oral polio vaccine, that is, about two drops in the mouth to prevent polio?	YES . . . . . 1<br>NO . . . . . 2<br>DON'T KNOW 8	 2, 8 → 517A
515A	Did (NAME) receive the first oral polio vaccine in the first two weeks after birth or later?	FIRST TWO WEEKS . . . . . 1<br>LATER 2	
516A	How many times did (NAME) receive the oral polio vaccine?	NUMBER OF TIMES . . . . . [ ]	
517A	Has (NAME) ever received a pentavalent vaccination, that is, an injection given in the thigh sometimes at the same time as polio drops?	YES . . . . . 1<br>NO . . . . . 2<br>DON'T KNOW 8	 2, 8 → 519A
518A	How many times did (NAME) receive the pentavalent vaccine?	NUMBER OF TIMES . . . . . [ ]	
519A	Has (NAME) ever received a pneumococcal vaccination, that is, an injection in the thigh to prevent pneumonia?	YES . . . . . 1<br>NO . . . . . 2<br>DON'T KNOW 8	 2, 8 → 521A
520A	How many times did (NAME) receive the pneumococcal vaccine?	NUMBER OF TIMES . . . . . [ ]	
521A	Has (NAME) ever received an inactivated polio vaccine (IPV), that is, an injection in the thigh to prevent polio?	YES . . . . . 1<br>NO . . . . . 2<br>DON'T KNOW 8	
523A	Has (NAME) ever received a measles vaccination, that is, an injection in the arm to prevent measles?	YES . . . . . 1<br>NO . . . . . 2<br>DON'T KNOW 8	 2, 8 → 526A
524A	How many times did (NAME) receive the measles vaccine?	NUMBER OF TIMES . . . . . [ ]	
526A	CONTINUE WITH 501B.		

SECTION 5B. CHILD IMMUNIZATION (NEXT-TO-LAST BIRTH)

NO.	QUESTIONS AND FILTERS	CODING CATEGORIES	SKIP
501B	CHECK 215 IN THE BIRTH HISTORY: ANY MORE BIRTHS IN 2015-2018?<br>MORE BIRTHS IN 2015-2018 ☐ ↓ NO MORE BIRTHS IN 2015-2018 ☐		→ 601
502B	RECORD THE NAME AND BIRTH HISTORY NUMBER FROM 212 OF THE NEXT-TO-LAST CHILD BORN IN 2015-2018.<br>NAME OF NEXT-TO-LAST BIRTH ________	BIRTH HISTORY NUMBER . . . . ☐☐	
503B	CHECK 216 FOR CHILD:<br>LIVING ☐ ↓ DEAD ☐		→ 526B
504B	Do you have a card or other document where (NAME)'s vaccinations are written down?	YES, HAS ONLY A CARD . . . . 1<br>YES, HAS ONLY AN OTHER DOCUMENT . . . . . 2<br>YES, HAS CARD AND OTHER DOCUMENT . . . . . 3<br>NO, NO CARD AND NO OTHER DOCUMENT . . 4	1 → 507B<br><br>3 → 507B
505B	Did you ever have a vaccination card for (NAME)?	YES . . . . 1<br>NO 2	
506B	CHECK 504B:<br>CODE '2' CIRCLED ☐ ↓ CODE '4' CIRCLED ☐		→ 511B
507B	May I see the card or other document where (NAME)'s vaccinations are written down?	YES, ONLY CARD SEEN . . . . 1<br>YES, ONLY OTHER DOCUMENT SEEN . . . . 2<br>YES, CARD AND OTHER DOCUMENT SEEN . . 3<br>NO CARD AND NO OTHER DOCUMENT SEEN . . 4	4 → 511B

SECTION 5B. CHILD IMMUNIZATION (NEXT-TO-LAST BIRTH)

NO.	QUESTIONS AND FILTERS	CODING CATEGORIES	SKIP
	NAME OF NEXT-TO-LAST BIRTH ____________	BIRTH HISTORY NUMBER . . . . [ ][ ]	
508B	COPY DATES FROM THE CARD.<br>WRITE '44' IN 'DAY' COLUMN IF CARD SHOWS THAT A DOSE WAS GIVEN, BUT NO DATE IS RECORDED.		

	DAY	MONTH	YEAR
BCG			
HEPATITIS B AT BIRTH			
ORAL POLIO VACCINE (OPV) 0 (BIRTH DOSE)			
ORAL POLIO VACCINE (OPV) 1			
ORAL POLIO VACCINE (OPV) 2			
ORAL POLIO VACCINE (OPV) 3			
DPT-HEP.B-HIB (PENTAVALENT) 1			
DPT-HEP.B-HIB (PENTAVALENT) 2			
DPT-HEP.B-HIB (PENTAVALENT) 3			
PNEUMOCOCCAL 1			
PNEUMOCOCCAL 2			
PNEUMOCOCCAL 3			
INACTIVATED POLIO VIRUS (IPV)			
MEASLES 1			
MEASLES/MMR 2			
VITAMIN A (MOST RECENT)			

NO.	QUESTIONS AND FILTERS	CODING CATEGORIES	SKIP
509B	CHECK 508B: 'BCG' TO 'MEASLES/MMR 2' ALL RECORDED?<br>NO [ ]	YES [ ]	526B
510B	In addition to what is recorded on (this document/these documents), did (NAME) receive any other vaccinations, including vaccinations received in campaigns or immunization days or child health days?<br><br>RECORD 'YES' ONLY IF THE RESPONDENT MENTIONS AT LEAST ONE OF THE VACCINATIONS IN 508B THAT ARE NOT RECORDED AS HAVING BEEN GIVEN.	YES . . . . 1<br>(PROBE FOR VACCINATIONS AND WRITE '66' IN THE CORRESPONDING DAY COLUMN IN 508B THEN WRITE '00' IN THE CORRESPONDING DAY COLUMN FOR ALL VACCINATIONS NOT GIVEN)<br>(THEN SKIP TO 526B)<br><br>NO . . . . 2<br>DON'T KNOW . . . . 8<br>(WRITE '00' IN THE CORRESPONDING DAY COLUMN FOR ALL VACCINATIONS NOT GIVEN)<br>(THEN SKIP TO 526B)	

SECTION 5B. CHILD IMMUNIZATION (NEXT-TO-LAST BIRTH)

NO.	QUESTIONS AND FILTERS	CODING CATEGORIES	SKIP
	NAME OF NEXT-TO-LAST BIRTH ____________	BIRTH HISTORY NUMBER . . . . [ ][ ]	
511B	Did (NAME) ever receive any vaccinations to prevent (NAME) from getting diseases, including vaccinations received in campaigns or immunization days or child health days?	YES . . . . . 1<br>NO . . . . . 2<br>DON'T KNOW 8	2, 8 → 526B
512B	Has (NAME) ever received a BCG vaccination against tuberculosis, that is, an injection in the arm or shoulder that usually causes a scar?	YES . . . . . 1<br>NO . . . . . 2<br>DON'T KNOW 8	
513B	Within 24 hours after birth, did (NAME) receive a Hepatitis B vaccination, that is, an injection in the thigh to prevent Hepatitis B?	YES . . . . . 1<br>NO . . . . . 2<br>DON'T KNOW 8	
514B	Has (NAME) ever received oral polio vaccine, that is, about two drops in the mouth to prevent polio?	YES . . . . . 1<br>NO . . . . . 2<br>DON'T KNOW 8	2, 8 → 517B
515B	Did (NAME) receive the first oral polio vaccine in the first two weeks after birth or later?	FIRST TWO WEEKS . . . . . 1<br>LATER 2	
516B	How many times did (NAME) receive the oral polio vaccine?	NUMBER OF TIMES . . . . . [ ]	
517B	Has (NAME) ever received a pentavalent vaccination, that is, an injection given in the thigh sometimes at the same time as polio drops?	YES . . . . . 1<br>NO . . . . . 2<br>DON'T KNOW 8	2, 8 → 519B
518B	How many times did (NAME) receive the pentavalent vaccine?	NUMBER OF TIMES . . . . . [ ]	
519B	Has (NAME) ever received a pneumococcal vaccination, that is, an injection in the thigh to prevent pneumonia?	YES . . . . . 1<br>NO . . . . . 2<br>DON'T KNOW 8	2, 8 → 521B
520B	How many times did (NAME) receive the pneumococcal vaccine?	NUMBER OF TIMES . . . . . [ ]	
521B	Has (NAME) ever received an inactivated polio vaccine (IPV), that is, an injection in the thigh to prevent polio?	YES . . . . . 1<br>NO . . . . . 2<br>DON'T KNOW 8	
523B	Has (NAME) ever received a measles vaccination, that is, an injection in the arm to prevent measles?	YES . . . . . 1<br>NO . . . . . 2<br>DON'T KNOW 8	2, 8 → 526B
524B	How many times did (NAME) receive the measles vaccine?	NUMBER OF TIMES . . . . . [ ]	
526B	CHECK 215 IN BIRTH HISTORY: ANY MORE BIRTHS IN 2015-2018?<br>MORE BIRTHS IN 2015-2018 [ ] (GO TO 502B IN AN ADDITIONAL QUESTIONNAIRE)<br>NO MORE BIRTHS IN 2015-2018 [ ] → 601		→ 601

SECTION 6. CHILD HEALTH AND NUTRITION

601	CHECK 224:<br>ONE OR MORE BIRTHS IN 2013-2018 ☐ ↓<br>NO BIRTHS IN 2013-2018 ☐ → 648		
602	CHECK 215: RECORD THE BIRTH HISTORY NUMBER IN 603 AND THE NAME AND SURVIVAL STATUS IN 604 FOR EACH BIRTH IN 2013-2018. ASK THE QUESTIONS ABOUT ALL OF THESE BIRTHS. BEGIN WITH THE LAST BIRTH.<br>IF THERE ARE MORE THAN 2 BIRTHS, USE LAST COLUMN OF ADDITIONAL QUESTIONNAIRE(S).<br>Now I would like to ask some questions about your children born in the last five years. (We will talk about each separately.)		
603	BIRTH HISTORY NUMBER FROM 212 IN BIRTH HISTORY.	LAST BIRTH<br>BIRTH HISTORY NUMBER . . . . ☐☐	NEXT-TO-LAST BIRTH<br>BIRTH HISTORY NUMBER . . . . ☐☐
604	FROM 212 AND 216:	NAME ______<br>LIVING ☐ ↓<br>DEAD ☐ (SKIP TO 646)	NAME ______<br>LIVING ☐ ↓<br>DEAD ☐ (SKIP TO 646)
605	In the last six months, was (NAME) given a vitamin A dose like this?<br>SHOW COMMON TYPES OF CAPSULES.	YES . . . . 1<br>NO . . . . 2<br>DON'T KNOW 8	YES . . . . 1<br>NO . . . . 2<br>DON'T KNOW 8
606	In the last seven days, was (NAME) given iron pills, sprinkles with iron, or iron syrup like [this/any of these]?<br>SHOW COMMON TYPES OF PILLS/SPRINKLES/SYRUPS.	YES . . . . 1<br>NO . . . . 2<br>DON'T KNOW 8	YES . . . . 1<br>NO . . . . 2<br>DON'T KNOW 8
607	Was (NAME) given any medicine for deworming in the last six months?	YES . . . . 1<br>NO . . . . 2<br>DON'T KNOW 8	YES . . . . 1<br>NO . . . . 2<br>DON'T KNOW 8
608	Has (NAME) had diarrhea in the last 2 weeks?	YES . . . . 1<br>NO . . . . 2<br>(SKIP TO 618)<br>DON'T KNOW 8	YES . . . . 1<br>NO . . . . 2<br>(SKIP TO 618)<br>DON'T KNOW 8

NO.	QUESTIONS AND FILTERS	LAST BIRTH<br>NAME ______	NEXT-TO-LAST BIRTH<br>NAME ______
609	CHECK 469: CURRENTLY BREASTFEEDING?<br>YES ↓ / NO/ NOT ASKED ↓<br>a) (YES) Now I would like to know how much (NAME) was given to drink during the diarrhea including breastmilk. Was (NAME) given less than usual to drink, about the same amount, or more than usual to drink?<br>IF LESS, PROBE: Was (NAME) given much less than usual to drink or somewhat less?<br>b) (NO/ NOT ASKED) Now I would like to know how much (NAME) was given to drink during the diarrhea. Was (NAME) given less than usual to drink, about the same amount, or more than usual to drink?<br>IF LESS, PROBE: Was (NAME) given much less than usual to drink or somewhat less?	MUCH LESS . . . . 1<br>SOMEWHAT LESS . . . . 2<br>ABOUT THE SAME . . . . 3<br>MORE . . . . 4<br>NOTHING TO DRINK . . . . 5<br>DON'T KNOW 8	MUCH LESS . . . . 1<br>SOMEWHAT LESS . . . . 2<br>ABOUT THE SAME . . . . 3<br>MORE . . . . 4<br>NOTHING TO DRINK . . . . 5<br>DON'T KNOW 8
610	When (NAME) had diarrhea, was (NAME) given less than usual to eat, about the same amount, more than usual, or nothing to eat?<br>IF LESS, PROBE: Was (NAME) given much less than usual to eat or somewhat less?	MUCH LESS . . . . 1<br>SOMEWHAT LESS . . . . 2<br>ABOUT THE SAME . . . . 3<br>MORE . . . . 4<br>STOPPED FOOD . . . . 5<br>NEVER GAVE FOOD . . . . 6<br>DON'T KNOW 8	MUCH LESS . . . . 1<br>SOMEWHAT LESS . . . . 2<br>ABOUT THE SAME . . . . 3<br>MORE . . . . 4<br>STOPPED FOOD . . . . 5<br>NEVER GAVE FOOD . . . . 6<br>DON'T KNOW 8
611	Did you seek advice or treatment for the diarrhea from any source?	YES . . . . 1<br>NO . . . . 2<br>(SKIP TO 615)	YES . . . . 1<br>NO . . . . 2<br>(SKIP TO 615)

NO.	QUESTIONS AND FILTERS	LAST BIRTH<br>NAME ________	NEXT-TO-LAST BIRTH<br>NAME ________
612	Where did you seek advice or treatment?<br><br>Anywhere else?<br><br>PROBE TO IDENTIFY THE TYPE OF SOURCE.<br><br>IF UNABLE TO DETERMINE IF PUBLIC OR PRIVATE SECTOR, WRITE THE NAME OF THE PLACE(S).<br><br>________<br>(NAME OF PLACE(S))	**PUBLIC SECTOR**<br>GOVERNMENT HOSPITAL . . A<br>GOVERNMENT HEALTH CENTER . . . . B<br>GOVERNMENT HEALTH POST . . . . C<br>MOBILE CLINIC . . . . D<br>FIELDWORKER . . . . E<br>OTHER PUBLIC SECTOR ________ F<br>(SPECIFY)<br><br>**PRIVATE MEDICAL SECTOR**<br>PRIVATE HOSPITAL/ CLINIC . . . . G<br>PHARMACY . . . . H<br>CHEMIST/PMS . . . . I<br>PRIVATE DOCTOR . . . . J<br>MOBILE CLINIC . . . . K<br>FIELDWORKER . . . . L<br>OTHER PRIVATE MEDICAL SECTOR ________ M<br>(SPECIFY)<br><br>**OTHER SOURCE**<br>SHOP . . . . N<br>TRADITIONAL PRACTITIONER . . . . O<br>MARKET . . . . P<br>ITINERANT DRUG SELLER . . . . Q<br>COMMUNITY-ORIENTED RESOURCE PERSON . . R<br><br>OTHER ________ X<br>(SPECIFY)	**PUBLIC SECTOR**<br>GOVERNMENT HOSPITAL . . A<br>GOVERNMENT HEALTH CENTER . . . . B<br>GOVERNMENT HEALTH POST . . . . C<br>MOBILE CLINIC . . . . D<br>FIELDWORKER . . . . E<br>OTHER PUBLIC SECTOR ________ F<br>(SPECIFY)<br><br>**PRIVATE MEDICAL SECTOR**<br>PRIVATE HOSPITAL/ CLINIC . . . . G<br>PHARMACY . . . . H<br>CHEMIST/PMS . . . . I<br>PRIVATE DOCTOR . . . . J<br>MOBILE CLINIC . . . . K<br>FIELDWORKER . . . . L<br>OTHER PRIVATE MEDICAL SECTOR ________ M<br>(SPECIFY)<br><br>**OTHER SOURCE**<br>SHOP . . . . N<br>TRADITIONAL PRACTITIONER . . . . O<br>MARKET . . . . P<br>ITINERANT DRUG SELLER . . . . Q<br>COMMUNITY-ORIENTED RESOURCE PERSON . . R<br><br>OTHER ________ X<br>(SPECIFY)
613	CHECK 612:	TWO OR MORE CODES CIRCLED ☐<br>ONLY ONE CODE CIRCLED ☐ (SKIP TO 615)	TWO OR MORE CODES CIRCLED ☐<br>ONLY ONE CODE CIRCLED ☐ (SKIP TO 615)
614	Where did you first seek advice or treatment?<br><br>USE LETTER CODE FROM 612.	FIRST PLACE . . . . ☐	FIRST PLACE . . . . ☐

NO.	QUESTIONS AND FILTERS	LAST BIRTH<br>NAME ________	NEXT-TO-LAST BIRTH<br>NAME ________
615	Was (NAME) given any of the following at any time since (NAME) started having the diarrhea:<br>a) A fluid made from a special packet called CHI ORS, Emzorlyte, Orasure, Olpharm ORS etc.?<br>c) A government-recommended homemade fluid?<br>d) Zinc tablets or syrup?	YES NO DK<br>a) FLUID FROM ORS PACKET .. 1 2 8<br>c) HOMEMADE FLUID ..... 1 2 8<br>d) ZINC 1 2 8	YES NO DK<br>a) FLUID FROM ORS PACKET .. 1 2 8<br>c) HOMEMADE FLUID ..... 1 2 8<br>d) ZINC 1 2 8
616	CHECK 615:<br>ANY 'YES' ☐ / ALL 'NO' OR 'DK' ☐<br>a) Was anything else given to treat the diarrhea? / b) Was anything given to treat the diarrhea?	YES .... 1<br>NO .... 2 (SKIP TO 618)<br>DON'T KNOW 8	YES .... 1<br>NO .... 2 (SKIP TO 618)<br>DON'T KNOW 8
617	CHECK 615:<br>ANY 'YES' ☐ / ALL 'NO' OR 'DK' ☐<br>a) What else was given to treat the diarrhea? / b) What was given to treat the diarrhea?<br>Anything else? / Anything else?<br>RECORD ALL TREATMENTS GIVEN.	**PILL OR SYRUP**<br>ANTIBIOTIC .... A<br>ANTIMOTILITY .... B<br>OTHER (NOT ANTIBIOTIC OR ANTIMOTILITY) ..... C<br>UNKNOWN PILL OR SYRUP .... D<br>**INJECTION**<br>ANTIBIOTIC .... E<br>NON-ANTIBIOTIC .... F<br>UNKNOWN INJECTION .... G<br>(IV) INTRAVENOUS .... H<br>HOME REMEDY/ HERBAL MEDICINE .... I<br>OTHER ________ X (SPECIFY)	**PILL OR SYRUP**<br>ANTIBIOTIC .... A<br>ANTIMOTILITY .... B<br>OTHER (NOT ANTIBIOTIC OR ANTIMOTILITY) ..... C<br>UNKNOWN PILL OR SYRUP .... D<br>**INJECTION**<br>ANTIBIOTIC .... E<br>NON-ANTIBIOTIC .... F<br>UNKNOWN INJECTION .... G<br>(IV) INTRAVENOUS .... H<br>HOME REMEDY/ HERBAL MEDICINE .... I<br>OTHER ________ X (SPECIFY)
618	Has (NAME) been ill with a fever at any time in the last 2 weeks?	YES .... 1<br>NO .... 2 (SKIP TO 620)<br>DON'T KNOW 8	YES .... 1<br>NO .... 2 (SKIP TO 620)<br>DON'T KNOW 8
619	At any time during the illness, did (NAME) have blood taken from (NAME)'s finger or heel for testing?	YES .... 1<br>NO .... 2<br>DON'T KNOW 8	YES .... 1<br>NO .... 2<br>DON'T KNOW 8
620	Has (NAME) had an illness with a cough at any time in the last 2 weeks?	YES .... 1<br>NO .... 2<br>DON'T KNOW 8	YES .... 1<br>NO .... 2<br>DON'T KNOW 8
621	Has (NAME) had fast, short, rapid breaths or difficulty breathing at any time in the last 2 weeks?	YES .... 1<br>NO .... 2 (SKIP TO 623)<br>DON'T KNOW 8	YES .... 1<br>NO .... 2 (SKIP TO 623)<br>DON'T KNOW 8

NO.	QUESTIONS AND FILTERS	LAST BIRTH<br>NAME ______	NEXT-TO-LAST BIRTH<br>NAME ______
622	Was the fast or difficult breathing due to a problem in the chest or to a blocked or runny nose?	CHEST ONLY . . . . 1<br>NOSE ONLY . . . . 2<br>BOTH . . . . 3<br>OTHER ______ 6<br>(SPECIFY)<br>DON'T KNOW . . . . 8<br>(SKIP TO 624)	CHEST ONLY . . . . 1<br>NOSE ONLY . . . . 2<br>BOTH . . . . 3<br>OTHER ______ 6<br>(SPECIFY)<br>DON'T KNOW . . . . 8<br>(SKIP TO 624)
623	CHECK 618: HAD FEVER?	YES ☐ ↓<br>NO OR DK ☐ (SKIP TO 646)	YES ☐ ↓<br>NO OR DK ☐ (SKIP TO 646)
624	Did you seek advice or treatment for the illness from any source?	YES . . . . 1<br>NO . . . . 2<br>(SKIP TO 629)	YES . . . . 1<br>NO . . . . 2<br>(SKIP TO 629)
625	Where did you seek advice or treatment?<br><br>Anywhere else?<br><br>PROBE TO IDENTIFY THE TYPE OF SOURCE.<br><br>IF UNABLE TO DETERMINE IF PUBLIC OR PRIVATE SECTOR, WRITE THE NAME OF THE PLACE(S).<br><br>______<br>(NAME OF PLACE(S))	**PUBLIC SECTOR**<br>GOVERNMENT HOSPITAL . . A<br>GOVERNMENT HEALTH CENTER . . . . B<br>GOVERNMENT HEALTH POST . . . . C<br>MOBILE CLINIC . . . . D<br>FIELDWORKER/CHW . . . . . E<br>OTHER PUBLIC SECTOR ______ F<br>(SPECIFY)<br>**PRIVATE MEDICAL SECTOR**<br>PRIVATE HOSPITAL/ CLINIC . . . . G<br>PHARMACY . . . . H<br>CHEMIST/PMS . . . . I<br>PRIVATE DOCTOR . . . . J<br>MOBILE CLINIC . . . . K<br>FIELDWORKER/CHW . . . . . L<br>OTHER PRIVATE MEDICAL SECTOR ______ M<br>(SPECIFY)<br>**OTHER SOURCE**<br>SHOP . . . . N<br>TRADITIONAL PRACTITIONER . . . . O<br>MARKET . . . . P<br>ITINERANT DRUG SELLER . . . . Q<br>COMMUNITY-ORIENTED RESOURCE PERSON . . R<br>OTHER ______ X<br>(SPECIFY)	**PUBLIC SECTOR**<br>GOVERNMENT HOSPITAL . . A<br>GOVERNMENT HEALTH CENTER . . . . B<br>GOVERNMENT HEALTH POST . . . . C<br>MOBILE CLINIC . . . . D<br>FIELDWORKER/CHW . . . . . E<br>OTHER PUBLIC SECTOR ______ F<br>(SPECIFY)<br>**PRIVATE MEDICAL SECTOR**<br>PRIVATE HOSPITAL/ CLINIC . . . . G<br>PHARMACY . . . . H<br>CHEMIST/PMS . . . . I<br>PRIVATE DOCTOR . . . . J<br>MOBILE CLINIC . . . . K<br>FIELDWORKER/CHW . . . . . L<br>OTHER PRIVATE MEDICAL SECTOR ______ M<br>(SPECIFY)<br>**OTHER SOURCE**<br>SHOP . . . . N<br>TRADITIONAL PRACTITIONER . . . . O<br>MARKET . . . . P<br>ITINERANT DRUG SELLER . . . . Q<br>COMMUNITY-ORIENTED RESOURCE PERSON . . R<br>OTHER ______ X<br>(SPECIFY)
626	CHECK 625:	TWO OR MORE CODES CIRCLED ☐ ↓<br>ONLY ONE CODE CIRCLED ☐ (SKIP TO 628)	TWO OR MORE CODES CIRCLED ☐ ↓<br>ONLY ONE CODE CIRCLED ☐ (SKIP TO 628)

NO.	QUESTIONS AND FILTERS	LAST BIRTH<br>NAME ______	NEXT-TO-LAST BIRTH<br>NAME ______
627	Where did you first seek advice or treatment?<br><br>USE LETTER CODE FROM 625.	FIRST PLACE . . . . [ ]	FIRST PLACE . . . . [ ]
628	How many days after the illness began did you first seek advice or treatment for (NAME)?<br>IF THE SAME DAY RECORD '00'.	DAYS . . . . [ ][ ]	DAYS . . . . [ ][ ]
629	At any time during the illness, did (NAME) take any drugs for the illness?	YES . . . . 1<br>NO . . . . 2<br>(SKIP TO 646)<br>DON'T KNOW 8	YES . . . . 1<br>NO . . . . 2<br>(SKIP TO 646)<br>DON'T KNOW 8
630	What drugs did (NAME) take?<br><br>Any other drugs?<br><br>RECORD ALL MENTIONED.	**ANTIMALARIAL DRUGS**<br>ARTEMISININ<br>COMBINATION<br>THERAPY (ACT) . . . . . A<br>SP/FANSIDAR . . . . B<br>CHLOROQUINE . . . . C<br>AMODIAQUINE . . . . D<br>QUININE<br>PILLS . . . . E<br>INJECTION/IV . . . . F<br>ARTESUNATE<br>RECTAL . . . . G<br>INJECTION/IV . . . . H<br><br>OTHER ANTIMALARIAL<br>______ I<br>(SPECIFY)<br><br>**ANTIBIOTIC DRUGS**<br>PILL/SYRUP . . . . J<br>INJECTION/IV . . . . K<br><br>**OTHER DRUGS**<br>ASPIRIN . . . . L<br>PARACETAMOL . . . . M<br>IBUPROFEN . . . . N<br><br>OTHER ______ X<br>(SPECIFY)<br>DON'T KNOW . . . . Z	**ANTIMALARIAL DRUGS**<br>ARTEMISININ<br>COMBINATION<br>THERAPY (ACT) . . . . . A<br>SP/FANSIDAR . . . . B<br>CHLOROQUINE . . . . C<br>AMODIAQUINE . . . . D<br>QUININE<br>PILLS . . . . E<br>INJECTION/IV . . . . F<br>ARTESUNATE<br>RECTAL . . . . G<br>INJECTION/IV . . . . H<br><br>OTHER ANTIMALARIAL<br>______ I<br>(SPECIFY)<br><br>**ANTIBIOTIC DRUGS**<br>PILL/SYRUP . . . . J<br>INJECTION/IV . . . . K<br><br>**OTHER DRUGS**<br>ASPIRIN . . . . L<br>PARACETAMOL . . . . M<br>IBUPROFEN . . . . N<br><br>OTHER ______ X<br>(SPECIFY)<br>DON'T KNOW . . . . Z
631	CHECK 630:<br>ANY CODE A-I CIRCLED?	YES [ ] NO [ ]<br>(SKIP TO 646)	YES [ ] NO [ ]<br>(SKIP TO 646)

NO.	QUESTIONS AND FILTERS	LAST BIRTH<br>NAME ______	NEXT-TO-LAST BIRTH<br>NAME ______
632	CHECK 630:<br>ARTEMISININ COMBINATION THERAPY ('A') GIVEN	CODE 'A' CIRCLED ☐ ↓<br>CODE 'A' NOT CIRCLED ☐ (SKIP TO 646)	CODE 'A' CIRCLED ☐ ↓<br>CODE 'A' NOT CIRCLED ☐ (SKIP TO 646)
633	How long after the fever started did (NAME) first take an artemisinin combination therapy?	SAME DAY . . . . 0<br>NEXT DAY . . . . 1<br>TWO DAYS AFTER FEVER . . . . 2<br>THREE OR MORE DAYS AFTER FEVER . . . . 3<br>DON'T KNOW 8	SAME DAY . . . . 0<br>NEXT DAY . . . . 1<br>TWO DAYS AFTER FEVER . . . . 2<br>THREE OR MORE DAYS AFTER FEVER . . . . 3<br>DON'T KNOW 8
646		GO BACK TO 604 IN NEXT COLUMN; OR, IF NO MORE BIRTHS, GO TO 647.	GO TO 604 IN NEXT-TO-LAST COLUMN OF NEW QUESTIONNAIRE; OR, IF NO MORE BIRTHS, GO TO 647.

NO.	QUESTIONS AND FILTERS	CODING CATEGORIES	SKIP
647	CHECK 615(a) AND 615(b), ALL COLUMNS:<br>NO CHILD RECEIVED FLUID FROM ORS PACKET ☐ ↓	ANY CHILD RECEIVED FLUID FROM ORS PACKET ☐	→ 649
648	Have you ever heard of a special product ORS called CHI ORS, Emzorlyte, Orasure, Olpharm ORS etc. you can get for the treatment of diarrhea?	YES . . . . 1<br>NO 2	
649	CHECK 215 AND 218, ALL ROWS: NUMBER OF CHILDREN BORN IN 2016-2018 LIVING WITH THE RESPONDENT<br>ONE OR MORE ☐ ↓<br>____________________<br>(NAME OF YOUNGEST CHILD LIVING WITH HER)<br>↓	NONE ☐	→ 653A

NO.	QUESTIONS AND FILTERS	CODING CATEGORIES	SKIP
650	Now I would like to ask you about liquids or foods that (NAME FROM 649) had yesterday during the day or at night. I am interested in whether your child had the item I mention even if it was combined with other foods.<br>Did (NAME FROM 649) drink or eat:	YES NO DK	
	a) Plain water?	a) 1 2 8	
	b) Juice or juice drinks?	b) 1 2 8	
	c) Clear broth?	c) 1 2 8	
	d) Milk such as tinned, powdered, or fresh animal milk?<br>IF YES: How many times did (NAME) drink milk?<br>IF 7 OR MORE TIMES, RECORD '7'.	d) . . . . 1 2 8<br>NUMBER OF TIMES DRANK [ ]	
	e) Infant formula (Nan, SMA Gold, My Boy, Friso, Lactogen, Peak Milk 123, Cow and Gate, etc.)?<br>IF YES: How many times did (NAME) drink infant formula?<br>IF 7 OR MORE TIMES, RECORD '7'.	e) . . . . 1 2 8<br>NUMBER OF TIMES DRANK [ ]	
	f) Any other liquids?	f) 1 2 8	
	g) Yogurt?<br>IF YES: How many times did (NAME) eat yogurt?<br>IF 7 OR MORE TIMES, RECORD '7'.	g) . . . . 1 2 8<br>NUMBER OF TIMES ATE [ ]	
	h) Any commercially fortified baby food like Cerelac, Nutren, Frisolac H, Weatabix, etc.?	h) 1 2 8	
	i) Bread, rice, noodles, porridge, macaroni, tuwo shinkafa, semo, masa, pap or other foods made from grains (e.g. millet, sorghum, maize, wheat, oats, etc.)?	i) 1 2 8	
	j) Pumpkin, carrots, squash, or sweet potatoes that are yellow or orange inside?	j) 1 2 8	
	k) Irish/white potatoes, white yams, cassava, plantain, cocoyam, garri, fufu, lafun, or any other foods made from roots?	k) 1 2 8	
	l) Any dark green, leafy vegetables like spinach, pumpkin leaf, ugu, zogale (moringa), yakuwa, soko, ewedu, oha leaf, lansir, yadiya, rama, tafasa, etc.?	l) 1 2 8	
	m) Ripe mangoes, ripe pawpaw, ripe passion fruit, dorowa, or red palm-nuts etc. ?	m) 1 2 8	
	n) Any other fruits or vegetables (e.g. banana, watermelon, apples, green beans, avocados, tomatoes)?	n) 1 2 8	
	o) Liver, kidney, heart, or other organ meats?	o) 1 2 8	
	p) Any meat, such as beef, mutton, pork, lamb, bat, bush rat/bush meat, kundi, kilishi, camel, chicken, or duck?	p) 1 2 8	
	q) Eggs?	q) 1 2 8	
	r) Fresh or dried fish or shellfish?	r) 1 2 8	
	s) Any foods made from beans, peas, lentils, or nuts like moimoi, akara?	s) 1 2 8	
	t) Cheese or other food made from milk?	t) 1 2 8	
	u) Any other solid, semi-solid, or soft food?	u) 1 2 8	

NO.	QUESTIONS AND FILTERS	CODING CATEGORIES	SKIP
651	CHECK 650 (CATEGORIES 'g' THROUGH 'u'):<br>NOT A SINGLE 'YES' ☐ ↓ AT LEAST ONE 'YES' ☐		→ 653
652	Did (NAME FROM 649) eat any solid, semi-solid, or soft foods yesterday during the day or at night?<br><br>IF 'YES' PROBE: What kind of solid, semi-solid or soft foods did (NAME) eat?	YES . . . . . 1<br>(GO BACK TO 650 TO RECORD FOOD EATEN YESTERDAY)<br>(THEN CONTINUE TO 653)<br>NO 2	 <br><br>→ 653A
653	How many times did (NAME FROM 649) eat solid, semi-solid, or soft foods yesterday during the day or at night?<br><br>IF 7 OR MORE TIMES, RECORD '7'.	NUMBER OF TIMES . . . . . ☐<br>DON'T KNOW 8	

653A Now I would like to ask you about foods and drinks that you ate or drank yesterday during the day or night, whether you ate it at home or anywhere else.
I am interested in whether you had the food items I will mention even if they were combined with other foods. For example, if you had a soup made with carrots, potatoes and meat, you should reply "yes" for each of these ingredients when I read you the list. However, if you consumed only the broth of a soup, but not the meat or vegetable, do not say "yes" for the meat or vegetable.
As I ask you about foods and drinks, please think of foods and drinks you had as snacks or small meals as well as during any main meals. Please also remember foods you may have eaten while preparing meals or preparing food for others.
Please do not include any food used in a small amount for seasoning or condiments (like spices, herbs or crayfish powder). I will ask you about those foods separately.

Yesterday during the day or at night, did you eat or drink:

		YES	NO	DK
**Any foods made from grains, like:**<br>a) Wheat, maize, rice, sorghum (guinea corn or dawa), millet (gero/jero), acha, spaghetti (talia), macaroni, noodles, bread,	a)	1	2	8
**Any vegetables or roots that are orange coloured inside like:**<br>b) Squash that is orange inside, pumpkin, carrot, red sweet pepper (tatase), sweet potato that is orange inside (orange flesh sweet potatoes)?	b)	1	2	8
**Any white roots and tubers or plantains, like:**<br>c) Yam, water yam, cocoyam, potato, cassava, tigernut flour,	c)	1	2	8
**Any dark green leafy vegetables, like:**<br>d) Ugu, bitter leaf (ewuro/ onugbu), zogale (moringa), yakuwa (sorrel leaves), soko, ewedu/ayoyo, afang/okazi, sweet potato leaves, cassava leaves, cocoyam leaves, amaranthus/spinach (green/tete), water leaf, oha leaf, karkashi, kuka (baobab, luru), lansir, yadiya, rama, tafasa, kanya, cress, lettuce, yanrin (wild spinach), eku gogoro, eku petere, ilasa (young okro leaves), igbagba, ebolo, atama, editan, scent leaf (ntong/nchuawu/ arigbe/aluluisi), chaya (iyana paja), egg plant leaves?	d)	1	2	8
**Any fruits that are dark yellow or orange inside, like:**<br>e) Ripe pawpaw (gwanda/ibeppe/okwuru oru/bobo), ripe mango, ripe passion fruit, dorowa (locust bean fruit), red palm fruit, hog plum (tsadan gida, iyeye, ngulungu), ripe cantaloupe, musk melon, monkey cola (ndiya), bush mango fruit (ugili/ogbono/mbupauyo) ?	e)	1	2	8
**Any other fruits, like:**<br>f) Apple, banana, watermelon, tangerine, grapes, avocado pear, oranges, pears, dates (dabino), guava, pineapple, grapefruit, coconut, African cherry/African star apple (agbalumo/udara/udala), breadfruit, cashew fruit, soursop, golden melon, baobab fruit (ose/nonkuku), figs, shea fruit, doum palm fruit (goruba)?	f)	1	2	8
**Any other vegetables, like:**<br>g) Cabbage, cucumber, fresh tomato, onion, green beans, green pepper, okro, garden egg, green peas, boiled or roasted fresh corn, beets, mushroom, ujuju?	g)	1	2	8

SECTION 6. CHILD HEALTH AND NUTRITION

NO.	QUESTIONS AND FILTERS	CODING CATEGORIES	SKIP
	**Any meat made from animal organs, like:**<br>h) Liver, kidney, heart, gizzard?	h) 1 2 8	
	**Any other types of meat or poultry, like:**<br>i) Meat, chicken, and other bush meat/bird, kundi, kilishi, dambu nama, ponmo (cow skin)?	i) 1 2 8	
	**Any eggs**<br>j) Any eggs?	j) 1 2 8	
	**Any fish or seafood, whether fresh or dried, like:**<br>k) Fish, crab, lobster, cray fish, shrimp, stock fish (okporoko)?	k) 1 2 8	
	**Any beans or peas, like:**<br>l) Beans, chickpeas, soya beans, bambara nut (ebi-abo)?	l) 1 2 8	
	**Any nuts or seeds, like:**<br>m) Melon seed (egusi), pumpkin seeds (mkpuru anyu/ugboguru), walnuts, groundnuts, shea nut, cashew nuts, bush mango seeds (ogbono)?	m) 1 2 8	
	**Any milk or milk products, like:**<br>n) Milk, sour milk (nono), yogurt, cheese (wara)?	n) 1 2 8	
	**Any insects and other small protein foods, like:**<br>o) Winged termite (aku, esunsun, chinge, ako), cricket, snails (igbin/ejuna), sea snails (nkonko/isawuru), periwinkle, ogongo, akankwu, African palm weevil larva (monini/ekuku/okuka/uton/ [illegible]	o) 1 2 8	
	**Any red palm oil, like:**<br>p) Foods made with red palm oil, red palm nut, or red palm nut pulp sauces?	p) 1 2 8	
	**Any other oils and fats, like:**<br>q) Oil, fats or butter added to food or used for cooking, including vegetable oil, any other type of oil, butter, margarine (blue band), mayonnaise, shea butter, manshanu, extracted oils from nuts, fruits and seeds, and all animal fat?	q) 1 2 8	
	**Any savoury and fried snacks, like:**<br>r) Crisps and chips, fried dough (puffpuff), other fried snacks (chinchin, kulikuli, donkuwa)?	r) 1 2 8	
	**Any sweets, like:**<br>s) Chocolates, candies, cookies/sweet biscuits and cakes, sweet pastries or ice cream?	s) 1 2 8	
	**Any sugar-sweetened beverages, like:**<br>t) Sweetened fruit juices and “juice drinks”, soft drinks/fizzy drinks, chocolate drinks(milo), malt drinks, sweet tea or coffee with	t) 1 2 8	
	**Any condiments and seasonings, like:**<br>u) Salt, Maggi, black pepper, alligator pepper, yaji, bay leaf, uziza, scent leaves, utazi, thyme, curry, ginger, garlic, cloves (kanafuru), tomato paste, ehuru, uyayak, uda, crayfish powder, locust bean used as seasoning, ogiri?	u) 1 2 8	
	**Any other beverages and foods, like:**<br>v) Coffee or tea if unsweetened, alcohol, clear broth, soup broth, olives, pickled cucumbers, herbal beverages/infusions (zobo), kunun aya, kunun dawa, water, kolanut, bitter kola?	v) 1 2 8	

NO.	QUESTIONS AND FILTERS	CODING CATEGORIES	SKIP
653B	CHECK 215 AND 218, ALL ROWS: NUMBER OF CHILDREN BORN IN 2016-2018 LIVING WITH THE RESPONDENT<br>ONE OR MORE ☐ ↓<br>NONE ☐<br>____________________<br>(NAME OF YOUNGEST CHILD LIVING WITH HER) ↓		NONE → 701
654	The last time (NAME FROM 649) passed stools, what was done to dispose of the stools?	CHILD USED TOILET OR LATRINE . . . . 01<br>PUT/RINSED<br>INTO TOILET OR LATRI . . . . 02<br>PUT/RINSED<br>INTO DRAIN OR DITCH . . . . 03<br>THROWN INTO GARBA(. . . . . 04<br>BURIED . . . . 05<br>LEFT IN THE OPI . . . . 06<br><br>OTHER ____________________ 96<br>(SPECIFY)	

SECTION 7. MARRIAGE AND SEXUAL ACTIVITY

NO.	QUESTIONS AND FILTERS	CODING CATEGORIES	SKIP
701	Are you currently married or living together with a man as if married?	YES, CURRENTLY MARRIED . . . . . 1<br>YES, LIVING WITH A MAN . . . . . 2<br>NO, NOT IN UNION 3	1, 2 → 704
702	Have you ever been married or lived together with a man as if married?	YES, FORMERLY MARRIED . . . . . 1<br>YES, LIVED WITH A MAN . . . . . 2<br>NO 3	3 → 712
703	What is your marital status now: are you widowed, divorced, or separated?	WIDOWED . . . . . 1<br>DIVORCED . . . . . 2<br>SEPARATED 3	1, 2, 3 → 709
704	Is your (husband/partner) living with you now or is he staying elsewhere?	LIVING WITH HER . . . . . 1<br>STAYING ELSEWHERE 2	
705	RECORD THE HUSBAND'S/PARTNER'S NAME AND LINE NUMBER FROM THE HOUSEHOLD QUESTIONNAIRE. IF HE IS NOT LISTED IN THE HOUSEHOLD, RECORD '00'.	NAME ______________<br>LINE NO. . . . . . [ ][ ]	
706	Does your (husband/partner) have other wives or does he live with other women as if married?	YES . . . . . 1<br>NO . . . . . 2<br>DON'T KNOW 8	2, 8 → 709
707	Including yourself, in total, how many wives or live-in partners does he have?	TOTAL NUMBER OF WIVES AND LIVE-IN PARTNERS . . . . . [ ][ ]<br>DON'T KNOW 98	
708	Are you the first, second, … wife?	RANK . . . . . [ ][ ]	
709	Have you been married or lived with a man only once or more than once?	ONLY ONCE . . . . . 1<br>MORE THAN ONCE 2	
710	CHECK 709:<br>MARRIED/ LIVED WITH A MAN ONLY ONCE [ ]<br>a) In what month and year did you start living with your (husband/partner)?<br>MARRIED/ LIVED WITH A MAN MORE THAN ONCE [ ]<br>b) Now I would like to ask about your first (husband/partner). In what month and year did you start living with him?	MONTH . . . . . [ ][ ]<br>DON'T KNOW MONTH . . . . . 98<br>YEAR . . . . . [ ][ ][ ][ ]<br>DON'T KNOW YEAR 9998	→ 712
711	How old were you when you first started living with him?	AGE . . . . . [ ][ ]	

NO.	QUESTIONS AND FILTERS	CODING CATEGORIES	SKIP
712	**CHECK FOR PRESENCE OF OTHERS. BEFORE CONTINUING, MAKE EVERY EFFORT TO ENSURE PRIVACY.**		
713	Now I would like to ask some questions about sexual activity in order to gain a better understanding of some important life issues. Let me assure you again that your answers are completely confidential and will not be told to anyone. If we should come to any question that you don't want to answer, just let me know and we will go to the next question. How old were you when you had sexual intercourse for the very first time?	NEVER HAD SEXUAL INTERCOURSE . . . . 00<br>AGE IN YEARS . . . . [ ][ ]	00 → 731
714	I would like to ask you about your recent sexual activity. When was the last time you had sexual intercourse?<br><br>IF LESS THAN 12 MONTHS, ANSWER MUST BE RECORDED IN DAYS, WEEKS OR MONTHS. IF 12 MONTHS (ONE YEAR) OR MORE, ANSWER MUST BE RECORDED IN YEARS.	DAYS AGO . . . . 1 [ ][ ]<br>WEEKS AGO . . . . 2 [ ][ ]<br>MONTHS AGO . . . . 3 [ ][ ]<br>YEARS AGO . . . . 4 [ ][ ]	1–3 → 716<br>4 → 727

SECTION 7. MARRIAGE AND SEXUAL ACTIVITY

		LAST SEXUAL PARTNER	SECOND-TO-LAST SEXUAL PARTNER	THIRD-TO-LAST SEXUAL PARTNER
715	When was the last time you had sexual intercourse with this person?		DAYS AGO . . 1 [ ][ ]<br>WEEKS AGO . . 2 [ ][ ]<br>MONTHS AGO . . 3 [ ][ ]	DAYS AGO . . 1 [ ][ ]<br>WEEKS AGO . . 2 [ ][ ]<br>MONTHS AGO . . 3 [ ][ ]
716	The last time you had sexual intercourse with this person, was a condom used?	YES . . . . 1<br>NO . . . . 2<br>(SKIP TO 718)	YES . . . . 1<br>NO . . . . 2<br>(SKIP TO 718)	YES . . . . 1<br>NO . . . . 2<br>(SKIP TO 718)
717	Was a condom used every time you had sexual intercourse with this person in the last 12 months?	YES . . . . 1<br>NO 2	YES . . . . 1<br>NO 2	YES . . . . 1<br>NO 2
718	What was your relationship to this person with whom you had sexual intercourse?<br><br>IF BOYFRIEND: Were you living together as if married?<br><br>IF YES, RECORD '2'.<br>IF NO, RECORD '3'.	HUSBAND . . . . 1<br>LIVE-IN PARTNER . . . . . 2<br>BOYFRIEND NOT LIVING WITH RESPONDENT . . . . . 3<br>CASUAL ACQUAINTANCE . . 4<br>CLIENT/SEX WORKER . . 5<br>OTHER ______ 6<br>(SPECIFY)	HUSBAND . . . . 1<br>LIVE-IN PARTNER . . . . . 2<br>BOYFRIEND NOT LIVING WITH RESPONDENT . . . . . 3<br>CASUAL ACQUAINTANCE . . 4<br>CLIENT/SEX WORKER . . 5<br>OTHER ______ 6<br>(SPECIFY)	HUSBAND . . . . 1<br>LIVE-IN PARTNER . . . . . 2<br>BOYFRIEND NOT LIVING WITH RESPONDENT . . . . . 3<br>CASUAL ACQUAINTANCE . . 4<br>CLIENT/SEX WORKER . . 5<br>OTHER ______ 6<br>(SPECIFY)
719	How long ago did you first have sexual intercourse with this person?	DAYS AGO . . 1 [ ][ ]<br>WEEKS AGO . . 2 [ ][ ]<br>MONTHS AGO . . 3 [ ][ ]<br>YEARS AGO . . 4 [ ][ ]	DAYS AGO . . 1 [ ][ ]<br>WEEKS AGO . . 2 [ ][ ]<br>MONTHS AGO . . 3 [ ][ ]<br>YEARS AGO . . 4 [ ][ ]	DAYS AGO . . 1 [ ][ ]<br>WEEKS AGO . . 2 [ ][ ]<br>MONTHS AGO . . 3 [ ][ ]<br>YEARS AGO . . 4 [ ][ ]
720	How many times during the last 12 months did you have sexual intercourse with this person?<br>IF NON-NUMERIC ANSWER, PROBE TO GET AN ESTIMATE. IF NUMBER OF TIMES IS 95 OR MORE, RECORD '95'.	NUMBER OF TIMES . . . . . [ ][ ]	NUMBER OF TIMES . . . . . [ ][ ]	NUMBER OF TIMES . . . . . [ ][ ]
721	How old is this person?	AGE OF PARTNER [ ][ ]<br><br>DON'T KNOW 98	AGE OF PARTNER [ ][ ]<br><br>DON'T KNOW 98	AGE OF PARTNER [ ][ ]<br><br>DON'T KNOW 98
722	Apart from this person, have you had sexual intercourse with any other person in the last 12 months?	YES . . . . 1<br>(GO BACK TO 715 IN NEXT COLUMN)<br>NO . . . . 2<br>(SKIP TO 724)	YES . . . . 1<br>(GO BACK TO 715 IN NEXT COLUMN)<br>NO . . . . 2<br>(SKIP TO 724)	
723	In total, with how many different people have you had sexual intercourse in the last 12 months?<br>IF NON-NUMERIC ANSWER, PROBE TO GET AN ESTIMATE. IF NUMBER OF PARTNERS IS 95 OR MORE, RECORD '95'.			NUMBER OF PARTNERS LAST 12 MONTHS . . [ ][ ]<br><br>DON'T KNOW 98

NO.	QUESTIONS AND FILTERS	CODING CATEGORIES	SKIP
724	CHECK 106:<br>AGE 15-24 ☐ ↓	AGE 25-49 ☐	727
725	CHECK 701:<br>NOT IN A UNION ☐ ↓	CURRENTLY MARRIED/ LIVING WITH A MAN ☐	727
726	In the past 12 months have you had sex or been sexually involved with anyone because he gave you or told you he would give you gifts, cash, or anything else?	YES . . . . 1<br>NO 2	
727	In total, with how many different people have you had sexual intercourse in your lifetime?<br><br>IF NON-NUMERIC ANSWER, PROBE TO GET AN ESTIMATE. IF NUMBER OF PARTNERS IS 95 OR MORE, RECORD '95'.	NUMBER OF PARTNERS IN LIFETIME . . . . ☐☐<br><br>DON'T KNOW 98	
728	CHECK 716, MOST RECENT PARTNER (FIRST COLUMN):<br>YES, CONDOM USED ☐ ↓	NO, CONDOM NOT USED ☐<br>NOT ASKED ☐	731<br>731
729	You told me that a condom was used the last time you had sex. What is the brand name of the condom used at that time?<br><br>IF BRAND NOT KNOWN, ASK TO SEE THE PACKAGE.	**MALE CONDOMS**<br>GOLD CIRCLE . . . . 01<br>DUREX . . . . 02<br>ROUGH RIDER . . . . 03<br>TWIN LOTUS . . . . 04<br>PLAIN CONDOMS . . . . 05<br>GO FLEX . . . . 06<br><br>OTHER ______________ 96<br>(SPECIFY)<br>DON'T KNOW 98	

NO.	QUESTIONS AND FILTERS	CODING CATEGORIES	SKIP
730	From where did you obtain the condom the last time?<br><br>PROBE TO IDENTIFY TYPE OF SOURCE.<br><br>IF UNABLE TO DETERMINE IF PUBLIC OR PRIVATE SECTOR, WRITE THE NAME OF THE PLACE.<br><br>________________<br>(NAME OF PLACE)	**PUBLIC SECTOR**<br>GOVERNMENT HOSPITAL . . . . 11<br>GOVERNMENT HEALTH CENTER . . . . 12<br>FAMILY PLANNING CLINIC . . . . 13<br>MOBILE CLINIC . . . . 14<br>FIELDWORKER . . . . 15<br>OTHER PUBLIC SECTOR<br>________________ 16<br>(SPECIFY)<br><br>**PRIVATE MEDICAL SECTOR**<br>PRIVATE HOSPITAL/CLINIC . . . . 21<br>PHARMACY . . . . 22<br>CHEMIST/PMS . . . . 23<br>PRIVATE DOCTOR . . . . 24<br>MOBILE CLINIC . . . . 25<br>FIELDWORKER . . . . 26<br>OTHER PRIVATE MEDICAL SECTOR<br>________________ 27<br>(SPECIFY)<br><br>**OTHER SOURCE**<br>SHOP . . . . 31<br>CHURCH . . . . 32<br>FRIEND/RELATIVE . . . . 33<br>NGO . . . . 34<br><br>OTHER ________________ 96<br>(SPECIFY)<br>DON'T KNOW 98	
731	PRESENCE OF OTHERS DURING THIS SECTION.	YES NO<br>CHILDREN <10 . . . . 1 2<br>MALE ADULTS . . . . 1 2<br>FEMALE ADULTS 1 2	

SECTION 8. FERTILITY PREFERENCES

NO.	QUESTIONS AND FILTERS	CODING CATEGORIES	SKIP
801	CHECK 304:<br>NEITHER STERILIZED ☐ ↓	HE OR SHE STERILIZED ☐	→ 813
802	CHECK 226:<br>PREGNANT ☐ ↓	NOT PREGNANT OR UNSURE ☐	→ 804
803	Now I have some questions about the future. After the child you are expecting now, would you like to have another child, or would you prefer not to have any more children?	HAVE ANOTHER CHILD . . . . 1<br>NO MORE . . . . 2<br>UNDECIDED/DON'T KNOW 8	→ 805<br>} → 812
804	Now I have some questions about the future. Would you like to have (a/another) child, or would you prefer not to have any (more) children?	HAVE (A/ANOTHER) CHILD . . . . 1<br>NO MORE/NONE . . . . 2<br>SAYS SHE CAN'T GET PREGNANT . . . . 3<br>UNDECIDED/DON'T KNOW 8	 → 807<br>→ 813<br>→ 811
805	CHECK 226:<br>NOT PREGNANT OR UNSURE ☐ ↓<br>a) How long would you like to wait from now before the birth of (a/another) child?<br>PREGNANT ☐ ↓<br>b) After the birth of the child you are expecting now, how long would you like to wait before the birth of another child?	MONTHS . . . . 1 ☐☐<br>YEARS . . . . 2 ☐☐<br>SOON/NOW . . . . .993<br>SAYS SHE CAN'T GET PREGNANT . . . . 994<br>AFTER MARRIAGE . . . . .995<br>OTHER ______________________ 996<br>(SPECIFY)<br>DON'T KNOW 998	 <br>→ 811<br>→ 813<br>} → 811
806	CHECK 226:<br>NOT PREGNANT OR UNSURE ☐ ↓	PREGNANT ☐	→ 812
807	CHECK 303: USING A CONTRACEPTIVE METHOD?<br>NOT CURRENTLY USING ☐ ↓	CURRENTLY USING ☐	→ 813
808	CHECK 805:<br>'24' OR MORE MONTHS OR '02' OR MORE YEARS ☐ ↓<br>NOT ASKED ☐ ↓	'00-23' MONTHS OR '00-01' YEAR ☐	→ 812
809	CHECK 714:<br>DAYS, WEEKS OR MONTHS AGO ☐ ↓	YEARS AGO ☐<br>NOT ASKED ☐	→ 811<br>→ 811

SECTION 8. FERTILITY PREFERENCES

NO.	QUESTIONS AND FILTERS	CODING CATEGORIES	SKIP
810	CHECK 804:<br>WANTS TO HAVE A/ANOTHER CHILD ☐ ¦ WANTS NO MORE/ NONE ☐<br>a) You have said that you do not want (a/another) child soon. Can you tell me why you are not using a method to prevent pregnancy?<br>Any other reason?<br>b) You have said that you do not want any (more) children. Can you tell me why you are not using a method to prevent pregnancy?<br>Any other reason?<br>RECORD ALL REASONS MENTIONED.	NOT MARRIED . . . . . A<br>**FERTILITY-RELATED REASONS**<br>NOT HAVING SEX . . . . . B<br>INFREQUENT SEX . . . . . C<br>MENOPAUSAL/HYSTERECTOMY . . . . . D<br>CAN'T GET PREGNANT . . . . . E<br>NOT MENSTRUATED SINCE LAST BIRTH . . . . . F<br>BREASTFEEDING . . . . . G<br>UP TO GOD/FATALISTIC . . . . . H<br>**OPPOSITION TO USE**<br>RESPONDENT OPPOSED . . . . . I<br>HUSBAND/PARTNER OPPOSED . . . . . J<br>OTHERS OPPOSED . . . . . K<br>RELIGIOUS PROHIBITION . . . . . L<br>**LACK OF KNOWLEDGE**<br>KNOWS NO METHOD . . . . . M<br>KNOWS NO SOURCE . . . . . N<br>**METHOD-RELATED REASONS**<br>SIDE EFFECTS/HEALTH CONCERNS . . . . . O<br>LACK OF ACCESS/TOO FAR . . . . . P<br>COSTS TOO MUCH . . . . . Q<br>PREFERRED METHOD NOT AVAILABLE . . . . . R<br>NO METHOD AVAILABLE . . . . . S<br>INCONVENIENT TO USE . . . . . T<br>INTERFERES WITH BODY'S NORMAL PROCESSES . . . . . U<br>OTHER ______ (SPECIFY) X<br>DON'T KNOW . . . . . Z	
811	CHECK 303: USING A CONTRACEPTIVE METHOD?<br>NOT ASKED ☐ NO, NOT CURRENTLY USING ☐ YES, CURRENTLY USING ☐ → 813		→ 813
812	Do you think you will use a contraceptive method to delay or avoid pregnancy at any time in the future?	YES . . . . . 1<br>NO . . . . . 2<br>DON'T KNOW 8	
813	CHECK 216:<br>HAS LIVING CHILDREN ☐ ¦ NO LIVING CHILDREN ☐<br>a) If you could go back to the time you did not have any children and could choose exactly the number of children to have in your whole life, how many would that be?<br>b) If you could choose exactly the number of children to have in your whole life, how many would that be?<br>PROBE FOR A NUMERIC RESPONSE.	NONE . . . . . 00<br>NUMBER . . . . . [__][__]<br>OTHER ______ (SPECIFY) 96	00 → 815<br>96 → 815
814	How many of these children would you like to be boys, how many would you like to be girls and for how many would it not matter if it's a boy or a girl?	NUMBER . . BOYS [__][__] GIRLS [__][__] EITHER [__][__]<br>OTHER ______ (SPECIFY) 96	

SECTION 8. FERTILITY PREFERENCES

NO.	QUESTIONS AND FILTERS	CODING CATEGORIES	SKIP
815	In the last few months have you:<br>a) Heard about family planning on the radio?<br>b) Seen anything about family planning on the television?<br>c) Read about family planning in a newspaper or magazine?<br>d) Received a voice or text message about family planning on a mobile phone?<br>e) Read/heard from social media (facebook, twitter, etc.)?<br>f) Read about family planning in a poster?<br>g) Read about family planning in a leaflet or brochures?<br>h) Heard about family planning from town crier?<br>i) Heard about family planning from mobile public announcement?	YES NO<br>a) RADIO . . . . 1 2<br>b) TELEVISION . . . . 1 2<br>c) NEWSPAPER OR MAGAZINE . . . . . 1 2<br>d) MOBILE PHONE . . . . 1 2<br>e) SOCIAL MEDIA . . . . 1 2<br>f) POSTER . . . . 1 2<br>g) LEAFLET OR BROCHURE . . . . . 1 2<br>h) TOWN CRIER . . . . 1 2<br>i) MOBILE PUBLIC ANNOUNCEMEN 1 2	
815A	CHECK 815:<br>AT LEAST ONE `YES' (HAS HEARD OR READ MESSAGE) ☐ ↓<br>NOT A SINGLE `YES' (HAS NOT HEARD OR READ MESSAGE) ☐ → 817		817
816	Please tell me which family planning messages you have heard or seen in the past few months?<br><br>PROBE: Any others?	AS FOR ME AND MY PARTNER WE `DEY KAMPE' WITH FEMALE CONDOM . . . . . A<br>UNSPACED CHILDREN MAKES THE GOING TOUGH FOR THE LOVE OF YOUR FAMILY, GO FOR CHILD SPACING TODAY . . . . . B<br>WELL-SPACED CHILDREN ARE EVERY PARENT'S JOY . . . . . C<br>IT'S NOT TOO LATE TO PREVENT UNWANTED PREGNANCY . . . . . D<br>WHY IS YOUR WIFE LOOKING SO GOO . . . . . E<br>OTHER ______ (SPECIFY) X	
817	CHECK 701:<br>YES, CURRENTLY MARRIED ☐ ↓<br>YES, LIVING WITH A MAN ☐ ↓<br>NO, NOT IN A UNION ☐ → 901		901
818	CHECK 303: USING A CONTRACEPTIVE METHOD?<br>CURRENTLY USING ☐ ↓<br>NOT CURRENTLY USING ☐ → 820<br>NOT ASKED ☐ → 822		820<br>822
819	Would you say that using contraception is mainly your decision, mainly your (husband's/partner's) decision, or did you both decide together?	MAINLY RESPONDENT . . . . . 1<br>MAINLY HUSBAND/PARTNER . . . . . 2<br>JOINT DECISION . . . . . 3<br>OTHER ______ (SPECIFY) 6	821
820	Would you say that not using contraception is mainly your decision, mainly your (husband's/partner's) decision, or did you both decide together?	MAINLY RESPONDENT . . . . . 1<br>MAINLY HUSBAND/PARTNER . . . . . 2<br>JOINT DECISION . . . . . 3<br>OTHER ______ (SPECIFY) 6	
821	CHECK 304:<br>NEITHER ARE STERILIZED ☐ ↓<br>HE OR SHE ARE STERILIZED ☐ → 901		901
822	Does your (husband/partner) want the same number of children that you want, or does he want more or fewer than you want?	SAME NUMBER . . . . . 1<br>MORE CHILDREN . . . . . 2<br>FEWER CHILDREN . . . . . 3<br>DON'T KNOW 8	

SECTION 9. HUSBAND'S BACKGROUND AND WOMAN'S WORK

NO.	QUESTIONS AND FILTERS	CODING CATEGORIES	SKIP
901	CHECK 701:<br>CURRENTLY MARRIED/ LIVING WITH A MAN ☐	NOT IN UNION ☐	→ 909
902	How old was your (husband/partner) on his last birthday?	AGE IN COMPLETED YEARS . . . . ☐☐	
903	Did your (husband/partner) ever attend school?	YES . . . . . . 1<br>NO 2	 → 906
904	What was the highest level of school he attended: primary, secondary, or higher?	PRIMARY . . . . . . 1<br>SECONDARY . . . . . . 2<br>HIGHER . . . . . . 3<br>DON'T KNOW 8	 <br><br>→ 906
905	What was the highest Class/Year he completed at that level?<br>IF COMPLETED LESS THAN ONE YEAR AT THAT LEVEL, RECORD '00'.	CLASS/YEAR . . . . ☐☐<br>DON'T KNOW 98	
906	Has your (husband/partner) done any work in the last 7 days?	YES . . . . . . 1<br>NO . . . . . . 2<br>DON'T KNOW 8	→ 908
907	Has your (husband/partner) done any work in the last 12 months?	YES . . . . . . 1<br>NO . . . . . . 2<br>DON'T KNOW 8	 → 909 (NO, DON'T KNOW)
908	What is your (husband's/partner's) occupation? That is, what kind of work does he mainly do?	____________<br>____________ ☐☐<br>____________	
909	Aside from your own housework, have you done any work in the last seven days?	YES . . . . . . 1<br>NO 2	→ 913
910	As you know, some women take up jobs for which they are paid in cash or kind. Others sell things, have a small business or work on the family farm or in the family business. In the last seven days, have you done any of these things or any other work?	YES . . . . . . 1<br>NO 2	→ 913
911	Although you did not work in the last seven days, do you have any job or business from which you were absent for leave, illness, vacation, maternity leave, or any other such reason?	YES . . . . . . 1<br>NO 2	→ 913
912	Have you done any work in the last 12 months?	YES . . . . . . 1<br>NO 2	 → 917
913	What is your occupation? That is, what kind of work do you mainly do?	____________<br>____________ ☐☐<br>____________	

NO.	QUESTIONS AND FILTERS	CODING CATEGORIES	SKIP
914	Do you do this work for a member of your family, for someone else, or are you self-employed?	FOR FAMILY MEMBER .... 1<br>FOR SOMEONE ELSE .... 2<br>SELF-EMPLOYED 3	
915	Do you usually work throughout the year, or do you work seasonally, or only once in a while?	THROUGHOUT THE YEAR .... 1<br>SEASONALLY/PART OF THE YEAR .... 2<br>ONCE IN A WHILE 3	
916	Are you paid in cash or kind for this work or are you not paid at all?	CASH ONLY .... 1<br>CASH AND KIND .... 2<br>IN KIND ONLY .... 3<br>NOT PAID 4	
917	CHECK 701:<br>CURRENTLY MARRIED/LIVING WITH A MAN ☐	NOT IN UNION ☐	→ 925
918	CHECK 916:<br>CODE '1' OR '2' CIRCLED ☐	OTHER ☐	→ 921
919	Who usually decides how the money you earn will be used: you, your (husband/partner), or you and your (husband/partner) jointly?	RESPONDENT .... 1<br>HUSBAND/PARTNER .... 2<br>RESPONDENT AND HUSBAND/PARTNER JOINTLY .... 3<br>OTHER ________ 6<br>(SPECIFY)	
920	Would you say that the money that you earn is more than what your (husband/partner) earns, less than what he earns, or about the same?	MORE THAN HIM .... 1<br>LESS THAN HIM .... 2<br>ABOUT THE SAME .... 3<br>HUSBAND/PARTNER HAS NO EARNINGS .... 4<br>DON'T KNOW 8	4 → 922
921	Who usually decides how your (husband's/partner's) earnings will be used: you, your (husband/partner), or you and your (husband/partner) jointly?	RESPONDENT .... 1<br>HUSBAND/PARTNER .... 2<br>RESPONDENT AND HUSBAND/PARTNER JOINTLY .... 3<br>HUSBAND/PARTNER HAS NO EARNINGS .... 4<br>OTHER ________ 6<br>(SPECIFY)	
922	Who usually makes decisions about health care for yourself: you, your (husband/partner), you and your (husband/partner) jointly, or someone else?	RESPONDENT .... 1<br>HUSBAND/PARTNER .... 2<br>RESPONDENT AND HUSBAND/PARTNER JOINTLY .... 3<br>SOMEONE ELSE .... 4<br>OTHER 6	
923	Who usually makes decisions about making major household purchases?	RESPONDENT .... 1<br>HUSBAND/PARTNER .... 2<br>RESPONDENT AND HUSBAND/PARTNER JOINTLY .... 3<br>SOMEONE ELSE .... 4<br>OTHER 6	

NO.	QUESTIONS AND FILTERS	CODING CATEGORIES	SKIP
924	Who usually makes decisions about visits to your family or relatives?	RESPONDENT . . . . 1<br>HUSBAND/PARTNER . . . . 2<br>RESPONDENT AND HUSBAND/PARTNER JOINTLY . . . . 3<br>SOMEONE ELSE . . . . 4<br>OTHER 6	
925	Do you own this or any other house either alone or jointly with someone else?	ALONE ONLY . . . . 1<br>JOINTLY ONLY . . . . 2<br>BOTH ALONE AND JOINTLY . . . . 3<br>DOES NOT OWN 4	4 → 928
926	Do you have a title deed for any house you own?	YES . . . . 1<br>NO . . . . 2<br>DON'T KNOW 8	2, 8 → 928
927	Is your name on the title deed?	YES . . . . 1<br>NO . . . . 2<br>DON'T KNOW 8	
928	Do you own any agricultural or non-agricultural land either alone or jointly with someone else?	ALONE ONLY . . . . 1<br>JOINTLY ONLY . . . . 2<br>BOTH ALONE AND JOINTLY . . . . 3<br>DOES NOT OWN 4	4 → 931
929	Do you have a title deed for any land you own?	YES . . . . 1<br>NO . . . . 2<br>DON'T KNOW 8	2, 8 → 931
930	Is your name on the title deed?	YES . . . . 1<br>NO . . . . 2<br>DON'T KNOW 8	

NO.	QUESTIONS AND FILTERS		PRES./ LISTEN.	PRES./ NOT LISTEN.	NOT PRES.
931	PRESENCE OF OTHERS AT THIS POINT (PRESENT AND LISTENING, PRESENT BUT NOT LISTENING, OR NOT PRESENT)	CHILDREN < 10	1	2	3
		HUSBAND	1	2	3
		OTHER MALES	1	2	3
		OTHER FEMALES	1	2	3

NO.	QUESTIONS AND FILTERS		YES	NO	DK
932	In your opinion, is a husband justified in hitting or beating his wife in the following situations:				
	a) If she goes out without telling him?	a) GOES OUT	1	2	8
	b) If she neglects the children?	b) NEGLECTS CHILDREN	1	2	8
	c) If she argues with him?	c) ARGUES	1	2	8
	d) If she refuses to have sex with him?	d) REFUSES SEX	1	2	8
	e) If she burns the food?	e) BURNS FOOD	1	2	8

SECTION 10. HIV/AIDS

NO.	QUESTIONS AND FILTERS	CODING CATEGORIES	SKIP
1001	Now I would like to talk about something else. Have you ever heard of HIV or AIDS?	YES . . . . . 1<br>NO 2	 → 1042
1002	HIV is the virus that can lead to AIDS. Can people reduce their chance of getting HIV by having just one uninfected sex partner who has no other sex partners?	YES . . . . . 1<br>NO . . . . . 2<br>DON'T KNOW 8	
1003	Can people get HIV from mosquito bites?	YES . . . . . 1<br>NO . . . . . 2<br>DON'T KNOW 8	
1004	Can people reduce their chance of getting HIV by using a condom every time they have sex?	YES . . . . . 1<br>NO . . . . . 2<br>DON'T KNOW 8	
1005	Can people get HIV by sharing food with a person who has HIV?	YES . . . . . 1<br>NO . . . . . 2<br>DON'T KNOW 8	
1006	Can people get HIV because of witchcraft or other supernatural means?	YES . . . . . 1<br>NO . . . . . 2<br>DON'T KNOW 8	
1007	Is it possible for a healthy-looking person to have HIV?	YES . . . . . 1<br>NO . . . . . 2<br>DON'T KNOW 8	
1008	Can HIV be transmitted from a mother to her baby:<br>a) During pregnancy?<br>b) During delivery?<br>c) By breastfeeding?	YES NO DK<br>a) DURING PREGNANCY . . 1 2 8<br>b) DURING DELIVERY . . . . . 1 2 8<br>c) BREASTFEEDING 1 2 8	
1009	CHECK 1008:<br>AT LEAST ONE 'YES' ☐ ↓	OTHER ☐	→ 1011
1010	Are there any special drugs that a doctor or a nurse can give to a woman infected with HIV to reduce the risk of transmission to the baby?	YES . . . . . 1<br>NO . . . . . 2<br>DON'T KNOW 8	
1011	CHECK 208 AND 215:<br>LAST BIRTH IN 2016-2018 ☐ ↓	NO BIRTHS ☐<br>LAST BIRTH IN 2015 OR EARLIER ☐	→ 1035<br>→ 1035
1012	CHECK 408 FOR LAST BIRTH:<br>HAD ANTENATAL CARE ☐ ↓	NO ANTENATAL CARE ☐	→ 1035
1014	During any of the antenatal visits for your last birth were you given any information about:<br>a) Babies getting HIV from their mother?<br>b) Things that you can do to prevent getting HIV?<br>c) Getting tested for HIV?	YES NO DK<br>a) HIV FROM MOTHER . . 1 2 8<br>b) THINGS TO DO . . . . 1 2 8<br>c) TESTED FOR HIV 1 2 8	
1035	Would you buy fresh vegetables from a shopkeeper or vendor if you knew that this person had HIV?	YES . . . . . 1<br>NO . . . . . 2<br>DON'T KNOW/NOT SURE/DEPENDS 8	

SECTION 10. HIV/AIDS

NO.	QUESTIONS AND FILTERS	CODING CATEGORIES	SKIP
1036	Do you think children living with HIV should be allowed to attend school with children who do not have HIV?	YES . . . . 1<br>NO . . . . 2<br>DON'T KNOW/NOT SURE/DEPENDS 8	
1037	Do you think people hesitate to take an HIV test because they are afraid of how other people will react if the test result is positive for HIV?	YES . . . . 1<br>NO . . . . 2<br>DON'T KNOW/NOT SURE/DEPENDS 8	
1038	Do people talk badly about people living with HIV, or who are thought to be living with HIV?	YES . . . . 1<br>NO . . . . 2<br>DON'T KNOW/NOT SURE/DEPENDS 8	
1039	Do people living with HIV, or thought to be living with HIV, lose the respect of other people?	YES . . . . 1<br>NO . . . . 2<br>DON'T KNOW/NOT SURE/DEPENDS 8	
1040	Do you agree or disagree with the following statement: I would be ashamed if someone in my family had HIV.	AGREE . . . . 1<br>DISAGREE . . . . 2<br>DON'T KNOW/NOT SURE/DEPENDS 8	
1041	Do you fear that you could get HIV if you come into contact with the saliva of a person living with HIV?	YES . . . . 1<br>NO . . . . 2<br>SAYS SHE HAS HIV . . . . 3<br>DON'T KNOW/NOT SURE/DEPENDS 8	
1042	CHECK 1001:<br>HEARD ABOUT HIV OR AIDS ☐<br>a) Apart from HIV, have you heard about other infections that can be transmitted through sexual contact?<br>NOT HEARD ABOUT HIV OR AIDS ☐<br>b) Have you heard about infections that can be transmitted through sexual contact?	YES . . . . 1<br>NO 2	
1043	CHECK 713:<br>HAS HAD SEXUAL INTERCOURSE ☐ NEVER HAD SEXUAL INTERCOURSE ☐ → 1051		1051
1044	CHECK 1042: HEARD ABOUT OTHER SEXUALLY TRANSMITTED INFECTIONS?<br>YES ☐ NO ☐ → 1046		1046
1045	Now I would like to ask you some questions about your health in the last 12 months. During the last 12 months, have you had a disease which you got through sexual contact?	YES . . . . 1<br>NO . . . . 2<br>DON'T KNOW 8	
1046	Sometimes women experience a bad-smelling abnormal genital discharge. During the last 12 months, have you had a bad-smelling abnormal genital discharge?	YES . . . . 1<br>NO . . . . 2<br>DON'T KNOW 8	
1047	Sometimes women have a genital sore or ulcer. During the last 12 months, have you had a genital sore or ulcer?	YES . . . . 1<br>NO . . . . 2<br>DON'T KNOW 8	
1048	CHECK 1045, 1046, AND 1047:<br>HAS HAD AN INFECTION (ANY 'YES') ☐ HAS NOT HAD AN INFECTION OR DOES NOT KNOW ☐ → 1051		1051
1049	The last time you had (PROBLEM FROM 1045/1046/1047), did you seek any kind of advice or treatment?	YES . . . . 1<br>NO 2	 → 1051

NO.	QUESTIONS AND FILTERS	CODING CATEGORIES	SKIP
1050	Where did you go?<br><br>Any other place?<br><br>PROBE TO IDENTIFY THE TYPE OF SOURCE.<br><br>IF UNABLE TO DETERMINE IF PUBLIC OR PRIVATE SECTOR, WRITE THE NAME OF THE PLACE.<br><br>________________<br>(NAME OF PLACE)	**PUBLIC SECTOR**<br>GOVERNMENT HOSPITAL . . . . A<br>GOVERNMENT HEALTH CENTER . . . . B<br>STAND-ALONE HTS CENTER . . . . C<br>FAMILY PLANNING CLINIC . . . . D<br>MOBILE HTS SERVICES . . . . E<br>OTHER PUBLIC SECTOR<br>________________ F<br>(SPECIFY)<br>**PRIVATE MEDICAL SECTOR**<br>PRIVATE HOSPITAL/CLINIC/<br>PRIVATE DOCTOR . . . . G<br>STAND-ALONE HTS CENTER . . . . H<br>PHARMACY . . . . I<br>CHEMIST/PMS STORE . . . . J<br>MOBILE HTS SERVICES . . . . K<br>OTHER PRIVATE MEDICAL SECTOR<br>________________ L<br>(SPECIFY)<br>**OTHER SOURCE**<br>SHOP . . . . M<br><br>OTHER ________________ X<br>(SPECIFY)	
1051	If a wife knows her husband has a disease that she can get during sexual intercourse, is she justified in asking that they use a condom when they have sex?	YES . . . . 1<br>NO . . . . 2<br>DON'T KNOW 8	
1052	Is a wife justified in refusing to have sex with her husband when she knows he has sex with other women?	YES . . . . 1<br>NO . . . . 2<br>DON'T KNOW 8	
1053	CHECK 701:<br>CURRENTLY MARRIED/ LIVING WITH A MAN ☐	NOT IN UNION ☐	→ 1101
1054	Can you say no to your (husband/partner) if you do not want to have sexual intercourse?	YES . . . . 1<br>NO . . . . 2<br>DEPENDS/NOT SURE 8	
1055	Could you ask your (husband/partner) to use a condom if you wanted him to?	YES . . . . 1<br>NO . . . . 2<br>DEPENDS/NOT SURE 8	

SECTION 11. OTHER HEALTH ISSUES

NO.	QUESTIONS AND FILTERS	CODING CATEGORIES	SKIP
1101	Now I would like to ask you some other questions relating to health matters. Have you had an injection for any reason in the last 12 months?<br><br>IF YES: How many injections have you had?<br><br>IF NUMBER OF INJECTIONS IS 90 OR MORE, OR DAILY FOR 3 MONTHS OR MORE, RECORD '90'. IF NON-NUMERIC ANSWER, PROBE TO GET AN ESTIMATE.	NUMBER OF INJECTIONS . . . . [ ][ ]<br><br>NONE 00	 <br>→1104
1102	Among these injections, how many were administered by a doctor, a nurse, a pharmacist, a dentist, or any other health worker?<br><br>IF NUMBER OF INJECTIONS IS 90 OR MORE, OR DAILY FOR 3 MONTHS OR MORE, RECORD '90'. IF NON-NUMERIC ANSWER, PROBE TO GET AN ESTIMATE.	NUMBER OF INJECTIONS . . . . [ ][ ]<br><br>NONE 00	 <br>→1104
1103	The last time you got an injection from a health worker, did he/she take the syringe and needle from a new, unopened package?	YES . . . . 1<br>NO . . . . 2<br>DON'T KNOW 8	
1104	Do you currently smoke cigarettes every day, some days, or not at all?	EVERY DAY . . . . 1<br>SOME DAYS . . . . 2<br>NOT AT ALL 3	2, 3 →1106
1105	On average, how many cigarettes do you currently smoke each day?	NUMBER OF CIGARETTES . . . . [ ][ ]	
1106	Do you currently smoke or use any other type of tobacco every day, some days, or not at all?	EVERY DAY . . . . 1<br>SOME DAYS . . . . 2<br>NOT AT ALL 3	 <br>→1108
1107	What other type of tobacco do you currently smoke or use?<br><br>RECORD ALL MENTIONED.	KRETEKS . . . . A<br>PIPES FULL OF TOBACCO . . . . B<br>CIGARS, CHEROOTS, OR CIGARILLOS . . . . C<br>WATER PIPE . . . . D<br>SNUFF BY MOUTH . . . . E<br>SNUFF BY NOSE . . . . F<br>CHEWING TOBACCO . . . . G<br>BETEL QUID WITH TOBACCO . . . . H<br><br>OTHER ______________________ X<br>(SPECIFY)	
1108	Many different factors can prevent women from getting medical advice or treatment for themselves. When you are sick and want to get medical advice or treatment, is each of the following a big problem or not a big problem:<br><br>a) Getting permission to go to the doctor?<br><br>b) Getting money needed for advice or treatment?<br><br>c) The distance to the health facility?<br><br>d) Not wanting to go alone?	BIG PROBLEM / NOT A BIG PROBLEM<br><br>a) PERMISSION TO GO . . . . . 1 2<br><br>b) GETTING MONEY . . . . 1 2<br><br>c) DISTANCE . . . . 1 2<br><br>d) GO ALONE 1 2	

SECTION 11. OTHER HEALTH ISSUES

NO.	QUESTIONS AND FILTERS	CODING CATEGORIES	SKIP
1108A	I am going to ask you about your opinion on behavior/practice on reducing the risk of malaria. Please tell me whether you agree or disagree with the following statements:	AGREE / DISAGREE / DON'T KNOW	
	b) The medicine given to pregnant women to prevent malaria works well to keep the mother healthy	b) 1 2 8	
	c) The medicine given to pregnant women to prevent malaria works well to keep the baby healthy when it is born	c) 1 2 8	
	d) The malaria tests are the only way to know if someone really has malaria or not	d) 1 2 8	
	f) Even if the malaria test shows that the fever is not caused by malaria, I will still seek out treatment for malaria because I don't trust the test result	f) 1 2 8	
	i) When the entire course of malaria medicine is taken, the disease will be fully cured	i) 1 2 8	
1108B	I am going to ask you about your opinion on consequences of malaria. Please tell me whether you agree or disagree with the following statements:	AGREE / DISAGREE / DON'T KNOW	
	a) Every case of malaria can potentially lead to death	a) 1 2 8	
	c) You don't worry about malaria because it can be easily treated	c) 1 2 8	
	d) You know people who have become dangerously sick with malaria.	d) 1 2 8	
	f) Only weak children can die from malaria	f) 1 2 8	
1109	Are you covered by any health insurance?	YES . . . . . 1<br>NO 2	 → 1200
1110	What type of health insurance are you covered by?<br><br>RECORD ALL MENTIONED.	MUTUAL HEALTH ORGANIZATION/ COMMUNITY-BASED HEALTH INSURANCE . . . . . A<br>HEALTH INSURANCE THROUGH EMPLOYER . . . . . B<br>SOCIAL SECURITY . . . . . C<br>OTHER PRIVATELY PURCHASED COMMERCIAL HEALTH INSURANCE . . . . . D<br>OTHER ______ X<br>(SPECIFY)	

SECTION 12. FEMALE GENITAL CUTTING/MUTILATION

NO.	QUESTIONS AND FILTERS	CODING CATEGORIES	SKIP
1200	CHECK COVER PAGE: HOUSEHOLD SELECTED FOR MAN'S SURVEY?<br>HOUSEHOLD NOT SELECTED FOR MAN'S SURVEY ☐ ↓	HOUSEHOLD SELECTED ☐	→ 1401
1201	Now I would like to ask some questions about a practice known as female circumcision, that is, a practice in which a girl may have part of her genitals cut, for example, excision of the clitoris and the labia minora, scraping of tissue surrounding the vaginal orifice (angurya cuts) or cutting of the vagina (gishiri cuts) and even use of corrosive sunstances or herbs into vagina to tighten or narrow it or to cause bleeding.<br><br>Have you ever heard about any of these practices?	YES . . . . . 1<br>NO 2	 → 1301
1202	Have you yourself ever had any of these procedures performed on you?	YES . . . . . 1<br>NO . . . . . 2<br>DON'T KNOW 8	 (NO, DON'T KNOW) → 1208
1203	Now I would like to ask you what was done to you at that time. Was any flesh removed from the genital area?	YES . . . . . 1<br>NO . . . . . 2<br>DON'T KNOW 8	→ 1205
1204	Was the genital area just nicked without removing any flesh?	YES . . . . . 1<br>NO . . . . . 2<br>DON'T KNOW 8	
1205	Was your genital area sewn closed?	YES . . . . . 1<br>NO . . . . . 2<br>DON'T KNOW 8	
1205A	Which type of procedure was performed on you?<br><br>a) Removal of clitoris along with partial or total excision of the labia minora?<br>b) Infibulation: removal of clitoris, labia minora and adjacent medial part of labia majora and stitching it?<br>c) Scraping of tissue surrounding the vaginal orifice (eg. Angurya cuts etc.)?<br>d) Cutting of the vagina (eg. Gishiri cuts etc)?	YES NO DK<br><br>a) REMOVAL OF CLITORIS . . 1 2 8<br>b) INFIBULATION . . . . . 1 2 8<br>c) ANGURYA . . . . . 1 2 8<br>d) GISHIRI 1 2 8	
1205B	Have you ever used corrosive substances or herbs into vagina with the aim of tightening or narrowing it or to cause bleeding?	YES . . . . . 1<br>NO . . . . . 2<br>DON'T KNOW 8	
1206	How old were you when this procedure (GC6A/GC6B) was performed for the first time?<br><br>IF THE RESPONDENT DOES NOT KNOW THE EXACT AGE, PROBE TO GET AN ESTIMATE.	AGE IN COMPLETED YEARS . . . . . [ ][ ]<br>AS A BABY/DURING INFANCY . . . . . 95<br>DON'T KNOW 98	
1207	Who performed this procedure?	TRADITIONAL<br>TRADITIONAL CURCUMCISER . . . . . 11<br>TRADITIONAL BIRTH ATTENDANT . . . . . 12<br>OTHER TRADITIONAL ______ 16<br>(SPECIFY)<br>HEALTH PROFESSIONAL<br>DOCTOR . . . . . 21<br>NURSE/MIDWIFE . . . . . 22<br>OTHER HEALTH PROFESSIONAL ______ 26<br>(SPECIFY)<br>DON'T KNOW 98	
1208	CHECK 213, 215 AND 216:<br>HAS ONE OR MORE LIVING DAUGHTERS BORN IN 2003 OR LATER ☐ ↓	HAS NO LIVING DAUGHTERS BORN IN 2003 OR LATER ☐	→ 1216

	CHECK 213, 215 AND 216: ENTER IN THE TABLE THE BIRTH HISTORY NUMBER AND NAME OF EACH LIVING DAUGHTER BORN IN 2003 OR LATER. ASK THE QUESTIONS ABOUT ALL OF THESE DAUGHTERS. BEGIN WITH THE YOUNGEST DAUGHTER. (IF THERE ARE MORE THAN 3 DAUGHTERS, USE ADDITIONAL QUESTIONNAIRES).			
1209	Now I would like to ask you some questions about your (daughter/daughters).			
		YOUNGEST LIVING DAUGHTER	NEXT-TO-YOUNGEST LIVING DAUGHTER	SECOND-TO-YOUNGEST LIVING DAUGHTER
1210	BIRTH HISTORY NUMBER AND NAME OF EACH LIVING DAUGHTER BORN IN 2003 OR LATER.	BIRTH HISTORY NUMBER [ ][ ]<br>NAME ______	BIRTH HISTORY NUMBER [ ][ ]<br>NAME ______	BIRTH HISTORY NUMBER [ ][ ]<br>NAME ______
1211	Is (NAME OF DAUGHTER) circumcised?	YES . . . . . 1<br>NO . . . . . 2<br>(GO TO 1211 IN NEXT COLUMN; OR IF NO MORE DAUGHTERS, GO TO 1216)	YES . . . . . 1<br>NO . . . . . 2<br>(GO TO 1211 IN NEXT COLUMN; OR IF NO MORE DAUGHTERS, GO TO 1216)	YES . . . . . 1<br>NO . . . . . 2<br>(GO TO 1211 IN NEXT COLUMN; OR IF NO MORE DAUGHTERS, GO TO 1216)
1212	How old was (NAME OF DAUGHTER) when she was circumcised?<br><br>IF THE RESPONDENT DOES NOT KNOW THE AGE, PROBE TO GET AN ESTIMATE.	AGE IN COMPLETED YRS . . [ ][ ]<br>DON'T KNOW 98	AGE IN COMPLETED YRS . . [ ][ ]<br>DON'T KNOW 98	AGE IN COMPLETED YRS . . [ ][ ]<br>DON'T KNOW 98
1213	Was her genital area sewn closed?	YES . . . . . 1<br>NO . . . . . 2<br>DON'T KNOW 8	YES . . . . . 1<br>NO . . . . . 2<br>DON'T KNOW 8	YES . . . . . 1<br>NO . . . . . 2<br>DON'T KNOW 8
1214	Who performed the circumcision?	**TRADITIONAL**<br>TRADITIONAL CIRCUMCISER . . 11<br>TRAD. BIRTH ATTENDANT . . 12<br>OTHER TRAD. ______ 16 (SPECIFY)<br><br>**HEALTH PROFESSIONAL**<br>DOCTOR . . . . . 21<br>NURSE/MIDWIFE . . 22<br>OTHER HEALTH PROFESSIONAL ______ 26 (SPECIFY)<br><br>DON'T KNOW 98	**TRADITIONAL**<br>TRADITIONAL CIRCUMCISER . . 11<br>TRAD. BIRTH ATTENDANT . . 12<br>OTHER TRAD. ______ 16 (SPECIFY)<br><br>**HEALTH PROFESSIONAL**<br>DOCTOR . . . . . 21<br>NURSE/MIDWIFE . . 22<br>OTHER HEALTH PROFESSIONAL ______ 26 (SPECIFY)<br><br>DON'T KNOW 98	**TRADITIONAL**<br>TRADITIONAL CIRCUMCISER . . 11<br>TRAD. BIRTH ATTENDANT . . 12<br>OTHER TRAD. ______ 16 (SPECIFY)<br><br>**HEALTH PROFESSIONAL**<br>DOCTOR . . . . . 21<br>NURSE/MIDWIFE . . 22<br>OTHER HEALTH PROFESSIONAL ______ 26 (SPECIFY)<br><br>DON'T KNOW 98
1215		GO BACK TO 1211 IN NEXT COLUMN; OR, IF NO MORE DAUGHTERS, GO TO 1216.	GO BACK TO 1211 IN NEXT COLUMN; OR, IF NO MORE DAUGHTERS, GO TO 1216.	GO BACK TO 1211 IN NEXT COLUMN; OR, IF NO MORE DAUGHTERS, GO TO 1216.
1216	Do you believe that female circumcision is required by your religion?		YES . . . . . 1<br>NO . . . . . 2<br>NO RELIGION . . . . . 3<br>DON'T KNOW 8	
1217	Do you think that female circumcision should be continued, or should it be stopped?		CONTINUED . . . . . 1<br>STOPPED . . . . . 2<br>DEPENDS . . . . . 3<br>DON'T KNOW 8	

SECTION 13. FISTULA

NO.	QUESTIONS AND FILTERS	CODING CATEGORIES	SKIP
1301	Sometimes a woman can have a problem of constant leakage of urine or stool from her vagina during the day and night. This problem usually occurs after a difficult childbirth, but may also occur after a sexual assault or after pelvic surgery.<br><br>Have you ever experienced a constant leakage of urine or stool from your vagina during the day and night?	YES . . . . 1<br>NO 2	1 → 1303
1302	Have you ever heard of this problem?	YES . . . . 1<br>NO 2	1, 2 → 1401
1303	Did this problem start after you delivered a baby or had a stillbirth?	AFTER DELIVERED BABY . . . . . . 1<br>AFTER HAD STILLBIRTH . . . . . . 2<br>NEITHER 3	3 → 1305
1304	Did this problem start after a normal labor and delivery, or after a very difficult labor and delivery?	NORMAL LABOR/DELIVERY . . . . . . 1<br>VERY DIFFICULT LABOR/DELIVERY 2	1, 2 → 1306
1305	What do you think caused this problem?	SEXUAL ASSAULT . . . . . . 1<br>PELVIC SURGERY . . . . . . 2<br>OTHER ____________ 6<br>(SPECIFY)<br>DON'T KNOW 8	8 → 1307
1306	How many days after (CAUSE OF PROBLEM FROM 1303 OR 1305) did the leakage start?<br><br>ENTER '90' IF 90 DAYS OR MORE.	NUMBER OF DAYS AFTER DELIVERY/OTHER EVENT . . . . . [ ][ ]	
1306A	How old were you when you experienced this problem?	AGE IN YEARS . . . . [ ][ ]	
1307	Have you sought treatment for this condition?	YES . . . . 1<br>NO 2	1 → 1309
1308	Why have you not sought treatment?	DO NOT KNOW CAN BE FIXED . . . . A<br>DO NOT KNOW WHERE TO GO . . . . B<br>TOO EXPENSIVE . . . . C<br>TOO FAR . . . . D<br>POOR QUALITY OF CARE . . . . . . E<br>COULD NOT GET PERMISSION . . . . F<br>EMBARRASSMENT . . . . G<br>PROBLEM DISAPPEARED . . . . . . H<br>OTHER ____________ X<br>(SPECIFY)	→ 1401
1309	From whom did you last seek treatment?	**HEALTH PROFESSIONAL**<br>DOCTOR . . . . 1<br>NURSE/MIDWIFE . . . . 2<br>**OTHER PERSON**<br>COMMUNITY/VILLAGE HEALTH WORKER . . . . . . 3<br>OTHER ____________ 6<br>(SPECIFY)	
1310	Did you have an operation to fix the problem?	YES . . . . 1<br>NO 2	
1311	Did the treatment stop the leakage completely?<br><br>IF NO: Did the treatment reduce the leakage?	YES, STOPPED COMPLETELY . . . . 1<br>NOT STOPPED BUT REDUCED . . . . 2<br>NOT STOPPED AT ALL . . . . . . 3<br>DID NOT RECEIVE TREATMENT 4	
1312	Are there any (other) women in your household who suffer from obstetric fistula?	YES . . . . 1<br>NO 2	2 → 1401
1313	How many (other) women in your household suffer from obstetric fistula?	NUMBER . . . . [ ][ ]<br>DON'T KNOW 98	

1401	Now I would like to ask you some questions about your brothers and sisters born to your natural mother, including those who are living with you, those living elsewhere and those who have died. From our experience in prior surveys, we know it may sometimes be difficult to establish a complete list of all the children born to your natural mother. We will work together to draw the most complete list and work to recall all your siblings. Could you please now give me the names of all of your brothers and sisters born to your natural mother.<br>DO NOT FILL IN THE ORDER NUMBER YET.<br><br>NAME / ORDER NUMBER / NAME / ORDER NUMBER<br>a ____ [ ][ ] k ____ [ ][ ]<br>b ____ [ ][ ] l ____ [ ][ ]<br>c ____ [ ][ ] m ____ [ ][ ]<br>d ____ [ ][ ] n ____ [ ][ ]<br>e ____ [ ][ ] o ____ [ ][ ]<br>f ____ [ ][ ] p ____ [ ][ ]<br>g ____ [ ][ ] q ____ [ ][ ]<br>h ____ [ ][ ] r ____ [ ][ ]<br>i ____ [ ][ ] s ____ [ ][ ]<br>j ____ [ ][ ] t ____ [ ][ ]	
1402	CHECK 1401:<br>ONE OR MORE BROTHERS OR SISTERS LISTED [ ]<br>NO BROTHERS OR SISTERS LISTED [ ]	1404
1403	READ THE NAMES OF THE BROTHERS AND SISTERS TO THE RESPONDENT AND AFTER THE LAST ONE ASK: Are there any other brothers and sisters from the same mother that you have not mentioned?<br>NO [ ] YES [ ] → LIST ADDITIONAL BROTHERS AND SISTERS IN 1401.	
1404	Sometimes people forget to mention children born to their natural mother because they do not live with them or they do not see them very often. Are there any brothers or sisters who do not live with you that you have not mentioned?<br>NO [ ] YES [ ] → LIST ADDITIONAL BROTHERS AND SISTERS IN 1401.	
1405	Sometimes people forget to mention children born to their natural mother because they have died. Are there any brothers or sisters who died that you have not mentioned?<br>NO [ ] YES [ ] → LIST ADDITIONAL BROTHERS AND SISTERS IN 1401.	
1406	Some people have brothers or sisters from the same mother but a different father. Are there any brothers or sisters born to your natural mother, but who have a different natural father, that you have not mentioned?<br>NO [ ] YES [ ] → LIST ADDITIONAL BROTHERS AND SISTERS IN 1401.	
1407	COUNT THE NUMBER OF BROTHERS AND SISTERS RECORDED IN 1401. — TOTAL BROTHERS AND SISTERS . . [ ][ ]	

1408	CHECK 1407:<br>Just to make make sure that I have this right: Your mother had in TOTAL ________ births, excluding you, during her lifetime. Is that correct?<br>YES ☐ NO ☐ → PROBE AND CORRECT 1401 AND/OR 1407	
1409	CHECK 1407:<br>ONE OR MORE BROTHERS/SISTERS ☐ NO BROTHER OR SISTER ☐ →	1501
1410	Please tell me, which brother or sister was born first? And which was born next?<br>RECORD '01' FOR THE ORDER NUMBER IN 1401 FOR THE FIRST BROTHER OR SISTER, '02' FOR THE SECOND, AND SO ON UNTIL YOU HAVE RECORDED THE ORDER NUMBER FOR ALL BROTHERS AND SISTERS.	
1411	How many births did your mother have before you were born?<br>NUMBER OF PRECEDING BIRTHS ☐☐	

1412	LIST THE BROTHERS AND SISTERS ACCORDING TO THE ORDER NUMBER IN 1401. ASK 1413 TO 1424 FOR ONE BROTHER OR SISTER BEFORE ASKING ABOUT THE NEXT BROTHER OR SISTER. IF THERE ARE MORE THAN 12 BROTHERS AND SISTERS, USE AN ADDITIONAL QUESTIONNAIRE.						
1413	NAME OF BROTHER OR SISTER	(01)<br>________	(02)<br>________	(03)<br>________	(04)<br>________	(05)<br>________	(06)<br>________
1414	Is (NAME) male or female?	MALE . . . 1<br>FEMALE . 2	MALE . . . 1<br>FEMALE . 2	MALE . . . 1<br>FEMALE . 2	MALE . . . 1<br>FEMALE . 2	MALE . . . 1<br>FEMALE . 2	MALE . . . 1<br>FEMALE . 2
1415	Is (NAME) still alive?	YES . . . . . 1<br>NO . . . . . 2<br>GO TO 1417<br>DK . . . . . 8<br>GO TO (02)	YES . . . . . 1<br>NO . . . . . 2<br>GO TO 1417<br>DK . . . . . 8<br>GO TO (03)	YES . . . . . 1<br>NO . . . . . 2<br>GO TO 1417<br>DK . . . . . 8<br>GO TO (04)	YES . . . . . 1<br>NO . . . . . 2<br>GO TO 1417<br>DK . . . . . 8<br>GO TO (05)	YES . . . . . 1<br>NO . . . . . 2<br>GO TO 1417<br>DK . . . . . 8<br>GO TO (06)	YES . . . . . 1<br>NO . . . . . 2<br>GO TO 1417<br>DK . . . . . 8<br>GO TO (07)
1416	How old is (NAME)?	☐☐<br>GO TO (02)	☐☐<br>GO TO (03)	☐☐<br>GO TO (04)	☐☐<br>GO TO (05)	☐☐<br>GO TO (06)	☐☐<br>GO TO (07)
1417	How many years ago did (NAME) die?	☐☐	☐☐	☐☐	☐☐	☐☐	☐☐
1418	How old was (NAME) when (he/she) died?<br>IF DON'T KNOW, PROBE AND ASK ADDITIONAL QUESTIONS TO GET AN ESTIMATE	☐☐<br>IF MALE OR DIED BEFORE 12 YEARS OF AGE, GO TO 1423	☐☐<br>IF MALE OR DIED BEFORE 12 YEARS OF AGE, GO TO 1423	☐☐<br>IF MALE OR DIED BEFORE 12 YEARS OF AGE, GO TO 1423	☐☐<br>IF MALE OR DIED BEFORE 12 YEARS OF AGE, GO TO 1423	☐☐<br>IF MALE OR DIED BEFORE 12 YEARS OF AGE, GO TO 1423	☐☐<br>IF MALE OR DIED BEFORE 12 YEARS OF AGE, GO TO 1423
1419	Was (NAME) pregnant when she died?	YES . . . . . 1<br>GO TO 1422A<br>NO 2	YES . . . . . 1<br>GO TO 1422A<br>NO 2	YES . . . . . 1<br>GO TO 1422A<br>NO 2	YES . . . . . 1<br>GO TO 1422A<br>NO 2	YES . . . . . 1<br>GO TO 1422A<br>NO 2	YES . . . . . 1<br>GO TO 1422A<br>NO 2
1420	Did (NAME) die during childbirth?	YES . . . . . 1<br>GO TO 1422A<br>NO 2	YES . . . . . 1<br>GO TO 1422A<br>NO 2	YES . . . . . 1<br>GO TO 1422A<br>NO 2	YES . . . . . 1<br>GO TO 1422A<br>NO 2	YES . . . . . 1<br>GO TO 1422A<br>NO 2	YES . . . . . 1<br>GO TO 1422A<br>NO 2
1421	Did (NAME) die within two months after the end of a pregnancy or childbirth?	YES . . . . . 1<br>NO . . . . . 2<br>GO TO 1423	YES . . . . . 1<br>NO . . . . . 2<br>GO TO 1423	YES . . . . . 1<br>NO . . . . . 2<br>GO TO 1423	YES . . . . . 1<br>NO . . . . . 2<br>GO TO 1423	YES . . . . . 1<br>NO . . . . . 2<br>GO TO 1423	YES . . . . . 1<br>NO . . . . . 2<br>GO TO 1423
1422	How many days after the end of the pregnancy did (NAME)	☐☐	☐☐	☐☐	☐☐	☐☐	☐☐
1422A	In which State did (NAME) WRITE THE STATE CODE.	☐☐	☐☐	☐☐	☐☐	☐☐	☐☐
1422B	CHECK 1420:	YES ☐ GO TO (02)<br>NO/ NOT ASKED ☐	YES ☐ GO TO (03)<br>NO/ NOT ASKED ☐	YES ☐ GO TO (04)<br>NO/ NOT ASKED ☐	YES ☐ GO TO (05)<br>NO/ NOT ASKED ☐	YES ☐ GO TO (06)<br>NO/ NOT ASKED ☐	YES ☐ GO TO (07)<br>NO/ NOT ASKED ☐
1423	Was (NAME)'s death due to an act of violence?	YES . . . . . 1<br>GO TO (02)<br>NO 2	YES . . . . . 1<br>GO TO (03)<br>NO 2	YES . . . . . 1<br>GO TO (04)<br>NO 2	YES . . . . . 1<br>GO TO (05)<br>NO 2	YES . . . . . 1<br>GO TO (06)<br>NO 2	YES . . . . . 1<br>GO TO (07)<br>NO 2
1424	Was (NAME)'s death due to an accident?	YES . . . . . 1<br>NO . . . . . 2<br>GO TO (02)	YES . . . . . 1<br>NO . . . . . 2<br>GO TO (03)	YES . . . . . 1<br>NO . . . . . 2<br>GO TO (04)	YES . . . . . 1<br>NO . . . . . 2<br>GO TO (05)	YES . . . . . 1<br>NO . . . . . 2<br>GO TO (06)	YES . . . . . 1<br>NO . . . . . 2<br>GO TO (07)

IF NO MORE BROTHERS OR SISTERS, GO TO NEXT SECTION.

1412	LIST THE BROTHERS AND SISTERS ACCORDING TO THE ORDER NUMBER IN 1401. ASK 1413 TO 1424 FOR ONE BROTHER OR SISTER BEFORE ASKING ABOUT THE NEXT BROTHER OR SISTER. IF THERE ARE MORE THAN 12 BROTHERS AND SISTERS, USE AN ADDITIONAL QUESTIONNAIRE.

1413	NAME OF BROTHER OR SISTER	(07) ________	(08) ________	(09) ________	(10) ________	(11) ________	(12) ________
1414	Is (NAME) male or female?	MALE . . . 1<br>FEMALE . 2	MALE . . . 1<br>FEMALE . 2	MALE . . . 1<br>FEMALE . 2	MALE . . . 1<br>FEMALE . 2	MALE . . . 1<br>FEMALE . 2	MALE . . . 1<br>FEMALE . 2
1415	Is (NAME) still alive?	YES . . . . . 1<br>NO . . . . . 2<br>GO TO 1417<br>DK . . . . . 8<br>GO TO (08)	YES . . . . . 1<br>NO . . . . . 2<br>GO TO 1417<br>DK . . . . . 8<br>GO TO (09)	YES . . . . . 1<br>NO . . . . . 2<br>GO TO 1417<br>DK . . . . . 8<br>GO TO (10)	YES . . . . . 1<br>NO . . . . . 2<br>GO TO 1417<br>DK . . . . . 8<br>GO TO (11)	YES . . . . . 1<br>NO . . . . . 2<br>GO TO 1417<br>DK . . . . . 8<br>GO TO (12)	YES . . . . . 1<br>NO . . . . . 2<br>GO TO 1417<br>DK . . . . . 8<br>GO TO (13)
1416	How old is (NAME)?	[ ][ ]<br>GO TO (08)	[ ][ ]<br>GO TO (09)	[ ][ ]<br>GO TO (10)	[ ][ ]<br>GO TO (11)	[ ][ ]<br>GO TO (12)	[ ][ ]<br>GO TO (13)
1417	How many years ago did (NAME) die?	[ ][ ]	[ ][ ]	[ ][ ]	[ ][ ]	[ ][ ]	[ ][ ]
1418	How old was (NAME) when (he/she) died?<br>IF DON'T KNOW, PROBE AND ASK ADDITIONAL QUESTIONS TO GET AN ESTIMATE	[ ][ ]<br>IF MALE OR DIED BEFORE 12 YEARS OF AGE, GO TO 1423	[ ][ ]<br>IF MALE OR DIED BEFORE 12 YEARS OF AGE, GO TO 1423	[ ][ ]<br>IF MALE OR DIED BEFORE 12 YEARS OF AGE, GO TO 1423	[ ][ ]<br>IF MALE OR DIED BEFORE 12 YEARS OF AGE, GO TO 1423	[ ][ ]<br>IF MALE OR DIED BEFORE 12 YEARS OF AGE, GO TO 1423	[ ][ ]<br>IF MALE OR DIED BEFORE 12 YEARS OF AGE, GO TO 1423
1419	Was (NAME) pregnant when she died?	YES . . . . . 1<br>GO TO 1422A<br>NO 2	YES . . . . . 1<br>GO TO 1422A<br>NO 2	YES . . . . . 1<br>GO TO 1422A<br>NO 2	YES . . . . . 1<br>GO TO 1422A<br>NO 2	YES . . . . . 1<br>GO TO 1422A<br>NO 2	YES . . . . . 1<br>GO TO 1422A<br>NO 2
1420	Did (NAME) die during childbirth?	YES . . . . . 1<br>GO TO 1422A<br>NO 2	YES . . . . . 1<br>GO TO 1422A<br>NO 2	YES . . . . . 1<br>GO TO 1422A<br>NO 2	YES . . . . . 1<br>GO TO 1422A<br>NO 2	YES . . . . . 1<br>GO TO 1422A<br>NO 2	YES . . . . . 1<br>GO TO 1422A<br>NO 2
1421	Did (NAME) die within two months after the end of a pregnancy or childbirth?	YES . . . . . 1<br>NO . . . . . 2<br>GO TO 1423	YES . . . . . 1<br>NO . . . . . 2<br>GO TO 1423	YES . . . . . 1<br>NO . . . . . 2<br>GO TO 1423	YES . . . . . 1<br>NO . . . . . 2<br>GO TO 1423	YES . . . . . 1<br>NO . . . . . 2<br>GO TO 1423	YES . . . . . 1<br>NO . . . . . 2<br>GO TO 1423
1422	How many days after the end of the pregnancy did (NAME)	[ ][ ]	[ ][ ]	[ ][ ]	[ ][ ]	[ ][ ]	[ ][ ]
1422A	In which State did (NAME)<br>WRITE THE STATE CODE.	[ ][ ]	[ ][ ]	[ ][ ]	[ ][ ]	[ ][ ]	[ ][ ]
1422B	CHECK 1420:	YES [ ] GO TO (08)<br>NO/NOT ASKED [ ]	YES [ ] GO TO (09)<br>NO/NOT ASKED [ ]	YES [ ] GO TO (10)<br>NO/NOT ASKED [ ]	YES [ ] GO TO (11)<br>NO/NOT ASKED [ ]	YES [ ] GO TO (12)<br>NO/NOT ASKED [ ]	YES [ ] GO TO (13)<br>NO/NOT ASKED [ ]
1423	Was (NAME)'s death due to an act of violence?	YES . . . . . 1<br>GO TO (08)<br>NO 2	YES . . . . . 1<br>GO TO (09)<br>NO 2	YES . . . . . 1<br>GO TO (10)<br>NO 2	YES . . . . . 1<br>GO TO (11)<br>NO 2	YES . . . . . 1<br>GO TO (12)<br>NO 2	YES . . . . . 1<br>GO TO (13)<br>NO 2
1424	Was (NAME)'s death due to an accident?	YES . . . . . 1<br>NO . . . . . 2<br>GO TO (08)	YES . . . . . 1<br>NO . . . . . 2<br>GO TO (09)	YES . . . . . 1<br>NO . . . . . 2<br>GO TO (10)	YES . . . . . 1<br>NO . . . . . 2<br>GO TO (11)	YES . . . . . 1<br>NO . . . . . 2<br>GO TO (12)	YES . . . . . 1<br>NO . . . . . 2<br>GO TO (13)

IF NO MORE BROTHERS OR SISTERS, GO TO NEXT SECTION.

SECTION 15: DOMESTIC VIOLENCE MODULE

NO.	QUESTIONS AND FILTERS	CODING CATEGORIES	SKIP
1500	CHECK COVER PAGE: WOMAN SELECTED FOR DV MODULE?<br>WOMAN SELECTED FOR THIS SECTION ☐ ↓	WOMAN NOT SELECTED ☐	→ 1533
1501	CHECK FOR PRESENCE OF OTHERS:<br>DO NOT CONTINUE UNTIL PRIVACY IS ENSURED.<br>PRIVACY OBTAINED .... 1 ↓	PRIVACY NOT POSSIBLE 2	→ 1532
1501A	READ TO THE RESPONDENT:<br>Now I would like to ask you questions about some other important aspects of a woman's life. You may find some of these questions very personal. However, your answers are crucial for helping to understand the condition of women in Nigeria. Let me assure you that your answers are completely confidential and will not be told to anyone and no one else in your household will know that you were asked these questions. If I ask you any question you don't want to answer, just let me know and I will go on to the next question.		
1502	CHECK 701 AND 702:<br>CURRENTLY MARRIED/ LIVING WITH A MAN ☐ ↓<br>FORMERLY MARRIED/ LIVED WITH A MAN (READ IN PAST TENSE AND USE 'LAST' WITH 'HUSBAND/PARTNER') ☐ ↓	NEVER MARRIED/ NEVER LIVED WITH A MAN ☐	→ 1516

NO.	QUESTIONS AND FILTERS		YES	NO	DK	SKIP
1503	First, I am going to ask you about some situations which happen to some women. Please tell me if these apply to your relationship with your (last) (husband/partner)?					
	a) He (is/was) jealous or angry if you (talk/talked) to other men?	JEALOUS	1	2	8	
	b) He frequently (accuses/accused) you of being unfaithful?	ACCUSES	1	2	8	
	c) He (does/did) not permit you to meet your female friends?	NOT MEET FRIENDS ..	1	2	8	
	d) He (tries/tried) to limit your contact with your family?	NO FAMILY	1	2	8	
	e) He (insists/insisted) on knowing where you (are/were) at all times?	WHERE YOU ARE	1	2	8	

NO.	QUESTIONS AND FILTERS	EVER	OFTEN	SOME-TIMES	NOT IN LAST 12 MONTHS	SKIP
1504	Now I need to ask some more questions about your relationship with your (last) (husband/partner).<br>A. Did your (last) (husband/partner) ever:		B. How often did this happen during the last 12 months: often, only sometimes, or not at all?			
	a) say or do something to humiliate you in front of others?	YES 1 →<br>NO 2 ↓	1	2	3	
	b) threaten to hurt or harm you or someone you care about?	YES 1 →<br>NO 2 ↓	1	2	3	
	c) insult you or make you feel bad about yourself?	YES 1 →<br>NO 2 ↓	1	2	3	

SECTION 15: DOMESTIC VIOLENCE MODULE

NO.	QUESTIONS AND FILTERS		CODING CATEGORIES			SKIP
1505	A. Did your (last) (husband/partner) ever do any of the following things to you:		B. How often did this happen during the last 12 months: often, only sometimes, or not at all?			
		EVER	OFTEN	SOME-TIMES	NOT IN LAST 12 MONTHS	
	a) push you, shake you, or throw something at you?	YES 1 → NO 2 ↓	1	2	3	
	b) slap you?	YES 1 → NO 2 ↓	1	2	3	
	c) twist your arm or pull your hair?	YES 1 → NO 2 ↓	1	2	3	
	d) punch you with his fist or with something that could hurt you?	YES 1 → NO 2 ↓	1	2	3	
	e) kick you, drag you, or beat you up?	YES 1 → NO 2 ↓	1	2	3	
	f) try to choke you or burn you on purpose?	YES 1 → NO 2 ↓	1	2	3	
	g) threaten or attack you with a knife, gun, or other weapon?	YES 1 → NO 2 ↓	1	2	3	
	h) physically force you to have sexual intercourse with him when you did not want to?	YES 1 → NO 2 ↓	1	2	3	
	i) physically force you to perform any other sexual acts you did not want to?	YES 1 → NO 2 ↓	1	2	3	
	j) force you with threats or in any other way to perform sexual acts you did not want to?	YES 1 → NO 2 ↓	1	2	3	
1506	CHECK 1505A (a-j): AT LEAST ONE 'YES' ☐ ↓		NOT A SINGLE 'YES' ☐ →			1509
1507	How long after you first (got married/started living together) with your (last) (husband/partner) did (this/any of these things) first happen? IF LESS THAN ONE YEAR, RECORD '00'.		NUMBER OF YEARS . . . . . . [ ][ ] BEFORE MARRIAGE/BEFORE LIVING TOGETHER 95			
1508	Did the following ever happen as a result of what your (last) (husband/partner) did to you:					
	a) You had cuts, bruises, or aches?		YES . . . . 1 NO 2			
	b) You had eye injuries, sprains, dislocations, or burns?		YES . . . . 1 NO 2			
	c) You had deep wounds, broken bones, broken teeth, or any other serious injury?		YES . . . . 1 NO 2			
1509	Have you ever hit, slapped, kicked, or done anything else to physically hurt your (last) (husband/partner) at times when he was not already beating or physically hurting you?		YES . . . . 1 NO 2			 → 1511

NO.	QUESTIONS AND FILTERS	CODING CATEGORIES	SKIP
1510	In the last 12 months, how often have you done this to your (last) (husband/partner): often, only sometimes, or not at all?	OFTEN . . . 1<br>SOMETIMES . . . 2<br>NOT AT ALL . . . 3	
1511	Does (did) your (last) (husband/partner) drink alcohol?	YES . . . 1<br>NO . . . 2	 2 → 1513
1512	How often does (did) he get drunk: often, only sometimes, or never?	OFTEN . . . 1<br>SOMETIMES . . . 2<br>NEVER . . . 3	
1513	Are (Were) you afraid of your (last) (husband/partner): most of the time, sometimes, or never?	MOST OF THE TIME AFRAID . . . 1<br>SOMETIMES AFRAID . . . 2<br>NEVER AFRAID . . . 3	
1514	CHECK 709:<br>MARRIED MORE THAN ONCE ☐ ↓	MARRIED ONLY ONCE ☐	→ 1516

1515

A. So far we have been talking about the behavior of your (current/last) (husband/partner). Now I want to ask you about the behavior of any previous (husband/partner).

B. How long ago did this last happen?

	EVER	0 - 11 MONTHS AGO	12+ MONTHS AGO	DON'T REMEMBER
a) Did any previous (husband/partner) ever hit, slap, kick, or do anything else to hurt you physically?	YES 1 →<br>NO 2 ↓	1	2	3
b) Did any previous (husband/partner) physically force you to have intercourse or perform any other sexual acts against your will?	YES 1 →<br>NO 2 ↓	1	2	3
c) Did any previous (husband/partner) humiliate you in front of others, threaten to hurt you or someone you care about, or insult you or make you feel bad about yourself?	YES 1 →<br>NO 2 ↓	1	2	3

NO.	QUESTIONS AND FILTERS	CODING CATEGORIES	SKIP
1516	CHECK 701 AND 702:<br>EVER MARRIED/EVER LIVED WITH A MAN ☐ ↓ / NEVER MARRIED/NEVER LIVED WITH A MAN ☐ ↓<br>a) From the time you were 15 years old has anyone other than (your/any) (husband/partner) hit you, slapped you, kicked you, or done anything else to hurt you physically?<br>b) From the time you were 15 years old has anyone hit you, slapped you, kicked you, or done anything else to hurt you physically?	YES . . . 1<br>NO . . . 2<br>REFUSED TO ANSWER/ NO ANSWER . . . 3	 2, 3 → 1519

NO.	QUESTIONS AND FILTERS	CODING CATEGORIES	SKIP
1517	Who has hurt you in this way?<br><br>Anyone else?<br><br>RECORD ALL MENTIONED.	MOTHER/STEP-MOTHER . . . . A<br>FATHER/STEP-FATHER . . . . B<br>SISTER/BROTHER . . . . C<br>DAUGHTER/SON . . . . D<br>OTHER RELATIVE . . . . E<br>CURRENT BOYFRIEND . . . . F<br>FORMER BOYFRIEND . . . . G<br>MOTHER-IN-LAW . . . . H<br>FATHER-IN-LAW . . . . I<br>OTHER IN-LAW . . . . J<br>TEACHER . . . . K<br>EMPLOYER/SOMEONE AT WORK . . . . . L<br>POLICE/SOLDIER . . . . M<br><br>OTHER ______________ X<br>(SPECIFY)	
1518	In the last 12 months, how often has (this person/have these persons) physically hurt you: often, only sometimes, or not at all?	OFTEN . . . . 1<br>SOMETIMES . . . . 2<br>NOT AT ALL 3	
1519	CHECK 201, 226, AND 230:<br><br>EVER BEEN PREGNANT ☐ ('YES' ON 201 OR 226 OR 230) ↓<br>NEVER BEEN PREGNANT ☐ → 1522		1522
1520	Has any one ever hit, slapped, kicked, or done anything else to hurt you physically while you were pregnant?	YES . . . . 1<br>NO 2	 → 1522
1521	Who has done any of these things to physically hurt you while you were pregnant?<br><br>Anyone else?<br><br>RECORD ALL MENTIONED.	CURRENT HUSBAND/PARTNER . . . . . A<br>MOTHER/STEP-MOTHER . . . . B<br>FATHER/STEP-FATHER . . . . C<br>SISTER/BROTHER . . . . D<br>DAUGHTER/SON . . . . E<br>OTHER RELATIVE . . . . F<br>FORMER HUSBAND/PARTNER . . . . . G<br>CURRENT BOYFRIEND . . . . H<br>FORMER BOYFRIEND . . . . I<br>MOTHER-IN-LAW . . . . J<br>FATHER-IN-LAW . . . . K<br>OTHER IN-LAW . . . . L<br>TEACHER . . . . M<br>EMPLOYER/SOMEONE AT WORK . . . . . N<br>POLICE/SOLDIER . . . . O<br><br>OTHER ______________ X<br>(SPECIFY)	
1522	CHECK 701 AND 702:<br><br>EVER MARRIED/EVER LIVED WITH A MAN ☐ ↓<br>NEVER MARRIED/NEVER LIVED WITH A MAN ☐ → 1522B		1522B

NO.	QUESTIONS AND FILTERS	CODING CATEGORIES	SKIP
1522A	Now I want to ask you about things that may have been done to you by someone other than (your/any) (husband/partner). At any time in your life, as a child or as an adult, has anyone ever forced you in any way to have sexual intercourse or perform any other sexual acts when you did not want to?	YES . . . . 1<br>NO . . . . 2<br>REFUSED TO ANSWER/ NO ANSWER 3	1 → 1523<br>2, 3 → 1524A
1522B	At any time in your life, as a child or as an adult, has anyone ever forced you in any way to have sexual intercourse or perform any other sexual acts when you did not want to?	YES . . . . 1<br>NO . . . . 2<br>REFUSED TO ANSWER/ NO ANSWER 3	2, 3 → 1526
1523	Who was the person who was forcing you the very first time this happened?	CURRENT HUSBAND/PARTNER . . . . 01<br>FORMER HUSBAND/PARTNER . . . . 02<br>CURRENT/FORMER BOYFRIEND . . . . 03<br>FATHER/STEP-FATHER . . . . 04<br>BROTHER/STEP-BROTHER . . . . 05<br>OTHER RELATIVE . . . . 06<br>IN-LAW . . . . 07<br>OWN FRIEND/ACQUAINTANCE . . . . 08<br>FAMILY FRIEND . . . . 09<br>TEACHER . . . . 10<br>EMPLOYER/SOMEONE AT WORK . . . . 11<br>POLICE/SOLDIER . . . . 12<br>PRIEST/RELIGIOUS LEADER . . . . 13<br>STRANGER . . . . 14<br><br>OTHER ______ 96<br>(SPECIFY)	
1524	CHECK 701 AND 702:<br>EVER MARRIED/EVER LIVED WITH A MAN ☐ / NEVER MARRIED/NEVER LIVED WITH A MAN ☐<br>a) In the last 12 months, has anyone other than (your/any) (husband/partner) physically forced you to have sexual intercourse when you did not want to?<br>b) In the last 12 months has anyone physically forced you to have sexual intercourse when you did not want to?	YES . . . . 1<br>NO 2	1, 2 → 1525
1524A	CHECK 1505A (h-j) and 1515A(b)<br>AT LEAST ONE 'YES' ☐ ↓ / NOT A SINGLE 'YES' ☐ → 1526		1526
1525	CHECK 701 AND 702:<br>EVER MARRIED/EVER LIVED WITH A MAN ☐ / NEVER MARRIED/NEVER LIVED WITH A MAN ☐<br>a) How old were you the first time you were forced to have sexual intercourse or perform any other sexual acts by anyone, including (your/any) husband/partner?<br>b) How old were you the first first time you were forced to have sexual intercourse or perform any other sexual acts?	AGE IN COMPLETED YEARS . . . . [ ][ ]<br><br>DON'T KNOW 98	
1526	CHECK 1505A (a-j), 1515A (a,b), 1516, 1520, 1522A, AND 1522B:<br>AT LEAST ONE 'YES' ☐ ↓ / NOT A SINGLE 'YES' ☐ → 1530		1530
1527	Thinking about what you yourself have experienced among the different things we have been talking about, have you ever tried to seek help?	YES . . . . 1<br>NO 2	2 → 1529

NO.	QUESTIONS AND FILTERS	CODING CATEGORIES	SKIP
1528	From whom have you sought help?<br><br>Anyone else?<br><br>RECORD ALL MENTIONED.	OWN FAMILY . . . . . A<br>HUSBAND'S/PARTNER'S FAMILY . . . . . B<br>CURRENT/FORMER HUSBAND/PARTNER . . . . . C<br>CURRENT/FORMER BOYFRIEND . . . . . D<br>FRIEND . . . . . E<br>NEIGHBOR . . . . . F<br>RELIGIOUS LEADER . . . . . G<br>DOCTOR/MEDICAL PERSONNEL . . . . . H<br>POLICE . . . . . I<br>LAWYER . . . . . J<br>SOCIAL SERVICE ORGANIZATION . . . . . K<br><br>OTHER ______ X<br>(SPECIFY)	→ 1530
1529	Have you ever told any one about this?	YES . . . . . 1<br>NO 2	
1530	As far as you know, did your father ever beat your mother?	YES . . . . . 1<br>NO . . . . . 2<br>DON'T KNOW 8	
	THANK THE RESPONDENT FOR HER COOPERATION AND REASSURE HER ABOUT THE CONFIDENTIALITY OF HER ANSWERS. FILL OUT THE QUESTIONS BELOW WITH REFERENCE TO THE DOMESTIC VIOLENCE MODULE ONLY.		
1531	DID YOU HAVE TO INTERRUPT THE INTERVIEW BECAUSE SOME ADULT WAS TRYING TO LISTEN, OR CAME INTO THE ROOM, OR INTERFERED IN ANY OTHER WAY?	(see below)	
1532	INTERVIEWER'S COMMENTS/EXPLANATION FOR NOT COMPLETING THE DOMESTIC VIOLENCE MODULE.<br>______<br>______<br>______		
1533	CHECK 223A:<br>ONE OR MORE DEATHS ☐ ↓	NO DEATHS ☐	→ 1535
1534	READ TO THE RESPONDENT:<br>I would like to inform you that detailed information on the circumstances surrounding the deaths of children under the age of 5 years will be collected in the near future so that the federal government of Nigeria can provide health services to help reduce these deaths. If you do not mind, another team will be coming at a leter date to interview members of the household about the death (s) you have told me about. Is this okay?	YES . . . . . 1<br><br>NO 2	
1535	RECORD THE TIME.	HOURS ☐☐<br>MINUTES ☐☐	

Question 1531 coding categories:

	YES, ONCE	YES, MORE THAN ONCE	NO
HUSBAND	1	2	3
OTHER MALE ADULT	1	2	3
FEMALE ADULT	1	2	3

INTERVIEWER'S OBSERVATIONS

TO BE FILLED IN AFTER COMPLETING INTERVIEW

COMMENTS ABOUT INTERVIEW:

COMMENTS ON SPECIFIC QUESTIONS:

ANY OTHER COMMENTS:

SUPERVISOR'S OBSERVATIONS

EDITOR'S OBSERVATIONS

INSTRUCTIONS:

ONLY ONE CODE SHOULD APPEAR IN ANY BOX.
COLUMN 1 REQUIRES A CODE IN EVERY MONTH.

CODES FOR EACH COLUMN:

COLUMN 1: BIRTHS, PREGNANCIES, CONTRACEPTIVE USE (2)

B BIRTHS
P PREGNANCIES
T TERMINATIONS

0 NO METHOD

1 FEMALE STERILIZATION
2 MALE STERILIZATION
3 IUD
4 INJECTABLES
5 IMPLANTS
6 PILL
7 CONDOM
8 FEMALE CONDOM
9 EMERGENCY CONTRACEPTION
J STANDARD DAYS METHOD
K LACTATIONAL AMENORRHEA METHOD
L RHYTHM METHOD

M WITHDRAWAL
X OTHER MODERN METHOD
Y OTHER TRADITIONAL METHOD

COLUMN 2: DISCONTINUATION OF CONTRACEPTIVE USE

0 INFREQUENT SEX/HUSBAND AWAY
1 BECAME PREGNANT WHILE USING
2 WANTED TO BECOME PREGNANT
3 HUSBAND/PARTNER DISAPPROVED
4 WANTED MORE EFFECTIVE METHOD
5 SIDE EFFECTS/HEALTH CONCERNS

6 LACK OF ACCESS/TOO FAR
7 COSTS TOO MUCH
8 INCONVENIENT TO USE
F UP TO GOD/FATALISTIC
A DIFFICULT TO GET PREGNANT/MENOPAUSAL
D MARITAL DISSOLUTION/SEPARATION
X OTHER

____
(SPECIFY)

Z DON'T KNOW

Year				COL. 1	COL. 2
2019	02	FEB	01		
	01	JAN	02		
2018	12	DEC	03		
	11	NOV	04		
	10	OCT	05		
	09	SEP	06		
	08	AUG	07		
	07	JUL	08		
	06	JUN	09		
	05	MAY	10		
	04	APR	11		
	03	MAR	12		
	02	FEB	13		
	01	JAN	14		
2017	12	DEC	15		
	11	NOV	16		
	10	OCT	17		
	09	SEP	18		
	08	AUG	19		
	07	JUL	20		
	06	JUN	21		
	05	MAY	22		
	04	APR	23		
	03	MAR	24		
	02	FEB	25		
	01	JAN	26		
2016	12	DEC	27		
	11	NOV	28		
	10	OCT	29		
	09	SEP	30		
	08	AUG	31		
	07	JUL	32		
	06	JUN	33		
	05	MAY	34		
	04	APR	35		
	03	MAR	36		
	02	FEB	37		
	01	JAN	38		
2015	12	DEC	39		
	11	NOV	40		
	10	OCT	41		
	09	SEP	42		
	08	AUG	43		
	07	JUL	44		
	06	JUN	45		
	05	MAY	46		
	04	APR	47		
	03	MAR	48		
	02	FEB	49		
	01	JAN	50		
2014	12	DEC	51		
	11	NOV	52		
	10	OCT	53		
	09	SEP	54		
	08	AUG	55		
	07	JUL	56		
	06	JUN	57		
	05	MAY	58		
	04	APR	59		
	03	MAR	60		
	02	FEB	61		
	01	JAN	62		
2013	12	DEC	63		
	11	NOV	64		
	10	OCT	65		
	09	SEP	66		
	08	AUG	67		
	07	JUL	68		
	06	JUN	69		
	05	MAY	70		
	04	APR	71		
	03	MAR	72		
	02	FEB	73		
	01	JAN	74		

FORMATTING DATE: 07 Jun 2017
ENGLISH LANGUAGE: 26 May 2018

NIGERIA DEMOGRAPHIC AND HEALTH SURVEY - 2018
MAN'S QUESTIONNAIRE

NIGERIA
NATIONAL POPULATION COMMISSION

IDENTIFICATION

STATE ______

LOCAL GOVT. AREA ______

LOCALITY ______

ENUMERATION AREA ______

NAME OF HOUSEHOLD HEAD ______

CLUSTER NUMBER . . . .

HOUSEHOLD NUMBER . . . .

NAME AND LINE NUMBER OF MAN ______

INTERVIEWER VISITS

	1	2	3	FINAL VISIT
DATE	______	______	______	DAY<br>MONTH<br>YEAR
INTERVIEWER'S NAME	______	______	______	INT. NO.
RESULT*	______	______	______	RESULT*
NEXT VISIT: DATE<br>TIME	______<br>______	______<br>______		TOTAL NUMBER OF VISITS

*RESULT CODES: 1 COMPLETED; 2 NOT AT HOME; 3 POSTPONED; 4 REFUSED; 5 PARTLY COMPLETED; 6 INCAPACITATED; 7 OTHER ______ (SPECIFY)

LANGUAGE OF QUESTIONNAIRE** 0 1

LANGUAGE OF INTERVIEW**

NATIVE LANGUAGE OF RESPONDENT**

TRANSLATOR USED (YES = 1, NO = 2)

LANGUAGE OF QUESTIONNAIRE** ENGLISH

**LANGUAGE CODES:
01 ENGLISH
02 HAUSA
03 YORUBA
04 IGBO

SUPERVISOR		FIELD EDITOR	
NAME	NUMBER	NAME	NUMBER

INTRODUCTION AND CONSENT

Hello. My name is ______________________________. I am working with Nigeria Population Commission. We are conducting a survey about health and other topics all over Nigeria. The information we collect will help the government to plan health services. Your household was selected for the survey. The questions usually take about 20 minutes. All of the answers you give will be confidential and will not be shared with anyone other than members of our survey team. You don't have to be in the survey, but we hope you will agree to answer the questions since your views are important. If I ask you any question you don't want to answer, just let me know and I will go on to the next question or you can stop the interview at any time.

In case you need more information about the survey, you may contact the person listed on the card that has already been given to your household.

Do you have any questions?
May I begin the interview now?

SIGNATURE OF INTERVIEWER ______________________________ DATE ________________

RESPONDENT AGREES TO BE INTERVIEWED . . 1 ↓

RESPONDENT DOES NOT AGREE TO BE INTERVIEWED . . 2 → END

SECTION 1. RESPONDENT'S BACKGROUND

NO.	QUESTIONS AND FILTERS	CODING CATEGORIES	SKIP
101	RECORD THE TIME.	HOURS . . . . [ ][ ]<br>MINUTES . . . . [ ][ ]	
102	How long have you been living continuously in (NAME OF CURRENT CITY, TOWN OR VILLAGE OF RESIDENCE)?<br><br>IF LESS THAN ONE YEAR, RECORD '00' YEARS.	YEARS . . . . [ ][ ]<br><br>ALWAYS . . . . 95<br>VISITOR 96	95, 96 → 105
103	Just before you moved here, did you live in a city, in a town, or in a rural area?	CITY . . . . 1<br>TOWN . . . . 2<br>RURAL AREA 3	
104	Before you moved here, which state did you live in?	ABIA . . . . 01<br>ADAMAWA . . . . 02<br>AKWA IBOM . . . . 03<br>ANAMBRA . . . . 04<br>BAUCHI . . . . 05<br>BAYELSA . . . . 06<br>BENUE . . . . 07<br>BORNO . . . . 08<br>CROSS RIVER . . . . 09<br>DELTA . . . . 10<br>EBONYI . . . . 11<br>EDO . . . . 12<br>EKITI . . . . 13<br>ENUGU . . . . 14<br>FCT-ABUJA . . . . 15<br>GOMBE . . . . 16<br>IMO . . . . 17<br>JIGAWA . . . . 18<br>KADUNA . . . . 19<br>KANO . . . . 20<br>KATSINA . . . . 21<br>KEBBI . . . . 22<br>KOGI . . . . 23<br>KWARA . . . . 24<br>LAGOS . . . . 25<br>NASARAWA 26	

NO.	QUESTIONS AND FILTERS	CODING CATEGORIES	SKIP
		NIGER . . . . . 27<br>OGUN . . . . . 28<br>ONDO . . . . . 29<br>OSUN . . . . . 30<br>OYO . . . . . 31<br>PLATEAU . . . . . 32<br>RIVERS . . . . . 33<br>SOKOTO . . . . . 34<br>TARABA . . . . . 35<br>YOBE . . . . . 36<br>ZAMFARA . . . . . 37<br><br>OUTSIDE OF NIGERIA 96	
105	In what month and year were you born?	MONTH . . . . . [ ][ ]<br><br>DON'T KNOW MONTH . . . . . 98<br><br>YEAR . . . . . [ ][ ][ ][ ]<br><br>DON'T KNOW YEAR 9998	
106	How old were you at your last birthday?<br><br>COMPARE AND CORRECT 105 AND/OR 106 IF INCONSISTENT.	AGE IN COMPLETED YEARS . . . . . [ ][ ]	
107	Have you ever attended school?	YES . . . . . 1<br>NO 2	 → 111
108	What is the highest level of school you attended: primary, secondary, or higher?	PRIMARY . . . . . 1<br>SECONDARY . . . . . 2<br>HIGHER 3	
109	What is the highest (class/year) you completed at that level?<br><br>IF COMPLETED LESS THAN ONE YEAR AT THAT LEVEL, RECORD '00'.	CLASS/YEAR . . . . . [ ][ ]	
110	CHECK 108:<br>PRIMARY OR SECONDARY [ ] ↓	HIGHER [ ]	→ 113
111	Now I would like you to read this sentence to me.<br><br>SHOW CARD TO RESPONDENT.<br><br>IF RESPONDENT CANNOT READ WHOLE SENTENCE,<br>PROBE: Can you read any part of the sentence to me?	CANNOT READ AT ALL . . . . . 1<br>ABLE TO READ ONLY PART OF THE SENTENCE . . . . . 2<br>ABLE TO READ WHOLE SENTENCE . . . . . 3<br>NO CARD WITH REQUIRED LANGUAGE ________ 4<br>(SPECIFY LANGUAGE)<br>BLIND/VISUALLY IMPAIRED 5	

SECTION 1. RESPONDENT'S BACKGROUND

NO.	QUESTIONS AND FILTERS	CODING CATEGORIES	SKIP
112	CHECK 111:<br>CODE '2', '3' OR '4' CIRCLED ☐ ↓<br>CODE '1' OR '5' CIRCLED ☐		→ 114
113	Do you read a newspaper or magazine at least once a week, less than once a week or not at all?	AT LEAST ONCE A WEEK . . . . 1<br>LESS THAN ONCE A WEEK . . . . 2<br>NOT AT ALL 3	
114	Do you listen to the radio at least once a week, less than once a week or not at all?	AT LEAST ONCE A WEEK . . . . 1<br>LESS THAN ONCE A WEEK . . . . 2<br>NOT AT ALL 3	
115	Do you watch television at least once a week, less than once a week or not at all?	AT LEAST ONCE A WEEK . . . . 1<br>LESS THAN ONCE A WEEK . . . . 2<br>NOT AT ALL 3	
116	Do you own a mobile telephone?	YES . . . . 1<br>NO 2	 → 118
117	Do you use your mobile phone for any financial transactions?	YES . . . . 1<br>NO 2	
118	Do you have an account in a bank or other financial institution that you yourself use?	YES . . . . 1<br>NO 2	
119	Have you ever used the internet?	YES . . . . 1<br>NO 2	 → 122
120	In the last 12 months, have you used the internet?<br><br>IF NECESSARY, PROBE FOR USE FROM ANY LOCATION, WITH ANY DEVICE.	YES . . . . 1<br>NO 2	 → 122
121	During the last one month, how often did you use the internet: almost every day, at least once a week, less than once a week, or not at all?	ALMOST EVERY DAY . . . . 1<br>AT LEAST ONCE A WEEK . . . . 2<br>LESS THAN ONCE A WEEK . . . . 3<br>NOT AT ALL 4	
122	What is your religion?	CATHOLIC . . . . 1<br>OTHER CHRISTIAN . . . . 2<br>ISLAM . . . . 3<br>TRADITIONALIST . . . . 4<br><br>OTHER ______ 6<br>(SPECIFY)	
123	What is your ethnic group?	______ ☐☐☐<br>(ETHNIC GROUP)	
124	In the last 12 months, how many times have you been away from home for one or more nights?	NUMBER OF TIMES . . . . ☐☐<br><br>NONE 00	 <br>→ 201
125	In the last 12 months, have you been away from home for more than one month at a time?	YES . . . . 1<br>NO 2	

SECTION 2. REPRODUCTION

NO.	QUESTIONS AND FILTERS	CODING CATEGORIES	SKIP
201	Now I would like to ask about any children you have had during your life. I am interested in all of the children that are biologically yours, even if they are not legally yours or do not have your last name. Have you ever fathered any children with any woman?	YES . . . 1<br>NO . . . 2<br>DON'T KNOW . . . 8	 (NO, DON'T KNOW) → 206
202	Do you have any sons or daughters that you have fathered who are now living with you?	YES . . . 1<br>NO . . . 2	 → 204
203	a) How many sons live with you?<br>b) And how many daughters live with you?<br>IF NONE, RECORD '00'.	a) SONS AT HOME . . . [ ][ ]<br>b) DAUGHTERS AT HOME . . . [ ][ ]	
204	Do you have any sons or daughters that you have fathered who are alive but do not live with you?	YES . . . 1<br>NO . . . 2	 → 206
205	a) How many sons are alive but do not live with you?<br>b) And how many daughters are alive but do not live with you?<br>IF NONE, RECORD '00'.	a) SONS ELSEWHERE . . . [ ][ ]<br>b) DAUGHTERS ELSEWHERE . . . [ ][ ]	
206	Have you ever fathered a son or a daughter who was born alive but later died?<br>IF NO, PROBE: Any baby who cried, who made any movement, sound, or effort to breathe, or who showed any other signs of life even if for a very short time?	YES . . . 1<br>NO . . . 2<br>DON'T KNOW . . . 8	 (NO, DON'T KNOW) → 208
207	a) How many boys have died?<br>b) And how many girls have died?<br>IF NONE, RECORD '00'.	a) BOYS DEAD . . . [ ][ ]<br>b) GIRLS DEAD . . . [ ][ ]	
208	SUM ANSWERS TO 203, 205, AND 207, AND ENTER TOTAL. IF NONE, RECORD '00'.	TOTAL CHILDREN . . . [ ][ ]	
209	CHECK 208:<br>HAS HAD MORE THAN ONE CHILD [ ] ↓<br>HAS HAD ONLY ONE CHILD [ ] → 211<br>HAS NOT HAD ANY CHILDREN [ ] → 301		211<br>301
210	Did all of the children you have fathered have the same biological mother?	YES . . . 1<br>NO . . . 2	
211	CHECK 208:<br>HAS HAD MORE THAN ONE CHILD [ ] ↓ a) How old were you when your first child was born?<br>HAS HAD ONLY ONE CHILD [ ] ↓ b) How old were you when your child was born?	AGE IN YEARS . . . [ ][ ]	
212	CHECK 203 AND 205:<br>AT LEAST ONE LIVING CHILD [ ] ↓<br>NO LIVING CHILDREN [ ] → 301		301

NO.	QUESTIONS AND FILTERS	CODING CATEGORIES	SKIP
213	CHECK 203 AND 205:<br>MORE THAN ONE LIVING CHILD [ ] ↓ / ONLY ONE LIVING CHILD [ ] ↓<br>a) How old is your youngest child?<br>b) How old is your child?	AGE IN YEARS . . . . [ ][ ]	
214	CHECK 213:<br>(YOUNGEST) CHILD IS AGE 0-2 YEARS [ ] ↓<br>(YOUNGEST) CHILD IS AGE 3 YEARS OR OLDER [ ]		→ 301
215	CHECK 203 AND 205:<br>MORE THAN ONE LIVING CHILD [ ] ↓ / ONLY ONE LIVING CHILD [ ] ↓<br>a) What is the name of your youngest child?<br>b) What is the name of your child?	________________<br>(NAME OF (YOUNGEST) CHILD)	
216	When (NAME)'s mother was pregnant with (NAME), did she have any antenatal check-ups?	YES . . . . 1<br>NO . . . . 2<br>DON'T KNOW 8	NO, DON'T KNOW → 218
217	Were you ever present during any of those antenatal check-ups?	PRESENT . . . . 1<br>NOT PRESENT 2	
218	Was (NAME) born in a hospital or health facility?	HOSPITAL/HEALTH FACILITY . . . . 1<br>OTHER 2	
219	When a child has diarrhea, how much should he or she be given to drink: more than usual, about the same as usual, less than usual, or nothing to drink at all?	MORE THAN USUAL . . . . 1<br>ABOUT THE SAME . . . . 2<br>LESS THAN USUAL . . . . 3<br>NOTHING TO DRINK . . . . 4<br>DON'T KNOW 8	

SECTION 3. CONTRACEPTION

301	Now I would like to talk about family planning - the various ways or methods that a couple can use to delay or avoid a pregnancy. Have you ever heard of (METHOD)?	
01	Female Sterilization.<br>PROBE: Women can have an operation to avoid having any more children.	YES . . . . . . 1<br>NO 2
02	Male Sterilization.<br>PROBE: Men can have an operation to avoid having any more children.	YES . . . . . . 1<br>NO 2
03	IUD.<br>PROBE: Women can have a loop or coil placed inside them by a doctor or a nurse which can prevent pregnancy for one or more years.	YES . . . . . . 1<br>NO 2
04	Injectables.<br>PROBE: Women can have an injection by a health provider that stops them from becoming pregnant for one or more months.	YES . . . . . . 1<br>NO 2
05	Implants.<br>PROBE: Women can have one or more small rods placed in their upper arm by a doctor or nurse which can prevent pregnancy for one or more years.	YES . . . . . . 1<br>NO 2
06	Pill.<br>PROBE: Women can take a pill every day to avoid becoming pregnant.	YES . . . . . . 1<br>NO 2
07	Condom.<br>PROBE: Men can put a rubber sheath on their penis before sexual intercourse.	YES . . . . . . 1<br>NO 2
08	Female Condom.<br>PROBE: Women can place a sheath in their vagina before sexual intercourse.	YES . . . . . . 1<br>NO 2
09	Emergency Contraception.<br>PROBE: As an emergency measure, within three days after they have unprotected sexual intercourse, women can take special pills to prevent pregnancy.	YES . . . . . . 1<br>NO 2
10	Standard Days Method.<br>PROBE: A woman uses a string of colored beads to know the days she can get pregnant. On the days she can get pregnant, she uses a condom or does not have sexual intercourse.	YES . . . . . . 1<br>NO 2
11	Lactational Amenorrhea Method (LAM).<br>PROBE: Up to six months after childbirth, before the menstrual period has returned, women use a method requiring frequent breastfeeding day and night.	YES . . . . . . 1<br>NO 2
12	Rhythm Method.<br>PROBE: To avoid pregnancy, women do not have sexual intercourse on the days of the month they think they can get pregnant.	YES . . . . . . 1<br>NO 2
13	Withdrawal.<br>PROBE: Men can be careful and pull out before climax.	YES . . . . . . 1<br>NO 2
14	Have you heard of any other ways or methods that women or men can use to avoid pregnancy?	YES, MODERN METHOD<br>__________ A<br>(SPECIFY)<br>YES, TRADITIONAL METHOD<br>__________ B<br>(SPECIFY)<br>NO . . . . . . Y

SECTION 3. CONTRACEPTION

NO.	QUESTIONS AND FILTERS	CODING CATEGORIES	SKIP
302	In the last few months have you:<br>a) Heard about family planning on the radio?<br>b) Seen anything about family planning on the television?<br>c) Read about family planning in a newspaper or magazine?<br>d) Received a voice or text message about family planning on a mobile phone?<br>e) Read/heard from social media (facebook, twitter, etc.)?<br>f) Read about family planning in a poster?<br>g) Read about family planning in a leaflet or brochures?<br>h) Heard about family planning from town crier?<br>i) Heard about family planning from mobile public announcement?	YES NO<br>a) RADIO . . . . . 1 2<br>b) TELEVISION . . . . . 1 2<br>c) NEWSPAPER OR MAGAZINE . . . . . 1 2<br>d) MOBILE PHONE . . . . . 1 2<br>e) SOCIAL MEDIA . . . . . 1 2<br>f) POSTER . . . . . 1 2<br>g) LEAFLET OR BROCHURE . . . . . 1 2<br>h) TOWN CRIER . . . . . 1 2<br>i) MOBILE PUBLIC ANNOUNCEMEN . . 1 2	
302A	CHECK 302:<br>AT LEAST ONE `YES' (HAS HEARD OR READ MESSAGE) ☐ ↓<br>NOT A SINGLE `YES' (HAS NOT HEARD OR READ MESSAGE) ☐		→ 303
302B	Please tell me which family planning messages you have heard or seen in the past few months?<br><br>PROBE: Any others?	AS FOR ME AND MY PARTNER WE `DEY KAMPE' WITH FEMALE CONDOM . . . . . A<br>UNSPACED CHILDREN MAKES THE GOING TOUGH FOR THE LOVE OF YOUR FAMILY, GO FOR CHILD SPACING TODAY . . . . . B<br>WELL-SPACED CHILDREN ARE EVERY PARENT'S JOY . . . . . C<br>IT'S NOT TOO LATE TO PREVENT UNWANTED PREGNANCY . . . . . D<br>WHY IS YOUR WIFE LOOKING SO GOO . . . . . E<br>OTHER ________ X<br>(SPECIFY)	
303	In the last few months, have you discussed family planning with a health worker or health professional?	YES . . . . . 1<br>NO 2	
304	Now I would like to ask you about a woman's risk of pregnancy. From one menstrual period to the next, are there certain days when a woman is more likely to become pregnant when she has sexual relations?	YES . . . . . 1<br>NO . . . . . 2<br>DON'T KNOW 8	 NO, DON'T KNOW → 306
305	Is this time just before her period begins, during her period, right after her period has ended, or halfway between two periods?	JUST BEFORE HER PERIOD BEGINS . . . . . 1<br>DURING HER PERIOD . . . . . 2<br>RIGHT AFTER HER PERIOD HAS ENDEI . . . . . 3<br>HALFWAY BETWEEN TWO PERIODS . . . . . 4<br>OTHER ________ 6<br>(SPECIFY)<br>DON'T KNOW 8	
306	After the birth of a child, can a woman become pregnant before her menstrual period has returned?	YES . . . . . 1<br>NO . . . . . 2<br>DON'T KNOW 8	
307	I will now read you some statements about contraception. Please tell me if you agree or disagree with each one.<br>a) Contraception is a woman's concern and a man should not have to worry about it.<br>b) Women who use contraception may become promiscuous.	AGREE DIS-AGREE DK<br>a) CONTRACEPTION WOMAN'S CONCERN 1 2 8<br>b) WOMEN MAY BECOME PROMISCUOUS 1 2 8	

SECTION 4. MARRIAGE AND SEXUAL ACTIVITY

NO.	QUESTIONS AND FILTERS	CODING CATEGORIES	SKIP
401	Are you currently married or living together with a woman as if married?	YES, CURRENTLY MARRIED . . . . . 1<br>YES, LIVING WITH A WOMAN . . . . . 2<br>NO, NOT IN UNION 3	1, 2 → 404
402	Have you ever been married or lived together with a woman as if married?	YES, FORMERLY MARRIED . . . . . 1<br>YES, LIVED WITH A WOMAN . . . . . 2<br>NO 3	3 → 413
403	What is your marital status now: are you widowed, divorced, or separated?	WIDOWED . . . . . 1<br>DIVORCED . . . . . 2<br>SEPARATED 3	1, 2, 3 → 410
404	Is your (wife/partner) living with you now or is she staying elsewhere?	LIVING WITH HIM . . . . . 1<br>STAYING ELSEWHERE 2	
405	Do you have other wives or do you live with other women as if married?	YES (MORE THAN ONE WIFE) . . . . . 1<br>NO (ONLY ONE WIFE) 2	2 → 407
406	Altogether, how many wives or live-in partners do you have?	TOTAL NUMBER OF WIVES AND LIVE-IN PARTNERS . . . . . [ ][ ]	
407	CHECK 405:<br>ONE WIFE/ PARTNER [ ] ↓ — a) Please tell me the name of (your wife/the woman you are living with as if married).<br>MORE THAN ONE WIFE/ PARTNER [ ] ↓ — b) Please tell me the name of each of your wives or each woman you are living with as if married.<br>RECORD THE NAME AND THE LINE NUMBER FROM THE HOUSEHOLD QUESTIONNAIRE FOR EACH WIFE AND LIVE-IN PARTNER.<br>IF A WOMAN IS NOT LISTED IN THE HOUSEHOLD, RECORD '00'.		
408	ASK 408 FOR EACH PERSON.		

NAME	LINE NUMBER	408<br>How old was (NAME) on her last birthday?<br>AGE
________	[ ][ ]	[ ][ ]
________	[ ][ ]	[ ][ ]
________	[ ][ ]	[ ][ ]
________	[ ][ ]	[ ][ ]

NO.	QUESTIONS AND FILTERS	CODING CATEGORIES	SKIP
409	CHECK 407:<br>ONE WIFE/ PARTNER [ ] ↓<br>MORE THAN ONE WIFE/ PARTNER [ ]		MORE THAN ONE WIFE/PARTNER → 411
410	Have you been married or lived with a woman only once or more than once?	MORE THAN ONCE . . . . . 1<br>ONLY ONCE 2	

NO.	QUESTIONS AND FILTERS	CODING CATEGORIES	SKIP
411	CHECK 405 AND 410:<br>BOTH ARE CODE '2' ☐ / OTHER ☐<br>a) In what month and year did you start living with your (wife/partner)?<br>b) Now I would like to ask about your first (wife/partner). In what month and year did you start living with her?	MONTH . . . . [ ][ ]<br>DON'T KNOW MONTH . . . . 98<br>YEAR . . . . [ ][ ][ ][ ]<br>DON'T KNOW YEAR 9998	→ 413
412	How old were you when you first started living with her?	AGE . . . . [ ][ ]	
413	**CHECK FOR PRESENCE OF OTHERS. BEFORE CONTINUING, MAKE EVERY EFFORT TO ENSURE PRIVACY.**		
414	I would like to ask some questions about sexual activity in order to gain a better understanding of some important life issues. Let me assure you again that your answers are completely confidential and will not be told to anyone. If we should come to any question that you don't want to answer, just let me know and we will go to the next question. How old were you when you had sexual intercourse for the very first time?	NEVER HAD SEXUAL INTERCOURSE . . . . 00<br>AGE IN YEARS . . . . [ ][ ]	00 → 501
415	I would like to ask you about your recent sexual activity. When was the last time you had sexual intercourse?<br>IF LESS THAN 12 MONTHS, ANSWER MUST BE RECORDED IN DAYS, WEEKS OR MONTHS. IF 12 MONTHS (ONE YEAR) OR MORE, ANSWER MUST BE RECORDED IN YEARS.	DAYS AGO . . . . 1 [ ][ ]<br>WEEKS AGO . . . . 2 [ ][ ]<br>MONTHS AGO . . . . 3 [ ][ ]<br>YEARS AGO . . . . 4 [ ][ ]	1–3 → 417<br>4 → 427

		LAST SEXUAL PARTNER	SECOND-TO-LAST SEXUAL PARTNER	THIRD-TO-LAST SEXUAL PARTNER
416	When was the last time you had sexual intercourse with this person?		DAYS AGO . . 1<br>WEEKS AGO . . 2<br>MONTHS AGO . . 3	DAYS AGO . . 1<br>WEEKS AGO . . 2<br>MONTHS AGO . . 3
417	The last time you had sexual intercourse with this person, was a condom used?	YES . . . . 1<br>NO . . . . 2<br>(SKIP TO 419)	YES . . . . 1<br>NO . . . . 2<br>(SKIP TO 419)	YES . . . . 1<br>NO . . . . 2<br>(SKIP TO 419)
418	Was a condom used every time you had sexual intercourse with this person in the last 12 months?	YES . . . . 1<br>NO 2	YES . . . . 1<br>NO 2	YES . . . . 1<br>NO 2
419	What was your relationship to this person with whom you had sexual intercourse?<br><br>IF GIRLFRIEND: Were you living together as if married?<br><br>IF YES, RECORD '2'.<br>IF NO, RECORD '3'.	WIFE . . . . 1<br>LIVE-IN PARTNER . . . . . 2<br>GIRLFRIEND NOT LIVING WITH RESPONDENT . . . . . 3<br>CASUAL ACQUAINTANCE . . 4<br>CLIENT/SEX WORKER . . 5<br>OTHER ______ 6<br>(SPECIFY)	WIFE . . . . 1<br>LIVE-IN PARTNER . . . . . 2<br>GIRLFRIEND NOT LIVING WITH RESPONDENT . . . . . 3<br>CASUAL ACQUAINTANCE . . 4<br>CLIENT/SEX WORKER . . 5<br>OTHER ______ 6<br>(SPECIFY)	WIFE . . . . 1<br>LIVE-IN PARTNER . . . . . 2<br>GIRLFRIEND NOT LIVING WITH RESPONDENT . . . . . 3<br>CASUAL ACQUAINTANCE . . 4<br>CLIENT/SEX WORKER . . 5<br>OTHER ______ 6<br>(SPECIFY)
420	How long ago did you first have sexual intercourse with this person?	DAYS AGO . . 1<br>WEEKS AGO . . 2<br>MONTHS AGO . . 3<br>YEARS AGO . . 4	DAYS AGO . . 1<br>WEEKS AGO . . 2<br>MONTHS AGO . . 3<br>YEARS AGO . . 4	DAYS AGO . . 1<br>WEEKS AGO . . 2<br>MONTHS AGO . . 3<br>YEARS AGO . . 4
421	How many times during the last 12 months did you have sexual intercourse with this person?<br>IF NON-NUMERIC ANSWER, PROBE TO GET AN ESTIMATE. IF NUMBER OF TIMES IS 95 OR MORE, RECORD '95'.	NUMBER OF TIMES	NUMBER OF TIMES	NUMBER OF TIMES . . . . .
422	How old is this person?	AGE OF PARTNER<br><br>DON'T KNOW 98	AGE OF PARTNER<br><br>DON'T KNOW 98	AGE OF PARTNER<br><br>DON'T KNOW 98
423	Apart from this person, have you had sexual intercourse with any other person in the last 12 months?	YES . . . . 1<br>(GO BACK TO 416 IN NEXT COLUMN)<br>NO . . . . 2<br>(SKIP TO 425)	YES . . . . 1<br>(GO BACK TO 416 IN NEXT COLUMN)<br>NO . . . . 2<br>(SKIP TO 425)	
424	In total, with how many different people have you had sexual intercourse in the last 12 months?<br>IF NON-NUMERIC ANSWER, PROBE TO GET AN ESTIMATE. IF NUMBER OF PARTNERS IS 95 OR MORE, RECORD '95'.			NUMBER OF PARTNERS LAST 12 MONTHS . .<br><br>DON'T KNOW 98

NO.	QUESTIONS AND FILTERS	CODING CATEGORIES	SKIP
425	CHECK 419 (ALL COLUMNS):<br>AT LEAST ONE PARTNER IS A SEX WORKER ☐ ↓<br>NO PARTNERS ARE SEX WORKERS ☐ → 427		427
426	CHECK 419 AND 417 (ALL COLUMNS):<br>CONDOM USED WITH EVERY SEX WORKER ☐ → 430<br>OTHER ☐ → 431		430<br>431
427	In the last 12 months, did you pay anyone in exchange for having sexual intercourse?	YES . . . . 1<br>NO 2	1 → 429
428	Have you ever paid anyone in exchange for having sexual intercourse?	YES . . . . 1<br>NO 2	1, 2 → 431
429	The last time you paid someone in exchange for having sexual intercourse, was a condom used?	YES . . . . 1<br>NO 2	2 → 431
430	Was a condom used during sexual intercourse every time you paid someone in exchange for having sexual intercourse in the last 12 months?	YES . . . . 1<br>NO . . . . 2<br>DON'T KNOW 8	
431	In the past 12 months have you given any gifts or other goods in order to have sex or to become sexually involved with anyone?	YES . . . . 1<br>NO 2	1 → 433
432	Have you ever given any gifts or other goods in order to have sex or to become sexually involved with anyone?	YES . . . . 1<br>NO 2	
433	In total, with how many different people have you had sexual intercourse in your lifetime?<br><br>IF NON-NUMERIC ANSWER, PROBE TO GET AN ESTIMATE. IF NUMBER OF PARTNERS IS 95 OR MORE, RECORD '95'.	NUMBER OF PARTNERS IN LIFETIME . . . . ☐☐<br><br>DON'T KNOW 98	
434	CHECK 417: MOST RECENT PARTNER (FIRST COLUMN)<br>NOT ASKED ☐ → 438<br>CONDOM USED ☐ ↓<br>NO CONDOM USED ☐ → 438		438<br>438
435	You told me that a condom was used the last time you had sex. What is the brand name of the condom used at that time?<br><br>IF BRAND NOT KNOWN, ASK TO SEE THE PACKAGE.	MALE CONDOMS . . . . 01<br>GOLD CIRCLE . . . . 02<br>DUREX . . . . 03<br>ROUGH RIDER . . . . 04<br>TWIN LOTUS . . . . 05<br>PLAIN CONDOMS . . . . 06<br>GO FLEX . . . . 07<br><br>OTHER ______ 96<br>(SPECIFY)<br>DON'T KNOW 98	

NO.	QUESTIONS AND FILTERS	CODING CATEGORIES	SKIP
436	From where did you obtain the condom the last time?<br><br>PROBE TO IDENTIFY TYPE OF SOURCE.<br><br>IF UNABLE TO DETERMINE IF PUBLIC OR PRIVATE SECTOR, WRITE THE NAME OF THE PLACE.<br><br>__________<br>(NAME OF PLACE)	**PUBLIC SECTOR**<br>GOVERNMENT HOSPITAL . . . . 11<br>GOVERNMENT HEALTH CENTER . . . . 12<br>FAMILY PLANNING CLINIC . . . . 13<br>MOBILE CLINIC . . . . 14<br>FIELDWORKER . . . . 15<br>OTHER PUBLIC SECTOR<br>__________ 16<br>(SPECIFY)<br><br>**PRIVATE MEDICAL SECTOR**<br>PRIVATE HOSPITAL/CLINIC . . . . 21<br>PHARMACY . . . . 22<br>CHEMIST/PMS STORE . . . . 23<br>PRIVATE DOCTOR . . . . 24<br>MOBILE CLINIC . . . . 25<br>FIELDWORKER . . . . 26<br>OTHER PRIVATE MEDICAL SECTOR<br>__________ 27<br>(SPECIFY)<br><br>**OTHER SOURCE**<br>SHOP . . . . 31<br>CHURCH . . . . 32<br>FRIEND/RELATIVE . . . . 33<br>NGO . . . . 34<br><br>OTHER __________ 96<br>(SPECIFY)<br>DON'T KNOW 98	
437	The last time you had sex did you or your partner use any method other than a condom to avoid or prevent a pregnancy?	YES . . . . 1<br>NO . . . . 2<br>DON'T KNOW 8	YES → 439<br>NO, DON'T KNOW → 440
438	The last time you had sex did you or your partner use any method to avoid or prevent a pregnancy?	YES . . . . 1<br>NO . . . . 2<br>DON'T KNOW 8	 NO, DON'T KNOW → 440
439	What method did you or your partner use?<br><br>PROBE: Did you or your partner use any other method to prevent pregnancy?<br><br>RECORD ALL MENTIONED.	FEMALE STERILIZATION . . . . A<br>MALE STERILIZATION . . . . B<br>IUD . . . . C<br>INJECTABLES . . . . D<br>IMPLANTS . . . . E<br>PILL . . . . F<br>CONDOM . . . . G<br>FEMALE CONDOM . . . . H<br>EMERGENCY CONTRACEPTION . . . . I<br>STANDARD DAYS METHOD . . . . J<br>LACTATIONAL AMENORRHEA METHOD . . . . K<br>RHYTHM METHOD . . . . L<br>WITHDRAWAL . . . . M<br>OTHER MODERN METHOD . . . . X<br>OTHER TRADITIONAL METHOD . . . . Y	→ 501
440	Do you know of a place where you can obtain a method of family planning?	YES . . . . 1<br>NO 2	

SECTION 5. FERTILITY PREFERENCES

NO.	QUESTIONS AND FILTERS	CODING CATEGORIES	SKIP
501	CHECK 401:<br>CURRENTLY MARRIED OR LIVING WITH A PARTNER ☐ ↓	NOT CURRENTLY MARRIED AND NOT LIVING WITH A PARTNER ☐	→ 514
502	CHECK 439:<br>MAN NOT STERILIZED ☐ ↓	MAN STERILIZED ☐	→ 514
503	CHECK 407:<br>ONE WIFE/ PARTNER ☐ ↓	MORE THAN ONE WIFE/ PARTNER ☐	→ 509
504	Is your (wife/partner) currently pregnant?	YES . . . . . 1<br>NO . . . . . 2<br>DON'T KNOW 8	 2, 8 → 507
505	Now I have some questions about the future. After the child you and your (wife/partner) are expecting now, would you like to have another child, or would you prefer not to have any more children?	HAVE ANOTHER CHILD . . . . . 1<br>NO MORE . . . . . 2<br>UNDECIDED/DON'T KNOW 8	 2, 8 → 514
506	After the birth of the child you are expecting now, how long would you like to wait before the birth of another child?	MONTHS . . . . . 1 ☐☐<br>YEARS . . . . . 2 ☐☐<br>SOON/NOW . . . . . 993<br>OTHER ______ 996<br>(SPECIFY)<br>DON'T KNOW 998	→ 514
507	CHECK 208:<br>HAS FATHERED CHILDREN ☐ ↓ a) Now I have some questions about the future. Would you like to have another child, or would you prefer not to have any more children?<br>HAS NOT FATHERED CHILDREN ☐ ↓ b) Now I have some questions about the future. Would you like to have a child, or would you prefer not to have any children?	HAVE (A/ANOTHER) CHILD . . . . . 1<br>NO MORE/NONE . . . . . 2<br>SAYS COUPLE CAN'T GET PREGNANT . . . . . 3<br>WIFE/PARTNER STERILIZED . . . . . 4<br>UNDECIDED/DON'T KNOW 8	 2, 3, 4, 8 → 514
508	CHECK 208:<br>HAS FATHERED CHILDREN ☐ ↓ a) How long would you like to wait from now before the birth of another child?<br>HAS NOT FATHERED CHILDREN ☐ ↓ b) How long would you like to wait from now before the birth of a child?	MONTHS . . . . . 1 ☐☐<br>YEARS . . . . . 2 ☐☐<br>SOON/NOW . . . . . 993<br>SAYS COUPLE CAN'T GET PREGNANT . . . . . 994<br>OTHER ______ 996<br>(SPECIFY)<br>DON'T KNOW 998	→ 514
509	Are any of your (wives/partners) currently pregnant?	YES . . . . . 1<br>NO . . . . . 2<br>DON'T KNOW 8	 2, 8 → 512

SECTION 5. FERTILITY PREFERENCES

NO.	QUESTIONS AND FILTERS	CODING CATEGORIES	SKIP
510	Now I have some questions about the future. After the (child/children) you and your (wives/partners) are expecting now, would you like to have another child, or would you prefer not to have any more children?	HAVE ANOTHER CHILD . . . . 1<br>NO MORE . . . . 2<br>UNDECIDED/DON'T KNOW 8	2, 8 → 514
511	After the birth of the child you are expecting now, how long would you like to wait before the birth of another child?	MONTHS . . . . 1 [ ][ ]<br>YEARS . . . . 2 [ ][ ]<br>SOON/NOW . . . . .993<br>OTHER ______________________ 996<br>(SPECIFY)<br>DON'T KNOW 998	→ 514
512	CHECK 208:<br>HAS FATHERED CHILDREN [ ] ¦ HAS NOT FATHERED CHILDREN [ ]<br>a) Now I have some questions about the future. Would you like to have another child, or would you prefer not to have any more children?<br>b) Now I have some questions about the future. Would you like to have a child, or would you prefer not to have any children?	HAVE (A/ANOTHER) CHILD . . . . 1<br>NO MORE/NONE . . . . 2<br>SAYS COUPLE CAN'T GET PREGNANT . . . . 3<br>(WIFE/WIVES/PARTNER(S)) STERILIZED . . . . . 4<br>UNDECIDED/DON'T KNOW 8	2, 3, 4, 8 → 514
513	CHECK 208:<br>HAS FATHERED CHILDREN [ ] ¦ HAS NOT FATHERED CHILDREN [ ]<br>a) How long would you like to wait from now before the birth of another child?<br>b) How long would you like to wait from now before the birth of a child?	MONTHS . . . . 1 [ ][ ]<br>YEARS . . . . 2 [ ][ ]<br>SOON/NOW . . . . .993<br>SAYS COUPLE<br>CAN'T GET PREGNANT . . . . 994<br>OTHER ______________________ 996<br>(SPECIFY)<br>DON'T KNOW 998	
514	CHECK 203 AND 205:<br>HAS LIVING CHILDREN [ ] ¦ NO LIVING CHILDREN [ ]<br>a) If you could go back to the time you did not have any children and could choose exactly the number of children to have in your whole life, how many would that be?<br>b) If you could choose exactly the number of children to have in your whole life, how many would that be?<br>PROBE FOR A NUMERIC RESPONSE.	NONE . . . . 00<br>NUMBER . . . . [ ][ ]<br>OTHER ______________________ 96<br>(SPECIFY)	00 → 601<br>96 → 601
515	How many of these children would you like to be boys, how many would you like to be girls and for how many would it not matter if it's a boy or a girl?	BOYS GIRLS EITHER<br>NUMBER . . [ ][ ] [ ][ ] [ ][ ]<br>OTHER ______________________ 96<br>(SPECIFY)	

SECTION 6. EMPLOYMENT AND GENDER ROLES

NO.	QUESTIONS AND FILTERS	CODING CATEGORIES	SKIP
601	Have you done any work in the last seven days?	YES . . . 1<br>NO . . . 2	1 → 604
602	Although you did not work in the last seven days, do you have any job or business from which you were absent for leave, illness, vacation, or any other such reason?	YES . . . 1<br>NO . . . 2	1 → 604
603	Have you done any work in the last 12 months?	YES . . . 1<br>NO . . . 2	2 → 607
604	What is your occupation? That is, what kind of work do you mainly do?	________<br>________ [ ][ ]<br>________	
605	Do you usually work throughout the year, or do you work seasonally, or only once in a while?	THROUGHOUT THE YEAR . . . 1<br>SEASONALLY/PART OF THE YEAR . . . 2<br>ONCE IN A WHILE . . . 3	
606	Are you paid in cash or kind for this work or are you not paid at all?	CASH ONLY . . . 1<br>CASH AND KIND . . . 2<br>IN KIND ONLY . . . 3<br>NOT PAID . . . 4	
607	CHECK 401:<br>CURRENTLY MARRIED OR LIVING WITH A PARTNER [ ] ↓<br>NOT CURRENTLY MARRIED AND NOT LIVING WITH A PARTNER [ ] → 612		→ 612
608	CHECK 606:<br>CODE '1' OR '2' CIRCLED [ ] ↓<br>OTHER [ ] → 610		→ 610
609	Who usually decides how the money you earn will be used: you, your (wife/partner), or you and your (wife/partner) jointly?	RESPONDENT . . . 1<br>WIFE/PARTNER . . . 2<br>RESPONDENT AND WIFE/PARTNER JOINTLY . . 3<br>OTHER ________ 6<br>(SPECIFY)	
610	Who usually makes decisions about health care for yourself: you, your (wife/partner), you and your (wife/partner) jointly, or someone else?	RESPONDENT . . . 1<br>WIFE/PARTNER . . . 2<br>RESPONDENT AND WIFE/PARTNER JOINTLY . . 3<br>SOMEONE ELSE . . . 4<br>OTHER . . . 6	
611	Who usually makes decisions about making major household purchases?	RESPONDENT . . . 1<br>WIFE/PARTNER . . . 2<br>RESPONDENT AND WIFE/PARTNER JOINTLY . . 3<br>SOMEONE ELSE . . . 4<br>OTHER . . . 6	

NO.	QUESTIONS AND FILTERS	CODING CATEGORIES	SKIP
612	Do you own this or any other house either alone or jointly with someone else?	ALONE ONLY . . . 1<br>JOINTLY ONLY . . . 2<br>BOTH ALONE AND JOINTLY . . . 3<br>DOES NOT OWN . . . 4	 <br><br>4 → 615
613	Do you have a title deed for any house you own?	YES . . . 1<br>NO . . . 2<br>DON'T KNOW . . . 8	 2, 8 → 615
614	Is your name on the title deed?	YES . . . 1<br>NO . . . 2<br>DON'T KNOW . . . 8	
615	Do you own any agricultural or non-agricultural land either alone or jointly with someone else?	ALONE ONLY . . . 1<br>JOINTLY ONLY . . . 2<br>BOTH ALONE AND JOINTLY . . . 3<br>DOES NOT OWN . . . 4	 <br><br>4 → 618
616	Do you have a title deed for any land you own?	YES . . . 1<br>NO . . . 2<br>DON'T KNOW . . . 8	 2, 8 → 618
617	Is your name on the title deed?	YES . . . 1<br>NO . . . 2<br>DON'T KNOW . . . 8	
618	In your opinion, is a husband justified in hitting or beating his wife in the following situations:<br>a) If she goes out without telling him?<br>b) If she neglects the children?<br>c) If she argues with him?<br>d) If she refuses to have sex with him?<br>e) If she burns the food?	YES NO DK<br>a) GOES OUT . . . 1 2 8<br>b) NEGLECTS CHILDREN . . 1 2 8<br>c) ARGUES . . . 1 2 8<br>d) REFUSES SEX . . . 1 2 8<br>e) BURNS FOOD . . . 1 2 8	

SECTION 7. HIV/AIDS

NO.	QUESTIONS AND FILTERS	CODING CATEGORIES	SKIP
701	Now I would like to talk about something else. Have you ever heard of HIV or AIDS?	YES . . . . . 1<br>NO 2	 → 727
702	HIV is the virus that can lead to AIDS. Can people reduce their chance of getting HIV by having just one uninfected sex partner who has no other sex partners?	YES . . . . . 1<br>NO . . . . . 2<br>DON'T KNOW 8	
703	Can people get HIV from mosquito bites?	YES . . . . . 1<br>NO . . . . . 2<br>DON'T KNOW 8	
704	Can people reduce their chance of getting HIV by using a condom every time they have sex?	YES . . . . . 1<br>NO . . . . . 2<br>DON'T KNOW 8	
705	Can people get HIV by sharing food with a person who has HIV?	YES . . . . . 1<br>NO . . . . . 2<br>DON'T KNOW 8	
706	Can people get HIV because of witchcraft or other supernatural means?	YES . . . . . 1<br>NO . . . . . 2<br>DON'T KNOW 8	
707	Is it possible for a healthy-looking person to have HIV?	YES . . . . . 1<br>NO . . . . . 2<br>DON'T KNOW 8	
708	Can HIV be transmitted from a mother to her baby:<br>a) During pregnancy?<br>b) During delivery?<br>c) By breastfeeding?	YES NO DK<br>a) DURING PREGNANCY . . 1 2 8<br>b) DURING DELIVERY . . . . . 1 2 8<br>c) BREASTFEEDING 1 2 8	
709	CHECK 708:<br>AT LEAST ONE 'YES' ☐ ↓ OTHER ☐		→ 720
710	Are there any special drugs that a doctor or a nurse can give to a woman infected with HIV to reduce the risk of transmission to the baby?	YES . . . . . 1<br>NO . . . . . 2<br>DON'T KNOW 8	

NO.	QUESTIONS AND FILTERS	CODING CATEGORIES	SKIP
720	Would you buy fresh vegetables from a shopkeeper or vendor if you knew that this person had HIV?	YES . . . . 1<br>NO . . . . 2<br>DON'T KNOW/NOT SURE/DEPENDS 8	
721	Do you think children living with HIV should be allowed to attend school with children who do not have HIV?	YES . . . . 1<br>NO . . . . 2<br>DON'T KNOW/NOT SURE/DEPENDS 8	
722	Do you think people hesitate to take an HIV test because they are afraid of how other people will react if the test result is positive for HIV?	YES . . . . 1<br>NO . . . . 2<br>DON'T KNOW/NOT SURE/DEPENDS 8	
723	Do people talk badly about people living with HIV, or who are thought to be living with HIV?	YES . . . . 1<br>NO . . . . 2<br>DON'T KNOW/NOT SURE/DEPENDS 8	
724	Do people living with HIV, or thought to be living with HIV, lose the respect of other people?	YES . . . . 1<br>NO . . . . 2<br>DON'T KNOW/NOT SURE/DEPENDS 8	
725	Do you agree or disagree with the following statement: I would be ashamed if someone in my family had HIV.	AGREE . . . . 1<br>DISAGREE . . . . 2<br>DON'T KNOW/NOT SURE/DEPENDS 8	
726	Do you fear that you could get HIV if you come into contact with the saliva of a person living with HIV?	YES . . . . 1<br>NO . . . . 2<br>SAYS HE HAS HIV . . . . 3<br>DON'T KNOW/NOT SURE/DEPENDS 8	
727	CHECK 701:<br>HEARD ABOUT HIV OR AIDS ☐ ↓ | NOT HEARD ABOUT HIV OR AIDS ☐ ↓<br>a) Apart from HIV, have you heard about other infections that can be transmitted through sexual contact? | b) Have you heard about infections that can be transmitted through sexual contact?	YES . . . . 1<br>NO 2	
728	CHECK 414:<br>HAS HAD SEXUAL INTERCOURSE ☐ ↓ NEVER HAD SEXUAL INTERCOURSE ☐ →		736
729	CHECK 727: HEARD ABOUT OTHER SEXUALLY TRANSMITTED INFECTIONS?<br>YES ☐ ↓ NO ☐ →		731
730	Now I would like to ask you some questions about your health in the last 12 months. During the last 12 months, have you had a disease which you got through sexual contact?	YES . . . . 1<br>NO . . . . 2<br>DON'T KNOW 8	
731	Sometimes men experience an abnormal discharge from their penis. During the last 12 months, have you had an abnormal discharge from your penis?	YES . . . . 1<br>NO . . . . 2<br>DON'T KNOW 8	
732	Sometimes men have a sore or ulcer near their penis. During the last 12 months, have you had a sore or ulcer on or near your penis?	YES . . . . 1<br>NO . . . . 2<br>DON'T KNOW 8	

NO.	QUESTIONS AND FILTERS	CODING CATEGORIES	SKIP
733	CHECK 730, 731 AND 732:<br>HAS HAD AN INFECTION (ANY 'YES') ☐	HAS NOT HAD AN INFECTION OR DOES NOT KNOW ☐	→ 736
734	The last time you had (PROBLEM FROM 730/731/732), did you seek any kind of advice or treatment?	YES . . . . 1<br>NO 2	 → 736
735	Where did you go?<br><br>Any other place?<br><br>PROBE TO IDENTIFY THE TYPE OF SOURCE.<br><br>IF UNABLE TO DETERMINE IF PUBLIC OR PRIVATE SECTOR, WRITE THE NAME OF THE PLACE.<br><br>__________<br>(NAME OF PLACE)	**PUBLIC SECTOR**<br>GOVERNMENT HOSPITAL . . . . A<br>GOVERNMENT HEALTH CENTER . . . . B<br>STAND-ALONE HTS CENTER . . . . C<br>FAMILY PLANNING CLINIC . . . . D<br>MOBILE HTS SERVICES . . . . E<br>OTHER PUBLIC SECTOR<br>__________ F<br>(SPECIFY)<br>**PRIVATE MEDICAL SECTOR**<br>PRIVATE HOSPITAL/CLINIC/<br>PRIVATE DOCTOR . . . . G<br>STAND-ALONE HTS CENTER . . . . H<br>PHARMACY . . . . I<br>CHEMIST/PMS STORE . . . . J<br>MOBILE HTS SERVICES . . . . K<br>OTHER PRIVATE MEDICAL SECTOR<br>__________ L<br>(SPECIFY)<br>**OTHER SOURCE**<br>SHOP . . . . M<br><br>OTHER __________ X<br>(SPECIFY)	
736	If a wife knows her husband has a disease that she can get during sexual intercourse, is she justified in asking that they use a condom when they have sex?	YES . . . . 1<br>NO . . . . 2<br>DON'T KNOW 8	
737	Is a wife justified in refusing to have sex with her husband when she knows he has sex with women other than his wives?	YES . . . . 1<br>NO . . . . 2<br>DON'T KNOW 8	

SECTION 8. OTHER HEALTH ISSUES

NO.	QUESTIONS AND FILTERS	CODING CATEGORIES	SKIP
801	Some men are circumcised, that is, the foreskin is completely removed from the penis. Are you circumcised?	YES . . . . 1<br>NO . . . . 2<br>DON'T KNOW 8	 NO, DON'T KNOW → 805
802	How old were you when you got circumcised?	AGE IN COMPLETED YEARS . . . . [ ][ ]<br><br>DURING CHILDHOOD (<5 YEARS) . . . . 95<br>DON'T KNOW 98	
803	Who did the circumcision?	TRADITIONAL PRACTITIONER/FAMILY/FRIEND . . 1<br>HEALTH WORKER/PROFESSIONAL . . . . 2<br>OTHER . . . . 3<br>DON'T KNOW 8	
804	Where was it done?	HEALTH FACILITY . . . . 1<br>HOME OF A HEALTH WORKER/PROFESSIONAL . . 2<br>CIRCUMCISION DONE AT HOME . . . . 3<br>RITUAL SITE . . . . 4<br>OTHER HOME/PLACE . . . . 5<br>DON'T KNOW 8	
805	Now I would like to ask you some other questions relating to health matters. Have you had an injection for any reason in the last 12 months?<br><br>IF YES: How many injections have you had?<br><br>IF NUMBER OF INJECTIONS IS 90 OR MORE, OR DAILY FOR 3 MONTHS OR MORE, RECORD '90'. IF NON-NUMERIC ANSWER, PROBE TO GET AN ESTIMATE.	NUMBER OF INJECTIONS . . . . [ ][ ]<br><br>NONE 00	 <br>NONE → 808
806	Among these injections, how many were administered by a doctor, a nurse, a pharmacist, a dentist, or any other health worker?<br><br>IF NUMBER OF INJECTIONS IS 90 OR MORE, OR DAILY FOR 3 MONTHS OR MORE, RECORD '90'. IF NON-NUMERIC ANSWER, PROBE TO GET AN ESTIMATE.	NUMBER OF INJECTIONS . . . . [ ][ ]<br><br>NONE 00	 <br>NONE → 808
807	The last time you got an injection from a health worker, did he/she take the syringe and needle from a new, unopened package?	YES . . . . 1<br>NO . . . . 2<br>DON'T KNOW 8	
808	Do you currently smoke tobacco every day, some days, or not at all?	EVERY DAY . . . . 1<br>SOME DAYS . . . . 2<br>NOT AT ALL 3	EVERY DAY → 811<br><br>NOT AT ALL → 810
809	In the past, have you smoked tobacco every day?	YES . . . . 1<br>NO 2	YES, NO → 812
810	In the past, have you ever smoked tobacco every day, some days, or not at all?	EVERY DAY . . . . 1<br>SOME DAYS . . . . 2<br>NOT AT ALL 3	EVERY DAY, SOME DAYS, NOT AT ALL → 813

SECTION 8. OTHER HEALTH ISSUES

NO.	QUESTIONS AND FILTERS	CODING CATEGORIES	SKIP
811	On average, how many of the following products do you currently smoke each day? Also, let me know if you use the product, but not every day.<br><br>IF RESPONDENT REPORTS USING THE PRODUCT BUT NOT EVERY DAY, RECORD '888'. IF THE PRODUCT IS NOT USED AT ALL, RECORD '000'.	NUMBER DAILY	
	a) Manufactured cigarettes?	a) MANUFACTURED CIGARETTES . . . . [ ][ ][ ]	→ 813
	b) Hand-rolled cigarettes?	b) HAND-ROLLED CIGARETTES . . . . [ ][ ][ ]	→ 813
	c) Kreteks?	c) KRETEKS . . . . [ ][ ][ ]	→ 813
	d) Pipes full of tobacco?	d) PIPES FULL OF TOBACCO . . . . [ ][ ][ ]	→ 813
	e) Cigars, cheroots, or cigarillos?	e) CIGARS, CHEROOTS, OR CIGARILLOS . . . . . [ ][ ][ ]	→ 813
	f) Number of water pipe sessions?	f) NUMBER OF WATER PIPE SESSIONS . . . . . [ ][ ][ ]	→ 813
	g) Any others? __________ (SPECIFY)	g) OTHERS . . . . [ ][ ][ ]	→ 813
812	On average, how many of the following products do you currently smoke each week? Also, let me know if you use the product, but not every week.<br><br>IF RESPONDENT REPORTS USING THE PRODUCT BUT NOT EVERY WEEK, RECORD '888'. IF THE PRODUCT IS NOT USED AT ALL, RECORD '000'.	NUMBER WEEKLY	
	a) Manufactured cigarettes?	a) MANUFACTURED CIGARETTES . . . . [ ][ ][ ]	
	b) Hand-rolled cigarettes?	b) HAND-ROLLED CIGARETTES . . . . [ ][ ][ ]	
	c) Kreteks?	c) KRETEKS . . . . [ ][ ][ ]	
	d) Pipes full of tobacco?	d) PIPES FULL OF TOBACCO . . . . [ ][ ][ ]	
	e) Cigars, cheroots, or cigarillos?	e) CIGARS, CHEROOTS, OR CIGARILLOS . . . . . [ ][ ][ ]	
	f) Number of water pipe sessions?	f) NUMBER OF WATER PIPE SESSIONS . . . . . [ ][ ][ ]	
	g) Any others? __________ (SPECIFY)	g) OTHERS . . . . [ ][ ][ ]	
813	Do you currently use smokeless tobacco every day, some days, or not at all?	EVERY DAY . . . . 1<br>SOME DAYS . . . . 2<br>NOT AT ALL 3	 → 815<br>→ 815A

NO.	QUESTIONS AND FILTERS	CODING CATEGORIES	SKIP
814	On average, how many times a day do you use the following products? Also, let me know if you use the product, but not every day.<br><br>IF RESPONDENT REPORTS USING THE PRODUCT BUT NOT EVERY DAY, RECORD '888'. IF THE PRODUCT IS NOT USED AT ALL, RECORD '000'.<br><br>a) Snuff, by mouth?<br>b) Snuff, by nose?<br>c) Chewing tobacco?<br>d) Betel quid with tobacco?<br>e) Any others?<br>________________<br>(SPECIFY)	TIMES DAILY<br>a) SNUFF, BY MOUTH . . . . [ ][ ][ ]<br>b) SNUFF, BY NOSE . . . . [ ][ ][ ]<br>c) CHEWING TOBACCO . . . . . [ ][ ][ ]<br>d) BETEL QUID WITH TOBACCO . . . . . [ ][ ][ ]<br>e) ANY OTHERS . . . . [ ][ ][ ]	→ 815A
815	On average, how many times a week do you use the following products? Also, let me know if you use the product, but not every week.<br><br>IF RESPONDENT REPORTS USING THE PRODUCT BUT NOT EVERY WEEK, RECORD '888'. IF THE PRODUCT IS NOT USED AT ALL, RECORD '000'.<br><br>a) Snuff, by mouth?<br>b) Snuff, by nose?<br>c) Chewing tobacco?<br>d) Betel quid with tobacco?<br>e) Any others?<br>________________<br>(SPECIFY)	TIMES WEEKLY<br>a) SNUFF, BY MOUTH . . . . [ ][ ][ ]<br>b) SNUFF, BY NOSE . . . . [ ][ ][ ]<br>c) CHEWING TOBACCO . . . . . [ ][ ][ ]<br>d) BETEL QUID WITH TOBACCO . . . . . [ ][ ][ ]<br>e) ANY OTHERS . . . . [ ][ ][ ]	

815A	I am going to ask you about your opinion on behavior/practice on reducing the risk of malaria. Please tell me whether you agree or disagree with the following statements:		AGREE	DISAGREE	DON'T KNOW
	b) The medicine given to pregnant women to prevent malaria works well to keep the mother healthy	b)	1	2	8
	c) The medicine given to pregnant women to prevent malaria works well to keep the baby healthy when it is born	c)	1	2	8
	d) The malaria tests are the only way to know if someone really has malaria or not	d)	1	2	8
	f) Even if the malaria test shows that the fever is not caused by malaria, I will still seek out treatment for malaria because I don't trust the test result	f)	1	2	8
	i) When the entire course of malaria medicine is taken, the disease will be fully cured	i)	1	2	8

SECTION 8. OTHER HEALTH ISSUES

NO.	QUESTIONS AND FILTERS	CODING CATEGORIES	SKIP
815B	I am going to ask you about your opinion on consequences of malaria. Please tell me whether you agree or disagree with the following statements:<br>a) Every case of malaria can potentially lead to death<br>c) You don't worry about malaria because it can be easily treated<br>d) You know people who have become dangerously sick with malaria.<br>f) Only weak children can die from malaria	AGREE / DISAGREE / DON'T KNOW<br>a) 1 2 8<br>c) 1 2 8<br>d) 1 2 8<br>f) 1 2 8	
816	Are you covered by any health insurance?	YES . . . . 1<br>NO 2	 →818
817	What type of health insurance are you covered by?<br><br>RECORD ALL MENTIONED.	MUTUAL HEALTH ORGANIZATION/ COMMUNITY-BASED HEALTH INSURANCE . . . . A<br>HEALTH INSURANCE THROUGH EMPLOYER . . . . B<br>SOCIAL SECURITY . . . . C<br>OTHER PRIVATELY PURCHASED COMMERCIAL HEALTH INSURANCE . . . . . D<br><br>OTHER ______________ X<br>(SPECIFY)	
818	RECORD THE TIME.	HOURS . . . . [ ][ ]<br>MINUTES . . . . [ ][ ]	

INTERVIEWER'S OBSERVATIONS

TO BE FILLED IN AFTER COMPLETING INTERVIEW

COMMENTS ABOUT INTERVIEW:

COMMENTS ON SPECIFIC QUESTIONS:

ANY OTHER COMMENTS:

SUPERVISOR'S OBSERVATIONS

EDITOR'S OBSERVATIONS

FORMATTING DATE: 09 Jun 2015
ENGLISH LANGUAGE: 26 May 2018

NIGERIA DEMOGRAPHIC AND HEALTH SURVEYS 2018
BIOMARKER QUESTIONNAIRE

NIGERIA

IDENTIFICATION

STATE ____________

LOCAL GOVT. AREA ____________

LOCALITY ____________

ENUMERATION AREA ____________

NAME OF HOUSEHOLD HEAD ____________

CLUSTER NUMBER . . . .

HOUSEHOLD NUMBER . . . .

HOUSEHOLD SELECTED FOR MAN'S SURVEY? (1=YES, 2=NO) . . . .

HOUSEHOLD SELECTED FOR MICROSCOPY OR DBS?
SELECTED FOR MICROSCOPY = 1
SELECTED FOR DBS = 2 . . . .

FIELDWORKER VISITS

	1	2	3	FINAL VISIT
DATE<br>FIELDWORKER'S NAME				DAY<br>MONTH<br>YEAR
NEXT VISIT: DATE<br>TIME				TOTAL NUMBER OF VISITS
NOTES:				TOTAL ELIGIBLE WOMEN<br>TOTAL ELIGIBLE CHILDREN

LANGUAGE OF QUESTIONNAIRE** 0 1 | LANGUAGE OF INTERVIEW** | NATIVE LANGUAGE OF RESPONDENT** | TRANSLATOR (YES = 1, NO = 2)

LANGUAGE OF QUESTIONNAIRE** ENGLISH

**LANGUAGE CODES:
01 ENGLISH
02 HAUSA
03 YORUBA
04 IGBO

SUPERVISOR	FIELD EDITOR
NAME / NUMBER	NAME / NUMBER

101	CHECK COLUMN 11 IN HOUSEHOLD QUESTIONNAIRE. RECORD THE LINE NUMBER AND NAME FOR ALL ELIGIBLE CHILDREN 0-5 YEARS IN QUESTION 102; IF MORE THAN SIX CHILDREN, USE ADDITIONAL QUESTIONNAIRE(S).			
		CHILD 1	CHILD 2	CHILD 3
102	CHECK HOUSEHOLD QUESTIONNAIRE:<br>LINE NUMBER FROM COLUMN 11.	LINE NUMBER . . . . . . [ ][ ]<br>NAME ________	LINE NUMBER . . . . . . [ ][ ]<br>NAME ________	LINE NUMBER . . . . . . [ ][ ]<br>NAME ________
103	IF MOTHER INTERVIEWED:<br>COPY CHILD'S DATE OF BIRTH (DAY, MONTH, AND YEAR) FROM BIRTH HISTORY. IF MOTHER NOT INTERVIEWED ASK:<br>What is (NAME)'s date of birth?	DAY . . . . [ ][ ]<br>MONTH . . . . [ ][ ]<br>YEAR . . . [ ][ ][ ][ ]	DAY . . . . [ ][ ]<br>MONTH . . . . [ ][ ]<br>YEAR . . . [ ][ ][ ][ ]	DAY . . . . [ ][ ]<br>MONTH . . . . [ ][ ]<br>YEAR . . . [ ][ ][ ][ ]
104	CHECK 103: CHILD BORN IN 2013-2018?	YES . . . . 1<br>NO . . . . 2<br>(SKIP TO 130)	YES . . . . 1<br>NO . . . . 2<br>(SKIP TO 130)	YES . . . . 1<br>NO . . . . 2<br>(SKIP TO 130)
105	WEIGHT IN KILOGRAMS.	KG. . . . [ ][ ] . [ ][ ]<br>NOT PRESENT . . . . .9994<br>REFUSED . . . . 9995<br>OTHER 9996	KG. . . . [ ][ ] . [ ][ ]<br>NOT PRESENT . . . . .9994<br>REFUSED . . . . 9995<br>OTHER 9996	KG. . . . [ ][ ] . [ ][ ]<br>NOT PRESENT . . . . . . .9994<br>REFUSED . . . . 9995<br>OTHER 9996
106	HEIGHT IN CENTIMETERS.	CM. . . . [ ][ ][ ] . [ ]<br>NOT PRESENT . . . . .9994<br>REFUSED . . . . 9995<br>OTHER . . . . 9996<br>(SKIP TO 108)	CM. . . . [ ][ ][ ] . [ ]<br>NOT PRESENT . . . . .9994<br>REFUSED . . . . 9995<br>OTHER . . . . 9996<br>(SKIP TO 108)	CM. . . . [ ][ ][ ] . [ ]<br>NOT PRESENT . . . . . . .9994<br>REFUSED . . . . 9995<br>OTHER . . . . 9996<br>(SKIP TO 108)
107	MEASURED LYING DOWN OR STANDING UP?	LYING DOWN . . . . . . 1<br>STANDING UP 2	LYING DOWN . . . . . . 1<br>STANDING UP 2	LYING DOWN . . . . 1<br>STANDING UP 2
108	MEASURER: ENTER YOUR FIELDWORKER NUMBER.	[ ][ ][ ][ ]<br>FIELDWORKER NUMBER	[ ][ ][ ][ ]<br>FIELDWORKER NUMBER	[ ][ ][ ][ ]<br>FIELDWORKER NUMBER
109	CHECK 103: CHILD AGE 0-5 MONTHS, I.E., WAS CHILD BORN IN MONTH OF INTERVIEW OR 5 PREVIOUS MONTHS?	0-5 MONTHS . . . . 1<br>(SKIP TO 130)<br>OLDER 2	0-5 MONTHS . . . . 1<br>(SKIP TO 130)<br>OLDER 2	0-5 MONTHS . . . . 1<br>(SKIP TO 130)<br>OLDER 2
110	NAME AND RELATIONSHIP TO THE CHILD OF ADULT RESPONSIBLE FOR THE CHILD. GET LINE NUMBER FROM COLUMN 1 OF HOUSEHOLD SCHEDULE.	NAME ________<br>RELATIONSHIP TO THE CHILD ________<br>LINE NUMBER . . . . . . [ ][ ]<br>(RECORD '00' IF NOT LISTED)	NAME ________<br>RELATIONSHIP TO THE CHILD ________<br>LINE NUMBER . . . . . . [ ][ ]<br>(RECORD '00' IF NOT LISTED)	NAME ________<br>RELATIONSHIP TO THE CHILD ________<br>LINE NUMBER . . . . . . [ ][ ]<br>(RECORD '00' IF NOT LISTED)
111	ASK CONSENT FOR ANEMIA TEST FROM PARENT/OTHER ADULT.	As part of this survey, we are asking people all over the country to take an anemia test. Anemia is a serious health problem that usually results from poor nutrition, infection, or chronic disease. This survey will assist the government to develop programs to prevent and treat anemia. We ask that all children born in 2013 or later take part in anemia testing in this survey and give a few drops of blood from a finger or heel. The equipment used to take the blood is clean and completely safe. It has never been used before and will be thrown away after each test.<br><br>The blood will be tested for anemia immediately, and the result will be told to you right away. The result will be kept strictly confidential and will not be shared with anyone other than members of our survey team.<br><br>Do you have any questions?<br>You can say yes or no. It is up to you to decide.<br>Will you allow (NAME OF CHILD) to participate in the anemia test?		

WEIGHT, HEIGHT AND HEMOGLOBIN, GENOTYPE, AND MALARIA MEASUREMENT FOR CHILDREN AGE 0-5

101	CHECK COLUMN 11 IN HOUSEHOLD QUESTIONNAIRE. RECORD THE LINE NUMBER AND NAME FOR ALL ELIGIBLE CHILDREN 0-5 YEARS IN QUESTION 102; IF MORE THAN SIX CHILDREN, USE ADDITIONAL QUESTIONNAIRE(S).			
		CHILD 1	CHILD 2	CHILD 3
102	CHECK HOUSEHOLD QUESTIONNAIRE: LINE NUMBER FROM COLUMN 11.	LINE NUMBER . . . . . . [ ][ ]<br>NAME ________	LINE NUMBER . . . . . . [ ][ ]<br>NAME ________	LINE NUMBER . . . . . . [ ][ ]<br>NAME ________
111A	CIRCLE THE CODE AND SIGN YOUR NAME.	GRANTED . . . . 1<br>________ (SIGN)<br>REFUSED . . . . 2<br>NOT PRESENT/OTHER . 3<br>(SKIP TO 130)	GRANTED . . . . 1<br>________ (SIGN)<br>REFUSED . . . . 2<br>NOT PRESENT/OTHER . 3<br>(SKIP TO 130)	GRANTED . . . . 1<br>________ (SIGN)<br>REFUSED . . . . 2<br>NOT PRESENT/OTHER . 3<br>(SKIP TO 130)
112	Has (NAME) had blood transfusion in the past 3 months?	YES . . . . 1<br>(SKIP TO 112C)<br>NO 2	YES . . . . 1<br>(SKIP TO 112C)<br>NO 2	YES . . . . 1<br>(SKIP TO 112C)<br>NO 2
112A	ASK CONSENT FOR GENOTYPE TEST FROM PARENT/OTHER ADULT.	As part of this survey, we are asking children all over the country to take a test to see if they have sickle cell anemia (SS) or its trait (AS). Sickle cell disease (SCD) is an inherited blood disorder, which affects children early in life often with repeated episodes of catastrophic illness and bone pains with varying periods of relative good health in between. This survey will assist the government to develop programs for the prevention and management of sickle cell disease.<br><br>We ask that all children born in 2013 or later take part in genotype testing in this survey and give a few drops of blood from a finger or heel. One blood drop will be tested for genotype immediately, and the result will be told to you right away. The equipment used to take the blood is clean and completely safe. It has never been used before and will be thrown away after each test. The result will be kept strictly confidential and will not be shared with anyone other than members of our survey team.<br><br>Do you have any questions? You can say yes or no. It is up to you to decide. Will you allow (NAME OF CHILD) to participate in the genotype test?		
112B	CIRCLE THE CODE AND SIGN YOUR NAME.	GRANTED . . . . 1<br>________ (SIGN)<br>REFUSED . . . . 2<br>NOT PRESENT/OTHER . 3<br>**(SKIP TO 130)**	GRANTED . . . . 1<br>________ (SIGN)<br>REFUSED . . . . 2<br>NOT PRESENT/OTHER . 3<br>**(SKIP TO 130)**	GRANTED . . . . 1<br>________ (SIGN)<br>REFUSED . . . . 2<br>NOT PRESENT/OTHER . 3<br>**(SKIP TO 130)**
112C	ASK CONSENT FOR MALARIA TEST FROM PARENT/OTHER ADULT.	As part of this survey, we are asking children all over the country to take a test to see if they have malaria. Malaria is a serious illness caused by a parasite transmitted by a mosquito bite. This survey will assist the government to develop programs to prevent malaria.<br><br>We ask that all children born in 2013 or later take part in malaria testing in this survey and give a few drops of blood from a finger or heel. The blood will be tested for malaria immediately, and the result will be told to you right away. All results will be kept strictly confidential and will not be shared with anyone other than members of our survey team.<br><br>Do you have any questions?<br>You can say yes or no. It is up to you to decide.<br>Will you allow (NAME OF CHILD) to participate in the malaria test?		
112D	CIRCLE THE CODE, SIGN YOUR NAME, AND ENTER YOUR FIELDWORKER NUMBER.	GRANTED . . . . 1<br>REFUSED . . . . 2<br>________<br>(SIGN AND ENTER YOUR FIELDWORKER NUMBER)<br>[ ][ ][ ][ ]	GRANTED . . . . 1<br>REFUSED . . . . 2<br>________<br>(SIGN AND ENTER YOUR FIELDWORKER NUMBER)<br>[ ][ ][ ][ ]	GRANTED . . . . 1<br>REFUSED . . . . 2<br>________<br>(SIGN AND ENTER YOUR FIELDWORKER NUMBER)<br>[ ][ ][ ][ ]

WEIGHT, HEIGHT AND HEMOGLOBIN, GENOTYPE, AND MALARIA MEASUREMENT FOR CHILDREN AGE 0-5

101	CHECK COLUMN 11 IN HOUSEHOLD QUESTIONNAIRE. RECORD THE LINE NUMBER AND NAME FOR ALL ELIGIBLE CHILDREN 0-5 YEARS IN QUESTION 102; IF MORE THAN SIX CHILDREN, USE ADDITIONAL QUESTIONNAIRE(S).			
		CHILD 1	CHILD 2	CHILD 3
102	CHECK HOUSEHOLD QUESTIONNAIRE:<br>LINE NUMBER FROM COLUMN 11.	LINE NUMBER ...... [ ][ ]<br>NAME ________	LINE NUMBER ...... [ ][ ]<br>NAME ________	LINE NUMBER ...... [ ][ ]<br>NAME ________

112E	PREPARE EQUIPMENT AND SUPPLIES ONLY FOR THE TEST(S) FOR WHICH CONSENT HAS BEEN OBTAINED AND PROCEED WITH THE TEST(S).			
112F	PLACE BAR CODE LABEL FOR MALARIA LAB TEST OR GENOTYPE CONFIRMATORY LAB TEST.	PUT THE 1ST BAR CODE LABEL HERE.<br>NOT PRESENT ... 99994<br>REFUSED .... 99995<br>OTHER 99996	PUT THE 1ST BAR CODE LABEL HERE.<br>NOT PRESENT ... 99994<br>REFUSED .... 99995<br>OTHER 99996	PUT THE 1ST BAR CODE LABEL HERE.<br>NOT PRESENT ..... 99994<br>REFUSED .... 99995<br>OTHER 99996
	MENTION WHERE BAR CODE PLACED BASED ON THE TEST.	SLIDE .... A<br>DBS .... B<br>TRANSMITTAL FORM ... C	SLIDE .... A<br>DBS .... B<br>TRANSMITTAL FORM ... C	SLIDE .... A<br>DBS .... B<br>TRANSMITTAL FORM ... C
	**RESULTS OF HEMOGLOBIN TEST**			
113	RECORD HEMOGLOBIN LEVEL HERE AND IN THE PAMPHLET.	G/DL ..... [ ][ ].[ ]<br>NOT PRESENT ..... 994<br>REFUSED .... 995<br>OTHER 996	G/DL ..... [ ][ ].[ ]<br>NOT PRESENT ..... 994<br>REFUSED .... 995<br>OTHER 996	G/DL ..... [ ][ ].[ ]<br>NOT PRESENT .... 994<br>REFUSED .... 995<br>OTHER 996
	**RESULTS OF GENOTYPE TEST**			
113A	CIRCLE THE CODE FOR THE GENOTYPE RDT.	TESTED .... 1<br>NOT PRESENT ...... 2<br>REFUSED .... 3<br>OTHER .... 6<br>(SKIP TO 114)	TESTED .... 1<br>NOT PRESENT ...... 2<br>REFUSED .... 3<br>OTHER .... 6<br>(SKIP TO 114)	TESTED .... 1<br>NOT PRESENT .... 2<br>REFUSED .... 3<br>OTHER .... 6<br>(SKIP TO 114)
113B	RECORD THE RESULT OF THE GENOTYPE RDT HERE AND IN THE PAMPHLET.	AA .... 1<br>AS .... 2<br>AC .... 3<br>SC .... 4<br>(SKIP TO 114)<br>SS 5	AA .... 1<br>AS .... 2<br>AC .... 3<br>SC .... 4<br>(SKIP TO 114)<br>SS 5	AA .... 1<br>AS .... 2<br>AC .... 3<br>SC .... 4<br>(SKIP TO 114)<br>SS 5
113C	<u>**SICKLE CELL ANEMIA (SS) REFERRAL**</u><br><br>RECORD THE RESULT OF THE GENOTYPE TEST ON THE REFERRAL FORM.	The genotype test shows that (NAME OF CHILD) has sickle cell anemia. Your child is very ill and must be taken to a health facility immediately.		
	**RESULTS OF MALARIA RDT TEST**			
114	CIRCLE THE CODE FOR THE MALARIA RDT.	TESTED .... 1<br>NOT PRESENT ...... 2<br>REFUSED .... 3<br>OTHER .... 6<br>(SKIP TO 128)	TESTED .... 1<br>NOT PRESENT ...... 2<br>REFUSED .... 3<br>OTHER .... 6<br>(SKIP TO 128)	TESTED .... 1<br>NOT PRESENT .... 2<br>REFUSED .... 3<br>OTHER .... 6<br>(SKIP TO 128)
115	RECORD THE RESULT OF THE MALARIA RDT HERE AND IN THE PAMPHLET.	POSITIVE .... 1<br>NEGATIVE .... 2<br>OTHER .... 6<br>(SKIP TO 128)	POSITIVE .... 1<br>NEGATIVE .... 2<br>OTHER .... 6<br>(SKIP TO 128)	POSITIVE .... 1<br>NEGATIVE .... 2<br>OTHER .... 6<br>(SKIP TO 128)

101	CHECK COLUMN 11 IN HOUSEHOLD QUESTIONNAIRE. RECORD THE LINE NUMBER AND NAME FOR ALL ELIGIBLE CHILDREN 0-5 YEARS IN QUESTION 102; IF MORE THAN SIX CHILDREN, USE ADDITIONAL QUESTIONNAIRE(S).			
		CHILD 1	CHILD 2	CHILD 3
102	CHECK HOUSEHOLD QUESTIONNAIRE:<br>LINE NUMBER FROM COLUMN 11.	LINE NUMBER . . . . . . [ ][ ]<br>NAME ________	LINE NUMBER . . . . . . [ ][ ]<br>NAME ________	LINE NUMBER . . . . . . [ ][ ]<br>NAME ________

118	Does (NAME) suffer from any of the following illnesses or symptoms:<br>a) Extreme weakness?<br>b) Heart problems?<br>c) Loss of consciousness?<br>d) Rapid or difficult breathing?<br>e) Seizures?<br>f) Abnormal bleeding?<br>g) Jaundice or yellow skin?<br>h) Dark urine?	YES NO<br>a) EXTREME WEAKNESS 1 2<br>b) HEART PROBLEMS 1 2<br>c) LOSS OF CONSCIOUS. 1 2<br>d) RAPID BREATHING 1 2<br>e) SEIZURES 1 2<br>f) BLEEDING 1 2<br>g) JAUNDICE 1 2<br>h) DARK URINE 1 2	YES NO<br>a) EXTREME WEAKNESS 1 2<br>b) HEART PROBLEMS 1 2<br>c) LOSS OF CONSCIOUS. 1 2<br>d) RAPID BREATHING 1 2<br>e) SEIZURES 1 2<br>f) BLEEDING 1 2<br>g) JAUNDICE 1 2<br>h) DARK URINE 1 2	YES NO<br>a) EXTREME WEAKNESS 1 2<br>b) HEART PROBLEMS 1 2<br>c) LOSS OF CONSCIOUS. 1 2<br>d) RAPID BREATHING 1 2<br>e) SEIZURES 1 2<br>f) BLEEDING 1 2<br>g) JAUNDICE 1 2<br>h) DARK URINE 1 2
119	CHECK 118:<br>ANY 'YES' CIRCLED?	NO [ ] YES [ ]<br>(SKIP TO 122)	NO [ ] YES [ ]<br>(SKIP TO 122)	NO [ ] YES [ ]<br>(SKIP TO 122)
120	CHECK 113:<br>HEMOGLOBIN RESULT	BELOW 8.0 G/DL, SEVERE ANEMIA . . . 1<br>(SKIP TO 122)<br>8.0 G/DL OR ABOVE . . . 2<br>NOT PRESENT . . . . . . 3<br>REFUSED . . . . 4<br>OTHER 6	BELOW 8.0 G/DL, SEVERE ANEMIA . . . 1<br>(SKIP TO 122)<br>8.0 G/DL OR ABOVE . . . 2<br>NOT PRESENT . . . . . . 3<br>REFUSED . . . . 4<br>OTHER 6	BELOW 8.0 G/DL, SEVERE ANEMIA . . . 1<br>(SKIP TO 122)<br>8.0 G/DL OR ABOVE . . . 2<br>NOT PRESENT . . . . 3<br>REFUSED . . . . 4<br>OTHER 6
121	In the past two weeks has (NAME) taken or is taking ACT given by a doctor or health center to treat the malaria?<br><br>VERIFY BY ASKING TO SEE TREATMENT	YES . . . . 1<br>(SKIP TO 123)<br>NO . . . . 2<br>(SKIP TO 124)	YES . . . . 1<br>(SKIP TO 123)<br>NO . . . . 2<br>(SKIP TO 124)	YES . . . . 1<br>(SKIP TO 123)<br>NO . . . . 2<br>(SKIP TO 124)
122	**SEVERE MALARIA REFERRAL**<br><br>RECORD THE RESULT OF THE MALARIA RDT ON THE REFERRAL FORM.	The malaria test shows that (NAME OF CHILD) has malaria. Your child also has symptoms of severe malaria. The malaria treatment I have will not help your child, and I cannot give you the medication. Your child is very ill and must be taken to a health facility right away.<br><br>**(SKIP TO 128)**		
123	ALREADY TAKING ACT REFERRAL STATEMENT	You have told me that (NAME OF CHILD) had already received ACT for malaria. Therefore, I cannot give you additional ACT. However, the test shows that he/she has malaria. If your child has a fever for two days after the last dose of ACT, you should take the child to the nearest health facility for further examination.<br><br>**(SKIP TO 130)**		
124	READ INFORMATION FOR MALARIA TREATMENT AND CONSENT STATEMENT TO PARENT/OTHER	The malaria test shows that your child has malaria. We can give you free medicine. The medicine is called ACT. ACT is very effective and in a few days it should get rid of the fever and other symptoms. You do not have to give the child the medicine. This is up to you. Please tell me whether you accept the medicine or not.		

101	CHECK COLUMN 11 IN HOUSEHOLD QUESTIONNAIRE. RECORD THE LINE NUMBER AND NAME FOR ALL ELIGIBLE CHILDREN 0-5 YEARS IN QUESTION 102; IF MORE THAN SIX CHILDREN, USE ADDITIONAL QUESTIONNAIRE(S).			
		CHILD 1	CHILD 2	CHILD 3
102	CHECK HOUSEHOLD QUESTIONNAIRE:<br>LINE NUMBER FROM COLUMN 11.	LINE NUMBER . . . . . . [ ][ ]<br>NAME ______	LINE NUMBER . . . . . . [ ][ ]<br>NAME ______	LINE NUMBER . . . . . . [ ][ ]<br>NAME ______
125	CIRCLE THE APPROPRIATE CODE AND SIGN YOUR NAME.	ACCEPTED MEDICINE . 1<br>______ (SIGN)<br>REFUSED . . . . 2<br>OTHER 6	ACCEPTED MEDICINE . 1<br>______ (SIGN)<br>REFUSED . . . . 2<br>OTHER 6	ACCEPTED MEDICINE . 1<br>______ (SIGN)<br>REFUSED . . . . 2<br>OTHER 6
126	CHECK 125:<br>MEDICATION ACCEPTED	ACCEPTED MEDICINE . 1<br>REFUSED . . . . 2<br>OTHER . . . . 6<br>(SKIP TO 130)	ACCEPTED MEDICINE . 1<br>REFUSED . . . . 2<br>OTHER . . . . 6<br>(SKIP TO 130)	ACCEPTED MEDICINE . 1<br>REFUSED . . . . 2<br>OTHER . . . . 6<br>(SKIP TO 130)
127	TREATMENT FOR CHILDREN WITH POSITIVE MALARIA TESTS	**TREATMENT WITH ACT** (see table below)		
128	CHECK 113:<br>HEMOGLOBIN RESULT	BELOW 8.0 G/DL, SEVERE ANEMIA 1<br>8.0 G/DL OR ABOVE 2<br>NOT PRESENT 3<br>REFUSED 4<br>OTHER 6<br>**(SKIP TO 130)**	BELOW 8.0 G/DL, SEVERE ANEMIA 1<br>8.0 G/DL OR ABOVE 2<br>NOT PRESENT 3<br>REFUSED 4<br>OTHER 6<br>**(SKIP TO 130)**	BELOW 8.0 G/DL, SEVERE ANEMIA 1<br>8.0 G/DL OR ABOVE 2<br>NOT PRESENT 3<br>REFUSED 4<br>OTHER 6<br>**(SKIP TO 130)**
129	**SEVERE ANEMIA REFERRAL**<br>RECORD THE RESULT OF THE ANEMIA TEST ON THE REFERRAL FORM.	The anemia test shows that (NAME OF CHILD) has severe anemia. Your child is very ill and must be taken to a health facility immediately.		
130	GO BACK TO 103 IN NEXT COLUMN OF THIS QUESTIONNAIRE OR IN THE FIRST COLUMN OF THE NEXT PAGE;<br>IF NO MORE CHILDREN, GO TO 201.			

TREATMENT WITH ACT (127)

WEIGHT (in kg)	AGE	ARTEMETHER-LUMEFANTRINE
LESS THAN 5 KGS	NOTHING	NOTHING
5-14 KGS	6 MONTHS - 3 YEARS	1 TABLET TWICE A DAY FOR 3 DAYS
15-25 KGS	4 - 8 YEARS	2 TABLETS TWICE A DAY FOR 3 DAYS

IF CHILD WEIGHS LESS THAN 5 KGS, DO NOT LEAVE DRUGS. TELL PARENTS TO TAKE CHILD TO HEALTH FACILITY.

ALSO TELL THE PARENT/OTHER ADULT: If [NAME] has a high fever, fast or difficult breathing, is not able to drink or breastfeed, gets sicker or does not get better in two days, you should take him/her to a health professional for treatment right away.
(SKIP TO 130)

WEIGHT, HEIGHT AND HEMOGLOBIN, GENOTYPE, AND MALARIA MEASUREMENT FOR CHILDREN AGE 0-5

		CHILD 4	CHILD 5	CHILD 6
102	CHECK HOUSEHOLD QUESTIONNAIRE:<br>LINE NUMBER FROM COLUMN 11.	LINE NUMBER . . . . . . [ ][ ]<br>NAME ________	LINE NUMBER . . . . . . [ ][ ]<br>NAME ________	LINE NUMBER . . . . . . [ ][ ]<br>NAME ________
103	IF MOTHER INTERVIEWED:<br>COPY CHILD'S DATE OF BIRTH (DAY, MONTH, AND YEAR) FROM BIRTH HISTORY. IF MOTHER NOT INTERVIEWED ASK:<br>What is (NAME)'s date of birth?	DAY . . . . [ ][ ]<br>MONTH . . . . [ ][ ]<br>YEAR . . . [ ][ ][ ][ ]	DAY . . . . [ ][ ]<br>MONTH . . . . [ ][ ]<br>YEAR . . . [ ][ ][ ][ ]	DAY . . . . [ ][ ]<br>MONTH . . . . [ ][ ]<br>YEAR . . . [ ][ ][ ][ ]
104	CHECK 103: CHILD BORN IN 2013-2018?	YES . . . . 1<br>NO . . . . 2<br>(SKIP TO 130)	YES . . . . 1<br>NO . . . . 2<br>(SKIP TO 130)	YES . . . . 1<br>NO . . . . 2<br>(SKIP TO 130)
105	WEIGHT IN KILOGRAMS.	KG. . . . [ ][ ].[ ][ ]<br>NOT PRESENT . . . . .9994<br>REFUSED . . . . 9995<br>OTHER 9996	KG. . . . [ ][ ].[ ][ ]<br>NOT PRESENT . . . . .9994<br>REFUSED . . . . 9995<br>OTHER 9996	KG. . . . [ ][ ].[ ][ ]<br>NOT PRESENT . . . . .9994<br>REFUSED . . . . 9995<br>OTHER 9996
106	HEIGHT IN CENTIMETERS.	CM. . . . [ ][ ][ ].[ ]<br>NOT PRESENT . . . . .9994<br>REFUSED . . . . 9995<br>OTHER . . . . 9996<br>(SKIP TO 108)	CM. . . . [ ][ ][ ].[ ]<br>NOT PRESENT . . . . .9994<br>REFUSED . . . . 9995<br>OTHER . . . . 9996<br>(SKIP TO 108)	CM. . . . [ ][ ][ ].[ ]<br>NOT PRESENT . . . . .9994<br>REFUSED . . . . 9995<br>OTHER . . . . 9996<br>(SKIP TO 108)
107	MEASURED LYING DOWN OR STANDING UP?	LYING DOWN . . . . . . 1<br>STANDING UP 2	LYING DOWN . . . . . . 1<br>STANDING UP 2	LYING DOWN . . . . . . 1<br>STANDING UP 2
108	MEASURER: ENTER YOUR FIELDWORKER NUMBER.	[ ][ ][ ][ ]<br>FIELDWORKER NUMBER	[ ][ ][ ][ ]<br>FIELDWORKER NUMBER	[ ][ ][ ][ ]<br>FIELDWORKER NUMBER
109	CHECK 103: CHILD AGE 0-5 MONTHS, I.E., WAS CHILD BORN IN MONTH OF INTERVIEW OR 5 PREVIOUS MONTHS?	0-5 MONTHS . . . . 1<br>(SKIP TO 130)<br>OLDER 2	0-5 MONTHS . . . . 1<br>(SKIP TO 130)<br>OLDER 2	0-5 MONTHS . . . . 1<br>(SKIP TO 130)<br>OLDER 2
110	NAME AND RELATIONSHIP TO THE CHILD OF ADULT RESPONSIBLE FOR THE CHILD. GET LINE NUMBER FROM COLUMN 1 OF HOUSEHOLD SCHEDULE.	NAME ________<br>RELATIONSHIP TO THE CHILD ________<br>LINE NUMBER . . . . . . [ ][ ]<br>(RECORD '00' IF NOT LISTED)	NAME ________<br>RELATIONSHIP TO THE CHILD ________<br>LINE NUMBER . . . . . . [ ][ ]<br>(RECORD '00' IF NOT LISTED)	NAME ________<br>RELATIONSHIP TO THE CHILD ________<br>LINE NUMBER . . . . . . [ ][ ]<br>(RECORD '00' IF NOT LISTED)
111	ASK CONSENT FOR ANEMIA TEST FROM PARENT/OTHER ADULT.	As part of this survey, we are asking people all over the country to take an anemia test. Anemia is a serious health problem that usually results from poor nutrition, infection, or chronic disease. This survey will assist the government to develop programs to prevent and treat anemia. We ask that all children born in 2013 or later take part in anemia testing in this survey and give a few drops of blood from a finger or heel. The equipment used to take the blood is clean and completely safe. It has never been used before and will be thrown away after each test.<br><br>The blood will be tested for anemia immediately, and the result will be told to you right away. The result will be kept strictly confidential and will not be shared with anyone other than members of our survey team.<br><br>Do you have any questions?<br>You can say yes or no. It is up to you to decide.<br>Will you allow (NAME OF CHILD) to participate in the anemia test?		

		CHILD 4	CHILD 5	CHILD 6
102	CHECK HOUSEHOLD QUESTIONNAIRE:<br>LINE NUMBER FROM COLUMN 11.	LINE NUMBER . . . . . .<br>NAME ______________	LINE NUMBER . . . . . .<br>NAME ______________	LINE NUMBER . . . . . .<br>NAME ______________
111A	CIRCLE THE CODE AND SIGN YOUR NAME.	GRANTED . . . . 1<br>______________<br>(SIGN)<br>REFUSED . . . . 2<br>NOT PRESENT/OTHER . 3<br>(SKIP TO 130)	GRANTED . . . . 1<br>______________<br>(SIGN)<br>REFUSED . . . . 2<br>NOT PRESENT/OTHER . 3<br>(SKIP TO 130)	GRANTED . . . . 1<br>______________<br>(SIGN)<br>REFUSED . . . . 2<br>NOT PRESENT/OTHER . 3<br>(SKIP TO 130)
112	Has (NAME) had blood transfusion in the past 3 months?	YES . . . . 1<br>(SKIP TO 112C)<br>NO 2	YES . . . . 1<br>(SKIP TO 112C)<br>NO 2	YES . . . . 1<br>(SKIP TO 112C)<br>NO 2
112A	ASK CONSENT FOR GENOTYPE TEST FROM PARENT/OTHER ADULT.	As part of this survey, we are asking children all over the country to take a test to see if they have sickle cell anemia (SS) or its trait (AS). Sickle cell disease (SCD) is an inherited blood disorder, which affects children early in life often with repeated episodes of catastrophic illness and bone pains with varying periods of relative good health in between. This survey will assist the government to develop programs for the prevention and management of sickle cell disease.<br><br>We ask that all children born in 2013 or later take part in genotype testing in this survey and give a few drops of blood from a finger or heel. One blood drop will be tested for genotype immediately, and the result will be told to you right away. The equipment used to take the blood is clean and completely safe. It has never been used before and will be thrown away after each test. The result will be kept strictly confidential and will not be shared with anyone other than members of our survey team.<br><br>Do you have any questions? You can say yes or no. It is up to you to decide. Will you allow (NAME OF CHILD) to participate in the genotype test?		
112B	CIRCLE THE CODE AND SIGN YOUR NAME.	GRANTED . . . . 1<br>______________<br>(SIGN)<br>REFUSED . . . . 2<br>NOT PRESENT/OTHER . 3<br>(SKIP TO 130)	GRANTED . . . . 1<br>______________<br>(SIGN)<br>REFUSED . . . . 2<br>NOT PRESENT/OTHER . 3<br>(SKIP TO 130)	GRANTED . . . . 1<br>______________<br>(SIGN)<br>REFUSED . . . . 2<br>NOT PRESENT/OTHER . 3<br>(SKIP TO 130)
112C	ASK CONSENT FOR MALARIA TEST FROM PARENT/OTHER ADULT.	As part of this survey, we are asking children all over the country to take a test to see if they have malaria. Malaria is a serious illness caused by a parasite transmitted by a mosquito bite. This survey will assist the government to develop programs to prevent malaria.<br><br>We ask that all children born in 2013 or later take part in malaria testing in this survey and give a few drops of blood from a finger or heel. The blood will be tested for malaria immediately, and the result will be told to you right away. All results will be kept strictly confidential and will not be shared with anyone other than members of our survey team.<br><br>Do you have any questions?<br>You can say yes or no. It is up to you to decide.<br>Will you allow (NAME OF CHILD) to participate in the malaria test?		
112D	CIRCLE THE CODE, SIGN YOUR NAME, AND ENTER YOUR FIELDWORKER NUMBER.	GRANTED . . . . 1<br>REFUSED . . . . 2<br>______________<br>(SIGN AND ENTER YOUR FIELDWORKER NUMBER)	GRANTED . . . . 1<br>REFUSED . . . . 2<br>______________<br>(SIGN AND ENTER YOUR FIELDWORKER NUMBER)	GRANTED . . . . 1<br>REFUSED . . . . 2<br>______________<br>(SIGN AND ENTER YOUR FIELDWORKER NUMBER)

		CHILD 4	CHILD 5	CHILD 6
102	CHECK HOUSEHOLD QUESTIONNAIRE:<br>LINE NUMBER FROM COLUMN 11.	LINE NUMBER . . . . . . [ ][ ]<br>NAME ______	LINE NUMBER . . . . . . [ ][ ]<br>NAME ______	LINE NUMBER . . . . . . [ ][ ]<br>NAME ______

112E	PREPARE EQUIPMENT AND SUPPLIES ONLY FOR THE TEST(S) FOR WHICH CONSENT HAS BEEN OBTAINED AND PROCEED WITH THE TEST(S).			
112F	PLACE BAR CODE LABEL FOR MALARIA LAB TEST OR GENOTYPE CONFIRMATORY LAB TEST.	PUT THE 1ST BAR CODE LABEL HERE.<br>NOT PRESENT . . . 99994<br>REFUSED . . . . 99995<br>OTHER 99996	PUT THE 1ST BAR CODE LABEL HERE.<br>NOT PRESENT . . . 99994<br>REFUSED . . . . 99995<br>OTHER 99996	PUT THE 1ST BAR CODE LABEL HERE.<br>NOT PRESENT . . . . . 99994<br>REFUSED . . . . 99995<br>OTHER 99996
	MENTION WHERE BAR CODE PLACED BASED ON THE TEST.	SLIDE . . . . A<br>DBS . . . . B<br>TRANSMITTAL FORM . . . C	SLIDE . . . . A<br>DBS . . . . B<br>TRANSMITTAL FORM . . . C	SLIDE . . . . A<br>DBS . . . . B<br>TRANSMITTAL FORM . C
	**RESULTS OF HEMOGLOBIN TEST**			
113	RECORD HEMOGLOBIN LEVEL HERE AND IN THE ANEMIA AND MALARIA PAMPHLET.	G/DL . . . . . [ ][ ].[ ]<br>NOT PRESENT . . . . . 994<br>REFUSED . . . . 995<br>OTHER 996	G/DL . . . . . [ ][ ].[ ]<br>NOT PRESENT . . . . . 994<br>REFUSED . . . . 995<br>OTHER 996	G/DL . . . . . [ ][ ].[ ]<br>NOT PRESENT . . . . . 994<br>REFUSED . . . . 995<br>OTHER 996
	**RESULTS OF GENOTYPE TEST**			
113A	CIRCLE THE CODE FOR THE GENOTYPE RDT.	TESTED . . . . 1<br>NOT PRESENT . . . . . . 2<br>REFUSED . . . . 3<br>OTHER . . . . 6<br>(SKIP TO 114)	TESTED . . . . 1<br>NOT PRESENT . . . . . . 2<br>REFUSED . . . . 3<br>OTHER . . . . 6<br>(SKIP TO 114)	TESTED . . . . 1<br>NOT PRESENT . . . . 2<br>REFUSED . . . . 3<br>OTHER . . . . 6<br>(SKIP TO 114)
113B	RECORD THE RESULT OF THE GENOTYPE RDT HERE AND IN THE PAMPHLET.	AA . . . . 1<br>AS . . . . 2<br>AC . . . . 3<br>SC . . . . 4<br>(SKIP TO 114)<br>SS 5	AA . . . . 1<br>AS . . . . 2<br>AC . . . . 3<br>SC . . . . 4<br>(SKIP TO 114)<br>SS 5	AA . . . . 1<br>AS . . . . 2<br>AC . . . . 3<br>SC . . . . 4<br>(SKIP TO 114)<br>SS 5
113C	**SICKLE CELL ANEMIA (SS) REFERRAL**<br><br>RECORD THE RESULT OF THE GENOTYPE TEST ON THE REFERRAL FORM.	The genotype test shows that (NAME OF CHILD) has sickle cell anemia. Your child is very ill and must be taken to a health facility immediately.		
	**RESULTS OF MALARIA RDT TEST**			
114	CIRCLE THE CODE FOR THE MALARIA RDT.	TESTED . . . . 1<br>NOT PRESENT . . . . . . 2<br>REFUSED . . . . 3<br>OTHER . . . . 6<br>(SKIP TO 128)	TESTED . . . . 1<br>NOT PRESENT . . . . . . 2<br>REFUSED . . . . 3<br>OTHER . . . . 6<br>(SKIP TO 128)	TESTED . . . . 1<br>NOT PRESENT . . . . . . 2<br>REFUSED . . . . 3<br>OTHER . . . . 6<br>(SKIP TO 128)
115	RECORD THE RESULT OF THE MALARIA RDT HERE AND IN THE PAMPHLET.	POSITIVE . . . . 1<br>NEGATIVE . . . . 2<br>OTHER . . . . 6<br>(SKIP TO 128)	POSITIVE . . . . 1<br>NEGATIVE . . . . 2<br>OTHER . . . . 6<br>(SKIP TO 128)	POSITIVE . . . . 1<br>NEGATIVE . . . . 2<br>OTHER . . . . 6<br>(SKIP TO 128)

		CHILD 4	CHILD 5	CHILD 6
102	CHECK HOUSEHOLD QUESTIONNAIRE:<br>LINE NUMBER FROM COLUMN 11.	LINE NUMBER . . . . . . [ ][ ]<br>NAME ____________	LINE NUMBER . . . . . . [ ][ ]<br>NAME ____________	LINE NUMBER . . . . . . [ ][ ]<br>NAME ____________
118	Does (NAME) suffer from any of the following illnesses or symptoms:<br>a) Extreme weakness?<br>b) Heart problems?<br>c) Loss of consciousness?<br>d) Rapid or difficult breathing?<br>e) Seizures?<br>f) Abnormal bleeding?<br>g) Jaundice or yellow skin?<br>h) Dark urine?	YES NO<br>a) EXTREME WEAKNESS 1 2<br>b) HEART PROBLEMS 1 2<br>c) LOSS OF CONSCIOUS. 1 2<br>d) RAPID BREATHING 1 2<br>e) SEIZURES 1 2<br>f) BLEEDING 1 2<br>g) JAUNDICE 1 2<br>h) DARK URINE 1 2	YES NO<br>a) EXTREME WEAKNESS 1 2<br>b) HEART PROBLEMS 1 2<br>c) LOSS OF CONSCIOUS. 1 2<br>d) RAPID BREATHING 1 2<br>e) SEIZURES 1 2<br>f) BLEEDING 1 2<br>g) JAUNDICE 1 2<br>h) DARK URINE 1 2	YES NO<br>a) EXTREME WEAKNESS 1 2<br>b) HEART PROBLEMS 1 2<br>c) LOSS OF CONSCIOUS. 1 2<br>d) RAPID BREATHING 1 2<br>e) SEIZURES 1 2<br>f) BLEEDING 1 2<br>g) JAUNDICE 1 2<br>h) DARK URINE 1 2
119	CHECK 118:<br>ANY 'YES' CIRCLED?	NO [ ] YES [ ]<br>(SKIP TO 122)	NO [ ] YES [ ]<br>(SKIP TO 122)	NO [ ] YES [ ]<br>(SKIP TO 122)
120	CHECK 113:<br>HEMOGLOBIN RESULT	BELOW 8.0 G/DL, SEVERE ANEMIA . . . 1<br>(SKIP TO 122)<br>8.0 G/DL OR ABOVE . . . 2<br>NOT PRESENT . . . . . . 3<br>REFUSED . . . . 4<br>OTHER 6	BELOW 8.0 G/DL, SEVERE ANEMIA . . . 1<br>(SKIP TO 122)<br>8.0 G/DL OR ABOVE . . . 2<br>NOT PRESENT . . . . . . 3<br>REFUSED . . . . 4<br>OTHER 6	BELOW 8.0 G/DL, SEVERE ANEMIA . . . 1<br>(SKIP TO 122)<br>8.0 G/DL OR ABOVE . . . 2<br>NOT PRESENT . . . . . . 3<br>REFUSED . . . . 4<br>OTHER 6
121	In the past two weeks has (NAME) taken or is taking ACT given by a doctor or health center to treat the malaria?<br><br>VERIFY BY ASKING TO SEE TREATMENT	YES . . . . 1<br>(SKIP TO 123)<br>NO . . . . 2<br>(SKIP TO 124)	YES . . . . 1<br>(SKIP TO 123)<br>NO . . . . 2<br>(SKIP TO 124)	YES . . . . 1<br>(SKIP TO 123)<br>NO . . . . 2<br>(SKIP TO 124)
122	**SEVERE MALARIA REFERRAL**<br><br>RECORD THE RESULT OF THE MALARIA RDT ON THE REFERRAL FORM.	The malaria test shows that (NAME OF CHILD) has malaria. Your child also has symptoms of severe malaria. The malaria treatment I have will not help your child, and I cannot give you the medication. Your child is very ill and must be taken to a health facility right away.<br><br>**(SKIP TO 128)**		
123	ALREADY TAKING ACT REFERRAL STATEMENT	You have told me that (NAME OF CHILD) had already received ACT for malaria. Therefore, I cannot give you additional ACT. However, the test shows that he/she has malaria. If your child has a fever for two days after the last dose of ACT, you should take the child to the nearest health facility for further examination.<br><br>**(SKIP TO 130)**		
124	READ INFORMATION FOR MALARIA TREATMENT AND CONSENT STATEMENT TO PARENT/OTHER	The malaria test shows that your child has malaria. We can give you free medicine. The medicine is called ACT. ACT is very effective and in a few days it should get rid of the fever and other symptoms. You do not have to give the child the medicine. This is up to you. Please tell me whether you accept the medicine or not.		

		CHILD 4	CHILD 5	CHILD 6
102	CHECK HOUSEHOLD QUESTIONNAIRE:<br>LINE NUMBER FROM COLUMN 11.	LINE NUMBER . . . . . .<br>NAME ______	LINE NUMBER . . . . . .<br>NAME ______	LINE NUMBER . . . . . .<br>NAME ______
125	CIRCLE THE APPROPRIATE CODE AND SIGN YOUR NAME.	ACCEPTED MEDICINE . 1<br>______ (SIGN)<br>REFUSED . . . . 2<br>OTHER 6	ACCEPTED MEDICINE . 1<br>______ (SIGN)<br>REFUSED . . . . 2<br>OTHER 6	ACCEPTED MEDICINE . 1<br>______ (SIGN)<br>REFUSED . . . . 2<br>OTHER 6
126	CHECK 125:<br>MEDICATION ACCEPTED	ACCEPTED MEDICINE . 1<br>REFUSED . . . . 2<br>OTHER . . . . 6<br>(SKIP TO 130)	ACCEPTED MEDICINE . 1<br>REFUSED . . . . 2<br>OTHER . . . . 6<br>(SKIP TO 130)	ACCEPTED MEDICINE . 1<br>REFUSED . . . . 2<br>OTHER . . . . 6<br>(SKIP TO 130)
127	TREATMENT FOR CHILDREN WITH POSITIVE MALARIA TESTS	**TREATMENT WITH ACT**		
		**WEIGHT (in kg)**	**AGE**	**ARTEMETHER-LUMEFANTRINE**
		LESS THAN 5 KGS	NOTHING	NOTHING
		5-14 KGS	6 MONTHS - 3 YEARS	1 TABLET TWICE A DAY FOR 3 DAYS
		15-25 KGS	4 - 8 YEARS	2 TABLETS TWICE A DAY FOR 3 DAYS
		IF CHILD WEIGHS LESS THAN 5 KGS, DO NOT LEAVE DRUGS. TELL PARENTS TO TAKE CHILD TO HEALTH FACILITY.		
		ALSO TELL THE PARENT/OTHER ADULT: If [NAME] has a high fever, fast or difficult breathing, is not able to drink or breastfeed, gets sicker or does not get better in two days, you should take him/her to a health professional for treatment right away.<br>**(SKIP TO 130)**		
128	CHECK 113:<br>HEMOGLOBIN RESULT	BELOW 8.0 G/DL, SEVERE ANEMIA 1<br>8.0 G/DL OR ABOVE 2<br>NOT PRESENT 3<br>REFUSED 4<br>OTHER 6<br>(SKIP TO 130)	BELOW 8.0 G/DL, SEVERE ANEMIA 1<br>8.0 G/DL OR ABOVE 2<br>NOT PRESENT 3<br>REFUSED 4<br>OTHER 6<br>(SKIP TO 130)	BELOW 8.0 G/DL, SEVERE ANEMIA 1<br>8.0 G/DL OR ABOVE 2<br>NOT PRESENT 3<br>REFUSED 4<br>OTHER 6<br>(SKIP TO 130)
129	**SEVERE ANEMIA REFERRAL**<br>RECORD THE RESULT OF THE ANEMIA TEST ON THE REFERRAL FORM.	The anemia test shows that (NAME OF CHILD) has severe anemia. Your child is very ill and must be taken to a health facility immediately.		
130	GO BACK TO 103 IN NEXT COLUMN OF THIS QUESTIONNAIRE OR IN THE FIRST COLUMN OF AN ADDITIONAL QUESTIONNAIRE; IF NO MORE CHILDREN, GO TO 201.			

WEIGHT, HEIGHT, AND HEMOGLOBIN MEASUREMENT FOR WOMEN AGE 15-49

201	CHECK COLUMN 9 IN HOUSEHOLD QUESTIONNAIRE. RECORD THE LINE NUMBER, NAME, AGE, AND MARITAL STATUS FOR ALL ELIGIBLE WOMEN IN 202, 203, AND 204.<br>IF THERE ARE MORE THAN THREE WOMEN, USE ADDITIONAL QUESTIONNAIRE(S).			
		WOMAN 1	WOMAN 2	WOMAN 3
202	CHECK HOUSEHOLD QUESTIONNAIRE:<br>LINE NUMBER FROM COLUMN 9.<br>NAME FROM COLUMN 2.	LINE NUMBER . . . . ☐☐<br>NAME ________	LINE NUMBER . . . . ☐☐<br>NAME ________	LINE NUMBER . . . . ☐☐<br>NAME ________
203	CHECK HOUSEHOLD QUESTIONNAIRE COLUMN 7 (AGE):	15-17 YEARS . . . . 1<br>18-49 YEARS 2	15-17 YEARS . . . . 1<br>18-49 YEARS 2	15-17 YEARS . . . . 1<br>18-49 YEARS 2
204	CHECK HOUSEHOLD QUESTIONNAIRE COLUMN 8 (MARITAL STATUS):	CODE 4 (NEVER IN UNION) . 1<br>OTHER 2	CODE 4 (NEVER IN UNION) . 1<br>OTHER 2	CODE 4 (NEVER IN UNION) . 1<br>OTHER 2
205	WEIGHT IN KILOGRAMS.	KG. . . . ☐☐☐.☐☐<br>NOT PRESENT . . . . . . 99994<br>REFUSED . . . . 99995<br>OTHER 99996	KG. . . . ☐☐☐.☐☐<br>NOT PRESENT . . . . . . 99994<br>REFUSED . . . . 99995<br>OTHER 99996	KG. . . . ☐☐☐.☐☐<br>NOT PRESENT . . . . . . 99994<br>REFUSED . . . . 99995<br>OTHER 99996
206	HEIGHT IN CENTIMETERS.	CM. . . . . . . ☐☐☐.☐<br>NOT PRESENT . . . . 9994<br>REFUSED . . . . .9995<br>OTHER 9996	CM. . . . . . . ☐☐☐.☐<br>NOT PRESENT . . . . 9994<br>REFUSED . . . . .9995<br>OTHER 9996	CM. . . . . . . ☐☐☐.☐<br>NOT PRESENT . . . . 9994<br>REFUSED . . . . .9995<br>OTHER 9996
207	MEASURER: ENTER YOUR FIELDWORKER NUMBER.	☐☐☐☐<br>FIELDWORKER NUMBER	☐☐☐☐<br>FIELDWORKER NUMBER	☐☐☐☐<br>FIELDWORKER NUMBER
208	CHECK 203: AGE	15-17 YEARS . . . . 1<br>18-49 YEARS . . . . 2<br>(SKIP TO 210) ←	15-17 YEARS . . . . 1<br>18-49 YEARS . . . . 2<br>(SKIP TO 210) ←	15-17 YEARS . . . . 1<br>18-49 YEARS . . . . 2<br>(SKIP TO 210) ←
209	CHECK 204: MARITAL STATUS	CODE 4 (NEVER IN UNION) . 1<br>(SKIP TO 216) ←<br>OTHER 2	CODE 4 (NEVER IN UNION) . 1<br>(SKIP TO 216) ←<br>OTHER 2	CODE 4 (NEVER IN UNION) . 1<br>(SKIP TO 216) ←<br>OTHER 2

WEIGHT, HEIGHT, AND HEMOGLOBIN MEASUREMENT FOR WOMEN AGE 15-49

		WOMAN 1	WOMAN 2	WOMAN 3
	NAME FROM COLUMN 2.	NAME ____________	NAME ____________	NAME ____________

ADULT RESPONDENT CONSENT FOR ANEMIA TEST

(ADULT RESPONDENT CONSENT)

210	ASK CONSENT FOR ANEMIA TEST.	As part of this survey, we are asking people all over the country to take an anemia test. Anemia is a serious health problem that usually results from poor nutrition, infection, or chronic disease. This survey will assist the government to develop programs to prevent and treat anemia.<br><br>For the anemia testing, we will need a few drops of blood from a finger. The equipment used to take the blood is clean and completely safe. It has never been used before and will be thrown away after we take your blood. The blood will be tested for anemia immediately, and the result will be told to you right away. The result will be kept strictly confidential and will not be shared with anyone other than members of our survey team.<br><br>Do you have any questions?<br>You can say yes or no. It is up to you to decide.<br>Will you take the anemia test?		
211	CIRCLE THE CODE AND SIGN YOUR NAME.	GRANTED . . . . 1<br>RESPONDENT REFUSED . . . 2<br>____________<br>(SIGN)<br>(IF REFUSED, SKIP TO 233)<br>NOT PRESENT/OTHER . . . . . 3<br>(SKIP TO 233)	GRANTED . . . . 1<br>RESPONDENT REFUSED . . . 2<br>____________<br>(SIGN)<br>(IF REFUSED, SKIP TO 233)<br>NOT PRESENT/OTHER . . . . . 3<br>(SKIP TO 233)	GRANTED . . . . 1<br>RESPONDENT REFUSED . . . 2<br>____________<br>(SIGN)<br>(IF REFUSED, SKIP TO 233)<br>NOT PRESENT/OTHER . . . . . 3<br>(SKIP TO 233)
211A	CHECK 226 IN WOMAN'S QUESTIONNAIRE OR ASK:<br>Are you pregnant?	YES . . . . 1<br>NO . . . . 2<br>DON'T KNOW . . . . 8<br>(SKIP TO 229)	YES . . . . 1<br>NO . . . . 2<br>DON'T KNOW . . . . 8<br>(SKIP TO 229)	YES . . . . 1<br>NO . . . . 2<br>DON'T KNOW . . . . 8<br>(SKIP TO 229)

216	NAME AND RELATIONSHIP TO THE ADOLESCENT OF ADULT RESPONSIBLE FOR THEM. GET LINE NUMBER FROM COLUMN 1 OF HOUSEHOLD SCHEDULE.	NAME ____________<br>RELATIONSHIP TO THE ADOLESCENT<br>____________<br>[ ][ ]<br>(RECORD '00' IF NOT LISTED)	NAME ____________<br>RELATIONSHIP TO THE ADOLESCENT<br>____________<br>[ ][ ]<br>(RECORD '00' IF NOT LISTED)	NAME ____________<br>RELATIONSHIP TO THE ADOLESCENT<br>____________<br>[ ][ ]<br>(RECORD '00' IF NOT LISTED)

PARENTAL/RESPONSIBLE ADULT CONSENT FOR ANEMIA TEST

(PARENT — RESP ADULT CONSENT)

217	ASK CONSENT FOR ANEMIA TEST FROM PARENT/ADULT.	As part of this survey, we are asking people all over the country to take an anemia test. Anemia is a serious health problem that usually results from poor nutrition, infection, or chronic disease. This survey will assist the government to develop programs to prevent and treat anemia.<br><br>For the anemia testing, we will need a few drops of blood from a finger. The equipment used to take the blood is clean and completely safe. It has never been used before and will be thrown away after each test. The blood will be tested for anemia immediately, and the result will be told to you and (NAME OF MINOR) right away. The result will be kept strictly confidential and will not be shared with anyone other than members of our survey team.<br><br>Do you have any questions?<br>You can say yes or no. It is up to you to decide.<br>Will you allow (NAME OF MINOR) to take the anemia test?		
218	CIRCLE THE CODE AND SIGN YOUR NAME.	GRANTED . . . . 1<br>PARENT/OTHER RESPONSIBLE ADULT REFUSED . . . . . . 2<br>____________<br>(SIGN)<br>(IF REFUSED, SKIP TO 233)<br>NOT PRESENT/OTHER . . . . . 3<br>(SKIP TO 233)	GRANTED . . . . 1<br>PARENT/OTHER RESPONSIBLE ADULT REFUSED . . . . . . 2<br>____________<br>(SIGN)<br>(IF REFUSED, SKIP TO 233)<br>NOT PRESENT/OTHER . . . . . 3<br>(SKIP TO 233)	GRANTED . . . . 1<br>PARENT/OTHER RESPONSIBLE ADULT REFUSED . . . . . . 2<br>____________<br>(SIGN)<br>(IF REFUSED, SKIP TO 233)<br>NOT PRESENT/OTHER . . . . . 3<br>(SKIP TO 233)

WEIGHT, HEIGHT, AND HEMOGLOBIN MEASUREMENT FOR WOMEN AGE 15-49

		WOMAN 1	WOMAN 2	WOMAN 3
	NAME FROM COLUMN 2.	NAME ____________	NAME ____________	NAME ____________

M I N O R R E S P O N D E N T C O N S E N T F O R A N E M I A T E S T

MINOR RESPONDENT CONSENT

219	ASK CONSENT FOR ANEMIA TEST FROM RESPONDENT.	As part of this survey, we are asking people all over the country to take an anemia test. Anemia is a serious health problem that usually results from poor nutrition, infection, or chronic disease. This survey will assist the government to develop programs to prevent and treat anemia.<br><br>For the anemia testing, we will need a few drops of blood from a finger. The equipment used to take the blood is clean and completely safe. It has never been used before and will be thrown away after we take your blood. The blood will be tested for anemia immediately, and the result will be told to you and (NAME OF PARENT/RESPONSIBLE ADULT) right away. The result will be kept strictly confidential and will not be shared with anyone other than members of our survey team.<br><br>Do you have any questions?<br>You can say yes or no. It is up to you to decide.<br>Will you take the anemia test?		
220	CIRCLE THE CODE AND SIGN YOUR NAME.	GRANTED . . . . 1<br>MINOR RESPONDENT REFUSED . . . . 2<br>____________<br>(SIGN)<br>(IF REFUSED, SKIP TO 233)<br>NOT PRESENT/OTHER . . . . . 3<br>(SKIP TO 233)	GRANTED . . . . 1<br>MINOR RESPONDENT REFUSED . . . . 2<br>____________<br>(SIGN)<br>(IF REFUSED, SKIP TO 233)<br>NOT PRESENT/OTHER . . . . . 3<br>(SKIP TO 233)	GRANTED . . . . 1<br>MINOR RESPONDENT REFUSED . . . . 2<br>____________<br>(SIGN)<br>(IF REFUSED, SKIP TO 233)<br>NOT PRESENT/OTHER . . . . . 3<br>(SKIP TO 233)
220A	CHECK 226 IN WOMAN'S QUESTIONNAIRE OR ASK:<br>Are you pregnant?	YES . . . . 1<br>NO . . . . 2<br>DON'T KNOW 8	YES . . . . 1<br>NO . . . . 2<br>DON'T KNOW 8	YES . . . . 1<br>NO . . . . 2<br>DON'T KNOW 8

229	PREPARE EQUIPMENT AND SUPPLIES ONLY FOR THOSE FOR WHICH CONSENT HAS BEEN OBTAINED AND PROCEED WITH THE TEST.			
231	RECORD HEMOGLOBIN LEVEL HERE AND IN ANEMIA PAMPHLET.	G/DL . . . . [ ][ ].[ ]<br>NOT PRESENT . . . . 994<br>REFUSED . . . . 995<br>OTHER 996	G/DL . . . . [ ][ ].[ ]<br>NOT PRESENT . . . . 994<br>REFUSED . . . . 995<br>OTHER 996	G/DL . . . . [ ][ ].[ ]<br>NOT PRESENT . . . . 994<br>REFUSED . . . . 995<br>OTHER 996
233	GO BACK TO 202 IN NEXT COLUMN OF THIS QUESTIONNAIRE OR IN THE FIRST COLUMN OF AN ADDITIONAL QUESTIONNAIRE; IF NO MORE WOMEN, END INTERVIEW.			

FIELDWORKER'S OBSERVATIONS

TO BE FILLED IN AFTER COMPLETING BIOMARKERS

SUPERVISOR'S OBSERVATIONS

EDITOR'S OBSERVATIONS

6 Mar 2018

NIGERIA DEMOGRAPHIC AND HEALTH SURVEY 2018
FIELDWORKER QUESTIONNAIRE

NIGERIA
NATIONAL POPULATION COMMISSION

LANGUAGE OF QUESTIONNAIRE ENGLISH

NO.	QUESTIONS AND FILTERS	CODING CATEGORIES	SKIP
100	What is your name?	NAME ________	
101	RECORD FIELDWORKER NUMBER	NUMBER . . . . [ ][ ][ ][ ]	

INSTRUCTIONS

Information on all Nigeria DHS field workers is collected as part of the Nigeria DHS survey. Please fill out the questions below. The information you provide will be part of the survey data file; however, your name will be removed and will not be part of the data file. Thank you for providing the information needed.

NO.	QUESTIONS AND FILTERS	CODING CATEGORIES	SKIP
102	In what state do you live?	ABIA . . . 01<br>ADAMAWA . . . 02<br>AKWA IBOM . . . 03<br>ANAMBRA . . . 04<br>BAUCHI . . . 05<br>BAYELSA . . . 06<br>BENUE . . . 07<br>BORNO . . . 08<br>CROSS RIVER . . . 09<br>DELTA . . . 10<br>EBONYI . . . 11<br>EDO . . . 12<br>EKITI . . . 13<br>ENUGU . . . 14<br>FCT-ABUJA . . . 15<br>GOMBE . . . 16<br>IMO . . . 17<br>JIGAWA . . . 18<br>KADUNA . . . 19<br>KANO . . . 20<br>KATSINA . . . 21<br>KEBBI . . . 22<br>KOGI . . . 23<br>KWARA . . . 24<br>LAGOS . . . 25<br>NASARAWA . . . 26<br>NIGER . . . 27<br>OGUN . . . 28<br>ONDO . . . 29<br>OSUN . . . 30<br>OYO . . . 31<br>PLATEAU . . . 32<br>RIVERS . . . 33<br>SOKOTO . . . 34<br>TARABA . . . 35<br>YOBE . . . 36<br>ZAMFARA . . . 37	
103	Do you live in a city, town, or rural area?	CITY . . . 1<br>TOWN . . . 2<br>RURAL . . . 3	

NO.	QUESTIONS AND FILTERS	CODING CATEGORIES	SKIP
104	How old are you?<br>RECORD AGE IN COMPLETED YEARS.	AGE . . . . [ ][ ]	
105	Are you male or female?	MALE . . . . 1<br>FEMALE 2	
106	What is your current marital status?	CURRENTLY MARRIED . . . . 1<br>LIVING WITH A MAN/WOMAN . . . . 2<br>WIDOWED . . . . 3<br>DIVORCED . . . . 4<br>SEPARATED . . . . 5<br>NEVER MARRIED OR LIVED<br>WITH A MAN/WOMAN 6	
107	How many living children do you have?<br>INCLUDE ONLY CHILDREN WHO ARE YOUR BIOLOGICAL CHILDREN.	LIVING<br>CHILDREN . . . . [ ][ ]	
108	Have you ever had a child who died?	YES . . . . 1<br>NO 2	
109	What is the highest level of school you attended: primary, secondary, or higher?	PRIMARY . . . . 1<br>SECONDARY . . . . 2<br>HIGHER 3	
110	What is the highest class/year you completed at that level?<br>IF COMPLETED LESS THAN ONE YEAR AT THAT LEVEL, RECORD '00'.	CLASS/YEAR . . . . [ ][ ]	
111	What is your religion?	CATHOLIC . . . . 01<br>OTHER CHRISTIAN . . . . 02<br>ISLAM . . . . 03<br>TRADITIONALIST . . . . 04<br><br>NO RELIGION . . . . 95<br><br>OTHER ______________________________ 96<br>(SPECIFY)	
112	What is your ethnicity?	ETHNICITY ____________________ [ ][ ]	
113	What languages can you speak?<br><br>RECORD ALL LANGUAGES YOU CAN SPEAK.	ENGLISH . . . . A<br>HAUSA . . . . B<br>YORUBA . . . . C<br>IGBO . . . . D<br>URHOBO . . . . E<br>IBIBIO . . . . F<br>EDO . . . . G<br>FULFULDE . . . . H<br>KANURI . . . . I<br><br>OTHER ______________________________ X<br>(SPECIFY)	

NO.	QUESTIONS AND FILTERS	CODING CATEGORIES	SKIP
114	What is your mother tongue/native language (language spoken at home growing up)?	ENGLISH . . . . . 01<br>HAUSA . . . . . 02<br>YORUBA . . . . . 03<br>IGBO . . . . . 04<br>URHOBO . . . . . 05<br>IBIBIO . . . . . 06<br>EDO . . . . . 07<br>FULFULDE . . . . . 08<br>KANURI . . . . . 09<br>OTHER ______ 96<br>(SPECIFY)	
115	Have you ever worked on a DHS survey prior to this one?	YES . . . . . 1<br>NO 2	
116	Have you ever worked on any other survey prior to this one (not a DHS)?	YES . . . . . 1<br>NO 2	
117	Were you already working for the National Population Commission (NPC) at the time you were employed to work on this DHS?	YES . . . . . 1<br>NO 2	 → 119
118	Are you a permanent or temporary employee of the National Population Commission (NPC)?	PERMANENT . . . . . 1<br>TEMPORARY 2	
119	If you have comments, please write them here.		
